# Et_2_Zn‐Mediated Gem‐Dicarboxylation of Cyclopropanols with CO_2_

**DOI:** 10.1002/advs.202307633

**Published:** 2023-12-21

**Authors:** Hongjian Liu, Lei Shi, Xiaobin Tan, Bangxiong Kang, Gen Luo, Huanfeng Jiang, Chaorong Qi

**Affiliations:** ^1^ Key Laboratory of Functional Molecular Engineering of Guangdong Province School of Chemistry and Chemical Engineering South China University of Technology Guangzhou 510640 China; ^2^ Institutes of Physical Science and Information Technology Anhui University Hefei 230601 China

**Keywords:** carbon dioxide, cyclopropanols, dicarboxylation, malonic acids

## Abstract

An unprecedented Et_2_Zn‐mediated gem‐dicarboxylation of C─C/C─H single bond of cyclopropanols with CO_2_ is disclosed, which provides a straightforward and efficient methodology for the synthesis of a variety of structurally diverse and useful malonic acids in moderate to excellent yields. The protocol features mild reaction conditions, excellent functional group compatibility, broad substrate scope, and facile derivatization of the products. DFT calculations confirm that the transition‐metal‐free transformation proceeds through a novel ring‐opening/*α*‐functionalization/ring‐closing/ring‐opening/*β*‐functionalization (ROFCOF) process, and 1,8‐diazabicyclo[5.4.0]undec‐7‐ene (DBU) plays dual important roles in the transformation.

## Graphical abstract

A zinc‐mediated gem‐dicarboxylation of cyclopropanols with CO_2_ is reported. The reaction proceeds through a novel ring‐opening/α‐functionalization/ring‐closing/ring‐opening/β‐functionalization (ROFCOF) process, providing a direct and efficient method for the synthesis of a variety of useful malonic acid derivatives. Mild conditions, excellent functional group compatibility and broad scope are the features of the protocol.

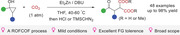

## 1 Introduction

Carbon dioxide (CO_2_), a main greenhouse gas, has been regarded as an ideal C1 feedstock in organic synthesis because it is nontoxic, abundant, inexpensive, and renewable.^[^ ^1^ ^]^ In this context, the synthesis of carboxylic acids via carboxylation reaction with CO_2_ as the carbonyl source has attracted great interest from chemists,^[^ ^2^ ^]^ because carboxylic acids are widely used as important synthetic intermediates and key precursors for polymers, and are also privileged motifs found in numerous bioactive natural products and pharmaceuticals.^[^ ^3^ ^]^ Notably, in the past decades, with great effort from many groups, significant progress has been made in dicarboxylation to generate dicarboxylic acids via transition metal catalysis,^[^ ^4^ ^]^ photocatalysis,^[^ ^5^ ^]^ or electrochemistry.^[^ ^6^ ^]^ However, most of the methods involve dicarboxylation of C─C multiple bonds of unsaturated hydrocarbons, and in sharp contrast, the example of direct dicarboxylation of C─C or C─H single bonds is rare. In 2022, Yu and co‐workers elegantly developed an electrochemical ring‐opening dicarboxylation of C─C single bonds in strained rings with CO_2_, providing efficient routes to a variety of glutaric acid and adipic acid derivatives from cyclopropanes and cyclobutanes.^[^ ^6e^ ^]^ To the best of our knowledge, direct dicarboxylation of C─C or C─H single bond to construct useful malonic acids has not yet been reported.^[^ ^4^, ^5^ ^]^ Thus, the development of novel strategies for direct and efficient synthesis of diacids including malonic acids and their derivatives via dicarboxylation of C─C or C─H bond under mild conditions is still highly desirable due to their widespread application in organic synthesis.

**Scheme 1:**
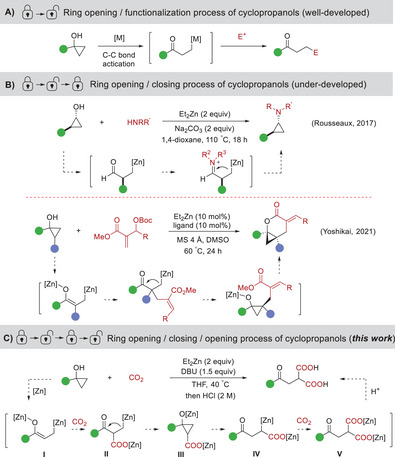
Reaction modes of cyclopropanols for the construction of valuable molecules.

Recently, cyclopropanols have been extensively investigated as versatile synthons in C─C or C‐heteratom bond‐forming reactions for the construction of an array of value‐added organic compounds.^[^ ^7^ ^]^ One of the most frequently used reaction mode of cyclopropanols is metal‐catalyzed or mediated ring‐opening/functionalization (ROF) via a metal homoenolate intermediate (**Scheme** **1**A). The ROF reaction mode has become a powerful platform for the synthesis of a range of *β*‐substituted ketones.^[^ ^7^, ^8^ ^]^ Interestingly, a new ring‐opening/functionalization/ ring‐closing (ROFC) reaction mode has recently been developed as a potential strategy for the synthesis of other valuable cyclopropane derivatives.^[^ ^9^ ^]^ The pioneering work was reported by Rousseaux and co‐workers, in which they developed an elegant method for converting cyclopropanols into valuable *trans*‐cyclopropylamines (Scheme 1B, upper).^[^ ^9a^ ^]^ This ROFC reaction involved in situ generation of zinc homoenolates, condensation with amines, and subsequent ring closure processes. Very recently, Yoshikai et al. reported a zinc‐catalyzed *β*‐allylation of cyclopropanols with Morita‐Baylis‐Hillmancarbonates, affording bicyclic cyclopropane derivatives in a diastereoselective fashion. This transformation also underwent a ROFC process, but involved a bis‐nucleophilic zinc enolate‐homoenolate species (Scheme 1B, bottom).^[^ ^9b^ ^]^

Inspired by the above significant achievements and as our continued interest in the fixation of CO_2_,^[^ ^6^, ^10^ ^]^ we wondered whether we could develop an efficient dicarboxylation of cyclopropanols with CO_2_ via a ring‐opening/functionalization/ring‐closing/ring‐opening/ functionalization (ROFCOF) process. We envisioned that cyclopropanols might undergo ring‐opening in the presence of zinc salts to yield a zinc enolate‐homoenolate species **I**, which would react with CO_2_ to give carboxylate **II**. The ring‐closing of **II** then results in the formation of intermediate **III**. Subsequent ring‐opening of intermediate **III** would generate alkylzinc species **IV**. The insertion of another CO_2_ to the C─Zn bond of **IV** would deliver dicarboxylic acid salt **V**, which would give final diacid products after hydrolysis (Scheme 1C). If successful, this strategy would provide an attractive route to a variety of malonic acid derivatives through dicarboxylation of C─C/C─H single bond of cyclopropanols. However, such a scenario faces several challenges. First, in the presence of znic salts, cyclopropanols might generate zinc homoenolate species,^[^ ^7^, ^8^, ^9^ ^]^ which would undergo protonation or monocarboxylation with CO_2_ to give ketone or monocarboxylic acid products, rendering the chemoselective dicarboxylation particularly problematic. Second, the site‐selective insertion of multiple CO_2_ units might be another challenge. For example, the nucleophilicity of the carbon─zinc bond of the intermediate **II** would lead to the formation of succinic acid salts via direct insertion of another CO_2_ instead of ring‐closing to give intermediate **III**.^[^ ^11^ ^]^

## 2 Results and Discussion

With this idea in mind, we initiated our investigations by using 1‐phenylcyclopropanol (**1a**) as the model substrate to react with CO_2_ under different conditions (**Table** **1**). When the reaction was conducted in the presence of two equivalents of Et_2_Zn without any base in dry tetrahydrofuran (THF) at 40 °C, no desired malonic acid derivative 2 was formed and only 14% of propiophenone was obtained as the main product (entry 1). To our delight, the reaction could proceed smoothly to give the product **2** in 81% yield by adding 1 equivalent of DBU (entry 2). A control experiment showed that both zinc salt and base are essential (entry 3). Other organic or inorganic bases, including Et_3_N, 1,4‐diazabicyclo[2.2.2]octane (DABCO), DBU, 1,1,3,3‐tetramethylguanidine (TMG), and K_2_CO_3_, were also investigated, but no better results were observed (entries 4–7). Replacing Et_2_Zn with other zinc salts such as Me_2_Zn, Zn(CN)_2_, and ZnCl_2_ led to no detectable yield of **2** (entries 8–10). Pleasingly, product **2** was obtained in 96% yield upon isolation by increasing the amount of DBU to 1.5 equivalents and extending reaction time to 14 h (entry 11). It was also showed that two equivalents of Et_2_Zn were necessary to obtain quantitative yield since decreasing the amount of Et_2_Zn to 1.5 equivalent resulted in a low yield (entry 12). The reaction temperature and solvent also have great impact on the transformation, and 40 °C and THF were proved to be optimal (Tables S1–S4, Supporting Information).

**Table 1:** Optimization of the reaction conditions.

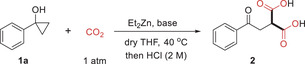				
Entry^a)^	Zn source (equivalent)	Base (equivalent)	t [h]	Yield [%]^b)^
1	Et_2_Zn (2)	–	12	n.d.
2	Et_2_Zn (2)	DBU (1)	12	81
3	–	DBU (1)	12	n.d.
4	Et_2_Zn (2)	Et_3_N (1)	12	26
5	Et_2_Zn (2)	DABCO (1)	12	33
6	Et_2_Zn (2)	TMG (1)	12	64
7	Et_2_Zn (2)	K_2_CO_3_ (1)	12	trace
8	Me_2_Zn (2)	DBU (1)	12	n.d.
9	Zn (CN)_2_ (2)	DBU (1)	12	n.d.
10	ZnCl_2_ (2)	DBU (1)	12	n.d.
11^c)^	Et_2_Zn (2)	DBU (1.5)	14	99 (96)^d)^
12	Et_2_Zn (1.5)	DBU (1.5)	14	64

^a)^ Reaction conditions: **1** (0.3 mmol), Et_2_Zn (2.0 equivalents), CO_2_ (1 atm), dry THF (2 mL), 40 °C, 12 h; then acidification with HCl (2 m);

^b)^ Yields were determined by 1H‐NMR with CH2Br2 as internal standard;

^c)^ 14 h;

^d)^ Isolated yield.

**Scheme 2:**
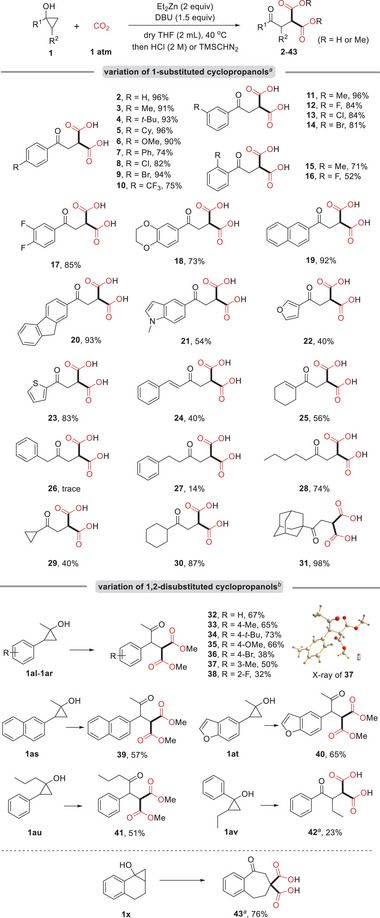
Substrate scope for gem‐dicarboxylation of cyclopropanols. a) Reaction conditions: cyclopropanol (0.3 mmol), Et_2_Zn (2 equivalents), CO_2_ (1 atm), DBU (1.5 equivalents), dry THF (2 mL), 40 °C, 14 h; then acidification with HCl (2 m). Isolated yields were reported. b) DBU (1.5 equivalents), 60 °C, 20 h; then esterification with TMSCHN_2_ before separation.

Having determined the optimal reaction conditions, we then investigate the generality and limitation of the dicarboxylation, and the results are summarized in **Scheme** **2**. To our delight, a variety of 1‐aryl‐substituted cyclopropanols could undergo the reaction to give the corresponding malonic acids **2**−**16** in moderate to excellent yields. Both electron‐donating groups (Me, *t*‐Bu, Cy, and OMe) and electron‐withdrawing groups (Ph, Cl, Br, and CF_3_) at the *para‐*, *meta‐*, and *ortho‐*position of the benzene ring were well tolerated under the dicarboxylation reaction. However, the *ortho*‐substituted substrates gave the products in lower yields than their *para*‐ and *meta*‐substituent analogues (**15** vs **3** and **11**), which might be due to the steric effect. The disubstituted substrate 1‐(3,4‐difluorophenyl)cyclopropan‐1‐ol worked well to give the corresponding product **17** in 85% yield. Cyclopropanols containing fused or heteroaryl rings were applicable to the reaction, giving the desired products **18**−**23** in satisfactory to excellent yields. Pleasingly, challenging alkenyl‐substituted substrates such as (*E*)−1‐styrylcyclopropan‐1‐ol and 1‐(cyclohex‐1‐en‐1‐yl)cyclopropan‐1‐ol could also undergo the reaction smoothly, giving the dicarboxylic acids **24** and **25** in 40% and 56% yield, respectively. Notably, 1‐alkyl‐substituted cyclopropanols uneventfully took part in the double carboxylation to give rise to the corresponding products **28**−**31** in moderate to near‐quantitative yields. Encouraged by the above results, we then investigated unsymmetrical 1,2‐disubstituted cyclopropanols. A broad range aryl and alkyl groups in cyclopropanols were found to be compatible with the zinc‐mediated dicarboxylation reaction to afford the desired malonic acid derivatives **32**–**42** although higher reaction temperature and longer reaction time were required in order to obtain satisfactory yields of the products in these cases. It is noteworthy that the reaction is highly regioselective, as the dicarboxylation occurred exclusively at the secondary rather than the tertiary carbon of the cyclopropanols. The structure of **37** was unambiguously confirmed by X‐ray diffraction analysis.^[^ ^12^ ^]^ Interestingly, the bicyclic substrate 1,1a,2,3‐tetrahydro‐7b*H*‐cyclopropa[*a*]naphthalen‐7b‐ol was also able to participate in the reaction, and the seven‐membered cyclic product **43** was predominantly formed in a high yield.^[^ ^13^ ^]^

**Scheme 3:**
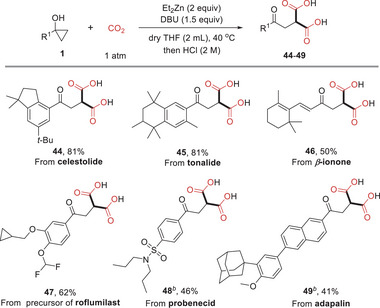
Applications of natural products and drug molecules: a) Reaction conditions: cyclopropanol (0.3 mmol), Et_2_Zn (2 equivalents), CO_2_ (1 atm), DBU (1.5 equivalents), dry THF (2 mL), 40 °C, 14 h; then acidification with HCl (2 m). isolated yields. b) 45 °C, 24 h.

The present method can be employed for late‐stage functionalization of complex molecules derived from natural products and drugs. As can be seen from **Scheme** **3**, cyclopropanols derived from perfume molecules such as celestolide, fixolide, and *β*‐ionone could undergo the transformation smoothly, yielding the desired products **44**–**46** in moderate to high yields. Moreover, cyclopropanols derived from the precursor of selective PDE4 inhibitor roflumilast, anti‐gout drug probenecid, and retinoic acid compound adapalin were compatible with this system, affording the expected products **47**, **48**, and **49** in 62%, 46%, and 41% yield, respectively.

**Scheme 4:**
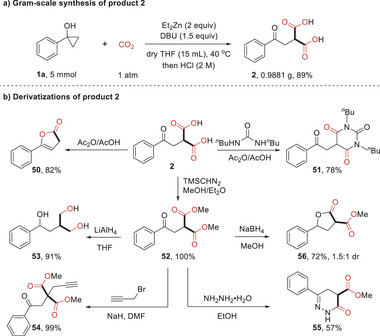
Gram‐scale synthesis and derivatizations of 2.

To showcase the practicability of the protocol, a gram‐scale synthesis of product **2** was performed under the standard conditions, which readily gave access to the product in 89% yield (**Scheme** **4**a). The derivatizations of product **2** were also investigated to demonstrate the utilities of this method (Scheme 2b). First, in the presence of acetic anhydride, **2** could undergo decarboxylative cyclization to give the unsaturated *γ*‐lactone **50** in 82% yield.^[^ ^14^ ^]^ The reaction of **2** and 1,3‐dibutylurea under acidic conditions gave barbituric acid derivative **51**.^[^ ^14^ ^]^ Moreover, the esterification of **2** with TMSCH_2_N_2_ furnished ester **52** in quantitative yield,^[^ ^15^ ^]^ which could be reduced to triol **53** by Lithium aluminohydride.^[^ ^16^ ^]^ Treatment of **52** with propargyl bromide under basic conditions, propargylated product **54** was obtained in almost quantitative yield.^[^ ^17^ ^]^ Furthermore, compound **52** could be used for the construction of a variety of heterocycles. For instance, condensation of **52** with hydrazine hydrate afforded the corresponding tetrahydropyridazine derivative **55**,^[^ ^18^ ^]^ while the reduction cyclization of **52** using NaBH_4_ as the reductant would yield *γ*‐lactone **56**.^[^ ^19^ ^]^

**Scheme 5:**
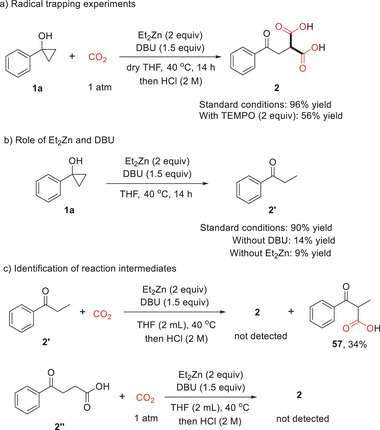
Mechanistic studies.

To gain some insight into the reaction pathway, we first performed the model reaction in the presence of 2 equivalents of TEMPO as the radical scavengers, and **2** could be obtained in 56% yield (**Scheme** **5**a). Second, to probe the role of Et_2_Zn and DBU in this transformation, phenylcyclopropanol **1a** was treated in the absence of CO_2_ under otherwise standard conditions, and ethyl phenyl ketone **2′** was formed in 90% yield. However, the reaction without DBU or Et_2_Zn only gave **2′** in very low yields, indicating the synergistic effect between DBU and Et_2_Zn in the ring opening of **1a** (Scheme 5b). Third, neither **2′** nor 4‐oxo‐4‐phenylbutanoic acid (**2″**) could undergo carboxylation with CO_2_ under standard conditions to give the desired product **2**, excluding the possibility of **2′** and **2″** as the key intermediates for the transformation (Scheme 5c).

**Figure 1:**
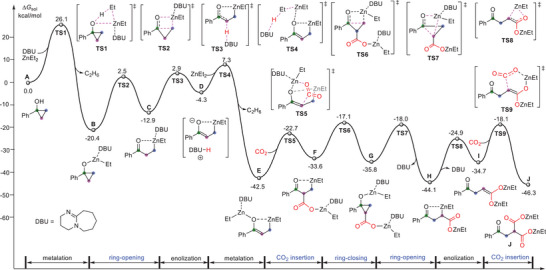
Energy profiles of Et_2_Zn‐mediated gem‐dicarboxylation of 1‐phenylcyclopropanol a) with CO_2_ calculated at the level of M06‐2X(SMD,THF)/6‐311+G(d,p)//M06‐2X/6‐31G(d).

To further elucidate the mechanism of the reaction, density functional theory (DFT) calculations were conducted on the model reaction of 1‐phenylcyclopropanol (**1a**) with CO_2_. The most favorable pathway is shown in **Figure** **1** for mechanism discussion (see Supporting Information for more details). First, the metalation of **1a** by ZnEt_2_ can take place with the aid of a molecule of DBU as a ligand to give an intermediate **B**. This process has an energy barrier of 26.1 kcal mol^−1^ (via **TS1**) and is significantly exothermic by 20.4 kcal mol^−1^. The ring‐opening of cyclopropane moiety in **B** can subsequently occur through **TS2** (∆*G* ^‡^ = 22.9 kcal mol^−1^) to form intermediate **C**. It is found that the direct insertion of CO_2_ into the Zn─C bond in **C** needs to overcome high energy barrier (∆*G* ^‡^ = 34.7 kcal mol^−1^, via **TS3ʹʹ** in Figure S1‐1, Supporting Information), which is kinetically inaccessible under the experimental conditions. Alternatively, the intermediate **C** could be easily converted to an ion‐pair species **D** via the deprotonation of the methylene (**TS3**) by DBU base. Then, the proton transfer from DBU‐H^+^ to a new incoming ZnEt_2_ through **TS4** could result in a dizinc enolate‐homoenolate species **E**, which is significantly more stable than **D** by 38.2 kcal mol^−1^. To be noted, CO_2_ insertion into **E** through six‐membered ring transition state **TS5** can take place with a moderate energy (∆*G* ^‡^ = 19.8 kcal mol^−1^), leading to mono‐carboxylation species **F**. Next, the sequential ring‐closing and ring‐opening events through **TS6**, **G**, and **TS7** could give a slightly more stable species **H**. The isomerization via EtZn‐transferred to adjacent oxygen forms the intermediate **I** with a newly formed C═C moiety. The subsequent CO_2_ insertion could take place via six‐membered ring **TS9**, similar to **TS5**, giving the dicarboxylation species **J**. It is worth noting that during this transformation, DBU plays two important roles. One is acting as a ligand to stabilize the Zn center(s) in some key intermediates and transition states and the other is serving as a Brønsted base to deprotonate the methylene (via **TS3**). Remarkably, DFT calculations reveal a new mechanism in difunctionalization of cyclopropanols, which involves ring‐opening/*α*‐functionalization/ring‐closing/ring‐opening/*β*‐functionalization (ROFCOF) process.

## 3 Conclusion

In summary, the Et_2_Zn‐mediated germinal dicarboxylation of cyclopropanols with CO_2_ has been reported for the first time, providing a novel, straightforward and efficient strategy for the synthesis of a variety of malonic acid derivatives. This reaction features mild reaction conditions, excellent functional group compatibility, broad substrate scope, and easy derivatization of the products. DFT calculations revealed that the transformation might proceed through an unprecedented ring‐opening/ functionalization/ring‐closing/ring‐opening/ functionalization (ROFCOF) process, in which DBU plays two crucial roles. This work represents a new reaction mode for the currently widely studied cyclopropanols and opens a new window for broadening the utility of this type of substrate. Further investigations on application of this protocol are ongoing in our laboratories.

## Conflict of Interest

The authors declare no conflict of interest.

## Supporting information

Supporting Information

## Data Availability

The data that support the findings of this study are available in the supplementary material of this article.
