# Supplementary material for: Et2Zn‐Mediated Gem‐Dicarboxylation of Cyclopropanols with CO2
Source: Adv Sci (Weinh). 2023 Dec 21;11(9):2307633. doi: 10.1002/advs.202307633 (PMC10916615; doi:10.1002/advs.202307633)

ADVANCED SCIENCE
Open Access

Supporting Information

for *Adv. Sci.*, DOI 10.1002/advs.202307633

Et_2Zn-Mediated Gem-Dicarboxylation of Cyclopropanols with CO_2

Hongjian Liu, Lei Shi, Xiaobin Tan, Bangxiong Kang, Gen Luo, Huanfeng Jiang and Chaorong Qi**

Supporting Information

Et_2Zn-Mediated *Gem*-Dicarboxylation of Cyclopropanols with CO_2

Hongjian Liu,[a†] Lei Shi,[b†] Xiaobin Tan,[a] Bangxiong Kang,[a] Gen Luo,*[b] Huanfeng Jiang,[a] Chaorong Qi*[a]

[a] Key Laboratory of Functional Molecular Engineering of Guangdong Province, School of Chemistry and Chemical Engineering, South China University of Technology, Guangzhou 510640, China

[b] Institutes of Physical Science and Information Technology, Anhui University, Hefei 230601, China

E-mail: luogen@ahu.edu.cn or crqi@scut.edu.cn

List of Contents

A. General methods S1

B. Procedure for the preparation of cyclopropanols S1

C. Optimization of the reaction conditions S6

D. Procedure for the synthesis of malonic acid derivatives **2-31** and **42-49** S8

E. Procedure for the synthesis of malonic acid derivatives **32-41** S21

F. Procedure for the synthesis of compounds **50-56** S25

G. X-ray crystal structure and data for compound **37** S29

H. Computational Details S30

I. References S94

J. Copies of NMR Spectroscopies S96

A. General methods

^{1}H, ^{13}C and ^{19}F NMR spectra were recorded using a 400 MHz NMR spectrometer using $CDCl_3$ or DMSO (d_6) as solvent and TMS as an internal standard. Multiplicity was indicated as follows: s (singlet), d (doublet), t (triplet), q (quartet), m (multiplet). Mass spectra were recorded on a gas chromatograph-mass spectrometer at an ionization voltage of 70 eV and equipped with a DB-WAX capillary column (internal diameter: 0.25 mm, length: 30 m). The data of HRMS was carried out on a high-resolution mass spectrometer (LCMS-IT-TOF). IR spectra were obtained either as potassium bromide plates or as liquid films between two potassium bromide plates with an infrared spectrometer. Melting points were determined with a digital melting point measuring instrument. All the reaction temperatures reported are oil bath temperatures. The cyclopropanols **1a-1av** were prepared according to the literature procedure.[1-3] Other reagents were commercially purchased and used without further purification.

B. Procedure for the preparation of cyclopropanols

i) Procedure for the preparation of cyclopropanols **1a-1ad** (ref.[1])

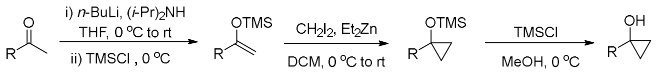

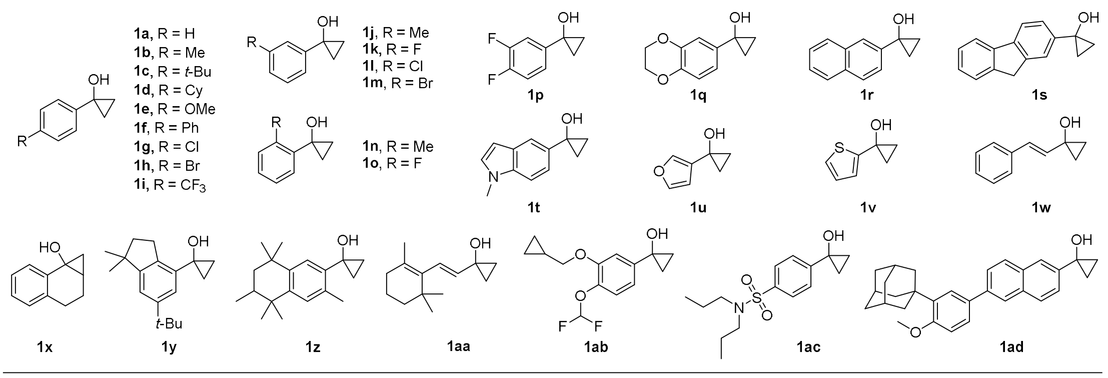

To a 100 mL dried round bottom flask equipped with a stir bar were added (*i*-Pr)$_2$NH (1.11 g, 11 mmol, 1.1 equiv) and dry THF (20 mL). The vessel was then evacuated, refilled with nitrogen gas three times. Subsequently, 4.5 mL of *n*-BuLi (2.5 M in hexane, 11 mmol, 1.1 equiv) was added dropwise to the solution via syringe at 0 °C. The resulting mixture was warmed to room temperature and stirred for an hour, followed by addition of ketone (10 mmol, 1.0 equiv) and TMSCl (1.4 mL, 11 mmol, 1.1equiv) to the mixture at 0 °C by syringe successively. After stirring at room temperature for 2 hours, the reaction mixture was quenched with $NaHCO_3$ saturated solution. The mixture was then extracted with ethyl acetate (30 mL × 3), washed with sodium chloride solution (20 mL × 3),

dried over anhydrous Na_2SO_4 and then filtered. After the solvent was removed by evaporation under a vacuum, crude enol ether product was obtained and used in the next step without further purification.

To a 100 mL dried round bottom flask equipped with a stir bar were added the enol ether above, CH_2I_2 (1.2 mL, 15 mmol, 1.5 equiv) and dry CH_2Cl_2 (20 mL) successively. The vessel was then evacuated, refilled with nitrogen gas three times. Then, 15 mL of Et_2Zn (1 M in toulene, 15 mmol, 1.5 equiv) was added to the mixture via syringe at 0 °C. The resulting mixture was warmed to room temperature and stirred overnight. The reaction mixture was quenched with NH_4Cl saturated solution, and the precipitated solid was removed by filtration. The filtrate was extracted with CH_2Cl_2 (30 mL × 3), washed with sodium chloride solution, dried over anhydrous Na_2SO_4 and then filtered. After the solvent was removed by evaporation under a vacuum, crude TMS ether product was obtained and used in the next step without further purification.

To a 100 mL dried round bottom flask equipped with a stir bar was added the TMS ether above and CH_3OH (20 mL). The vessel was then evacuated and refilled with nitrogen gas three times. A single drop of TMSCl was added to the solution by a syringe at 0 °C. The reaction was monitored using thin-layer chromatography (TLC). After the reaction was completed, the solvents were removed in vacuo, and the residue was purified by column chromatography on silica gel using petroleum ether/ethyl acetate (v/v = 5:1-10:1) as the eluent to give the desired product.

ii) Procedure for the preparation of cyclopropanols **1ae-1ak** (ref.[1])

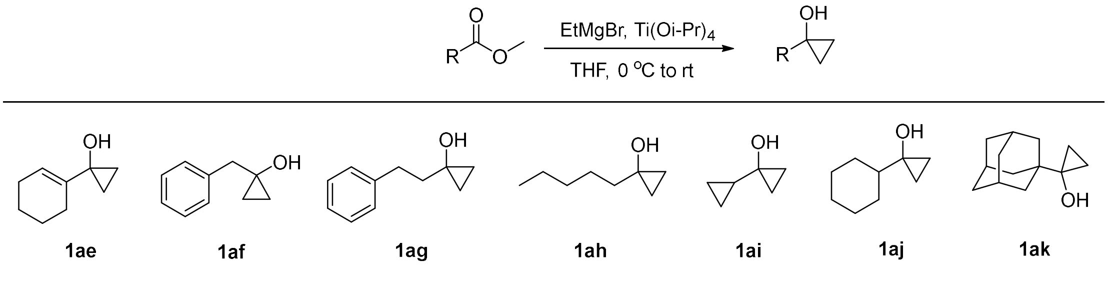

To a 100 mL dried round bottom flask equipped with a stir bar was added ester (10 mmol, 1.0 equiv), titanium isopropoxide (4.5 mL, 15 mmol, 1.5 equiv) and dry THF (20 mL). The vessel was then evacuated, refilled with nitrogen gas three times. Then, 15 mL of EtMgBr (1 M in THF, 28 mmol, 2.8 equiv) was added dropwise to the solution via syringe at 0 °C over a period of 30 mins. The resulting mixture was warmed to room temperature and stirred overnight. The reaction mixture

was quenched with NH_4Cl saturated solution, and the precipitated solid was removed by filtration. The filtrate was extracted with ethyl acetate (30 mL × 3), washed with sodium chloride solution, dried over anhydrous Na_2SO_4 and then filtered. The solvents were removed in vacuo, and the residue was purified by column chromatography on silica gel using petroleum ether/ethyl acetate (v/v = 5:1-10:1) as the eluent to give the desired product.

iii) Procedure for the preparation of cyclopropanols **1al-1au** (ref.[2])

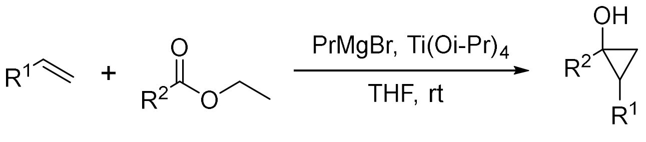

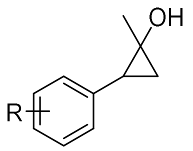

1al, R = H
1am, R = 4-Me
1an, R = 4-*t*-Bu
1ao, R = 4-OMe
1ap, R = 4-Br
1aq, R = 3-Me
1ar, R = 2-F

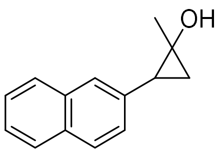

1as

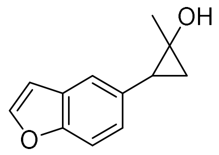

1at

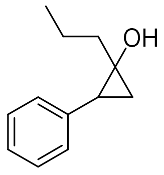

1au

To a 100 mL dried round bottom flask equipped with a stir bar were added alkene (10 mmol, 1.0 equiv), titanium isopropoxide (4.2 mL, 14 mmol, 1.0 equiv) and dry THF (20 mL). The vessel was then evacuated, refilled with nitrogen gas three times. Then, 20 mL of PrMgBr (2 M in THF, 40 mmol, 4 equiv) was added dropwise to the solution over a period of 30 mins at room temperature, and the resulting mixture stirred for 30 mins. The reaction mixture was quenched with NH_4Cl saturated solution, and the precipitated solid was removed by filtration. The filtrate was extracted with ethyl acetate (30 mL × 3), washed with sodium chloride solution, dried over anhydrous Na_2SO_4 and then filtered. The solvents were removed in vacuo, and the residue was purified by column chromatography on silica gel using petroleum ether/ethyl acetate (v/v = 2:1-10:1) as the eluent to give the desired products.

iv) Procedure for the preparation of cyclopropanols **1av** (ref.[3])

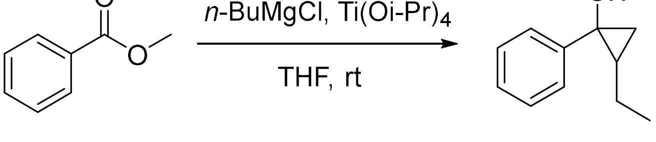

1av

To a 100 mL dried round bottom flask equipped with a stir bar was added methyl benzoate (10 mmol, 1.0 equiv), titanium isopropoxide (4.2 mL, 14 mmol, 1.4 equiv) and dry THF (20 mL). The

vessel was then evacuated, refilled with nitrogen gas three times. Then, 15 mL of *n*-BuMgCl (1 M in THF, 28 mmol, 2.8 equiv) was added dropwise to the solution over a period of 30 mins at room temperature. The resulting mixture was stirred overnight. The reaction mixture was quenched with water, and the precipitated solid was removed by filtration. The filtrate was extracted with ethyl acetate (30 mL × 3), washed with sodium chloride solution, dried over anhydrous Na_2SO_4 and then filtered. The solvents were removed in vacuo, and the residue was purified by column chromatography on silica gel using petroleum ether/ethyl acetate (v/v = 10:1) as the eluent to give the desired product.

The analytic data of the new substrates are given as follows:

1-(4-Cyclohexylphenyl)cyclopropan-1-ol (1d)

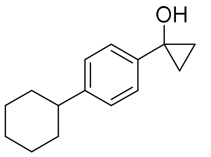

White solid (1.63 g, 76%), mp: 65 – 67 °C. ^{1}H NMR (400 MHz, $CDCl_3$): δ = 7.32 – 7.28 (m, 2H), 7.27 – 7.22 (m, 2H), 3.15 (s, 1H), 2.78 – 2.49 (m, 1H), 2.08 – 1.91 (m, 4H), 1.89 – 1.83 (m, 1H), 1.59 – 1.44 (m, 4H), 1.43 – 1.31 (m, 1H), 1.31 – 1.23 (m, 2H), 1.15 – 0.98 (m, 2H). ^{13}C{^{1}H} NMR (100 MHz, $CDCl_3$): δ = 146.1, 141.5, 126.6, 124.5, 56.3, 44.1, 34.4, 26.8, 26.1, 17.2. IR (KBr): 3346, 2920, 2852, 1434, 1234, 821, 636 cm^{-1}. HRMS-ESI (*m/z*): calcd for $C_{15}H_{19}O$ [M - H]$^-$: 215.1441; found: 215.1436.

1-(9*H*-fluoren-2-yl)cyclopropan-1-ol (1s)

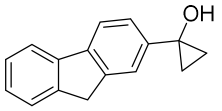

White solid (1.74 g, 78%), mp: 154 – 156 °C. ^{1}H NMR (400 MHz, $CDCl_3$): δ = 7.75 (dd, *J* = 16.4, 7.6 Hz, 2H), 7.58 – 7.50 (m, 2H), 7.38 (t, *J* = 7.2 Hz, 1H), 7.34 – 7.28 (m, 2H), 3.87 (s, 2H), 2.22 (s, 1H), 1.29 (t, *J* = 6.0 Hz, 2H), 1.11 (t, *J* = 5.6 Hz, 2H). ^{13}C{^{1}H} NMR (100 MHz, $CDCl_3$): δ = 143.5, 143.3, 142.9, 141.4, 140.2, 126.7, 126.5, 125.0, 123.3, 121.4, 119.7, 119.6, 57.0, 36.8, 17.8. IR (KBr): 3352, 2933, 1726, 1417, 1240, 1051, 833, 744 cm^{-1}. HRMS-ESI (*m/z*): calcd for $C_{11}H_{11}O_5$ [M + H]$^+$: 223.1117; found: 223.1116.

1-(1-Methyl-1*H*-indol-5-yl)cyclopropan-1-ol (1t)

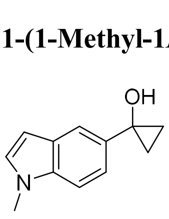

Yellow solid (1.18 g, 64%), mp: 59 – 61 °C. ^{1}H NMR (400 MHz, $CDCl_3$): δ = 7.66 (dd, *J* = 1.6, 0.8 Hz, 1H), 7.30 – 7.25 (m, 2H), 7.06 (d, *J* = 3.2 Hz, 1H), 6.47 (dd, *J* = 2.8, 0.8 Hz, 1H), 3.79 (s, 3H), 2.32 (s, 1H), 1.33 – 1.14 (m, 2H), 1.12 – 1.00 (m, 2H). ^{13}C{^{1}H} NMR (100 MHz, $CDCl_3$): δ = 134.7, 129.3, 128.4, 120.0, 117.9, 109.2, 101.0, 57.8, 32.9, 16.3. IR (KBr): 3333, 2932, 1688, 1496, 1445, 1342, 1233, 1082, 870, 796, 722 cm^{-1}. HRMS-ESI (*m/z*): calcd for $C_{12}H_{14}NO$ [M + H]$^+$: 188.1070; found: 188.1063.

1-(6-(*tert*-butyl)-1,1-dimethyl-2,3-dihydro-1*H*-inden-4-yl)cyclopropan-1-ol (1y)

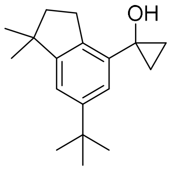

White solid (1.46 g, 56%), mp: 154 – 156 °C. ^{1}H NMR (400 MHz, $CDCl_3$): δ = 7.24 (t, J = 1.2 Hz, 1H), 7.15 (t, J = 1.6 Hz, 1H), 3.07 (td, J = 7.2, 1.2 Hz, 2H), 2.04 (s, 1H), 1.99 (td, J = 7.2, 1.2 Hz, 2H), 1.35 (s, 9H), 1.29 (s, 6H), 1.16 – 1.11 (m, 2H), 0.99 – 0.94 (m, 2H). ^{13}C{^{1}H} NMR (100 MHz, $CDCl_3$): δ = 153.1, 149.9, 140.0, 136.7, 122.9, 118.6, 57.5, 43.9, 41.5, 34.7, 31.6, 28.7, 28.2, 13.5. IR (KBr): 3346, 1703, 1476, 1371, 923, 759 cm^{-1}. HRMS-ESI (*m/z*): calcd for $C_{18}H_{25}O$ [M - H]$^-$: 257.1911; found: 257.1908.

1-(3,5,5,6,8,8-hexamethyl-5,6,7,8-tetrahydronaphthalen-2-yl)cyclopropan-1-ol (1z)

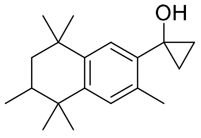

White solid (1.62 g, 59%), mp: 154 – 156 °C. ^{1}H NMR (400 MHz, $CDCl_3$): δ = 7.35 (s, 2H), 2.59 (s, 3H), 2.15 (s, 1H), 1.99 – 1.90 (m, 1H), 1.72 (t, J = 13.2 Hz, 1H), 1.44 (dd, J = 13.6, 2.4 Hz, 1H), 1.40 (s, 3H), 1.37 (s, 3H), 1.33 (s, 3H), 1.23 – 1.19 (m, 2H), 1.15 (s, 3H), 1.07 (d, J = 6.8 Hz, 3H), 1.03 – 0.98 (m, 2H). ^{13}C{^{1}H} NMR (100 MHz, $CDCl_3$): δ = 145.8, 142.0, 137.0, 135.6, 129.1, 126.7, 57.4, 43.8, 37.5, 34.6, 34.1, 32.4, 32.1, 28.6, 24.9, 18.8, 16.8, 13.7, 13.6. IR (KBr): 3329, 2949, 1659, 1455, 1377, 902, 750, 633 cm^{-1}. HRMS-ESI (*m/z*): calcd for $C_{19}H_{27}O$ [M - H]$^-$: 271.2067; found: 271.2066.

1-(3-(Cyclopropylmethoxy)-4-(difluoromethoxy)phenyl)cyclopropan-1-ol (1ab)

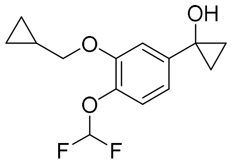

White solid (1.41 g, 52%), mp: 154 – 156 °C. ^{1}H NMR (400 MHz, $CDCl_3$): δ = 7.10 (t, J = 8.8 Hz, 1H), 6.99 (dd, J = 20.0, 2.0 Hz, 1H), 6.81 (ddd, J = 44.8 Hz, 8.4 Hz, 2.0 Hz, 1H), 6.81 (td, J = 75.6 Hz, 4.4 Hz, 1H), 3.87 (d, J = 7.2 Hz, 2H), 2.11 (s, 1H), 1.91 – 1.62 (m, 1H), 1.36 – 1.18 (m, 2H), 1.07 – 0.96 (m, 1H), 0.91 (t, J = 7.6 Hz, 1H), 0.72 – 0.57 (m, 2H), 0.40 – 0.26 (m, 2H). ^{13}C{^{1}H} NMR (100 MHz, $CDCl_3$): δ = 150.4, 143.3, 143.1, 138.9 (t, J = 3.1 Hz), 122.6, 122.4, 118.5, 116.7 (t, J = 257.8 Hz), 116.3, 111.9, 111.5, 75.5, 73.9, 73.9, 56.5, 32.0, 17.9, 10.2, 10.1, 10.0, 3.1. IR (KBr): 3373, 2935, 1682, 1599, 1511, 1404, 1265, 1125, 1027, 833, 649 cm^{-1}. HRMS-ESI (*m/z*): calcd for $C_{11}H_{11}O_5$ [M + H]$^+$: 223.0601; found: 223.0599.

1-(6-(3-((3*r*,5*r*,7*r*)-Adamantan-1-yl)-4-methoxyphenyl)naphthalen-2-yl)cyclopropan-1-ol (1ad)

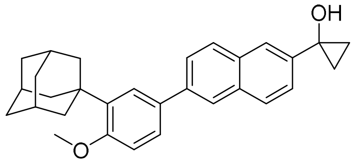

White solid (1.84 g, 43%), mp: 154 – 156 °C. ^{1}H NMR (400 MHz, $CDCl_3$): δ = 7.98 (s, 1H), 7.85 (d, J = 8.4 Hz, 3H), 7.75 (dd, J = 8.4, 2.0 Hz, 1H), 7.64 (d, J = 2.4 Hz, 1H), 7.54 (dd, J = 8.4, 2.4 Hz, 1H), 7.00 (d, J = 8.4 Hz, 1H), 3.91 (s, 3H), 2.47 (s, 1H), 2.24 (d, J = 3.2 Hz, 6H), 2.15 (t, J = 3.2 Hz,

3H), 1.85 (s, 6H), 1.38 – 1.33 (m, 2H), 1.20 – 1.16 (m, 2H). $^{13}C\{^1H\}$ NMR (100 MHz, $CDCl_3$): δ = 158.5, 138.8, 133.1, 132.0, 128.3, 128.1, 125.9, 125.8, 125.5, 124.6, 123.2, 122.7, 112.1, 56.8, 55.1, 40.6, 37.1, 37.1, 29.1, 17.7. IR (KBr): 3412, 2907, 1606, 1464, 1029, 813, 738 cm^{-1}. HRMS-ESI (*m/z*): calcd for $C_{30}H_{33}O_2$ $[M + H]^+$: 425.2475; found: 425.2470.

2-(Benzofuran-5-yl)-1-methylcyclopropan-1-ol (1at)

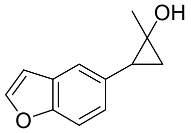

White solid (1.21 g, 64%), mp: 154 – 156 °C. ^1H NMR (400 MHz, $CDCl_3$): δ = 7.60 (d, J = 2.4 Hz, 1H), 7.42 (d, J = 8.4 Hz, 1H), 7.36 (s, 1H), 7.14 (dd, J = 8.4, 2.0 Hz, 1H), 6.71 (d, J = 2.4 Hz, 1H), 2.64 (s, 1H), 2.47 (dd, J = 10.0, 6.8 Hz, 1H), 1.30 (dd, J = 10.4, 6.0 Hz, 1H), 1.20 (s, 3H), 1.01 (t, J = 6.4 Hz, 1H). $^{13}C\{^1H\}$ NMR (100 MHz, $CDCl_3$): δ = 153.6, 145.1, 133.0, 127.4, 125.3, 120.4, 110.8, 106.4, 57.3, 30.4, 20.8, 19.1. IR (KBr): 2956, 1738, 1446, 1284, 1157, 1029, 747 cm^{-1}. HRMS-ESI (*m/z*): calcd for $C_{12}H_{13}O_2$ $[M + H]^+$: 189.0910; found: 189.0907.

C. Optimization of the reaction conditions

Table S1. The effect of base on the reaction[a]

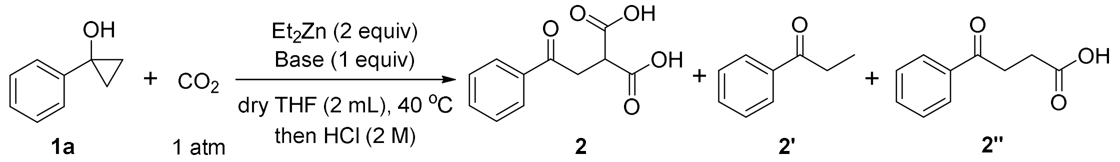

Entry[a]	Base	Yield of **2** (%)[b]	Yield of **2′** (%)[b]	Yield of **2″** (%)[b]
1	bpy	37	24	n.d.
2	DABCO	33	16	8
3	TMEDA	52	37	n.d.
**4**	**DBU**	**81**	**10**	**n.d.**
6	DBN	53	19	n.d.
6	TMG	64	35	7
7	Et_3N	26	15	7
8	K_2CO_3	trace	46	trace

[a] Reaction conditions：**1a** (0.3 mmol), Et_2Zn (2.0 equiv), CO_2 (1 atm), base (1.0 equiv), dry THF (2 mL), 40 °C, 12 h; then acidification with HCl (2 M). [b] Yields were determined by ^1H-NMR with CH_2Br_2 as internal standard.

Table S2. The effect of the mount of DBU on the reaction[a]

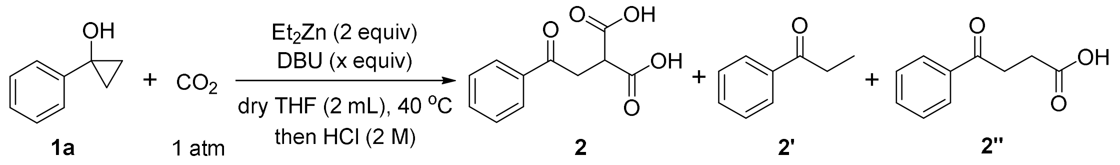

Entry[a]	DBU (x equiv)	Yield of **2** (%)[b]	Yield of **2′** (%)[b]	Yield of **2″** (%)[b]
1	1	81	10	n.d.
2	1.2	82	trace	n.d.
3	1.4	89	trace	n.d.
4	1.5	95	trace	n.d.
5	2	96	trace	n.d.
**6**[c]	**1.5**	**99 (96)**[d]	trace	n.d.

[a]Reaction conditions：**1a** (0.3 mmol), Et_2Zn (2.0 equiv), DBU (x equiv), CO_2 (1 atm), dry THF (2 mL), 40 °C, 12 h; then acidification with HCl (2 M). [b]Yields were determined by ^{1}H-NMR with CH_2Br_2 as internal standard. [c]14 h. [d]Isolated yields.

Table S3. The effect of solvent on the reaction[a]

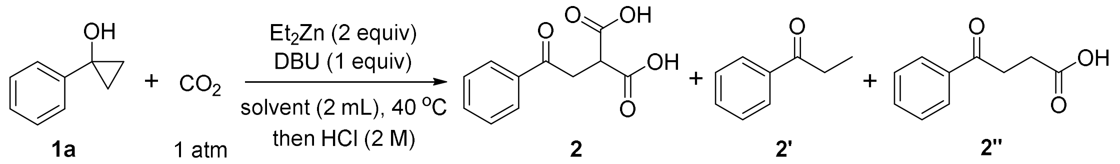

Entry[a]	Sol. (2 mL)	Yield of **2** (%)[b]	Yield of **2′** (%)[b]	Yield of **2″** (%)[b]
**1**	**THF**	**81**	**10**	**n.d.**
2	1,2-Dimethoxyethane	69	9	n.d.
3	MeCN	56	6	17
4	*t*-Butyl methyl ether	45	19	n.d.
5	DMSO	66	trace	n.d.
6	DMF	28	trace	n.d.
7	DCM	52	8	n.d.
8	Touene	54	12	n.d.
9	1,4-Dioxane	77	11	trace
10	EA	62	9	n.d.

[a]Reaction conditions：**1a** (0.3 mmol), Et_2Zn (2.0 equiv), CO_2 (1 atm), DBU (1.0 equiv), solvent (2 mL), 40 °C, 12 h; then acidification with HCl (2 M). [b]Yields were determined by ^{1}H-NMR with CH_2Br_2 as internal standard.

Table S4. The effect of different [Zn] source on the reaction[a]

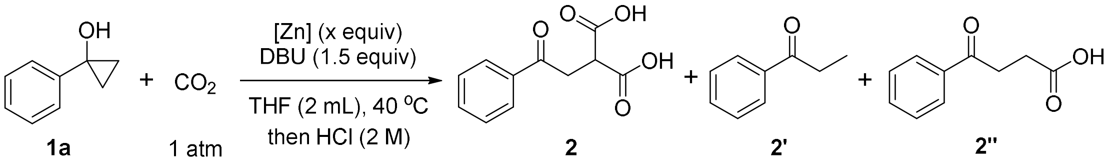

Entry[a]	[Zn] (x equiv)	Yield of **2** (%)[b]	Yield of **2′** (%)[b]	Yield of **2″** (%)[b]
**1**	**Et_2Zn (2.0)**	**99 (96)[c]**	**trace**	**n.d.**
2	Et_2Zn (1.5)	64	8	7
3	Et_2Zn (1.0)	15	58	7
4	Et_2Zn (20 mol%)	trace	trace	n.d.
5	Me_2Zn (2)	n.d.	trace	n.d.
6	$Zn(CN)_2$ (2)	n.d.	trace	n.d.

[a]Reaction conditions：**1a** (0.3 mmol), Et_2Zn (y equiv), CO_2 (1 atm), DBU (1.5 equiv), dry THF (2 mL), 40 °C, 14 h; then acidification with HCl (2 M). [b]Yields were determined by ^{1}H-NMR with CH_2Br_2 as internal standard. [c]Isolated yields.

D. Procedure for the synthesis of malonic acid derivatives 2-31 and 42-49

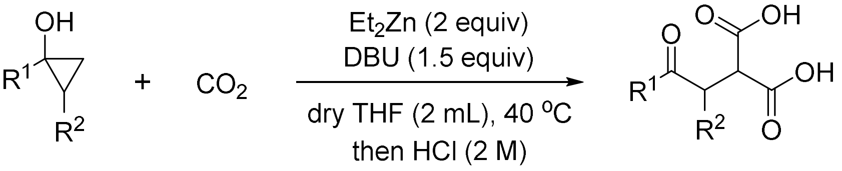

To a 25 mL oven-dried Schlenk tube equipped with a magnetic stirring bar was added cyclopropanols **1** (0.3 mmol). The tube was then evacuated, refilled with CO_2 (1 atm) three times, and charged with dry THF (2 mL), 1,8-diazabicyclo[5.4.0]undec-7-ene (DBU, 67 μL, 0.45 mmol, 1.5 equiv) and Et_2Zn (0.6 mL, 1 M in toluene, 2 equiv) successively via a syringe. Subsequently, the reaction mixture was stirred at 40 °C for 14 h. After the reaction was completed, the reaction mixture was quenched with NH_4Cl saturated solution (5 mL), acidified to pH = 1 with HCl (2 M) and extracted with ethyl acetate (10 mL × 3). The combined organic layers were dried over anhydrous Na_2SO_4 and then filtered. After removing the solvent under vacuum, the residue was purified by column chromatography on silica gel using dichloromethane/methanol (v/v = 10:1 – 20:1) as the eluent to give the desired products.

2-(2-Oxo-2-phenylethyl)malonic acid (2)

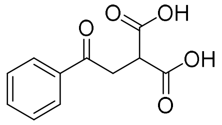

1a (0.3 mmol, 40.2 mg) was used as starting material and **2** was obtained after purification by column chromatography on silica gel using CH_2Cl_2/CH_3OH (v/v = 20:1) as the eluent. White solid (64.1 mg, 96%), mp: 154 – 156 °C. ^1H NMR (400 MHz, DMSO(d_6)): δ = 12.77 (s, 2H), 8.38 – 7.87 (m, 2H), 7.70 – 7.60 (m, 1H), 7.57 – 7.46 (m, 2H), 3.81 (t, J = 6.8 Hz, 1H), 3.54 (d, J = 7.2 Hz, 2H). $^{13}C\{^1H\}$ NMR (100 MHz, DMSO(d_6)): δ = 197.7, 171.1, 136.6, 134.1, 129.4, 128.6, 47.9, 38.2. IR (KBr): 2924, 1735, 1685, 1244, 1171, 983, 759 cm^{-1}. HRMS-ESI (m/z): calcd for $C_{11}H_{11}O_5$ $[M + H]^+$: 223.0601; found: 223.0599.

2-(2-Oxo-2-(p-tolyl)ethyl)malonic acid (3)

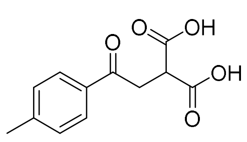

1b (0.3 mmol, 44.4 mg) was used as starting material and **3** was obtained after purification by column chromatography on silica gel using CH_2Cl_2/CH_3OH (v/v = 20:1) as the eluent. White solid (64.5 mg, 91%), mp: 151 – 152 °C. ^1H NMR (400 MHz, DMSO(d_6)): δ = 12.83 (s, 2H), 7.89 (d, J = 8.0 Hz, 2H), 7.33 (d, J = 8.0 Hz, 2H), 3.78 (t, J = 6.8 Hz, 1H), 3.50 (d, J = 7.2 Hz, 2H), 2.37 (s, 3H). $^{13}C\{^1H\}$ NMR (100 MHz, DMSO(d_6)): δ = 197.1, 171.0, 144.5, 134.1, 129.9, 128.7, 47.8, 38.0, 21.8. IR (KBr): 2927, 1736, 1680, 1241, 1177, 986, 810 cm^{-1}. HRMS-ESI (m/z): calcd for $C_{12}H_{13}O_5$ $[M + H]^+$: 237.0757; found: 237.0754.

2-(2-(4-(*tert*-Butyl)phenyl)-2-oxoethyl)malonic acid (4)

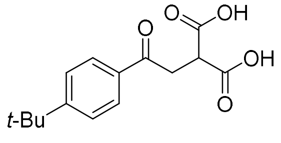

1c (0.3 mmol, 57.0 mg) was used as starting material and **4** was obtained after purification by column chromatography on silica gel using CH_2Cl_2/CH_3OH (v/v = 20:1) as the eluent. White solid (77.6 mg, 93%), mp: 168 – 170 °C. ^1H NMR (400 MHz, DMSO(d_6)): δ = 12.83 (s, 2H), 7.92 (d, J = 8.4 Hz, 2H), 7.54 (d, J = 8.4 Hz, 2H), 3.79 (t, J = 7.2 Hz, 1H), 3.50 (d, J = 7.2 Hz, 2H), 1.29 (s, 9H). $^{13}C\{^1H\}$ NMR (100 MHz, DMSO(d_6)): δ = 197.1, 171.0, 157.1, 134.1, 128.5, 126.1, 47.8, 38.0, 35.4, 31.4. IR (KBr): 3448, 2961, 1668, 1405, 1244, 825 cm^{-1}. HRMS-ESI (m/z): calcd for $C_{15}H_{19}O_5$ $[M + H]^+$: 279.1227; found: 279.1225.

2-(2-(4-Cyclohexylphenyl)-2-oxoethyl)malonic acid (5)

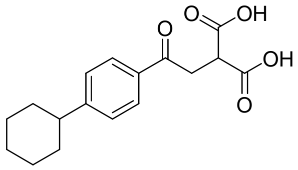

1d (0.3 mmol, 64.9 mg) was used as starting material and **5** was obtained after purification by column chromatography on silica gel using CH_2Cl_2/CH_3OH (v/v = 20:1) as the eluent. White solid (87.6 mg, 96%), mp: 179 – 180 °C. ^1H NMR (400 MHz, DMSO(d_6)): δ = 12.50 (s, 2H), 8.18 – 7.78 (m, 2H), 7.54 – 7.10 (m, 2H), 3.79 (t, J = 7.2 Hz, 1H), 3.50 (d, J = 7.2 Hz, 2H), 2.60 – 2.52 (m, 1H), 1.81 – 1.72 (m, 4H), 1.71 – 1.64 (m, 1H), 1.48 – 1.29 (m, 4H), 1.27 – 1.15 (m, 1H). $^{13}C\{^1H\}$ NMR (100 MHz, DMSO(d_6)): δ = 197.1, 171.0, 154.1, 134.5, 128.8, 127.7, 47.8, 44.4, 38.1, 34.1, 26.8, 26.1. IR (KBr): 2927, 2714, 1414, 1241, 928, 819 cm^{-1}. HRMS-ESI (*m/z*): calcd for $C_{17}H_{21}O_5$ $[M + H]^+$: 305.1384; found: 305.1379.

2-(2-(4-Methoxyphenyl)-2-oxoethyl)malonic acid (6)

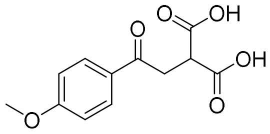

1e (0.3 mmol, 49.2 mg) was used as starting material and **6** was obtained after purification by column chromatography on silica gel using CH_2Cl_2/ CH_3OH (v/v = 20:1) as the eluent. White solid (68.1 mg, 90%), mp: 177 – 179 °C. ^1H NMR (400 MHz, DMSO(d_6)): δ = 12.72 (s, 2H), 8.24 – 7.67 (m, 2H), 7.32 – 6.68 (m, 2H), 3.82 (s, 3H), 3.78 (t, J = 7.2 Hz, 1H), 3.47 (d, J = 7.2 Hz, 2H). $^{13}C\{^1H\}$NMR (100 MHz, DMSO(d_6)): δ = 196.0, 171.2, 164.0, 130.9, 129.6, 114.6, 56.2, 47.9, 37.9. IR (KBr): 2954, 1730, 1674, 1454, 1248, 860 cm^{-1}. HRMS-ESI (*m/z*): calcd for $C_{12}H_{13}O_6$ $[M + H]^+$: 253.0707; found: 253.0704.

2-(2-([1,1'-Biphenyl]-4-yl)-2-oxoethyl)malonic acid (7)

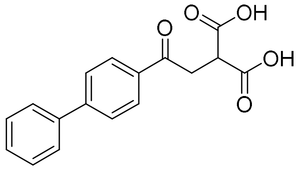

1f (0.3 mmol, 63.0 mg) was used as starting material and **7** was obtained after purification by column chromatography on silica gel using CH_2Cl_2/CH_3OH (v/v = 20:1) as the eluent. White solid (66.2 mg, 74%), mp: 191 – 193 °C. ^1H NMR (400 MHz, DMSO(d_6)): δ = 12.88 (s, 2H), 8.09 – 8.05 (m, 2H), 7.83 – 7.78 (m, 2H), 7.75 – 7.69 (m, 2H), 7.52 – 7.45 (m, 2H), 7.44 – 7.38 (m, 1H), 3.86 (t, J = 7.0 Hz, 1H), 3.59 (d, J = 7.1 Hz, 2H). $^{13}C\{^1H\}$ NMR (100 MHz, DMSO(d_6)): δ = 197.2, 171.0, 145.4, 139.4, 135.4, 129.7, 129.3, 129.0, 127.6, 127.5, 47.9, 38.3. IR

(KBr): 2920, 1709, 1538, 1409, 1283, 926, 762 cm^{-1}. HRMS-ESI (*m/z*): calcd for $C_{17}H_{15}O_5$ [M + H]$^+$: 299.0914; found: 299.0911.

2-(2-(4-Chlorophenyl)-2-oxoethyl)malonic acid (8)

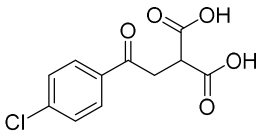

1g (0.3 mmol, 50.4 mg) was used as starting material and **8** was obtained after purification by column chromatography on silica gel using CH_2Cl_2/CH_3OH (v/v = 20:1) as the eluent. White solid (62.9 mg, 82%), mp: 178 – 180 °C. ^{1}H NMR (400 MHz, DMSO(d_6)): δ = 12.82 (s, 2H), 8.03 – 7.98 (m, 2H), 7.61 – 7.57 (m, 2H), 3.77 (t, J = 7.2 Hz, 1H), 3.52 (d, J = 6.8 Hz, 2H). ^{13}C{^{1}H} NMR (100 MHz, DMSO(d_6)): δ = 196.7, 170.8, 139.0, 135.2, 130.5, 129.4, 47.7, 38.2. IR (KBr): 2934, 1714, 1585, 1409, 1233, 1089, 993, 819 cm^{-1}. HRMS-ESI (*m/z*): calcd for $C_{11}H_{10}ClO_5$ [M + H]$^+$: 257.0211; found: 257.0207.

2-(2-(4-Bromophenyl)-2-oxoethyl)malonic acid (9)

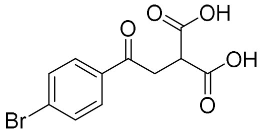

1h (0.3 mmol, 63.6 mg) was used as starting material and **9** was obtained after purification by column chromatography on silica gel using CH_2Cl_2/CH_3OH (v/v = 20:1) as the eluent. White solid (84.7 mg, 94%), mp: 163 – 165 °C. ^{1}H NMR (400 MHz, DMSO(d_6)): δ = 12.75 (s, 2H), 7.94 – 7.89 (m, 2H), 7.74 – 7.69 (m, 2H), 3.78 (t, J = 7.2 Hz, 1H), 3.52 (d, J = 7.2 Hz, 2H). ^{13}C{^{1}H} NMR (100 MHz, DMSO(d_6)): δ = 197.0, 170.9, 135.6, 132.5, 130.6, 128.2, 47.8, 38.2. IR (KBr): 2829, 1594, 1362, 1092, 775 cm^{-1}. HRMS-ESI (*m/z*): calcd for $C_{11}H_{11}BrO_5$ [M + H]$^+$: 300.9706; found: 300.9702.

2-(2-Oxo-2-(4-(trifluoromethyl)phenyl)ethyl)malonic acid (10)

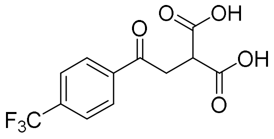

1i (0.3 mmol, 60.6 mg) was used as starting material and **10** was obtained after purification by column chromatography on silica gel using CH_2Cl_2/CH_3OH (v/v = 20:1) as the eluent. White solid (65.2 mg, 75%), mp: 170 – 171 °C. ^{1}H NMR (400 MHz, DMSO(d_6)): δ = 12.90 (s, 2H), 8.19 (d, J = 8.0 Hz, 2H), 7.89 (d, J = 8.4 Hz, 2H), 3.81 (t, J = 7.2 Hz, 1H), 3.59 (d, J = 6.8 Hz, 2H). ^{13}C{^{1}H} NMR (100 MHz, DMSO(d_6)): δ = 197.3, 170.8, 139.7, 133.4 (q, J = 31.7 Hz), 129.4, 126.3 (q, J = 3.7 Hz), 124.3 (d, J = 271.0 Hz), 47.8, 38.5. IR (KBr): 2866, 1716, 1430, 1325, 1232, 1179, 1132, 1066, 826 cm^{-1}. HRMS-ESI (*m/z*): calcd for $C_{12}H_{11}F_3O_5$ [M + H]$^+$: 291.0475; found: 291.0470.

2-(2-Oxo-2-(m-tolyl)ethyl)malonic acid (11)

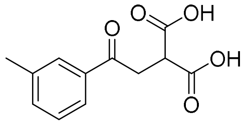

1j (0.3 mmol, 44.43 mg) was used as starting material and **11** was obtained after purification by column chromatography on silica gel using (CH_2Cl_2 / CH_3OH (v/v = 20:1) as the eluent. White solid (68.0 mg, 96%), mp: 171 – 173 °C. ^{1}H NMR (400 MHz, DMSO(d_6)): δ = 12.69 (s, 2H), 7.91 – 7.68 (m, 2H), 7.55 – 7.30 (m, 2H), 3.80 (t, J = 7.2 Hz, 1H), 3.52 (d, J = 7.2 Hz, 2H), 2.36 (s, 3H). ^{13}C{^{1}H} NMR (100 MHz, DMSO(d_6)): δ = 197.8, 171.1, 138.9, 136.7, 134.7, 129.3, 129.1, 125.8, 47.9, 38.3, 21.5. IR (KBr): 2930, 1700, 1428, 1304, 1167, 934, 788 cm^{-1}. HRMS-ESI (m/z): calcd for $C_{12}H_{13}O_5$ [M + H]$^+$: 237.0757; found: 237.0754.

2-(2-(3-Fluorophenyl)-2-oxoethyl)malonic acid (12)

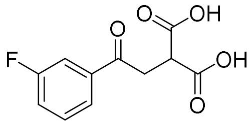

1k (0.3 mmol, 45.6 mg) was used as starting material and **12** was obtained after purification by column chromatography on silica gel using CH_2Cl_2/CH_3OH (v/v = 20:1) as the eluent. White solid (60.5 mg, 84%), mp: 163 – 165 °C. ^{1}H NMR (400 MHz, DMSO(d_6)): δ = 12.85 (s, 2H), 7.84 (d, J = 7.6 Hz, 1H), 7.77 – 7.69 (m, 1H), 7.61 – 7.51 (m, 1H), 7.51 – 7.42 (m, 1H), 3.80 (t, J = 7.2 Hz, 1H), 3.55 (d, J = 7.2 Hz, 2H). ^{13}C{^{1}H} NMR (100 MHz, DMSO(d_6)): δ = 196.9 (d, J = 2.1 Hz), 171.1, 163.0 (d, J = 244.1 Hz), 138.9 (d, J = 6.1 Hz), 131.7 (d, J = 7.8 Hz), 125.0 (d, J = 2.8 Hz), 121.1 (d, J = 21.1 Hz), 115.1 (d, J = 22.1 Hz), 48.0, 38.5. IR (KBr): 2898, 2790, 1698, 1575, 1428, 1258, 878, 673 cm^{-1}. HRMS-ESI (m/z): calcd for $C_{11}H_{10}FO_5$ [M + H]$^+$: 241.0507; found: 241.0502.

2-(2-(3-Chlorophenyl)-2-oxoethyl)malonic acid (13)

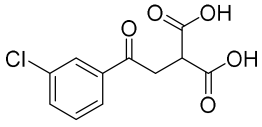

1l (0.3 mmol, 50.4 mg) was used as starting material and **13** was obtained after purification by column chromatography on silica gel using CH_2Cl_2/CH_3OH (v/v = 20:1) as the eluent. White solid (64.6 mg, 84%), mp: 176 – 177 °C. ^{1}H NMR (400 MHz, DMSO(d_6)): δ = 12.80 (s, 2H), 7.99 – 7.93 (m, 2H), 7.73 – 7.69 (m, 1H), 7.56 (t, J = 7.6 Hz, 1H), 3.78 (t, J = 6.8 Hz, 1H), 3.54 (d, J = 7.2 Hz, 2H). ^{13}C{^{1}H} NMR (100 MHz, DMSO(d_6)): δ = 196.8, 170.8, 138.3, 134.4, 133.7, 131.4, 128.1, 127.3, 47.8, 38.3. IR (KBr): 2931, 1704, 1416, 1239, 787 cm^{-1}. HRMS-ESI (m/z): calcd for $C_{11}H_{10}ClO_5$ [M + H]$^+$: 257.0211 found: 257.0207.

2-(2-(3-Bromophenyl)-2-oxoethyl)malonic acid (14)

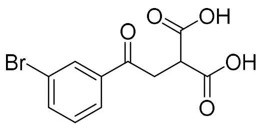

1m (0.3 mmol, 63.6 mg) was used as starting material and **14** was obtained after purification by column chromatography on silica gel using CH_2Cl_2/CH_3OH (v/v = 20:1) as the eluent. White solid (64.0 mg, 81%), mp: 170 – 172 °C. ^{1}H NMR (400 MHz, DMSO(d_6)): δ = 12.88 (s, 2H), 8.10 (t, J = 1.9 Hz, 1H), 8.00 (d, J = 8.3 Hz, 1H), 7.91 – 7.82 (m, 1H), 7.50 (td, J = 8.0, 2.4 Hz, 1H), 3.76 (t, J = 7.2 Hz, 1H), 3.54 (d, J = 6.8 Hz, 2H). ^{13}C{^{1}H} NMR (100 MHz, DMSO(d_6)): δ = 196.7, 170.8, 138.5, 136.6, 131.6, 131.0, 127.6, 122.8, 47.7, 38.2. IR (KBr): 2885, 2784, 1710, 1420, 1302, 814, 675 cm^{-1}. HRMS-ESI (*m/z*): calcd for $C_{11}H_{10}BrO_5$ $[M + H]^+$: 300.9706; found: 300.9701.

2-(2-Oxo-2-(o-tolyl)ethyl)malonic acid (15)

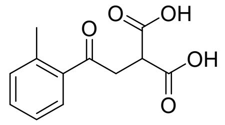

1n (0.3 mmol, 44.4 mg) was used as starting material and **15** was obtained after purification by column chromatography on silica gel using CH_2Cl_2/CH_3OH (v/v = 20:1) as the eluent. White solid (50.3 mg, 71%), mp: 122 – 124 °C. ^{1}H NMR (400 MHz, DMSO(d_6)): δ = 12.57 (s, 2H), 7.83 – 7.75 (m, 1H), 7.46 – 7.39 (m, 1H), 7.37 – 7.25 (m, 2H), 3.78 (t, J = 7.2 Hz, 1H), 3.42 (d, J = 7.2 Hz, 2H), 2.37 (s, 3H). ^{13}C{^{1}H} NMR (100 MHz, DMSO(d_6)): δ = 201.6, 171.0, 137.8, 137.6, 132.2, 132.1, 129.2, 126.5, 48.1, 40.9, 21.1. IR (KBr): 3449, 2805, 1654, 1607, 1288, 984, 758 cm^{-1}. HRMS-ESI (*m/z*): calcd for $C_{12}H_{13}O_5$ $[M + H]^+$: 237.0757; found: 237.0752.

2-(2-(2-Fluorophenyl)-2-oxoethyl)malonic acid (16)

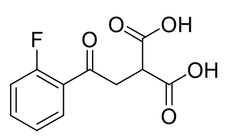

1o (0.3 mmol, 45.6 mg) was used as starting material and **16** was obtained after purification by column chromatography on silica gel using CH_2Cl_2/CH_3OH (v/v = 20:1) as the eluent. White solid (37.5 mg, 52%), mp: 176 – 178 °C. ^{1}H NMR (400 MHz, DMSO(d_6)): δ = 12.51 (s, 2H), 7.84 (td, J = 7.6, 2.0 Hz, 1H), 7.72 – 7.63 (m, 1H), 7.39 – 7.29 (m, 2H), 3.78 (t, J = 6.8 Hz, 1H), 3.46 (dd, J = 7.2, 2.8 Hz, 2H). ^{13}C{^{1}H} NMR (100 MHz, DMSO(d_6)): δ = 195.6, 170.9, 161.9 (d, J = 252.4 Hz), 136.1 (d, J = 9.2 Hz), 130.9 (d, J = 2.2 Hz), 125.5 (d, J = 3.3 Hz), 125.1 (d, J = 12.0 Hz), 117.6 (d, J = 23.1 Hz), 47.8, 42.4 (d, J = 7.5 Hz). IR (KBr): 3461, 2929, 1690, 1445, 1276, 965, 769 cm^{-1}. HRMS-ESI (*m/z*): calcd for $C_{16}H_{17}F_2O_7$ $[M + H]^+$: 239.0361; found: 239.0356.

2-(2-(3,4-Difluorophenyl)-2-oxoethyl)malonic acid (17)

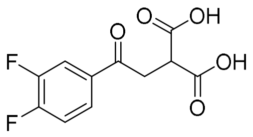

1p (0.3 mmol, 51.0 mg) was used as starting material and **17** was obtained after purification by column chromatography on silica gel using CH_2Cl_2/CH_3OH (v/v = 20:1) as the eluent. White solid (65.8 mg, 85%), mp: 141 – 143 °C. ^1H NMR (400 MHz, DMSO(d_6)): δ = 12.52 (s, 2H), 8.04 – 7.97 (m, 1H), 7.92 – 7.87 (m, 1H), 7.60 – 7.50 (m, 1H), 3.77 (t, J = 7.2 Hz, 1H), 3.53 (d, J = 7.2 Hz, 2H). $^{13}C\{^1H\}$ NMR (100 MHz, DMSO(d_6)): δ = 195.8, 170.9, 153.5 (dd, J = 252.4, 12.6 Hz), 150.2 (dd, J = 246.5, 13.0 Hz), 134.1 (t, J = 4.0 Hz), 126.5 (dd, J = 7.7, 3.4 Hz), 118.6 (d, J = 17.7 Hz), 118.0 (d, J = 17.9 Hz), 47.9, 38.3. IR (KBr): 2831, 1680, 1601, 1362, 1238, 774 cm^{-1}. HRMS-ESI (m/z): calcd for $C_{11}H_9F_2O_5$ [M + H]$^+$: 259.0413; found: 259.0409.

2-(2-(2,3-Dihydrobenzo[*b*][1,4]dioxin-6-yl)-2-oxoethyl)malonic acid (18)

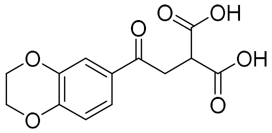

1q (0.3 mmol, 57.6 mg) was used as starting material and **18** was obtained after purification by column chromatography on silica gel using CH_2Cl_2/CH_3OH (v/v = 20:1) as the eluent. White solid (61.4 mg, 73%), mp: 185 – 187 °C. ^1H NMR (400 MHz, DMSO(d_6)): δ = 12.82 (s, 2H), 7.52 (dd, J = 8.5, 2.0 Hz, 1H), 7.44 (d, J = 2.1 Hz, 1H), 6.97 (d, J = 8.4 Hz, 1H), 4.37 – 4.24 (m, 4H), 3.74 (t, J = 7.2 Hz, 1H), 3.43 (d, J = 7.2 Hz, 2H). $^{13}C\{^1H\}$ NMR (100 MHz, DMSO(d_6)): δ = 195.8, 170.9, 148.6, 143.8, 130.2, 122.6, 117.7, 117.4, 65.1, 64.5, 47.8, 37.8. IR (KBr): 2832, 1604, 1362, 1068, 774 cm^{-1}. HRMS-ESI (m/z): calcd for $C_{13}H_{13}O_7$ [M + H]$^+$: 281.0656; found: 281.0653. **2-(2-(Naphthalen-2-yl)-2-oxoethyl)malonic acid (19)**

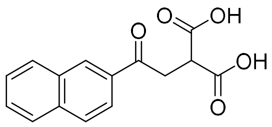

1r (0.3 mmol, 55.2 mg) was used as starting material and **19** was obtained after purification by column chromatography on silica gel using CH_2Cl_2/CH_3OH (v/v = 20:1) as the eluent. White solid (75.1 mg, 92%), mp: 174 – 176 °C. ^1H NMR (400 MHz, DMSO(d_6)): δ = 12.75 (s, 2H), 8.76 (s, 1H), 8.16 (d, J = 8.0 Hz, 1H), 8.05 – 7.94 (m, 3H), 7.74 – 7.56 (m, 2H), 3.86 (t, J = 7.2 Hz, 1H), 3.69 (d, J = 6.8 Hz, 2H). $^{13}C\{^1H\}$ NMR (100 MHz, DMSO(d_6)): δ = 197.6, 171.0, 135.7, 133.8, 132.8, 130., 130.3, 129.3, 128.9, 128.2, 127.5, 123.9, 47.9, 38.2. IR (KBr): 2833, 1603, 1362, 1067, 775 cm^{-1}. HRMS-ESI (m/z): calcd for $C_{15}H_{13}O_5$ [M + H]$^+$: 273.0757; found: 273.0752.

2-(2-(9*H*-Fluoren-2-yl)-2-oxoethyl)malonic acid (20)

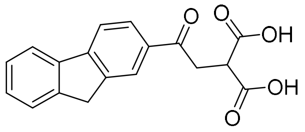

1s (0.3 mmol, 66.6 mg) was used as starting material and **20** was obtained after purification by column chromatography on silica gel using CH_2Cl_2/CH_3OH (v/v = 20:1) as the eluent. White solid (86.6 mg, 93%), mp: 186 – 188 °C. ^{1}H NMR (400 MHz, DMSO(d_6)): δ = 12.85 (s, 2H), 8.19 (s, 1H), 8.06 – 7.94 (m, 3H), 7.65 – 7.59 (m, 1H), 7.45 – 7.36 (m, 2H), 3.96 (s, 2H), 3.85 (t, J = 7.2 Hz, 1H), 3.60 (d, J = 6.8 Hz, 2H). $^{13}C\{^1H\}$ NMR (100 MHz, DMSO(d_6)): δ = 197.4, 171.3, 146.6, 145.2, 144.0, 140.6, 135.1, 128.8, 127.9, 127.7, 126.0, 125.5, 121.8, 120.7, 48.1, 38.4, 37.1. IR (KBr): 2884, 2786, 1717, 1426, 1262, 957, 823, 730 cm^{-1}. HRMS-ESI (*m/z*): calcd for $C_{18}H_{15}O_5$ [M + H]$^+$: 311.0914; found: 311.0912.

2-(2-(1-Methyl-1*H*-indol-5-yl)-2-oxoethyl)malonic acid (21)

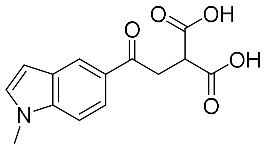

1t (0.3 mmol, 56.1 mg) was used as starting material and **21** was obtained after purification by column chromatography on silica gel using CH_2Cl_2/CH_3OH (v/v = 20:1) as the eluent. White solid (44.6 mg, 54%), mp: 165 – 167 °C. ^{1}H NMR (400 MHz, DMSO(d_6)): δ = 12.03 (s, 2H), 8.35 (d, J = 1.6 Hz, 1H), 7.81 (dd, J = 8.8, 1.6 Hz, 1H), 7.50 (d, J = 8.8 Hz, 1H), 7.42 (d, J = 3.2 Hz, 1H), 6.62 (d, J = 3.2 Hz, 1H), 3.85 (t, J = 6.8 Hz, 1H), 3.80 (s, 3H), 3.61 (d, J = 6.8 Hz, 2H). $^{13}C\{^1H\}$ NMR (100 MHz, DMSO(d_6)): δ = 197.1, 171.3, 139.5, 132.2, 128.5, 128.2, 123.1, 121.5, 110.4, 103.2, 48.1, 38.1, 33.3. IR (KBr): 3478, 2989, 1759, 1657, 1383, 1243, 1056 cm^{-1}. HRMS-ESI (*m/z*): calcd for $C_{14}H_{14}NO_5$ [M + H]$^+$: 276.0866; found: 276.0864.

2-(2-(Furan-3-yl)-2-oxoethyl)malonic acid (22)

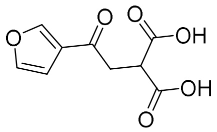

1u (0.3 mmol, 37.2 mg) was used as starting material and **22** was obtained after purification by column chromatography on silica gel using CH_2Cl_2/CH_3OH (v/v = 20:1) as the eluent. White solid (25.4 mg, 40%), mp: 176 – 177 °C. ^{1}H NMR (400 MHz, DMSO(d_6)): δ = 12.03 (s, 2H), 9.01 – 8.23 (m, 1H), 7.76 (t, J = 1.6 Hz, 1H), 6.89 – 6.72 (m, 1H), 3.75 (t, J = 7.2 Hz, 1H), 3.32 (d, J = 7.2 Hz, 2H). $^{13}C\{^1H\}$ NMR (100 MHz, DMSO(d_6)): δ = 192.6, 171.1, 150.0, 145.7, 127.3, 108.9, 47.6, 39.5. IR (KBr): 2984,

2929, 1746, 1673, 1513, 1368, 1243, 1160, 1053 cm^{-1}. HRMS-ESI (*m/z*): calcd for $C_{15}H_{14}O_5$ [M + H]$^+$: 213.0394; found: 213.0390.

2-(2-Oxo-2-(thiophen-2-yl)ethyl)malonic acid (23)

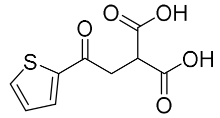

1v (0.3 mmol, 42.0 mg) was used as starting material and **23** was obtained after purification by column chromatography on silica gel using CH_2Cl_2/CH_3OH (v/v = 20:1) as the eluent. White solid (56.8 mg, 83%), mp: 169 – 171 °C. ^{1}H NMR (400 MHz, DMSO(d_6)): δ = 12.77 (s, 2H), 8.02 (dd, J = 3.6, 1.2 Hz, 1H), 7.98 (dd, J = 5.2, 1.2 Hz, 1H), 7.25 – 7.21 (m, 1H), 3.78 (t, J = 7.2 Hz, 1H), 3.48 (d, J = 7.2 Hz, 2H). ^{13}C{^{1}H} NMR (100 MHz, DMSO(d_6)): δ = 190.8, 170.9, 143.5, 135.6, 134.3, 129.5, 47.8, 38.4. IR (KBr): 2967, 1730, 1658, 1415, 1243, 849, 731 cm^{-1}. HRMS-ESI (*m/z*): calcd for $C_9H_9O_5S$ [M + H]$^+$: 229.0165; found: 229.0163.

(*E*)-2-(2-Oxo-4-phenylbut-3-en-1-yl)malonic acid (24)

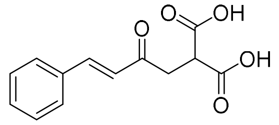

1w (0.3 mmol, 48.0 mg) was used as starting material and **24** was obtained after purification by column chromatography on silica gel using CH_2Cl_2/CH_3OH (v/v = 20:1) as the eluent. White solid (29.8 mg, 40%), mp: 161 – 163 °C. ^{1}H NMR (400 MHz, DMSO(d_6)): δ = 12.76 (s, 2H), 7.77 – 7.70 (m, 2H), 7.68 (d, J = 16.4 Hz, 1H), 7.49 – 7.39 (m, 3H), 6.91 (d, J = 16.4 Hz, 1H), 3.67 (t, J = 6.8 Hz, 1H), 3.23 (d, J = 7.2 Hz, 2H). ^{13}C{^{1}H} NMR (100 MHz, DMSO(d_6)): δ = 197.6, 171.0, 143.3, 134.9, 131.2, 129.5, 129.1, 126.5, 47.6, 39.7. IR (KBr): 3461, 1645, 1243 cm^{-1}. HRMS-ESI (*m/z*): calcd for $C_{13}H_{13}O_5$ [M + H]$^+$: 249.0757; found: 249.0756.

2-(2-(Cyclohex-1-en-1-yl)-2-oxoethyl)malonic acid (25)

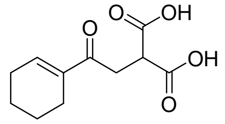

1ae (0.3 mmol, 49.2 mg) was used as starting material and **25** was obtained after purification by column chromatography on silica gel using CH_2Cl_2/CH_3OH (v/v = 20:1) as the eluent. White solid (38.0 mg, 56%), mp: 165 – 166 °C. ^{1}H NMR (400 MHz, DMSO(d_6)): δ = 12.69 (s, 2H), 7.63 – 6.51 (m, 1H), 3.61 (t, J = 7.2 Hz, 1H), 3.15 (d, J = 7.2 Hz, 2H), 2.26 – 2.18 (m, 2H), 2.13 – 2.05 (m, 2H), 1.65 – 1.48 (m, 4H). ^{13}C{^{1}H} NMR (100 MHz, DMSO(d_6)): δ = 197.8, 171.1, 141.6, 138.1, 47.7, 36.7, 26.1, 23.3, 22.1,

21.7. IR (KBr): 3456, 2934, 1729, 1655, 1403, 1240, 787 cm^{-1}. HRMS-ESI (*m/z*): calcd for $C_{11}H_{15}O_5$ [M + H]$^+$: 227.0914; found: 227.0912.

2-(2-Oxo-4-phenylbutyl)malonic acid (27)

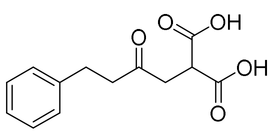

1ag (0.3 mmol, 48.6 mg) was used as starting material and **27** was obtained after purification by column chromatography on silica gel using CH_2Cl_2/CH_3OH (v/v = 20:1) as the eluent. Colorless oil (10.8 mg, 14%). ^{1}H NMR (400 MHz, DMSO(d_6)): δ = 12.77 (s, 2H), 7.29 – 7.23 (m, 2H), 7.22 – 7.14 (m, 3H), 3.56 (t, J = 7.2 Hz, 1H), 2.95 (d, J = 7.2 Hz, 2H), 2.84 – 2.70 (m, 4H). ^{13}C{^{1}H} NMR (100 MHz, DMSO(d_6)): δ = 207.5, 170.9, 141.5, 128.8, 128.7, 126.4, 47.3, 43.5, 41.3, 29.4. IR (KBr): 2932, 2833, 1712, 1598, 1361, 1070, 774 cm^{-1}. HRMS-ESI (*m/z*): calcd for $C_{13}H_{15}O_5$ [M + H]$^+$: 251.0914; found: 251.0911.

2-(2-Oxoheptyl)malonic acid (28)

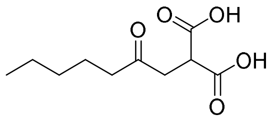

1ah (0.3 mmol, 38.4 mg) was used as starting material and **28** was obtained after purification by column chromatography on silica gel using CH_2Cl_2/CH_3OH (v/v = 20:1) as the eluent. White solid (48.0 mg, 74%), mp: 117 – 119 °C. ^{1}H NMR (400 MHz, DMSO(d_6)): δ = 11.91 (s, 2H), 3.55 (t, J = 7.2 Hz, 1H), 2.90 (d, J = 7.2 Hz, 2H), 2.43 (t, J = 7.2 Hz, 2H), 1.45 (p, J = 7.2 Hz, 2H), 1.33 – 1.14 (m, 4H), 0.84 (t, J = 6.8 Hz, 3H). ^{13}C{^{1}H} NMR (100 MHz, DMSO(d_6)): δ = 208.4, 170.9, 47.4, 42.1, 41.4, 31.2, 23.4, 22.5, 14.4. IR (KBr): 2930, 2864, 1714, 1457, 1244, 1056, 828, 750 cm^{-1}. HRMS-ESI (*m/z*): calcd for $C_{10}H_{15}O_5$ [M - H]$^-$: 215.0925; found: 215.0920.

2-(2-Cyclopropyl-2-oxoethyl)malonic acid (29)

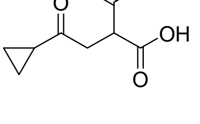

1ai (0.3 mmol, 29.4 mg) was used as starting material and **29** was obtained after purification by column chromatography on silica gel using CH_2Cl_2/CH_3OH (v/v = 20:1) as the eluent. Pale yellow oil (22.4 mg, 40%). ^{1}H NMR (400 MHz, DMSO(d_6)): δ = 12.89 (s, 2H), 3.58 (t, J = 7.2 Hz, 1H), 3.06 (d, J = 7.2 Hz, 2H), 2.14 – 2.00 (m, 1H), 0.93 – 0.86 (m, 2H), 0.86 – 0.81 (m, 2H). ^{13}C{^{1}H} NMR (100 MHz, DMSO(d_6)): δ = 207.9, 170.8, 47.4, 41.7, 20.6, 10.7. IR (KBr): 3341, 3213, 2928, 1708, 1538, 1340, 1181 cm^{-1}. HRMS-ESI (*m/z*): calcd for $C_8H_{11}O_5$ [M + H]$^+$: 187.0601; found: 187.0598.

2-(2-Cyclohexyl-2-oxoethyl)malonic acid (30)

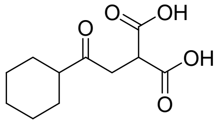

1aj (0.3 mmol, 42.0 mg) was used as starting material and **30** was obtained after purification by column chromatography on silica gel using CH_2Cl_2/CH_3OH (v/v = 20:1) as the eluent. White solid (59.6 mg, 87%). ^1H NMR (400 MHz, DMSO(d_6)): δ = 12.78 (s, 2H), 3.55 (t, J = 7.2 Hz, 1H), 2.95 (d, J = 7.2 Hz, 2H), 2.46 – 2.35 (m, 1H), 1.81 – 1.53 (m, 5H), 1.33 – 1.06 (m, 5H). $^{13}C\{^1H\}$ NMR (100 MHz, DMSO(d_6)): δ = 211.0, 170.9, 49.8, 47.4, 28.5, 26.0, 25.6. IR (KBr): 2928, 2855, 1707, 1630, 1450, 1365, 1248, 998, 774 cm^{-1}. HRMS-ESI (m/z): calcd for $C_{11}H_{15}O_5$ [M - H]$^-$: 227.0925; found: 227.0919.

2-(2-((3*r*,5*r*,7*r*)-Adamantan-1-yl)-2-oxoethyl)malonic acid (31)

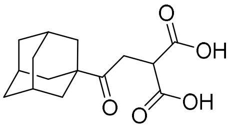

1ak (0.3 mmol, 57.7 mg) was used as starting material and **31** was obtained after purification by column chromatography on silica gel using CH_2Cl_2/CH_3OH (v/v = 20:1) as the eluent. White solid (82.5 mg, 98%), mp: 162 – 164 °C. ^1H NMR (400 MHz, DMSO(d_6)): δ = 12.76 (s, 2H), 3.54 (t, J = 7.2 Hz, 1H), 2.96 (d, J = 6.8 Hz, 2H), 2.07 – 1.89 (m, 3H), 1.75 (d, J = 3.2 Hz, 6H), 1.72 – 1.60 (m, 6H). $^{13}C\{^1H\}$ NMR (100 MHz, DMSO(d_6)): δ = 212.5, 171.0, 47.4, 45.9, 38.2, 36.6, 36.0, 27.9. IR (KBr): 2914, 1714, 1421, 1251, 1169, 664 cm^{-1}. HRMS-ESI (m/z): calcd for $C_{15}H_{19}O_5$ [M - H]$^-$: 279.1238; found: 279.1239.

2-(1-Oxo-1-phenylbutan-2-yl)malonic acid (42)

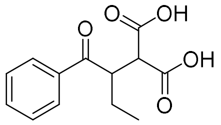

1av (0.3 mmol, 48.6 mg) was used as starting material and **42** was obtained after purification by column chromatography on silica gel using CH_2Cl_2/CH_3OH (v/v = 20:1) as the eluent. White solid (17.4 mg, 23%), mp: 159 – 161 °C. ^1H NMR (400 MHz, DMSO(d_6)): δ = 12.93 (s, 2H), 7.99 (d, J = 7.6 Hz, 2H), 7.66 (t, J = 7.2 Hz, 1H), 7.55 (t, J = 7.6 Hz, 2H), 4.15 – 4.06 (m, 1H), 3.71 (d, J = 11.2 Hz, 1H), 1.72 – 1.51 (m, 2H), 0.69 (t, J = 7.6 Hz, 3H). $^{13}C\{^1H\}$ NMR (100 MHz, DMSO(d_6)): δ = 197.8, 173.3, 137.0, 133.9, 129.4, 128.4, 55.4, 41.5, 26.9, 9.7. IR (KBr): 3456, 1648, 1243, 702 cm^{-1}. HRMS-ESI (m/z): calcd for $C_{13}H_{13}O_5$ [M - H]$^-$: 249.0768; found: 249.0766.

5-Oxo-5,6,8,9-tetrahydro-7*H*-benzo[7]annulene-7,7-dicarboxylic acid (43)

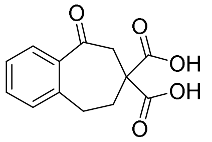

1x (0.3 mmol, 48.6 mg) was used as starting material and **43** was obtained after purification by column chromatography on silica gel using CH_2Cl_2/CH_3OH (v/v = 20:1) as the eluent. White solid (56.6 mg, 76%), mp: 137 – 138 °C. ^1H NMR (400 MHz, DMSO(d_6)): δ = 12.98 (s, 2H), 7.62 (dd, J = 7.6, 1.6 Hz, 1H), 7.47 (td, J = 7.2, 1.2 Hz, 1H), 7.31 (t, J = 7.6 Hz, 2H), 3.21 (s, 2H), 3.04 – 2.96 (m, 2H), 2.45 – 2.30 (m, 2H). $^{13}C\{^1H\}$ NMR (100 MHz, DMSO(d_6)): δ = 200.0, 172.7, 142.9, 137.8, 133.0, 130.6, 128.9, 127.1, 54.1, 46.6, 32.8, 30.6. IR (KBr): 3436, 2928, 1721, 1446, 1245, 1174, 757 cm^{-1}. HRMS-ESI (*m/z*): calcd for $C_{13}H_{13}O_5$ $[M + H]^+$: 249.0757; found: 249.0754.

2-(2-(6-(*tert*-Butyl)-1,1-dimethyl-2,3-dihydro-1*H*-inden-4-yl)-2-oxoethyl)malonic acid (44)

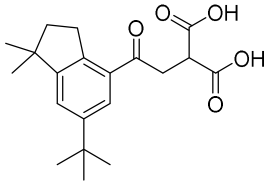

1y (0.3 mmol, 77.5 mg) was used as starting material and **44** was obtained after purification by column chromatography on silica gel using CH_2Cl_2/CH_3OH (v/v = 20:1) as the eluent. White solid (84.2 mg, 81%), mp: 187 – 189 °C. ^1H NMR (400 MHz, DMSO(d_6)): δ = 12.79 (s, 2H), 7.75 (d, J = 2.0 Hz, 1H), 7.44 (d, J = 1.6 Hz, 1H), 3.77 (t, J = 7.2 Hz, 1H), 3.49 (d, J = 7.2 Hz, 2H), 3.05 (t, J = 7.2 Hz, 2H), 1.85 (t, J = 7.2 Hz, 2H), 1.32 (s, 9H), 1.22 (s, 6H). $^{13}C\{^1H\}$ NMR (100 MHz, DMSO(d_6)): δ = 199.3, 171.0, 154.4, 150.3, 140.7, 133.1, 124.3, 123.8, 47.9, 43.5, 41.4, 39.7, 35.0, 31.8, 30.8, 29.1. IR (KBr): 2954, 1730, 1674, 1629, 1454, 1248, 1046, 860 cm^{-1}. HRMS-ESI (*m/z*): calcd for $C_{20}H_{27}O_5$ $[M + H]^+$: 347.1853; found: 347.1849.

2-(2-(3,5,5,6,8,8-Hexamethyl-5,6,7,8-tetrahydronaphthalen-2-yl)-2-oxoethyl)malonic acid (45)

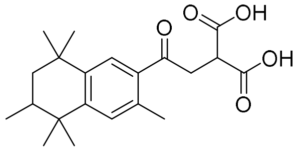

1z (0.3 mmol, 81.7 mg) was used as starting material and **45** was obtained after purification by column chromatography on silica gel using CH_2Cl_2/CH_3OH (v/v = 20:1) as the eluent. White solid (87.5 mg, 81%), mp: 168 – 170 °C. ^1H NMR (400 MHz, DMSO(d_6)): δ = 12.44 (s, 2H), 7.73 (s, 1H), 7.25 (s, 1H), 3.76 (t, J = 7.2 Hz, 1H), 3.43 (d, J = 7.2 Hz, 2H), 2.33 (s, 3H), 1.87 – 1.75 (m, 1H), 1.56 (t, J = 13.2 Hz, 1H), 1.37 (dd, J = 13.2, 2.4 Hz, 1H), 1.29 (s, 3H), 1.27 (s, 3H), 1.23 (s, 3H), 1.01 (s, 3H), 0.94 (d, J = 6.4 Hz, 3H). $^{13}C\{^1H\}$ NMR (100 MHz, DMSO(d_6)): δ = 201.1, 171.0, 171.0, 149.9, 142.5, 135.3, 134.6, 130.6, 127.6, 48.2, 43.6, 40.5, 38.0, 34.5, 34.3, 32.5, 32.2, 28.6, 25.0, 21.1,

17.1. IR (KBr): 3463, 2962, 1683, 1452, 1238, 1041 cm^{-1}. HRMS-ESI (*m/z*): calcd for $C_{21}H_{27}O_5$ [M - H]$^-$: 359.1864; found: 359.1867.

(*E*)-2-(2-Oxo-4-(2,6,6-trimethylcyclohex-1-en-1-yl)but-3-en-1-yl)malonic acid (46)

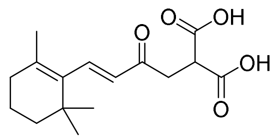

1aa (0.3 mmol, 61.9 mg) was used as starting material and **46** was obtained after purification by column chromatography on silica gel using CH_2Cl_2/CH_3OH (v/v = 20:1) as the eluent. White solid (44.2 mg, 50%), mp: 158 – 159 °C. ^{1}H NMR (400 MHz, DMSO(d_6)): δ = 12.11 (s, 2H), 7.29 (d, J = 16.4 Hz, 1H), 6.16 (d, J = 16.0 Hz, 1H), 3.64 (t, J = 7.2 Hz, 1H), 3.12 (d, J = 7.2 Hz, 2H), 2.06 (t, J = 6.4 Hz, 2H), 1.74 (s, 3H), 1.62 – 1.53 (m, 2H), 1.47 – 1.41 (m, 2H), 1.05 (s, 6H). $^{13}C\{^1H\}$ NMR (100 MHz, DMSO(d_6)): δ = 197.3, 170.9, 141.9, 137.2, 136.0, 130.0, 47.7, 40.0, 39.7, 34.3, 33.7, 29.1, 22.0, 19.0. IR (KBr): 3460, 2928, 1651, 1454, 1368, 1243 cm^{-1}. HRMS-ESI (*m/z*): calcd for $C_{16}H_{23}O_5$ [M + H]$^+$: 295.1540; found: 295.1538.

2-(2-(3-(Cyclopropylmethoxy)-4-(difluoromethoxy)phenyl)-2-oxoethyl)malonic acid (47)

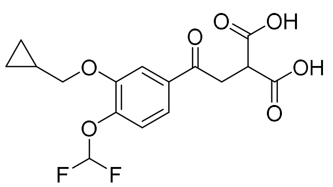

1ab (0.3 mmol, 81.0 mg) was used as starting material and **47** was obtained after purification by column chromatography on silica gel using CH_2Cl_2/CH_3OH (v/v = 20:1) as the eluent. White solid (66.7 mg, 62%), mp: 138 – 140 °C. ^{1}H NMR (400 MHz, DMSO(d_6)): δ = 12.59 (s, 2H), 7.69 – 7.57 (m, 2H), 7.31 (d, J = 8.4 Hz, 1H), 7.25 (d, J = 73.9 Hz, 1H), 3.99 (d, J = 7.2 Hz, 2H), 3.75 (t, J = 7.2 Hz, 1H), 3.53 (d, J = 7.2 Hz, 2H), 1.34 – 1.21 (m, 1H), 0.65 – 0.52 (m, 2H), 0.46 – 0.31 (m, 2H). $^{13}C\{^1H\}$ NMR (100 MHz, DMSO(d_6)): δ = 196.5, 171.0, 150.1, 144.4, 134.4, 122.0, 120.5, 116.90 (t, J = 257.0 Hz), 113.8, 73.8, 47.7, 38.1, 10.4, 3.6. IR (KBr): 3463, 1668, 1413, 1270, 1125 cm^{-1}. HRMS-ESI (*m/z*): calcd for $C_{16}H_{17}F_2O_7$ [M + H]$^+$: 359.0937; found: 359.0933.

2-(2-(4-(*N*,*N*-Dipropylsulfamoyl)phenyl)-2-oxoethyl)malonic acid (48)

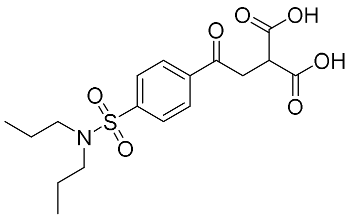

1ac (0.3 mmol, 89.1 mg) was used as starting material and **48** was obtained after purification by column chromatography on silica gel using CH_2Cl_2/CH_3OH (v/v = 10:1) as the eluent. White solid (53.2 mg, 46%), mp: 145 – 147 °C. ^{1}H NMR (400 MHz, DMSO(d_6)): δ = 8.17 (d, J = 8.4 Hz, 2H), 7.93 (d, J = 8.0 Hz, 2H), 3.78 (t, J = 6.8 Hz, 1H), 3.57 (d,

J = 7.2 Hz, 2H), 3.38 (s, 2H), 3.09 – 3.00 (m, 4H), 1.54 – 1.39 (m, 4H), 0.81 (t, J = 7.6 Hz, 6H). $^{13}C\{^1H\}$ NMR (100 MHz, DMSO(d_6)): δ = 197.2, 170.7, 144.0, 139.2, 129.5, 127.7, 50.2, 47.7, 38.4, 22.1, 11.5. IR (KBr): 2925, 1749, 1388, 1243, 1164, 987 cm^{-1}. HRMS-ESI (m/z): calcd for $C_{17}H_{24}NO_7S$ [M + H]$^+$: 386.1268; found: 386.1267.

2-(2-(6-(3-((3*r*,5*r*,7*r*)-Adamantan-1-yl)-4-methoxyphenyl)naphthalen-2-yl)-2-oxoethyl)malonic acid (49)

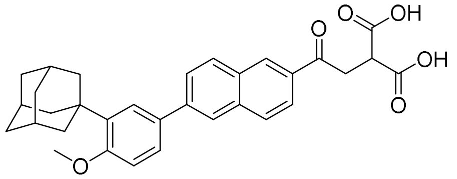

1ad (0.3 mmol, 127.3 mg) was used as starting material and **49** was obtained after purification by column chromatography on silica gel using CH_2Cl_2/CH_3OH (v/v = 10:1) as the eluent. White solid (63.0 mg, 41%), mp: 185 – 187 °C. ^1H NMR (400 MHz, DMSO(d_6)): δ = 12.84 (s, 2H), 8.76 (s, 1H), 8.23 – 8.18 (m, 2H), 8.08 (d, J = 8.8 Hz, 1H), 7.98 (dd, J = 8.4, 1.6 Hz, 1H), 7.92 (dd, J = 8.4, 2.0 Hz, 1H), 7.67 (dd, J = 8.4, 2.0 Hz, 1H), 7.59 (d, J = 2.4 Hz, 1H), 7.12 (d, J = 8.8 Hz, 1H), 3.86 (s, 3H), 3.82 (t, J = 7.2 Hz, 1H), 3.69 (d, J = 6.8 Hz, 2H), 2.13 (s, 6H), 2.06 (s, 3H), 1.75 (t, J = 3.2 Hz, 6H). $^{13}C\{^1H\}$ NMR (100 MHz, DMSO(d_6)): δ = 197.4, 171.1, 159.2, 141.1, 138.6, 136.2, 133.4, 131.9, 131.5, 130.8, 130.4, 129.0, 126.5, 126.3, 125.6, 124.5, 124.2, 113.2, 55.9, 47.7, 40.6, 38.1, 37.1, 37.1, 28.9. IR (KBr): 3335, 3212, 2910, 1717, 1461, 1352, 1240, 958 cm^{-1}. HRMS-ESI (m/z): calcd for $C_{32}H_{33}O_6$ [M + H]$^+$: 513.2272; found: 513.2270.

E. Procedure for the synthesis of malonic acid derivatives 32-41

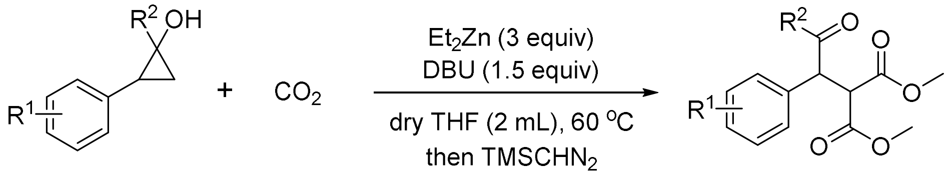

To a 25 mL oven-dried Schlenk tube equipped with a magnetic stirring bar was added cyclopropanol **1** (0.3 mmol). The tube was then evacuated, refilled with CO_2 (1 atm) three times, and charged with dry THF (2 mL), 1,8-diazabicyclo[5.4.0]undec-7-ene (67 μL, 0.45 mmol, 1.5 equiv) and Et_2Zn (0.9 mL, 1 M in toluene, 3 equiv) successively via a syringe. The reaction mixture was stirred at 60 °C for 20 h. After the reaction was completed, the reaction mixture was quenched with NH_4Cl saturated solution (5 mL), acidified to pH = 1 with HCl (2 M) and extracted with ethyl acetate (10 mL × 3). Then combined organic layers were dried over anhydrous Na_2SO_4 and then

filtered. After removing the solvent under vacuum, the crude product was dissolved in Et_2O (1.6 mL) and MeOH (0.4 mL) at 0 °C. Then, trimethylsilyldiazomethane (8 equiv, 2 M in diethyl ether, 1.2 mL) was added dropwise to the mixture, and the resulting mixture was stirred at room temperature for 1 h. After removing the solvent under vacuum, the residue was purified by column chromatography on silica gel using petroleum ether/ethyl acetate (v/v = 2:1-5:1) as the eluent to give the desired products.

Dimethyl 2-(2-oxo-1-phenylpropyl)malonate (32)

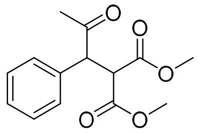

1al (0.3 mmol, 44.4 mg) was used as starting material and **32** was obtained after purification by column chromatography on silica gel using petroleum/EtOAc (v/v = 5:1) as the eluent. White solid (53.1 mg, 67%), mp: 79 – 81 °C. ^{1}H NMR (400 MHz, $CDCl_3$): δ = 7.35 – 7.27 (m, 3H), 7.24 – 7.18 (m, 2H), 4.46 (d, J = 11.6 Hz, 1H), 4.24 (d, J = 11.6 Hz, 1H), 3.75 (s, 3H), 3.42 (s, 3H), 2.14 (s, 3H). $^{13}C\{^{1}H\}$ NMR (100 MHz, $CDCl_3$): δ = 205.4, 168.6, 168.0, 133.8, 129.1, 128.8, 128.3, 57.9, 54.4, 52.9, 52.4, 28.8. IR (KBr): 3465, 2922, 1648, 1235, 1142, 690 cm^{-1}. HRMS-ESI (*m/z*): calcd for $C_{14}H_{15}O_5$ [M - H]$^{-}$: 263.0925; found: 263.0924.

Dimethyl 2-(2-oxo-1-(*p*-tolyl)propyl)malonate (33)

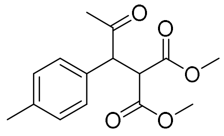

1am (0.3 mmol, 48.6 mg) was used as starting material and **33** was obtained after purification by column chromatography on silica gel using petroleum/EtOAc (v/v = 5:1) as the eluent. White solid (54.2 mg, 65%), mp: 80 – 81 °C. ^{1}H NMR (400 MHz, $CDCl_3$): δ = 7.13 – 7.06 (m, 4H), 4.41 (d, J = 11.6 Hz, 1H), 4.20 (d, J = 11.6 Hz, 1H), 3.73 (s, 3H), 3.44 (s, 3H), 2.30 (s, 3H), 2.12 (s, 3H). $^{13}C\{^{1}H\}$ NMR (100 MHz, $CDCl_3$): δ = 205.6, 168.6, 168.1, 138.1, 130.8, 129.8, 128.6, 57.6, 54.5, 52.8, 52.4, 28.8, 21.1. IR (KBr): 3465, 2923, 1648, 1236, 1140, 798 cm^{-1}. HRMS-ESI (*m/z*): calcd for $C_{15}H_{19}O_5$ [M + H]$^{+}$: 279.1227; found: 279.1222.

Dimethyl 2-(1-(4-(*t*-butyl)phenyl)-2-oxopropyl)malonate (34)

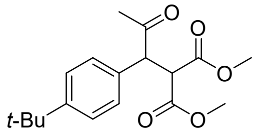

1an (0.3 mmol, 61.3 mg) was used as starting material and **34** was obtained after purification by column chromatography on silica gel using petroleum/EtOAc (v/v = 5:1) as the eluent. Colorless oil (70.1 mg, 73%).

^{1}H NMR (400 MHz, $CDCl_3$): δ = 7.33 – 7.26 (m, 2H), 7.13 – 7.06 (m, 2H), 4.41 (d, J = 11.6 Hz, 1H), 4.19 (d, J = 11.6 Hz, 1H), 3.71 (s, 3H), 3.38 (s, 3H), 2.11 (s, 3H), 1.26 (s, 9H). $^{13}C\{^1H\}$ NMR (100 MHz, $CDCl_3$): δ = 205.5, 168.6, 168.0, 151.1, 130.6, 128.3, 125.9, 57.4, 54.4, 52.7, 52.2, 34.4, 31.1, 28.8. IR (KBr): 2959, 1739, 1510, 1440, 1286, 1156, 1027, 830 cm^{-1}. HRMS-ESI (*m/z*): calcd for $C_{18}H_{25}O_5$ $[M + H]^+$: 321.1697; found: 321.1691.

Dimethyl 2-(1-(4-methoxyphenyl)-2-oxopropyl)malonate (35)

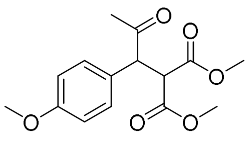

1ao (0.3 mmol, 53.4 mg) was used as starting material and **35** was obtained after purification by column chromatography on silica gel using petroleum/EtOAc (v/v = 5:1) as the eluent. White solid (58.3 mg, 66%), mp: 55 – 57 °C. ^{1}H NMR (400 MHz, $CDCl_3$): δ = 87.11 – 7.07 (m, 2H), 6.85 – 6.79 (m, 2H), 4.37 (d, J = 11.6 Hz, 1H), 4.16 (d, J = 11.6 Hz, 1H), 3.74 (s, 3H), 3.70 (s, 3H), 3.42 (s, 3H), 2.09 (s, 3H). $^{13}C\{^1H\}$ NMR (100 MHz, $CDCl_3$): δ = 205.6, 168.5, 168.0, 159.4, 129.8, 125.6, 114.4, 57.0, 55.1, 54.4, 52.7, 52.3, 28.6. IR (KBr): 3470, 2954, 1737, 1612, 1511, 1443, 1251, 1165, 1030, 827 cm^{-1}. HRMS-ESI (*m/z*): calcd for $C_{15}H_{19}O_6$ $[M + H]^+$: 295.1176; found: 295.1170.

Dimethyl 2-(1-(4-bromophenyl)-2-oxopropyl)malonate (36)

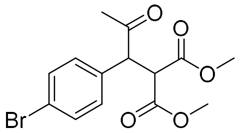

1ap (0.3 mmol, 67.8 mg) was used as starting material and **36** was obtained after purification by column chromatography on silica gel using petroleum/EtOAc (v/v = 5:1) as the eluent. White solid (39.1 mg, 38%), mp: 58 – 60 °C. ^{1}H NMR (400 MHz, $CDCl_3$): δ = 7.48 – 7.43 (m, 2H), 7.12 – 7.06 (m, 2H), 4.41 (d, J = 11.8 Hz, 1H), 4.19 (d, J = 11.6 Hz, 1H), 3.73 (s, 3H), 3.46 (s, 3H), 2.13 (s, 3H). $^{13}C\{^1H\}$ NMR (100 MHz, $CDCl_3$): δ = 204.9, 168.3, 167.8, 132.9, 132.3, 130.4, 122.6, 57.2, 54.3, 52.9, 52.5, 28.9. IR (KBr): 2959, 1739, 1510, 1440, 1286, 1156, 1027, 830 cm^{-1}. HRMS-ESI (*m/z*): calcd for $C_{14}H_{16}BrO_5$ $[M + H]^+$: 343.0176; found: 343.0168.

Dimethyl 2-(2-oxo-1-(*m*-tolyl)propyl)malonate (37)

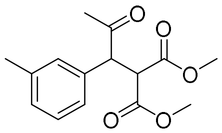

1aq (0.3 mmol, 48.6 mg) was used as starting material and **37** was obtained after purification by column chromatography on silica gel using petroleum/EtOAc (v/v = 5:1) as the eluent. White solid (41.8 mg, 50%), mp: 84 – 86 °C. ^{1}H NMR (400 MHz, $CDCl_3$): δ = 7.19 (t, J = 8.0 Hz, 1H), 7.11 – 7.06 (m, 1H), 7.02 –

6.96 (m, 2H), 4.40 (d, *J* = 11.6 Hz, 1H), 4.20 (d, *J* = 11.2 Hz, 1H), 3.73 (s, 3H), 3.42 (s, 3H), 2.30 (s, 3H), 2.12 (s, 3H). $^{13}C\{^1H\}$ NMR (100 MHz, $CDCl_3$): δ = 205.5, 168.6, 168.0, 138.7, 133.7, 129.2, 129.0, 128.9, 125.9, 57.9, 54.4, 52.8, 52.3, 28.8, 21.3. IR (KBr): 2956, 1743, 1451, 1274, 1165, 743 cm^{-1}. HRMS-ESI (*m/z*): calcd for $C_{13}H_{13}O_5$ $[M - H]^-$: 249.0768; found: 249.0766.

Dimethyl 2-(1-(2-fluorophenyl)-2-oxopropyl)malonate (38)

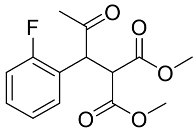

1ar (0.3 mmol, 49.8 mg) was used as starting material and **38** was obtained after purification by column chromatography on silica gel using petroleum/EtOAc (v/v = 5:1) as the eluent. White solid (27.1 mg, 32%), mp: 59 – 61 °C. ^1H NMR (400 MHz, $CDCl_3$): δ = 7.33 – 7.25 (m, 1H), 7.19 (td, *J* = 7.6, 2.0 Hz, 1H), 7.15 – 7.05 (m, 1H), 4.80 (d, *J* = 11.6 Hz, 1H), 4.27 (d, *J* = 11.6 Hz, 1H), 3.76 (s, 3H), 3.45 (s, 3H), 2.13 (s, 3H). $^{13}C\{^1H\}$ NMR (100 MHz, $CDCl_3$): δ = 204.2, 168.4, 167.8, 160.8 (d, *J* = 246.5 Hz), 130.1 (d, *J* = 8.1 Hz), 130.0 (d, *J* = 3.2 Hz), 124.8 (d, *J* = 3.6 Hz), 121.3 (d, *J* = 15.0 Hz), 116.0 (d, *J* = 22.1 Hz), 53.1, 52.9, 52.5, 50.6, 28.6. IR (KBr): 3462, 1646, 1237, 1145, 752 cm^{-1}. HRMS-ESI (*m/z*): calcd for $C_{14}H_{14}FO_5$ $[M - H]^-$: 281.0831; found: 281.0830.

Dimethyl 2-(1-(naphthalen-2-yl)-2-oxobutyl)malonate (39)

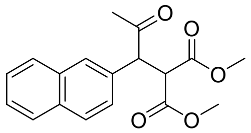

1as (0.3 mmol, 59.4 mg) was used as starting material and **39** was obtained after purification by column chromatography on silica gel using petroleum/EtOAc (v/v = 2:1) as the eluent. Colorless oil (53.9 mg, 57%). ^1H NMR (400 MHz, $CDCl_3$): δ = 7.86 – 7.78 (m, 3H), 7.71 (s, 1H), 7.52 – 7.47 (m, 2H), 7.32 (dd, *J* = 8.8, 2.0 Hz, 1H), 4.64 (d, *J* = 11.6 Hz, 1H), 4.36 (d, *J* = 11.6 Hz, 1H), 3.78 (s, 3H), 3.37 (s, 3H), 2.17 (s, 3H). $^{13}C\{^1H\}$ NMR (100 MHz, $CDCl_3$): δ = 205.4, 168.6, 168.0, 133.4, 133.0, 131.4, 129.0, 128.3, 127.9, 127.7, 126.5, 126.5, 126.0, 58.1, 54.5, 52.9, 52.4, 29.0. IR (KBr): 3042, 2947, 1735, 1441, 1281, 1157, 749 cm^{-1}. HRMS-ESI (*m/z*): calcd for $C_{18}H_{17}O_5$ $[M - H]^-$: 313.1081; found: 313.1082.

Dimethyl 2-(1-(benzofuran-5-yl)-2-oxopropyl)malonate (40)

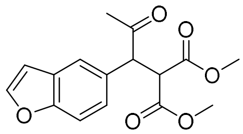

1at (0.3 mmol, 56.4 mg) was used as starting material and **40** was obtained after purification by column chromatography on silica gel using petroleum/EtOAc (v/v = 2:1) as the eluent. White solid (59.3 mg, 65%), mp: 107 – 109 °C. ^1H NMR (400 MHz, $CDCl_3$): δ = 7.59 (d, *J* = 2.4 Hz, 1H), 7.50 – 7.38 (m, 2H), 7.11

(dd, J = 8.4, 2.0 Hz, 1H), 6.70 (d, J = 2.4 Hz, 1H), 4.53 (d, J = 11.6 Hz, 1H), 4.25 (d, J = 11.6 Hz, 1H), 3.72 (s, 3H), 3.36 (s, 3H), 2.11 (s, 3H). $^{13}C\{^1H\}$ NMR (100 MHz, $CDCl_3$): δ = 205.6, 168.5, 168.0, 154.5, 145.7, 128.2, 128.0, 124.8, 121.5, 111.9, 106.4, 57.6, 54.7, 52.7, 52.3, 28.7. IR (KBr): 2950, 1736, 1449, 1278, 1159, 1027, 755 cm^{-1}. HRMS-ESI (m/z): calcd for $C_{16}H_{17}O_6$ $[M + H]^+$: 305.1020; found: 305.1015.

Dimethyl 2-(2-oxo-1-phenylpentyl)malonate (41)

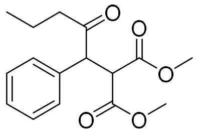

1au (0.3 mmol, 52.8 mg) was used as starting material and **41** was obtained after purification by column chromatography on silica gel using petroleum/EtOAc (v/v = 5:1) as the eluent. Colorless oil (44.6 mg, 51%). ^1H NMR (400 MHz, $CDCl_3$): δ = 7.34 – 7.25 (m, 3H), 7.23 – 7.18 (m, 2H), 4.44 (d, J = 11.2 Hz, 1H), 4.26 (d, J = 11.2i Hz, 1H), 3.74 (s, 3H), 3.42 (s, 3H), 2.55 – 2.45 (m, 1H), 2.42 – 2.30 (m, 1H), 1.63 – 1.44 (m, 2H), 0.78 (t, J = 7.4 Hz, 3H). $^{13}C\{^1H\}$ NMR (100 MHz, $CDCl_3$): δ = 207.5, 168.5, 168.1, 134.0, 128.9, 128.7, 128.1, 57.3, 54.4, 52.7, 52.3, 43.3, 16.9, 13.3. IR (KBr): 2955, 1735, 1443, 1280, 1026, 745, 698 cm^{-1}. HRMS-ESI (m/z): calcd for $C_{16}H_{19}O_5$ $[M - H]^-$: 291.1238; found: 291.1239.

F. Procedure for the synthesis of compounds 50-56

i) Procedure for the synthesis of compound **50** (ref.[4])

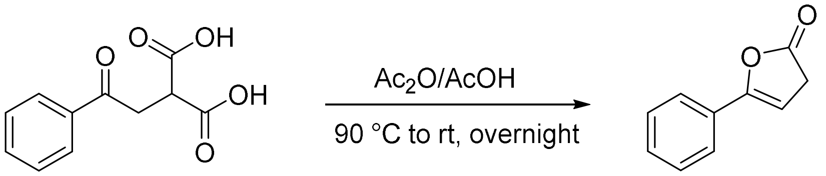

To a solution of Ac_2O (1 mL) in AcOH (1.7 mL) was added **2** (0.5 mmol). The resulting mixture was stirred at 90 °C for 4 h, then cooled to room temperature and stirred overnight. The reaction mixture was quenched with H_2O (10 mL) and extracted with ethyl acetate (10 mL × 3). The combined organic layer was washed with saturated brine water, dried over anhydrous Na_2SO_4 and then filtered. After removing the solvent under vacuum, the residue was purified by column chromatography on silica gel using petroleum ether/ethyl acetate (v/v = 20:1) as the eluent to afford the desired product **50**[5] as yellow solid (65.6 mg, 82%). Mp: 70 – 72 °C. ^1H NMR (400 MHz, $CDCl_3$): δ = 7.65 – 7.57 (m, 2H), 7.43 – 7.36 (m, 3H), 5.78 (t, J = 2.8 Hz, 1H), 3.40 (d, J = 2.8 Hz, 2H).

$^{13}C\{^1H\}$ NMR (100 MHz, $CDCl_3$): δ = 175.8, 153.9, 129.5, 128.6, 128.3, 124.6, 97.6, 34.5. IR (KBr): 2928, 1792, 1254, 1002, 722 cm^{-1}. HRMS-ESI (m/z): calcd for $C_{10}H_9O_2$ $[M + H]^+$: 161.0597; found: 161.0598.

ii) Procedure for the synthesis of compound **51** (ref.[4])

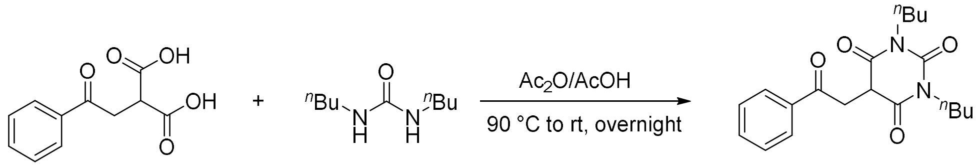

2 **51**, 78%

To a solution of Ac_2O (1 mL) in AcOH (1.7 mL) was added **2** (0.5 mmol) and dibutylurea (0.5 mmol). The reaction mixture was stirred at 90 °C for 4 hours, then cooled to room temperature and stirred overnight. The reaction mixture was quenched with H_2O (10 mL), and extracted with ethyl acetate (10 mL × 3). The combined organic layer was washed with saturated brine water, dried over anhydrous Na_2SO_4 and then filtered. After removing the solvent under vacuum, the residue was purified by column chromatography on silica gel using petroleum ether/ethyl acetate (v/v = 5:1) as the eluent to afford the desired product **51** as light yellow solid (83.6 mg, 78%). Mp: 45 – 46 °C. ^1H NMR (400 MHz, $CDCl_3$): δ = 7.97 – 7.90 (m, 2H), 7.61 – 7.55 (m, 1H), 7.49 – 7.42 (m, 2H), 4.01 (d, J = 3.6 Hz, 2H), 3.97 – 3.83 (m, 4H), 3.55 (t, J = 3.6 Hz, 1H), 1.66 – 1.55 (m, 4H), 1.36 (h, J = 7.6 Hz, 4H), 0.93 (t, J = 7.2 Hz, 6H). $^{13}C\{^1H\}$ NMR (100 MHz, $CDCl_3$): δ = 196.8, 167.8, 151.2, 135.5, 133.8, 128.7, 128.3, 44.6, 42.1, 37.9, 29.9, 20.0, 13.7. IR (KBr): 2956, 1683, 1416, 1363, 1210, 753 cm^{-1}. HRMS-ESI (m/z): calcd for $C_{20}H_{25}N_2O_4$ $[M - H]^-$: 357.1820; found: 357.1819.

iii) Procedure for the synthesis of compound **52** (ref.[6])

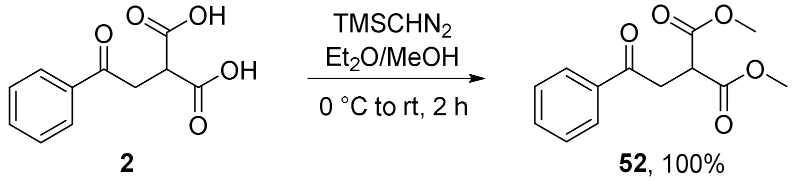

2 **52**, 100%

To a mixed solvent of diethyl ether (1.6 mL) and MeOH (0.4 mL) was added **2** (0.3 mmol). Then, trimethylsilyldiazomethane (8 equiv, 2 M in diethyl ether, 1.2 mL) was added dropwise to the mixture at 0 °C. The resulting mixture was stirred at room temperature for 2 h. After removing the solvent under vacuum, the residue was purified by column chromatography on silica gel using using petroleum ether/ethyl acetate (v/v = 5:1) as the eluent to give the desired product **52**[7] as white solid

(74.9 mg, 100%). Mp: 78 – 80 °C. ^{1}H NMR (400 MHz, $CDCl_3$): δ = 8.00 – 7.93 (m, 2H), 7.60 – 7.54 (m, 1H), 7.46 (t, J = 7.6 Hz, 2H), 4.08 (t, J = 7.2 Hz, 1H), 3.77 (s, 6H), 3.64 (d, J = 7.2 Hz, 2H). $^{13}C\{^1H\}$ NMR (100 MHz, $CDCl_3$): δ = 196.4, 169.4, 135.9, 133.5, 128.6, 128.1, 52.8, 46.7, 37.9. IR (KBr):2937, 1781, 1673, 1425, 1203, 794 cm^{-1}.

iv) Procedure for the synthesis of compound **53** (ref.[8])

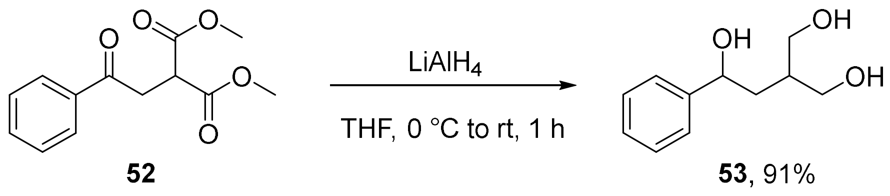

To a solution of **52** (0.3 mmol) in dry THF (2 mL) was added $LiAlH_4$ (60 μL, 2.5 M in THF, 0.5 equiv) slowly at 0 °C under N_2 atmosphere. The resulting mixture was stirred at room temperature for 1 h. The reaction mixture was quenched by H_2O (10 mL), and extracted with ethyl acetate (10 mL × 3). The combined organic layer was washed with saturated brine water, dried over anhydrous Na_2SO_4 and then filtered. After removing the solvent under vacuum, the residue was purified by column chromatography on silica gel using petroleum ether/ethyl acetate (v/v = 1:1) as the eluent to afford the desired product **53**[8] as colorless oil (53.6 mg, 91%). ^{1}H NMR (400 MHz, $CDCl_3$): δ = 7.31 (d, J = 4.4 Hz, 4H), 7.25 – 7.21 (m, 1H), 4.75 (dd, J = 8.8, 3.6 Hz, 1H), 3.77 – 3.54 (m, 4H), 3.22 (s, 3H), 1.96 – 1.85 (m, 1H), 1.82 – 1.62 (m, 2H). $^{13}C\{^1H\}$ NMR (100 MHz, $CDCl_3$): δ = 144.7, 128.5, 127.5, 125.7, 72.5, 64.9, 64.7, 40.5, 38.7. IR (KBr): 3343, 2922, 1647, 1535, 1451, 1251, 1025, 755, 693 cm^{-1}.

v) Procedure for the synthesis of compound **54** (ref.[9])

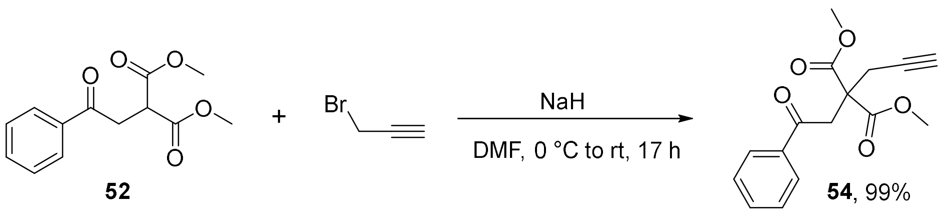

To a solution of NaH (0.33 mmol, 1.1 equiv) in dry DMF (2 mL) was slowly added **52** (0.3 mmol) at 0 °C and the resulting mixture was stirred at 0 °C for 1 h. Then, the reaction mixture was warmed to room temperature and propargyl bromide (0.36 mmol, 1.2 equiv) was added dropwise. After stirring at room temperature for 16 h, the reaction mixture was quenched with 3 mL 2 M HCl and extracted with diethyl ether (10 mL × 3). The combined organic layer was washed with saturated

brine water, dried over anhydrous Na_2SO_4 and then filtered. After removing the solvent under vacuum, the residue was purified by column chromatography on silica gel using petroleum ether/ethyl acetate (v/v = 5:1) as the eluent to give the desired product **54**[10] as colorless oil (85.6 mg, 99%). ^{1}H NMR (400 MHz, $CDCl_3$): δ = 8.01 – 7.96 (m, 2H), 7.61 – 7.54 (m, 1H), 7.49 – 7.43 (m, 2H), 3.91 (s, 2H), 3.75 (s, 6H), 3.11 (d, J = 2.8 Hz, 2H), 2.01 (t, J = 2.8 Hz, 1H). ^{13}C{^{1}H} NMR (100 MHz, $CDCl_3$): δ = 196.6, 169.6, 136.1, 133.5, 128.6, 128.0, 79.1, 71.8, 54.5, 53.1, 40.9, 23.3. IR (KBr): 3285, 2952, 1744, 1686, 1441, 1206, 1064, 984, 756, 681 cm^{-1}.

vi) Procedure for the synthesis of compound **55** (ref.[11])

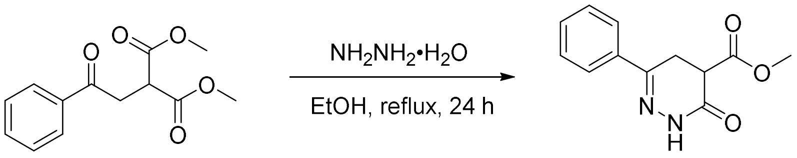

52 **55**, 57%

A mixture of **52** (0.3 mmol) and hydrazine monohydrate (15 μL, 0.3 mmol, 1 equiv) in anhydrous EtOH (2 mL) was heated to reflux for 24 h. the reaction mixture was then cooled to room temperature, and the precipitated solid was removed by filtration. The filtrate was concentrated in vacuum, and the residue was purified by column chromatography on silica gel using petroleum ether/ethyl acetate (v/v = 2:1) as the eluent to give the desired product **55**[12] as white solid (39.7 mg, 57%). Mp: 162 – 164 °C. ^{1}H NMR (400 MHz, $CDCl_3$): δ = 9.00 (s, 1H), 7.78 – 7.67 (m, 2H), 7.47 – 7.39 (m, 3H), 3.80 (s, 3H), 3.68 – 3.59 (m, 1H), 3.45 (dd, J = 17.2, 8.8 Hz, 1H), 3.11 (dd, J = 16.8, 6.8 Hz, 1H). ^{13}C{^{1}H} NMR (100 MHz, $CDCl_3$): δ = 168.5, 162.9, 150.7, 135.0, 130.2, 128.7, 126.0, 53.1, 43.2, 25.8. IR (KBr): 3218, 2925, 1731, 1667, 1342, 1253, 1200, 761 cm^{-1}.

vii) Procedure for the synthesis of compound **56** (ref.[13])

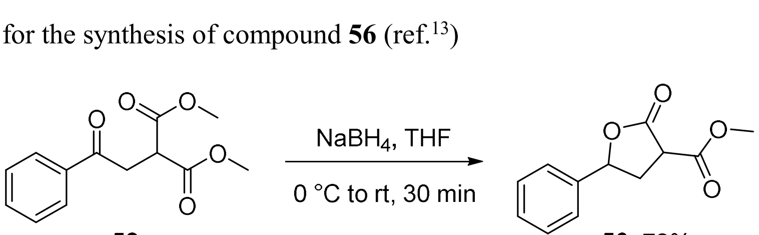

52 **56**, 72%

To a solution of **52** (0.3 mmol) in dry MeOH (2 mL) at 0 °C was slowly added $NaBH_4$ (0.3mol, 1 equiv), and the mixture was stirred at room temperature for 30 mins. After the reaction was completed, the reaction mixture was quenched with 3 mL 2 M HCl, and extracted with ethyl acetate (10 mL × 3). The combined organic layer was washed with brine (10 mL) and dried over anhydrous

Na_2SO_4. After filtration and evaporation of the solvent under vacuum, the residue was purified by column chromatography on silica gel using petroleum ether/ethyl acetate (v/v = 10:1) as the eluent to obtain the desired product **56** as white solid (47.5 mg, 72%, d.r. = 1.5:1). ^{1}H NMR (400 MHz, $CDCl_3$): (two diastereomers) δ = 7.46 – 7.37 (m, 7H), 7.37 – 7.32 (m, 2H), 5.73 (t, J = 7.2 Hz, 1H), 5.46 (dd, J = 10.4, 6.0 Hz, 1H), 3.85 (s, 3H), 3.83 (s, 3H), 3.83 – 3.78 (m, 1H), 3.74 (dd, J = 9.2, 4.8 Hz, 1H), 3.10 – 3.01 (m, 1H), 2.94 – 2.84 (m, 1H), 2.75 – 2.63 (m, 1H), 2.52 – 2.40 (m, 1H). ^{13}C{^{1}H} NMR (100 MHz, $CDCl_3$): (two diastereomers) δ = 171.6, 171.5, 168.8, 168.0, 138.6, 137.9, 129.0, 128.9, 128.9, 128.8, 125.8, 125.3, 80.5, 80.1, 53.3, 53.1, 47.7, 46.8, 34.9, 34.8. IR (KBr): 3463, 2848, 1646, 1155, 696 cm^{-1}. HRMS-ESI (m/z): calcd for $C_{12}H_{11}O_4$ [M - H]$^-$: 219.0663; found: 219.0657.

G. X-ray crystal structure and data for compound 37

Single-crystal X-ray diffraction data for **37** was collected on an X-ray diffractometer operated at 90 kV and 50 mA using MoKα radiation (λ = 0.71073 Å) at 100 K. All empirical absorption corrections were performed using the CrystalClear program. The structure was solved by a direct method and refined on F^2 by the full-matrix least squares technique using the SHELXTL-97 program package. All non-hydrogen atoms were refined with anisotropic displacement parameters. Hydrogen atoms attached to carbon were placed in geometrically idealized positions and refined using a riding model. The X-ray crystal structure of compound **37** is shown in Figure S1, and the crystallographic data for compound **37** is given in Table S1.

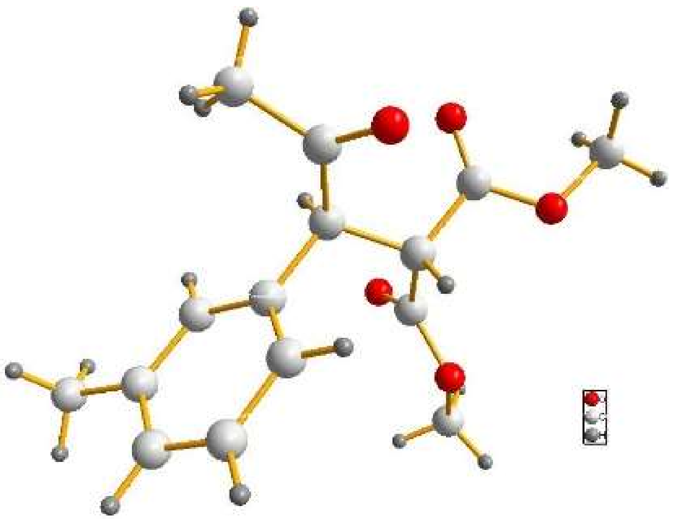

Figure S1. X-ray crystal structures of compound **37**.

Table S2. Crystal data and structure refinements for **37**.

Compound	**37**
Empirical formula	$C_{15}H_{18}O_5$
Formula weight	278.29
Temperature (K)	100
Wavelength (Å)	0.71073
Crystal system	monoclinic
Space group	$P2_1/c$
	a = 8.1012(4) Å α = 90°
	b = 18.3892(9) Å β = 104.417(2)°
	c = 9.8673(6) Å γ = 90°
Volume (Å^3)	1423.69(13)
Z	4
Density (calcd g cm^{-3})	1.298
Absorption coeff. (mm^{-1})	0.097
F(000)	592.0
Crystal size (mm)	0.15 × 0.08 × 0.05
Crystal color and shape	Colorless block
θ range for data collection	4.804 to 52.752
Limiting indices	$-10 \leqslant h \leqslant 10$, $-22 \leqslant k \leqslant 20$, $-12 \leqslant l \leqslant 10$
Reflections collected	11097
Unique	2884 [R_{int} = 0.0440, R_{sigma} = 0.0425]
Refinement method	Full-matrix least-squares on F^2
Data/restraints/parameters	2884/0/185
Goodness-of-fit on F^2	1.029
Final R indexes [$I>=2\sigma (I)$]	$R_1 = 0.0432$, $wR_2 = 0.0968$
R indexes (all data)	$R_1 = 0.0625$, $wR_2 = 0.1084$

H. Computational Details

All calculations were carried out with Gaussian 16 program.[14] Geometrical optimizations and vibrational frequency analysis were performed at the M06-2X[15]/6-31G(d) level. The subsequent single-point calculations were carried out at the level of M06-2X/6-311+G(d,p) together with SMD implicit solvent model[16] for considering the solvation effect of THF. Gibbs free energies in solution

described in the text were obtained from such single-point energy calculations including gas-phase free energy corrections.

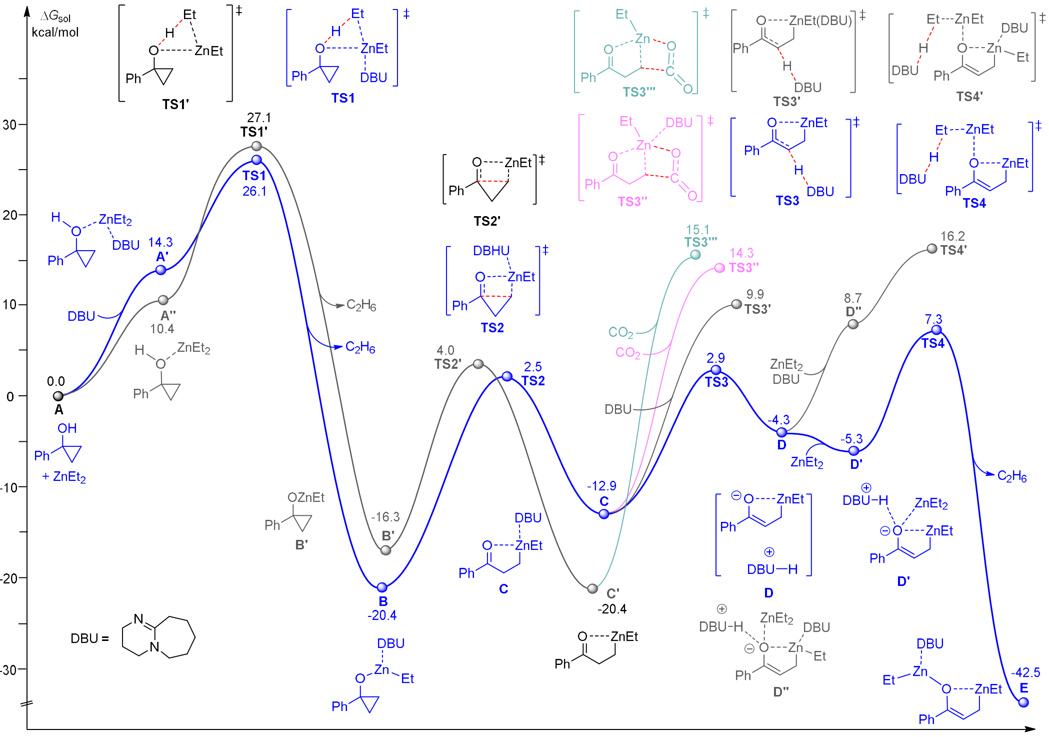

Figure S1-1. Energy profiles of Et_2Zn-mediated gem-dicarboxylation of 1-phenylcyclopropanol (**1a**) with CO_2 calculated at the level of M06-2X(SMD,THF)/6-311+G(d,p)//M06-2X/6-31G(d).

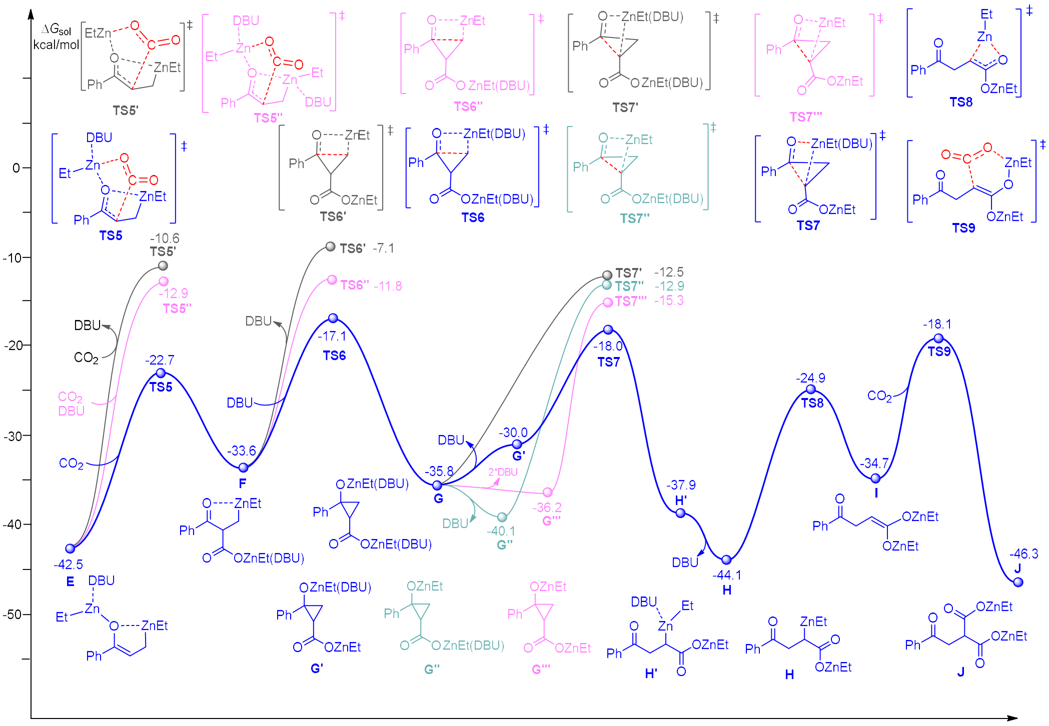

Figure S1-2. Energy profiles of Et_2Zn-mediated gem-dicarboxylation of 1-phenylcyclopropanol (**1a**) with CO_2 calculated at the level of M06-2X(SMD,THF)/6-311+G(d,p)//M06-2X/6-31G(d).

Optimized Coordinates Information

Optimized Cartesian coordinates and their corresponding single-point energies (E(SCRF), a.u.) calculated at the M06-2X/6-311+G(d,p) (SMD, THF)//M06-2X/6-31G(d) level as well as the imaginary frequency (IF, cm^{-1}) of transition states.

20 (the number of atoms involved in the molecule)

A, *E*(SCRF) = -424.114381062 a.u.

C	-2.083103000	-1.276084000	-0.055401000
C	-0.696492000	-1.181807000	-0.061734000
C	-0.064487000	0.065904000	-0.008985000
C	-0.856570000	1.213738000	0.058973000
C	-2.246305000	1.118760000	0.061091000
C	-2.866192000	-0.124681000	0.001565000
H	-2.554334000	-2.253499000	-0.096774000
H	-0.103994000	-2.092367000	-0.104063000
H	-0.374031000	2.182629000	0.128606000
H	-2.844693000	2.023380000	0.114940000
H	-3.949053000	-0.199188000	0.003702000
C	1.427343000	0.192215000	-0.010222000
C	2.282885000	-0.863610000	-0.662415000
H	3.171679000	-0.500976000	-1.167682000
H	1.796253000	-1.712079000	-1.132047000
C	2.251564000	-0.731993000	0.834458000
H	3.115820000	-0.287592000	1.315141000
H	1.722261000	-1.481055000	1.414396000
O	1.920578000	1.501362000	-0.058200000
H	1.763611000	1.835202000	-0.954598000

27

DBU, *E* (SCRF) = -462.023050714 a.u.

C	1.892848000	-1.251057000	0.617984000
C	0.885323000	-1.553584000	-0.505224000
C	1.964534000	1.306862000	0.294096000
C	-0.406429000	-0.779409000	-0.369789000
C	0.906279000	1.288868000	-0.814505000
H	3.317853000	-0.135394000	-0.539693000
H	2.563877000	-2.110985000	0.720026000
H	1.351852000	-1.372242000	-1.482780000
H	1.465122000	1.525107000	1.247607000
H	0.619197000	2.313246000	-1.073898000
C	-2.696072000	-0.671142000	0.013420000
H	-3.437551000	-1.288896000	0.528788000
C	-1.412050000	1.443970000	0.018108000

C	-2.436624000	0.621435000	0.784447000
H	-2.040530000	0.364892000	1.773445000
H	-3.350691000	1.205226000	0.927609000
H	-0.992158000	2.228314000	0.662677000
N	-0.321221000	0.597028000	-0.454803000
N	-1.477767000	-1.448016000	-0.144815000
H	1.330192000	0.851656000	-1.726773000
H	2.666013000	2.128386000	0.105294000
H	3.487312000	0.078538000	1.196497000
H	1.347984000	-1.167743000	1.567378000
H	0.590544000	-2.603506000	-0.475601000
H	-3.114362000	-0.427634000	-0.975571000
H	-1.887352000	1.946960000	-0.836665000

15

$ZnEt_2$, E(SCRF) = -385.604531539 a.u.

Zn	0.000031000	0.335016000	0.000002000
C	-1.924318000	0.336706000	0.343383000
H	-2.077064000	0.389353000	1.431158000
H	-2.340125000	1.272744000	-0.057138000
C	1.924386000	0.336756000	-0.343381000
H	2.077429000	0.389665000	-1.431094000
H	2.340084000	1.272715000	0.057458000
C	2.693691000	-0.861143000	0.231790000
H	2.334331000	-1.813703000	-0.177235000
H	3.768747000	-0.803180000	0.009166000
H	2.597099000	-0.924708000	1.322875000
C	-2.693854000	-0.861032000	-0.231831000
H	-2.334382000	-1.813737000	0.176756000
H	-3.768822000	-0.803101000	-0.008783000
H	-2.597652000	-0.924252000	-1.322970000

62

A', E(SCRF) = -1271.7628343 a.u.

Zn	0.059827000	0.511826000	0.238162000
O	1.501418000	-1.268879000	-0.172235000
H	1.689593000	-1.720130000	0.668540000
C	-0.168453000	0.047398000	2.231716000
C	4.530216000	0.932138000	1.726038000
C	5.808157000	1.017854000	1.185369000
H	7.065960000	0.472172000	-0.475910000
H	5.300876000	-0.751632000	-1.664461000
H	2.523694000	0.215751000	1.477338000
H	4.303402000	1.413972000	2.671970000
H	6.589699000	1.560535000	1.707292000
C	2.698059000	-1.104676000	-0.895471000

C	2.991207000	-2.191096000	-1.894782000
H	2.298315000	-3.025382000	-1.893632000
H	4.025580000	-2.448116000	-2.096674000
C	2.458879000	-0.860243000	-2.351934000
H	1.418165000	-0.805692000	-2.655320000
H	3.130834000	-0.168647000	-2.849847000
C	5.074488000	-0.288689000	-0.707706000
C	3.787114000	-0.384278000	-0.167997000
C	6.074356000	0.407278000	-0.038758000
C	0.537867000	1.988429000	-1.080876000
C	3.525075000	0.238601000	1.055403000
H	0.272871000	0.842337000	2.850232000
H	-1.245360000	0.091384000	2.460129000
C	0.369900000	-1.299296000	2.739643000
H	1.467585000	-1.352960000	2.670687000
H	0.135810000	-1.500676000	3.794650000
H	-0.028821000	-2.151153000	2.169685000
C	1.954812000	2.576221000	-1.020146000
H	2.206624000	2.925703000	-0.011301000
H	2.714408000	1.831428000	-1.283106000
H	2.093790000	3.429138000	-1.700174000
C	-4.837457000	2.233195000	0.211945000
C	-3.320391000	2.160915000	0.022147000
C	-2.877764000	1.256890000	-1.141645000
C	-5.517708000	0.893729000	0.502758000
C	-2.803554000	-0.196779000	-0.745326000
C	-5.271056000	-0.162636000	-0.581018000
H	-5.281213000	2.655664000	-0.700851000
H	-2.941953000	3.171187000	-0.165614000
H	-3.555703000	1.384558000	-1.995043000
H	-5.171957000	0.492130000	1.464909000
H	-6.029921000	-0.948780000	-0.521202000
C	-1.615962000	-2.157878000	-0.293170000
H	-0.598956000	-2.424192000	-0.001283000
C	-4.005198000	-2.085343000	0.300477000
C	-2.620378000	-2.436663000	0.819192000
H	-2.366897000	-1.811661000	1.682708000
H	-2.597596000	-3.482913000	1.137334000
H	-4.707943000	-1.962703000	1.134352000
N	-3.980325000	-0.834148000	-0.463240000
N	-1.634490000	-0.747663000	-0.658725000
H	-5.370252000	0.283785000	-1.576359000
H	-6.597572000	1.057495000	0.596528000
H	-5.067824000	2.934866000	1.021120000

H	-2.835196000	1.816695000	0.946287000
H	-1.880497000	1.535890000	-1.482278000
H	-1.868413000	-2.763547000	-1.177053000
H	-4.394427000	-2.887398000	-0.340977000
H	0.343514000	1.635534000	-2.105757000
H	-0.175931000	2.813867000	-0.925314000

35

A'', E(SCRF) = -809.721745135 a.u.

Zn	-2.396989000	0.290220000	-0.233019000
O	0.010744000	0.204283000	-0.511989000
H	0.112634000	1.157826000	-0.346607000
C	-2.207970000	2.273159000	0.004320000
C	4.056115000	0.682851000	-1.457599000
C	5.059273000	0.187948000	-0.631754000
H	5.485428000	-0.930869000	1.158627000
H	3.126154000	-1.317789000	1.714482000
H	1.939515000	0.831686000	-1.809231000
H	4.312996000	1.239358000	-2.353611000
H	6.103184000	0.358520000	-0.873984000
C	0.917594000	-0.480193000	0.323314000
C	0.461610000	-0.705981000	1.740921000
H	-0.498046000	-0.271403000	2.009335000
H	1.201613000	-0.695672000	2.534100000
C	0.451436000	-1.836967000	0.749814000
H	-0.509229000	-2.165575000	0.367685000
H	1.210928000	-2.607362000	0.834613000
C	3.375383000	-0.749908000	0.822001000
C	2.359553000	-0.249012000	-0.000229000
C	4.711854000	-0.534559000	0.508298000
C	-2.996672000	-1.609424000	-0.357007000
H	-2.372886000	-2.123589000	-1.100308000
H	-2.792752000	-2.111876000	0.599180000
C	2.715761000	0.463501000	-1.147107000
H	-1.692774000	2.695835000	-0.871107000
H	-3.196150000	2.747313000	0.006815000
C	-1.459914000	2.684757000	1.284287000
H	-0.450694000	2.252562000	1.346562000
H	-1.337868000	3.771654000	1.372150000
H	-1.988090000	2.347518000	2.183387000
C	-4.478904000	-1.793252000	-0.714968000
H	-4.718983000	-1.344770000	-1.685236000
H	-4.767037000	-2.850798000	-0.770657000
H	-5.134935000	-1.320939000	0.024543000

62

TS1, E(SCRF) = -1271.7407164 a.u., IF = i1006.1575 cm^{-1}

Zn	-0.000771000	0.665907000	-0.112986000
O	1.491259000	-0.895466000	-0.319518000
H	1.153719000	-0.658435000	0.754186000
C	0.409306000	-0.208422000	1.980965000
C	4.618392000	1.736695000	0.748878000
C	5.937060000	1.464346000	0.402971000
H	7.257665000	0.050354000	-0.544415000
H	5.474802000	-1.531281000	-1.120708000
H	2.582924000	1.061984000	0.713088000
H	4.366867000	2.654832000	1.272132000
H	6.727961000	2.164226000	0.653464000
C	2.786157000	-1.306385000	-0.593100000
C	3.048024000	-2.785077000	-0.420682000
H	2.216766000	-3.370488000	-0.040781000
H	4.030594000	-3.092159000	-0.075719000
C	2.916537000	-2.196929000	-1.797661000
H	1.990700000	-2.381876000	-2.331437000
H	3.801092000	-2.108394000	-2.420340000
C	5.219401000	-0.614322000	-0.595666000
C	3.886181000	-0.346947000	-0.257499000
C	6.232358000	0.279358000	-0.269415000
C	0.024635000	2.640692000	-0.536153000
C	3.604343000	0.836672000	0.426599000
H	0.914888000	0.619569000	2.495575000
H	-0.669598000	-0.044491000	2.147378000
C	0.793295000	-1.530041000	2.674771000
H	1.868522000	-1.726363000	2.577938000
H	0.555716000	-1.542795000	3.745729000
H	0.278542000	-2.386061000	2.218057000
C	1.274241000	3.413235000	-0.094626000
H	1.466775000	3.287806000	0.978685000
H	2.169186000	3.062138000	-0.619004000
H	1.190064000	4.492312000	-0.281740000
C	-5.232753000	1.914352000	0.209582000
C	-3.714057000	2.049772000	0.078161000
C	-3.106534000	1.233601000	-1.076480000
C	-5.734645000	0.492449000	0.466180000
C	-2.848506000	-0.204408000	-0.700212000
C	-5.299140000	-0.506486000	-0.610953000
H	-5.694942000	2.283442000	-0.716851000
H	-3.469504000	3.104041000	-0.086163000
H	-3.764013000	1.275785000	-1.953917000
H	-5.379301000	0.131393000	1.440600000

H	-5.937683000	-1.394321000	-0.584514000
C	-1.428277000	-2.034084000	-0.296179000
H	-0.386150000	-2.183088000	-0.013279000
C	-3.810024000	-2.257364000	0.284805000
C	-2.395392000	-2.460618000	0.800168000
H	-2.215217000	-1.844412000	1.688438000
H	-2.250231000	-3.507061000	1.081918000
H	-4.528435000	-2.239218000	1.113734000
N	-3.929740000	-0.988867000	-0.443748000
N	-1.618957000	-0.619074000	-0.611407000
H	-5.420689000	-0.069934000	-1.608053000
H	-6.829534000	0.504547000	0.512211000
H	-5.584835000	2.569923000	1.013275000
H	-3.220867000	1.762160000	1.016906000
H	-2.150692000	1.662215000	-1.378667000
H	-1.612396000	-2.626089000	-1.204814000
H	-4.100282000	-3.077067000	-0.385135000
H	-0.124497000	2.786192000	-1.615480000
H	-0.857584000	3.092191000	-0.057199000

35

TS1', *E*(SCRF) = -809.689112443 a.u., IF = *i*-1319.3210 cm^{-1}

Zn	-2.129058000	0.141784000	-0.364171000
O	-0.140875000	-0.080921000	-0.868306000
H	-0.465144000	0.967008000	-0.337493000
C	-1.149314000	2.052051000	0.308088000
C	3.809229000	1.201522000	-0.920854000
C	4.807060000	0.530106000	-0.222486000
H	5.247216000	-1.141224000	1.063003000
H	2.931500000	-1.948672000	1.135523000
H	1.717848000	1.262122000	-1.439113000
H	4.052674000	2.085666000	-1.502239000
H	5.832160000	0.885307000	-0.251892000
C	0.729265000	-0.853727000	-0.103450000
C	0.205559000	-1.454537000	1.185163000
H	-0.825541000	-1.243654000	1.466161000
H	0.884008000	-1.528603000	2.029118000
C	0.434081000	-2.323461000	-0.021940000
H	-0.441596000	-2.660244000	-0.568973000
H	1.267994000	-3.017930000	-0.017363000
C	3.165134000	-1.060320000	0.553899000
C	2.154128000	-0.389557000	-0.144463000
C	4.478040000	-0.605837000	0.514769000
C	-3.840213000	-0.861169000	-0.373384000
H	-4.067701000	-1.130657000	-1.411089000

H	-3.679103000	-1.809005000	0.154151000
C	2.493123000	0.745630000	-0.883932000
H	-0.850947000	2.839245000	-0.394452000
H	-2.190534000	2.307561000	0.569377000
C	-0.297785000	2.107439000	1.588597000
H	0.752802000	1.880843000	1.372601000
H	-0.333439000	3.089254000	2.072954000
H	-0.635826000	1.367501000	2.323299000
C	-5.021140000	-0.104588000	0.248292000
H	-5.223313000	0.833727000	-0.278844000
H	-5.944229000	-0.696155000	0.220408000
H	-4.833099000	0.150444000	1.296650000

8

C_2H_6, E(SCRF) = -79.7984908556 a.u.

C	0.000000000	0.000000000	0.763233000
H	-0.509404000	0.883169000	1.159585000
H	1.019549000	-0.000428000	1.159585000
H	-0.510145000	-0.882741000	1.159585000
C	0.000000000	0.000000000	-0.763233000
H	0.510145000	-0.882741000	-1.159585000
H	-1.019549000	-0.000428000	-1.159585000
H	0.509404000	0.883169000	-1.159585000

54

B, E(SCRF) = -1192.00235577 a.u.

Zn	-0.428316000	1.058780000	0.681041000
O	-1.612079000	-0.084667000	1.628595000
C	-2.033752000	-2.013080000	-2.051572000
C	-3.224538000	-1.965679000	-2.767517000
H	-5.265054000	-1.277257000	-2.753918000
H	-5.105945000	-0.220375000	-0.546233000
H	-1.022311000	-1.480910000	-0.223005000
H	-1.169992000	-2.525753000	-2.466031000
H	-3.298617000	-2.430365000	-3.745667000
C	-2.885397000	-0.091926000	1.114523000
C	-4.026015000	-0.243169000	2.092881000
H	-3.732827000	-0.344455000	3.132171000
H	-4.896482000	-0.819344000	1.792148000
C	-3.782072000	1.086199000	1.431461000
H	-3.318079000	1.859526000	2.036429000
H	-4.484138000	1.446855000	0.685327000
C	-4.232653000	-0.718286000	-0.959146000
C	-3.034686000	-0.745925000	-0.231610000
C	-4.326273000	-1.318309000	-2.209326000
C	-0.527377000	2.597772000	-0.602228000

C	-1.942688000	-1.413300000	-0.796467000
C	-1.966785000	2.949715000	-1.003664000
H	-2.496202000	2.076750000	-1.403866000
H	-2.545924000	3.298117000	-0.141379000
H	-2.011206000	3.739703000	-1.765677000
C	4.305180000	0.801358000	-2.089616000
C	2.897317000	1.217104000	-1.658231000
C	2.750357000	1.473466000	-0.147718000
C	4.821627000	-0.488539000	-1.449842000
C	2.506946000	0.206184000	0.635099000
C	4.825745000	-0.444047000	0.081509000
H	4.999668000	1.615230000	-1.837880000
H	2.626709000	2.137333000	-2.185699000
H	3.641872000	1.984587000	0.236537000
H	4.215066000	-1.345663000	-1.771328000
H	5.496012000	-1.210615000	0.480652000
C	1.154822000	-1.199472000	1.973172000
H	0.080576000	-1.341558000	2.105245000
C	3.295046000	-2.084685000	1.115948000
C	1.811896000	-2.375775000	1.263437000
H	1.352255000	-2.500549000	0.276062000
H	1.669786000	-3.306825000	1.818201000
H	3.750602000	-2.748606000	0.371164000
N	3.516605000	-0.701948000	0.678890000
N	1.346718000	0.028592000	1.197236000
H	5.213931000	0.517453000	0.434578000
H	5.843893000	-0.674060000	-1.798490000
H	4.332299000	0.697751000	-3.179644000
H	2.162496000	0.460255000	-1.963913000
H	1.903096000	2.134288000	0.036620000
H	1.607421000	-1.065167000	2.966023000
H	3.819471000	-2.245823000	2.066787000
H	-0.030716000	3.488050000	-0.191716000
H	0.044977000	2.331228000	-1.502913000

27

B', *E*(SCRF) = -729.948570361 a.u.

C	3.498392000	-0.918230000	-0.889130000
C	2.684831000	0.201478000	-0.765974000
C	1.572631000	0.194213000	0.086707000
C	1.322176000	-0.965205000	0.828653000
C	2.134402000	-2.091633000	0.701160000
C	3.223074000	-2.076132000	-0.162558000
H	4.351250000	-0.888474000	-1.560560000
H	2.922726000	1.090977000	-1.342407000

H	0.508402000	-0.966462000	1.548029000
H	1.917986000	-2.977598000	1.290548000
H	3.857027000	-2.950967000	-0.264697000
C	0.665294000	1.383183000	0.232664000
C	0.621949000	2.416218000	-0.869293000
H	-0.352026000	2.866226000	-1.034016000
H	1.225506000	2.286212000	-1.761950000
C	1.297882000	2.734300000	0.437849000
H	0.777350000	3.396299000	1.120948000
H	2.381908000	2.792129000	0.458016000
O	-0.513839000	1.157266000	0.921939000
Zn	-1.689000000	-0.051986000	0.224047000
C	-3.047537000	-1.318252000	-0.415584000
H	-2.647302000	-1.802601000	-1.313120000
H	-3.147228000	-2.108836000	0.336524000
C	-4.418589000	-0.697444000	-0.715681000
H	-4.355103000	0.074159000	-1.489361000
H	-5.129291000	-1.453394000	-1.069653000
H	-4.857537000	-0.231190000	0.171883000

54

TS2, *E*(SCRF) = -1191.96584282 a.u., IF = *i*308.3316 cm^{-1}

Zn	0.462310000	0.369580000	-0.558851000
O	1.516855000	-1.414083000	-1.109105000
C	1.962167000	-1.376999000	0.095000000
C	0.699884000	-0.713943000	1.440987000
C	1.328911000	-2.096380000	1.218672000
C	5.802238000	0.491203000	0.619562000
H	5.499865000	0.244220000	2.738042000
H	3.293073000	-0.812808000	2.494029000
H	3.575916000	-0.471399000	-1.767595000
H	5.819134000	0.594721000	-1.530182000
H	6.775494000	0.958796000	0.732506000
C	3.287551000	-0.702736000	0.334528000
C	4.014688000	-0.305567000	-0.789639000
H	-0.387703000	-0.771855000	1.530198000
H	1.155008000	-0.130873000	2.237628000
C	5.265720000	0.287049000	-0.648265000
H	0.616658000	-2.847537000	0.876033000
H	1.983489000	-2.489667000	1.997739000
C	3.839888000	-0.510515000	1.606170000
C	5.085843000	0.089481000	1.746453000
C	0.881400000	2.288871000	-1.038069000
H	0.402604000	2.539910000	-1.995438000
H	0.389595000	2.930368000	-0.290251000

C	2.366701000	2.667999000	-1.100795000
H	2.885172000	2.120244000	-1.895197000
H	2.520670000	3.739313000	-1.292242000
H	2.886919000	2.424564000	-0.166870000
C	-4.224645000	2.012520000	1.502302000
C	-2.759374000	1.968264000	1.063121000
C	-2.560699000	1.586657000	-0.414426000
C	-4.982142000	0.690959000	1.358121000
C	-2.567524000	0.093967000	-0.635026000
C	-4.973799000	0.137734000	-0.071312000
H	-4.742307000	2.775758000	0.904296000
H	-2.315369000	2.956094000	1.223021000
H	-3.335867000	2.058455000	-1.030999000
H	-4.556255000	-0.068621000	2.027399000
H	-5.782563000	-0.587566000	-0.200079000
C	-1.529863000	-1.931495000	-1.228962000
H	-0.509550000	-2.315660000	-1.249916000
C	-3.788341000	-2.019315000	-0.242676000
C	-2.388806000	-2.606040000	-0.166232000
H	-1.950342000	-2.416581000	0.821036000
H	-2.433269000	-3.688794000	-0.311664000
H	-4.349453000	-2.229376000	0.676496000
N	-3.741262000	-0.564314000	-0.421495000
N	-1.461847000	-0.491973000	-0.982100000
H	-5.163916000	0.940239000	-0.792024000
H	-6.021782000	0.841960000	1.670484000
H	-4.280165000	2.345911000	2.544236000
H	-2.192636000	1.269915000	1.693951000
H	-1.603391000	1.960831000	-0.779117000
H	-1.973092000	-2.099985000	-2.221268000
H	-4.347105000	-2.461587000	-1.077779000

27

TS2', *E*(SCRF) = -729.915683922 a.u., IF = *i*331.6865 cm^{-1}

Zn	-1.546685000	0.255596000	-0.213968000
O	-0.637107000	0.742842000	1.534760000
C	0.293206000	1.205570000	0.746251000
C	-0.373980000	2.076407000	-0.782230000
C	0.417753000	2.629395000	0.417676000
C	3.364791000	-1.606419000	-0.348453000
H	4.189037000	-0.039991000	-1.576239000
H	2.451515000	1.596774000	-0.993103000
H	0.571333000	-1.285766000	1.568848000
H	2.320210000	-2.955353000	0.962863000
H	4.133311000	-2.326128000	-0.611954000

C	1.385596000	0.252847000	0.325187000
C	1.360960000	-1.035049000	0.868027000
H	-1.324980000	2.605010000	-0.894162000
H	0.184654000	1.984248000	-1.709244000
C	2.346886000	-1.959672000	0.531526000
H	-0.167570000	3.271034000	1.071510000
H	1.410239000	3.024239000	0.212229000
C	2.416265000	0.604136000	-0.554308000
C	3.396959000	-0.321270000	-0.889530000
C	-2.895302000	-1.083179000	-0.782362000
H	-3.883653000	-0.610560000	-0.747654000
H	-2.719018000	-1.344063000	-1.831202000
C	-2.884390000	-2.345384000	0.089769000
H	-3.072388000	-2.110157000	1.142280000
H	-3.649782000	-3.065516000	-0.225888000
H	-1.917976000	-2.858665000	0.043368000

54

C, *E*(SCRF) = -1191.99061404 a.u.

N	0.758152000	-0.225610000	1.271528000
H	-1.683934000	-1.655798000	1.667443000
C	-1.912664000	-2.131347000	0.700563000
C	-2.158185000	-0.969589000	-0.239869000
O	-1.360597000	-0.687242000	-1.129897000
C	-5.565267000	1.591142000	0.220115000
H	-6.044599000	0.423147000	1.963695000
H	-4.100672000	-1.076514000	1.718216000
H	-2.873425000	1.058691000	-1.791192000
H	-4.840439000	2.575698000	-1.553302000
H	-6.422321000	2.248498000	0.329795000
C	-3.365248000	-0.103062000	-0.062372000
C	-3.580021000	0.934315000	-0.976814000
C	-4.676261000	1.775784000	-0.838346000
C	-4.259969000	-0.282025000	0.997078000
C	-5.355026000	0.564689000	1.137797000
C	2.200832000	-1.292487000	-1.734785000
H	1.852062000	-0.940433000	-2.716171000
H	2.717772000	-0.428743000	-1.286552000
C	3.212841000	-2.427970000	-1.949061000
H	2.744233000	-3.294100000	-2.429979000
H	4.065585000	-2.134805000	-2.577222000
H	3.627203000	-2.789758000	-0.999055000
Zn	0.603194000	-1.790703000	-0.597022000
C	-0.753329000	-3.025024000	0.262675000
H	-0.407434000	-3.609231000	1.120882000

H	-1.113611000	-3.749185000	-0.478759000
H	-2.867464000	-2.659660000	0.842167000
C	1.797685000	3.154428000	-1.495750000
C	0.753129000	2.044643000	-1.364090000
C	0.263387000	1.803956000	0.072402000
C	3.074631000	2.935212000	-0.682609000
C	1.182688000	0.945997000	0.911306000
C	2.814703000	2.802893000	0.822789000
H	1.344855000	4.104304000	-1.175844000
H	-0.116750000	2.310609000	-1.974814000
H	0.099524000	2.765439000	0.578039000
H	3.597036000	2.032869000	-1.027397000
H	3.726757000	3.027107000	1.383962000
C	1.625396000	-0.966805000	2.181324000
H	1.304124000	-2.013332000	2.185526000
C	3.473946000	0.628713000	1.783603000
C	3.092930000	-0.843992000	1.780394000
H	3.225260000	-1.264411000	0.778245000
H	3.745232000	-1.393539000	2.465240000
H	4.374153000	0.789471000	1.176920000
N	2.402169000	1.465629000	1.237362000
H	2.068570000	3.539697000	1.142774000
H	3.751028000	3.781720000	-0.849451000
H	2.061459000	3.276709000	-2.552044000
H	1.140047000	1.104248000	-1.772389000
H	-0.695196000	1.287029000	0.053889000
H	1.489680000	-0.581737000	3.203378000
H	3.700780000	0.968075000	2.803495000

27

C', *E*(SCRF) = -729.954613865 a.u.

Zn	2.399048000	-0.444714000	0.011789000
O	0.300637000	0.465937000	-0.148297000
C	-0.544803000	-0.421238000	-0.069188000
C	1.343958000	-2.135092000	-0.272890000
C	-0.137515000	-1.882069000	0.020955000
C	-4.658537000	0.754482000	0.061931000
H	-5.108957000	-1.325494000	0.387575000
H	-2.750343000	-2.037244000	0.308579000
H	-1.528729000	2.025480000	-0.335660000
H	-3.913981000	2.748741000	-0.267845000
H	-5.698498000	1.064106000	0.095272000
C	-1.987571000	-0.043284000	-0.027139000
C	-2.325975000	1.305821000	-0.183193000
H	1.696122000	-2.988826000	0.311301000

H	1.457893000	-2.420912000	-1.325710000
C	-3.655993000	1.702208000	-0.142442000
H	-0.391410000	-2.186516000	1.047930000
H	-0.814016000	-2.458859000	-0.624006000
C	-2.997517000	-0.989021000	0.177926000
C	-4.328944000	-0.589020000	0.224829000
C	3.709280000	1.033136000	0.272840000
H	4.104763000	0.987479000	1.295740000
H	4.577048000	0.878989000	-0.380592000
C	3.109761000	2.423437000	0.016492000
H	2.256653000	2.621581000	0.674216000
H	3.838855000	3.228817000	0.175965000
H	2.741215000	2.520426000	-1.010780000

54

TS3, *E*(SCRF) = -1191.9615993 a.u., IF = *i*1205.0135 cm^{-1}

N	0.500955000	-0.002714000	-1.740791000
H	-0.466678000	0.762359000	-1.456523000
C	-1.239202000	1.945666000	-0.976092000
C	-1.725956000	1.176643000	0.099405000
O	-1.022341000	0.959831000	1.143128000
C	-5.440686000	-0.992416000	0.000124000
H	-5.496459000	-0.545034000	-2.105481000
H	-3.374791000	0.709521000	-2.087090000
H	-2.951229000	-0.022471000	2.109271000
H	-5.104218000	-1.285553000	2.105999000
H	-6.375297000	-1.544868000	-0.011059000
C	-3.033252000	0.440963000	0.025947000
C	-3.527121000	-0.142222000	1.197762000
C	-4.726271000	-0.847546000	1.187110000
C	-3.754794000	0.285706000	-1.163264000
C	-4.948792000	-0.427713000	-1.175352000
C	2.393338000	0.974578000	1.794742000
H	2.071220000	0.669313000	2.799087000
H	2.588272000	0.029742000	1.258882000
C	3.704235000	1.766155000	1.891293000
H	3.568418000	2.697851000	2.450980000
H	4.510318000	1.208650000	2.388370000
H	4.079811000	2.053070000	0.900201000
Zn	0.875758000	1.867070000	0.808538000
C	-0.084553000	2.886491000	-0.653747000
H	0.485887000	3.157356000	-1.548060000
H	-0.482495000	3.832491000	-0.257315000
H	-1.965022000	2.228062000	-1.740053000
C	0.868715000	-3.121378000	1.561266000

C	-0.014365000	-1.927652000	1.198704000
C	-0.301688000	-1.787372000	-0.305933000
C	2.278787000	-3.061352000	0.974258000
C	0.786813000	-1.096718000	-1.084997000
C	2.287082000	-2.980281000	-0.553377000
H	0.384211000	-4.046782000	1.217061000
H	-0.980647000	-2.025236000	1.703725000
H	-0.467606000	-2.778239000	-0.752104000
H	2.823885000	-2.199374000	1.378900000
H	3.268821000	-3.265522000	-0.941982000
C	1.526972000	0.592112000	-2.584815000
H	1.220736000	1.613688000	-2.812846000
C	3.201935000	-0.907966000	-1.557634000
C	2.875534000	0.543381000	-1.876769000
H	2.818958000	1.131771000	-0.955365000
H	3.668589000	0.967351000	-2.498369000
H	3.957890000	-0.964357000	-0.764804000
N	2.018875000	-1.645322000	-1.095632000
H	1.566985000	-3.692101000	-0.973966000
H	2.831799000	-3.960285000	1.270243000
H	0.939803000	-3.194637000	2.651653000
H	0.424979000	-0.996267000	1.569890000
H	-1.221072000	-1.223741000	-0.465373000
H	1.577981000	0.034160000	-3.530288000
H	3.603741000	-1.419883000	-2.441599000

81

TS3', *E*(SCRF) = -1653.99417493 a.u., IF = *i*1151.7381 cm^{-1}

N	-2.642055000	-1.125167000	-1.546823000
H	-2.087497000	-0.009826000	-1.738363000
C	-1.084488000	1.042604000	-2.032562000
C	-1.340889000	1.668213000	-0.797053000
O	-0.774680000	1.307093000	0.288554000
C	-4.407346000	4.648798000	-0.254227000
H	-5.070666000	4.138998000	-2.238269000
H	-3.329712000	2.430447000	-2.586264000
H	-1.775992000	3.181027000	1.328497000
H	-3.526452000	4.922198000	1.690551000
H	-5.176888000	5.399679000	-0.102445000
C	-2.416379000	2.712465000	-0.653206000
C	-2.499078000	3.414096000	0.554223000
C	-3.481216000	4.379943000	0.750619000
C	-3.357454000	2.983861000	-1.653438000
C	-4.344906000	3.943372000	-1.454594000
C	0.444556000	-1.969015000	1.095690000

H	0.351922000	-1.640878000	2.143158000
H	-0.442483000	-2.594224000	0.911190000
C	1.703398000	-2.837349000	0.962045000
H	2.606778000	-2.245309000	1.163562000
H	1.734289000	-3.702587000	1.641781000
H	1.814579000	-3.233177000	-0.058337000
Zn	0.519200000	-0.355590000	-0.180292000
C	0.180429000	0.201544000	-2.135084000
H	0.080298000	-0.573636000	-2.905111000
H	1.012236000	0.841558000	-2.472248000
H	-1.467986000	1.560259000	-2.915562000
C	-3.213343000	-1.432086000	2.961672000
C	-2.747365000	-0.405921000	1.929064000
C	-3.523213000	-0.445198000	0.602600000
C	-3.020533000	-2.886823000	2.533430000
C	-3.049786000	-1.498708000	-0.363924000
C	-3.760083000	-3.231326000	1.238741000
H	-4.278871000	-1.267392000	3.180851000
H	-2.862839000	0.601808000	2.341204000
H	-4.594093000	-0.606772000	0.793283000
H	-1.953386000	-3.103530000	2.402521000
H	-3.871584000	-4.315522000	1.143976000
C	-2.266143000	-2.151685000	-2.506157000
H	-1.694022000	-1.670675000	-3.300859000
C	-2.341376000	-3.834360000	-0.704604000
C	-1.468592000	-3.243104000	-1.802305000
H	-0.566387000	-2.794271000	-1.373885000
H	-1.168968000	-4.034165000	-2.495191000
H	-1.724183000	-4.365300000	0.030552000
N	-3.088374000	-2.793550000	0.013514000
H	-4.774523000	-2.815000000	1.261333000
H	-3.393719000	-3.548541000	3.324042000
H	-2.672893000	-1.264276000	3.899713000
H	-1.679954000	-0.527831000	1.720369000
H	-3.445894000	0.514799000	0.090202000
H	-3.177599000	-2.574984000	-2.951733000
H	-3.061131000	-4.551252000	-1.121679000
C	4.503991000	1.993493000	1.802375000
C	3.132513000	2.644345000	1.800442000
C	2.081632000	1.554303000	1.679101000
N	2.358066000	0.660255000	0.560159000
C	3.561559000	0.489734000	0.126767000
N	4.672607000	1.142355000	0.625597000
C	6.060231000	0.709899000	0.435388000

C	6.468631000	0.355055000	-0.994363000
C	6.164248000	-1.097379000	-1.371850000
C	4.794652000	-1.542836000	-0.856720000
C	3.722122000	-0.461713000	-1.045062000
H	4.644655000	1.404342000	2.722536000
H	5.292373000	2.753474000	1.778462000
H	3.056018000	3.326107000	0.946325000
H	2.994938000	3.230295000	2.713917000
H	1.083381000	1.971044000	1.521624000
H	6.303665000	-0.123418000	1.115112000
H	6.668191000	1.558654000	0.762622000
H	7.542878000	0.540756000	-1.097952000
H	5.968997000	1.052542000	-1.675026000
H	6.941905000	-1.755599000	-0.967161000
H	6.202176000	-1.199508000	-2.462559000
H	4.857132000	-1.807441000	0.205992000
H	4.481706000	-2.455733000	-1.372473000
H	2.744023000	-0.922805000	-1.191863000
H	3.920491000	0.118349000	-1.955316000
H	2.040174000	0.955708000	2.600066000

57

TS3'', *E*(SCRF) = -1380.53913331 a.u., IF = i249.5613 cm^{-1}

Zn	-0.152379000	-1.142842000	-0.125781000
C	-0.422078000	0.337074000	-1.856730000
C	1.356251000	-0.785503000	-2.113202000
O	1.139514000	-1.968140000	-2.104171000
C	-1.953071000	0.273598000	-1.706212000
C	-6.589140000	1.028527000	0.599290000
H	-6.946400000	0.728829000	-1.502644000
H	-4.559964000	0.439689000	-2.053210000
H	-3.524530000	0.980440000	2.074990000
H	-5.936410000	1.282385000	2.636314000
H	-7.640546000	1.155052000	0.838200000
C	-3.889477000	0.702439000	-0.016161000
C	-4.287450000	0.936127000	1.304889000
H	-0.256327000	0.375492000	-2.937938000
H	-0.051464000	1.293311000	-1.473448000
C	-5.631672000	1.101139000	1.610730000
H	-2.353895000	-0.700240000	-2.027674000
H	-2.444854000	1.019411000	-2.348720000
C	-4.853981000	0.629243000	-1.026112000
C	-6.200548000	0.790304000	-0.716978000
C	-1.339419000	-2.690797000	0.364730000
H	-2.192157000	-2.745091000	-0.327241000

H	-0.790661000	-3.630451000	0.218478000
C	-1.864467000	-2.618615000	1.805339000
H	-2.451953000	-1.707314000	1.971368000
H	-2.505949000	-3.469523000	2.073158000
H	-1.042590000	-2.595474000	2.532110000
C	-2.436834000	0.512604000	-0.292378000
O	-1.635475000	0.532809000	0.637176000
O	2.147812000	0.092636000	-2.275737000
C	4.442393000	-0.985529000	0.257813000
C	3.864426000	-2.065157000	1.155524000
C	2.379947000	-2.169035000	0.852595000
N	1.727298000	-0.857361000	0.766168000
C	2.416554000	0.248418000	0.672667000
N	3.769834000	0.284429000	0.535724000
C	4.543828000	1.468403000	0.136627000
C	4.223524000	2.756368000	0.894553000
C	3.076881000	3.556913000	0.271429000
C	1.939641000	2.648253000	-0.198360000
C	1.624623000	1.534736000	0.807695000
H	4.311440000	-1.248623000	-0.802023000
H	5.510209000	-0.848055000	0.446039000
H	4.029767000	-1.785611000	2.201542000
H	4.361902000	-3.022272000	0.976404000
H	1.855087000	-2.739244000	1.626570000
H	4.451717000	1.621021000	-0.948650000
H	5.586170000	1.203264000	0.331919000
H	5.128582000	3.371963000	0.920471000
H	4.002118000	2.494614000	1.935216000
H	3.452324000	4.139870000	-0.577213000
H	2.702799000	4.278008000	1.007536000
H	2.175392000	2.195004000	-1.166442000
H	1.034517000	3.244838000	-0.352707000
H	0.574201000	1.250513000	0.743963000
H	1.770138000	1.899218000	1.833914000
H	2.228387000	-2.700715000	-0.094739000

30

TS3''', *E*(SCRF) = -918.491767225 a.u., IF = *i*286.2201 cm^{-1}

Zn	1.876473000	0.493899000	-0.144527000
C	1.262780000	-1.359862000	1.035628000
C	2.797970000	-1.759480000	-0.411238000
O	2.831254000	-0.944044000	-1.321330000
C	-0.076887000	-0.644599000	1.364489000
C	-5.056949000	-0.188228000	-0.285726000
H	-5.130802000	-0.712918000	1.800989000

H	-2.684418000	-0.733369000	2.071645000
H	-2.205536000	0.339037000	-2.061113000
H	-4.680633000	0.343305000	-2.339626000
H	-6.136295000	-0.183355000	-0.401157000
C	-2.285747000	-0.207476000	0.011049000
C	-2.860046000	0.105066000	-1.228488000
H	1.853635000	-1.443408000	1.950539000
H	0.989141000	-2.377272000	0.750560000
C	-4.239449000	0.108196000	-1.376774000
H	0.090612000	0.266053000	1.962814000
H	-0.716238000	-1.294055000	1.970020000
C	-3.111149000	-0.504419000	1.101439000
C	-4.493696000	-0.488897000	0.952026000
C	2.308581000	2.385911000	0.316995000
H	2.943900000	2.418173000	1.209172000
H	2.905185000	2.821687000	-0.492387000
C	1.047660000	3.232258000	0.543424000
H	0.443041000	2.848752000	1.375096000
H	1.283835000	4.277519000	0.780314000
H	0.401095000	3.238447000	-0.340921000
C	-0.807715000	-0.186804000	0.120219000
O	-0.140006000	0.244007000	-0.824707000
O	3.238398000	-2.746910000	0.051236000

3

CO_2, *E*(SCRF) = -188.57623298 a.u.

C	0.000000000	0.000000000	0.000000000
O	0.000000000	0.000000000	1.162703000
O	0.000000000	0.000000000	-1.162703000

54

D, *E*(SCRF) = -1191.97831229 a.u.

N	0.431696000	-0.032493000	1.640968000
H	-0.464153000	-0.486546000	1.377481000
C	-1.508073000	-2.245016000	0.631996000
C	-1.779542000	-1.169877000	-0.169864000
O	-0.925282000	-0.725892000	-1.076809000
C	-5.387146000	1.176329000	0.049515000
H	-5.652225000	0.339080000	2.016082000
H	-3.591673000	-1.009466000	1.929183000
H	-2.761042000	0.462342000	-1.998903000
H	-4.840965000	1.841503000	-1.922694000
H	-6.291654000	1.775380000	0.093025000
C	-3.047965000	-0.381446000	-0.060072000
C	-3.410215000	0.446903000	-1.129589000
C	-4.571154000	1.213861000	-1.078045000

C	-3.870472000	-0.400295000	1.074559000
C	-5.028956000	0.366604000	1.126923000
C	2.451779000	-0.979411000	-1.679527000
H	2.227413000	-0.609368000	-2.689394000
H	2.676919000	-0.070508000	-1.093737000
C	3.709576000	-1.857099000	-1.728595000
H	3.546483000	-2.749346000	-2.342528000
H	4.588128000	-1.340779000	-2.141246000
H	3.993705000	-2.220846000	-0.731707000
Zn	0.786334000	-1.817856000	-0.870197000
C	-0.256422000	-3.050437000	0.384639000
H	0.191691000	-3.421374000	1.316076000
H	-0.516622000	-3.963007000	-0.176447000
H	-2.277187000	-2.596047000	1.319200000
C	1.155414000	3.194578000	-1.477174000
C	0.179037000	2.047105000	-1.224797000
C	-0.213590000	1.857582000	0.250926000
C	2.518993000	3.017300000	-0.809098000
C	0.797922000	1.089185000	1.052163000
C	2.425089000	2.889780000	0.711789000
H	0.711159000	4.136381000	-1.123439000
H	-0.749827000	2.231011000	-1.772915000
H	-0.360293000	2.831770000	0.738715000
H	3.029305000	2.132948000	-1.210219000
H	3.393571000	3.095574000	1.174657000
C	1.331611000	-0.793323000	2.499126000
H	0.959806000	-1.817290000	2.526547000
C	3.132143000	0.743599000	1.751428000
C	2.737969000	-0.715713000	1.923207000
H	2.758351000	-1.227706000	0.956659000
H	3.454988000	-1.208050000	2.584575000
H	3.981072000	0.827314000	1.064023000
N	2.039997000	1.552018000	1.185563000
H	1.720170000	3.625967000	1.114650000
H	3.149445000	3.884884000	-1.034193000
H	1.301634000	3.306273000	-2.556524000
H	0.570733000	1.100921000	-1.609200000
H	-1.159348000	1.318441000	0.307409000
H	1.299592000	-0.375084000	3.512998000
H	3.425233000	1.190531000	2.709464000

69

D', *E*(SCRF) = -1577.60753017 a.u.

Zn	1.928182000	0.861157000	1.491543000
O	1.128482000	0.257077000	-0.431655000

C	0.626455000	0.842772000	-1.551978000
C	0.807877000	-0.176528000	2.832290000
H	-0.097560000	-0.742809000	-0.059105000
N	-0.970464000	-1.289484000	-0.299563000
C	-2.127699000	-0.768107000	0.032176000
H	-1.771718000	0.961230000	-2.802290000
H	0.564815000	2.873239000	0.237334000
H	-1.308810000	4.423076000	0.711166000
H	-3.429768000	4.247920000	-0.572052000
C	-0.479347000	1.810917000	-1.314462000
C	-0.367157000	2.790342000	-0.318764000
C	-1.418701000	3.662681000	-0.056136000
C	-1.676668000	1.727268000	-2.036008000
C	-2.733208000	2.598827000	-1.773673000
C	3.371566000	-1.998672000	0.487159000
H	4.325778000	-2.507919000	0.305936000
H	3.581682000	-1.279566000	1.295675000
C	2.336101000	-3.020905000	0.983580000
H	2.149631000	-3.798632000	0.230807000
H	2.643886000	-3.539000000	1.902438000
H	1.370951000	-2.543162000	1.203262000
Zn	2.724894000	-1.027560000	-1.152380000
C	1.053923000	0.515987000	-2.788657000
C	2.157956000	-0.481287000	-3.011130000
C	3.386233000	2.151276000	0.961272000
H	2.987532000	3.176146000	0.947586000
H	4.183967000	2.164891000	1.714671000
C	1.670104000	-0.851173000	3.909801000
H	1.078930000	-1.407337000	4.651073000
H	2.377777000	-1.561040000	3.465552000
H	2.266412000	-0.115485000	4.462088000
C	3.989640000	1.834616000	-0.414355000
H	3.223524000	1.818140000	-1.201361000
H	4.756150000	2.556444000	-0.729028000
H	4.479525000	0.848903000	-0.414434000
H	1.803345000	-1.313825000	-3.637065000
H	2.974407000	-0.019182000	-3.581781000
H	0.596400000	1.045064000	-3.626754000
C	-4.380762000	-0.030307000	2.315299000
C	-2.858640000	-0.168601000	2.373176000
C	-2.141961000	0.294225000	1.092372000
C	-5.060822000	-0.859412000	1.224383000
H	-3.652612000	2.522859000	-2.348155000
C	-4.552357000	-0.548665000	-0.184981000

H	-4.627811000	1.027798000	2.152524000
H	-2.477336000	0.439413000	3.198847000
H	-2.608064000	1.201936000	0.688219000
H	-4.926623000	-1.932065000	1.414599000
H	-5.263058000	-0.908649000	-0.932832000
C	-0.814365000	-2.279582000	-1.355479000
H	0.082207000	-2.864588000	-1.130717000
C	-3.295298000	-2.269755000	-1.502985000
C	-2.059122000	-3.152429000	-1.389087000
H	-2.113435000	-3.743945000	-0.469370000
H	-2.024360000	-3.843517000	-2.234264000
H	-4.202207000	-2.858501000	-1.335322000
N	-3.274072000	-1.197456000	-0.501069000
C	-2.609672000	3.566932000	-0.778007000
H	-4.451999000	0.532933000	-0.336986000
H	-6.138632000	-0.664175000	1.248644000
H	-4.805473000	-0.303183000	3.287031000
H	-2.570232000	-1.203014000	2.598511000
H	-1.102397000	0.542358000	1.314059000
H	-0.647027000	-1.758295000	-2.307516000
H	-3.364237000	-1.820919000	-2.502207000
H	0.085786000	0.493568000	3.325553000
H	0.197828000	-0.945586000	2.333591000

96

D'', *E*(SCRF) = -2039.63161638 a.u.

Zn	0.252652000	1.437789000	-1.320333000
O	0.127623000	0.061041000	0.193929000
C	0.728523000	-0.352601000	1.321150000
C	0.926690000	0.626123000	-3.113617000
H	1.914022000	-0.379132000	-1.607342000
N	2.414158000	-1.154591000	-1.153114000
C	3.475866000	-0.878010000	-0.433007000
H	2.846171000	-0.987513000	2.955475000
H	0.952382000	2.321029000	0.990001000
H	2.678331000	3.838821000	1.872556000
H	4.494026000	2.960699000	3.334554000
C	1.767039000	0.550065000	1.896977000
C	1.750177000	1.921707000	1.607099000
C	2.723241000	2.780841000	2.114840000
C	2.794856000	0.073967000	2.727568000
C	3.757745000	0.932956000	3.248684000
C	-2.200892000	-1.144450000	-2.045719000
H	-1.380800000	-1.339768000	-2.752121000
H	-2.934081000	-1.941440000	-2.245089000

C	-2.842466000	0.201484000	-2.413517000
H	-2.129505000	1.037047000	-2.333279000
H	-3.238730000	0.237437000	-3.439169000
H	-3.675885000	0.457009000	-1.741539000
Zn	-1.543333000	-1.308571000	-0.112080000
C	0.388520000	-1.520962000	1.925405000
C	-0.684789000	-2.430978000	1.387054000
C	-0.305028000	3.351814000	-0.897555000
H	-1.165427000	3.619182000	-1.528332000
H	-0.655257000	3.469143000	0.138065000
C	2.260190000	1.081882000	-3.728443000
H	3.121897000	0.573299000	-3.271787000
H	2.327348000	0.882475000	-4.806259000
H	2.422843000	2.157623000	-3.589706000
C	0.831161000	4.352214000	-1.163802000
H	1.161838000	4.310093000	-2.208972000
H	0.553582000	5.395840000	-0.958839000
H	1.715565000	4.133190000	-0.548655000
H	-0.254474000	-3.410458000	1.116092000
H	-1.403204000	-2.653422000	2.188692000
H	0.869519000	-1.739866000	2.881101000
C	6.597194000	0.096720000	-0.451621000
C	5.401114000	0.498468000	-1.315784000
C	4.065095000	0.499732000	-0.551025000
C	6.515045000	-1.311306000	0.141368000
H	4.535400000	0.536905000	3.897182000
C	5.304470000	-1.522910000	1.054783000
H	6.687815000	0.816946000	0.373114000
H	5.565388000	1.508800000	-1.702402000
H	4.190565000	0.925731000	0.452404000
H	6.495913000	-2.066326000	-0.654735000
H	5.469604000	-2.383921000	1.705750000
C	1.710990000	-2.431541000	-1.156793000
H	1.329877000	-2.593776000	-2.168285000
C	3.391760000	-3.094971000	0.538428000
C	2.670059000	-3.529910000	-0.730129000
H	3.403846000	-3.728335000	-1.519050000
H	2.114816000	-4.451267000	-0.539485000
H	4.155776000	-3.822630000	0.820382000
N	4.061323000	-1.803412000	0.327466000
C	3.737051000	2.293021000	2.935153000
H	5.157130000	-0.654735000	1.706814000
H	7.418547000	-1.495338000	0.733193000
H	7.513711000	0.182767000	-1.044827000

H	5.321148000	-0.160641000	-2.189772000
H	3.332618000	1.126622000	-1.069078000
H	0.860092000	-2.357656000	-0.473567000
H	2.673601000	-2.985542000	1.362096000
H	0.116121000	1.047668000	-3.728095000
H	0.810299000	-0.458988000	-3.253731000
C	-4.860374000	2.174660000	0.870104000
C	-3.568101000	2.342882000	1.647736000
C	-2.530616000	1.413418000	1.043401000
N	-3.019341000	0.046135000	0.932414000
C	-4.283067000	-0.191969000	0.834061000
N	-5.275373000	0.771441000	0.860690000
C	-6.599608000	0.597607000	0.253676000
C	-7.364815000	-0.669415000	0.634903000
C	-7.021476000	-1.883184000	-0.234770000
C	-5.518635000	-1.996882000	-0.504106000
C	-4.680693000	-1.652002000	0.735150000
H	-4.729497000	2.538656000	-0.161700000
H	-5.670036000	2.756299000	1.324394000
H	-3.738337000	2.085416000	2.699200000
H	-3.234119000	3.383671000	1.600969000
H	-1.607936000	1.392767000	1.631240000
H	-6.530626000	0.681285000	-0.844087000
H	-7.180049000	1.461476000	0.591911000
H	-8.435709000	-0.456424000	0.549480000
H	-7.177927000	-0.876233000	1.694073000
H	-7.560686000	-1.818018000	-1.186904000
H	-7.378949000	-2.790578000	0.266102000
H	-5.224582000	-1.335515000	-1.328382000
H	-5.276224000	-3.010928000	-0.836064000
H	-3.746952000	-2.217821000	0.738838000
H	-5.215964000	-1.940758000	1.648396000
H	-2.248129000	1.766419000	0.042025000

69

TS4, E(SCRF) = -1577.58526588 a.u., IF = i1092.6222 cm^{-1}

Zn	-1.230215000	1.526412000	-0.190108000
O	-1.570095000	-0.082584000	0.881133000
C	-0.728891000	-0.866053000	1.598271000
C	-1.341876000	1.025410000	-2.350918000
H	-0.401343000	0.156508000	-1.620291000
N	0.420591000	-0.765200000	-1.396446000
C	1.630942000	-0.578202000	-0.958523000
H	1.712689000	-1.955820000	2.159903000
H	-0.494049000	1.715613000	2.372394000

H	1.475829000	2.852167000	3.318783000
H	3.574150000	1.580842000	3.738168000
C	0.465569000	-0.201046000	2.182522000
C	0.425848000	1.159002000	2.518720000
C	1.536680000	1.797441000	3.067135000
C	1.654830000	-0.903841000	2.426322000
C	2.756917000	-0.272435000	2.994440000
C	-4.425044000	-0.196889000	-0.858063000
H	-4.043394000	-0.120479000	-1.885205000
H	-5.406899000	-0.677221000	-0.950089000
C	-4.620309000	1.214074000	-0.283036000
H	-3.671595000	1.760873000	-0.158744000
H	-5.255674000	1.851244000	-0.913113000
H	-5.074394000	1.186198000	0.713568000
Zn	-3.189979000	-1.343619000	0.236153000
C	-1.010784000	-2.171104000	1.808953000
C	-2.256677000	-2.814622000	1.262369000
C	-0.917852000	3.474401000	0.214686000
H	-1.545050000	4.059836000	-0.471163000
H	-1.293973000	3.698175000	1.221497000
C	-0.404169000	1.856578000	-3.232537000
H	0.537287000	1.318705000	-3.411318000
H	-0.830011000	2.096406000	-4.213499000
H	-0.146489000	2.807239000	-2.749213000
C	0.534530000	3.960239000	0.099439000
H	0.920869000	3.843529000	-0.921709000
H	0.644223000	5.021913000	0.357580000
H	1.206428000	3.396898000	0.759230000
H	-2.009151000	-3.709341000	0.672024000
H	-2.875451000	-3.186491000	2.090869000
H	-0.339457000	-2.730572000	2.461233000
C	4.618421000	0.784676000	-1.365247000
C	3.217371000	1.236892000	-1.777985000
C	2.105061000	0.843118000	-0.790217000
C	4.786080000	-0.729317000	-1.231193000
H	3.662036000	-0.842490000	3.187232000
C	3.842491000	-1.344127000	-0.194025000
H	4.864223000	1.248467000	-0.399933000
H	3.211020000	2.328340000	-1.865443000
H	2.425575000	1.002098000	0.249447000
H	4.625487000	-1.224681000	-2.197292000
H	4.220203000	-2.316087000	0.133603000
C	-0.132259000	-2.095633000	-1.590632000
H	-0.863537000	-2.036323000	-2.403377000

C	2.051661000	-2.975986000	-0.840633000
C	0.975595000	-3.087875000	-1.911009000
H	1.409850000	-2.861371000	-2.891018000
H	0.585336000	-4.108329000	-1.938621000
H	2.926725000	-3.574848000	-1.108819000
N	2.488105000	-1.585464000	-0.695093000
C	2.710049000	1.086978000	3.305129000
H	3.796403000	-0.709947000	0.697767000
H	5.816944000	-0.944914000	-0.928382000
H	5.345522000	1.166888000	-2.089756000
H	2.968890000	0.842639000	-2.771963000
H	1.242436000	1.495646000	-0.954619000
H	-0.661670000	-2.392898000	-0.677781000
H	1.667169000	-3.340130000	0.122367000
H	-2.278409000	1.587216000	-2.190584000
H	-1.688159000	0.135951000	-2.897179000

96

TS4', *E*(SCRF) = -918.491767225 a.u., IF = *i*286.2201 cm^{-1}

Zn	1.038253000	1.652966000	1.168504000
O	-0.067867000	0.394034000	0.161925000
C	0.064269000	-0.896527000	-0.208652000
C	1.959824000	3.212288000	-0.109213000
H	2.158016000	1.856561000	-0.626681000
N	2.478658000	0.911970000	-1.398168000
C	3.244626000	-0.084450000	-1.070338000
H	1.505348000	-3.123792000	-0.879996000
H	0.463611000	-0.635852000	2.455286000
H	1.799873000	-2.067139000	3.947057000
H	2.985102000	-4.060666000	3.043118000
C	0.884911000	-1.769048000	0.673718000
C	0.988767000	-1.492404000	2.044251000
C	1.738483000	-2.308074000	2.889681000
C	1.555917000	-2.898840000	0.182041000
C	2.294351000	-3.721071000	1.027264000
C	-1.654204000	3.198738000	-0.713391000
H	-0.727985000	3.641497000	-1.105518000
H	-2.465849000	3.723574000	-1.235748000
C	-1.771970000	3.505976000	0.786497000
H	-0.989780000	3.007645000	1.387536000
H	-1.701816000	4.575835000	1.032656000
H	-2.721322000	3.134927000	1.191117000
Zn	-1.723169000	1.195596000	-1.040841000
C	-0.600844000	-1.386581000	-1.283355000
C	-1.510079000	-0.539524000	-2.133308000

C	1.468588000	1.832284000	3.131399000
H	1.425704000	2.900600000	3.383414000
H	0.677190000	1.358330000	3.728319000
C	3.369810000	3.623565000	0.325668000
H	4.128349000	3.056389000	-0.231996000
H	3.579280000	4.687151000	0.163304000
H	3.531864000	3.420143000	1.391444000
C	2.831562000	1.270640000	3.563264000
H	3.658960000	1.785127000	3.057460000
H	3.009465000	1.374310000	4.642259000
H	2.928194000	0.204937000	3.321268000
H	-1.089395000	-0.423612000	-3.145731000
H	-2.453325000	-1.083581000	-2.287075000
H	-0.513516000	-2.460691000	-1.462321000
C	6.267326000	-1.077676000	-0.211208000
C	5.489111000	0.203049000	0.092849000
C	3.972299000	-0.002408000	0.248651000
C	5.850963000	-1.788688000	-1.499546000
H	2.801168000	-4.592181000	0.620122000
C	4.382300000	-2.224492000	-1.500363000
H	6.133520000	-1.774315000	0.627990000
H	5.867031000	0.632147000	1.026639000
H	3.755922000	-0.901483000	0.843075000
H	6.032851000	-1.148593000	-2.372180000
H	4.227866000	-3.021956000	-2.231461000
C	1.711335000	0.935362000	-2.632770000
H	1.643240000	1.977624000	-2.960753000
C	2.664603000	-1.302696000	-3.092772000
C	2.366368000	0.064192000	-3.693671000
H	3.301079000	0.523344000	-4.034859000
H	1.705710000	-0.047354000	-4.557476000
H	3.243479000	-1.913874000	-3.790382000
N	3.447798000	-1.158796000	-1.862880000
C	2.399775000	-3.424906000	2.386373000
H	4.113259000	-2.641393000	-0.523295000
H	6.473071000	-2.682537000	-1.622942000
H	7.336698000	-0.844327000	-0.256021000
H	5.667515000	0.951382000	-0.690334000
H	3.554160000	0.847276000	0.797906000
H	0.695093000	0.590619000	-2.413437000
H	1.723474000	-1.822318000	-2.864652000
H	1.216425000	3.792574000	0.463520000
H	1.771472000	3.534649000	-1.142896000
C	-4.194477000	-1.850449000	1.960069000

C	-2.732655000	-1.448196000	2.041181000
C	-2.658290000	0.057191000	1.840029000
N	-3.219720000	0.426568000	0.546833000
C	-4.275629000	-0.196938000	0.137256000
N	-4.845285000	-1.271389000	0.781249000
C	-6.219060000	-1.743782000	0.595681000
C	-6.682593000	-1.911189000	-0.852000000
C	-7.234685000	-0.621716000	-1.465407000
C	-6.386303000	0.597490000	-1.098003000
C	-4.880512000	0.304317000	-1.162703000
H	-4.726109000	-1.532067000	2.869758000
H	-4.293476000	-2.940274000	1.890504000
H	-2.155014000	-1.938409000	1.248716000
H	-2.317095000	-1.754686000	3.005958000
H	-1.627206000	0.413714000	1.876306000
H	-6.925896000	-1.103094000	1.147042000
H	-6.252098000	-2.723383000	1.081362000
H	-7.455997000	-2.686163000	-0.877980000
H	-5.839962000	-2.295410000	-1.436946000
H	-8.266564000	-0.464971000	-1.129893000
H	-7.271768000	-0.731425000	-2.555358000
H	-6.642255000	0.948598000	-0.090651000
H	-6.621975000	1.425094000	-1.773658000
H	-4.336470000	1.222251000	-1.395706000
H	-4.664141000	-0.404766000	-1.970733000
H	-3.218051000	0.573790000	2.633960000

61

E, *E*(SCRF) = -1497.85004316 a.u.

Zn	-0.943913000	1.335919000	0.148678000
O	-1.717423000	-0.278038000	0.933213000
C	-0.962529000	-1.379200000	1.180720000
N	0.305346000	0.279604000	-1.169345000
C	1.566208000	0.088817000	-0.908043000
H	1.371384000	-2.804671000	1.091008000
H	-0.462701000	0.617945000	2.906441000
H	1.625428000	1.078563000	4.157609000
H	3.580668000	-0.438670000	3.921345000
C	0.301525000	-1.132382000	1.921669000
C	0.403065000	-0.026659000	2.778339000
C	1.574758000	0.223210000	3.490542000
C	1.420624000	-1.966599000	1.780831000
C	2.585447000	-1.725046000	2.502855000
C	-3.984294000	0.812706000	-1.077609000
H	-3.165617000	1.311198000	-1.627792000

H	-4.779461000	0.687822000	-1.821847000
C	-4.467806000	1.752184000	0.039421000
H	-3.702039000	1.896806000	0.812662000
H	-4.739932000	2.749559000	-0.331012000
H	-5.349022000	1.343890000	0.546841000
Zn	-3.306416000	-0.945653000	-0.367397000
C	-1.378197000	-2.606217000	0.801883000
C	-2.665855000	-2.812779000	0.048247000
C	-1.097559000	3.319606000	0.316023000
H	-1.840911000	3.635418000	-0.427707000
H	-1.525392000	3.582458000	1.290244000
C	0.210114000	4.090919000	0.094153000
H	0.690849000	3.814801000	-0.853416000
H	0.057369000	5.177633000	0.072131000
H	0.937381000	3.890830000	0.891373000
H	-2.494379000	-3.444592000	-0.834253000
H	-3.379595000	-3.372737000	0.668368000
H	-0.772607000	-3.460961000	1.104620000
C	4.711928000	1.175278000	-0.792466000
C	3.356574000	1.879720000	-0.709409000
C	2.263725000	1.065100000	0.006336000
C	4.701319000	-0.137701000	-1.575754000
H	3.434975000	-2.393684000	2.391588000
C	3.695753000	-1.152341000	-1.026026000
H	5.057480000	0.967682000	0.229924000
H	3.484564000	2.820422000	-0.163866000
H	2.676882000	0.537371000	0.875233000
H	4.470354000	0.046540000	-2.633106000
H	3.941292000	-2.157412000	-1.380359000
C	-0.372341000	-0.653581000	-2.067991000
H	-1.228537000	-0.136745000	-2.514035000
C	1.775747000	-1.797285000	-2.467809000
C	0.575529000	-1.158903000	-3.145928000
H	0.905386000	-0.319909000	-3.768668000
H	0.080376000	-1.888253000	-3.792033000
H	2.571034000	-1.996723000	-3.194644000
N	2.317792000	-0.901418000	-1.446368000
C	2.670253000	-0.626803000	3.360946000
H	3.743790000	-1.187700000	0.068631000
H	5.702319000	-0.582509000	-1.539951000
H	5.445752000	1.855964000	-1.237536000
H	3.004271000	2.150030000	-1.713465000
H	1.504386000	1.749809000	0.391280000
H	-0.757046000	-1.500001000	-1.479913000

H	1.494647000	-2.753395000	-2.005431000

64

TS5, *E*(SCRF) = -1686.41458292 a.u., IF = *i*209.4395 cm^{-1}

C	-1.377659000	2.196854000	1.736400000
C	-2.201181000	2.209096000	-0.317203000
C	-1.085518000	1.467213000	-0.658787000
O	-1.519692000	1.068029000	2.129200000
O	-1.086545000	3.323289000	1.933602000
H	2.074420000	4.913194000	-0.911744000
H	-0.083005000	4.000413000	-0.153901000
H	0.803348000	0.268742000	-2.066012000
H	2.982015000	1.193340000	-2.856094000
H	3.623731000	3.518383000	-2.262923000
C	0.215720000	2.065695000	-1.066212000
C	1.101725000	1.282636000	-1.816414000
H	-2.150392000	3.285788000	-0.461026000
C	2.316986000	1.803186000	-2.250536000
C	0.579142000	3.383373000	-0.751767000
C	1.803287000	3.894536000	-1.171215000
C	-1.885996000	-2.308196000	1.920373000
H	-1.509640000	-3.277482000	1.568431000
H	-1.683010000	-2.288065000	2.999417000
C	-3.402438000	-2.231977000	1.693019000
H	-3.657869000	-2.379667000	0.635807000
H	-3.955015000	-2.994724000	2.257802000
H	-3.806824000	-1.256523000	1.992102000
Zn	-0.852658000	-0.791334000	1.117350000
O	-1.160523000	0.149026000	-0.669102000
C	2.675979000	3.110312000	-1.924854000
C	-3.542448000	1.536699000	-0.169987000
H	-4.334150000	2.209024000	-0.509542000
H	-3.766352000	1.300877000	0.879502000
Zn	-3.349666000	-0.245800000	-1.113458000
C	-3.369792000	-1.974034000	-2.117829000
H	-4.224531000	-2.595662000	-1.824767000
H	-3.502163000	-1.772639000	-3.188804000
C	-2.062932000	-2.752626000	-1.903512000
H	-1.931831000	-3.028560000	-0.848537000
H	-2.019781000	-3.683222000	-2.486096000
H	-1.190976000	-2.148186000	-2.183481000
N	1.168029000	-0.278735000	1.230826000
C	2.021402000	-0.803285000	0.398722000
C	3.797720000	-3.397271000	-0.713105000
C	2.471617000	-3.279646000	0.040467000

C	1.623953000	-2.054034000	-0.351181000
C	4.764573000	-2.233243000	-0.495617000
C	4.151583000	-0.875785000	-0.847008000
H	3.580140000	-3.473601000	-1.787558000
H	1.877319000	-4.175986000	-0.162053000
H	1.682380000	-1.882574000	-1.433207000
H	5.102482000	-2.201872000	0.548110000
H	4.937165000	-0.129903000	-0.995363000
C	1.560216000	0.899470000	2.004589000
H	0.991789000	0.894561000	2.938125000
C	3.788449000	0.802585000	0.950922000
C	3.056235000	0.915056000	2.276204000
H	3.323768000	0.068671000	2.918697000
H	3.346966000	1.835882000	2.788199000
H	4.860098000	0.644025000	1.112299000
N	3.277650000	-0.340496000	0.195104000
H	3.606892000	-0.939360000	-1.796696000
H	5.654231000	-2.388001000	-1.116486000
H	4.289939000	-4.333742000	-0.429686000
H	2.647454000	-3.260494000	1.123524000
H	0.573179000	-2.260869000	-0.135348000
H	1.276689000	1.806878000	1.451374000
H	3.661843000	1.717570000	0.355271000

37

TS5', E(SCRF) = -1224.34695236 a.u., IF = i276.8301 cm^{-1}

Zn	-0.235519000	1.257672000	-0.353946000
C	0.278651000	-2.310402000	0.132276000
C	0.109148000	-1.412188000	-1.745264000
O	-0.626169000	-0.436852000	-1.683708000
C	4.699986000	0.390890000	0.616829000
H	5.110220000	-1.139379000	-0.842087000
H	2.805292000	-2.004910000	-0.866972000
H	1.670946000	0.932705000	2.058886000
H	3.990144000	1.816701000	2.065870000
H	5.715495000	0.773851000	0.617108000
C	2.083864000	-0.599978000	0.609863000
C	2.429254000	0.489759000	1.420383000
H	1.077404000	-3.014243000	-0.092791000
C	3.732109000	0.978669000	1.426516000
C	3.063983000	-1.185186000	-0.204182000
C	4.361524000	-0.688076000	-0.199233000
C	0.164563000	3.080343000	-1.011499000
H	1.217257000	3.290573000	-0.791829000
H	0.071807000	3.069873000	-2.102737000

C	-0.730988000	4.170910000	-0.412285000
H	-0.636988000	4.221358000	0.677932000
H	-0.476109000	5.162243000	-0.806063000
H	-1.788692000	3.996091000	-0.636018000
C	0.671585000	-1.073223000	0.618722000
O	-0.235342000	-0.188200000	0.983179000
O	0.718319000	-2.131021000	-2.459934000
C	-1.121853000	-2.832253000	0.333266000
H	-1.126322000	-3.462931000	1.232704000
H	-1.399769000	-3.490822000	-0.496373000
Zn	-2.298861000	-1.236459000	0.582369000
C	-3.641616000	0.230535000	0.717212000
H	-4.498489000	0.012124000	0.069972000
H	-4.040951000	0.274919000	1.737316000
C	-3.064613000	1.601470000	0.343904000
H	-2.690441000	1.616802000	-0.693060000
H	-3.796595000	2.417705000	0.404673000
H	-2.240851000	1.891942000	1.015463000

91

TS5'', *E*(SCRF) = -2148.44725433 a.u., IF = *i*127.5458 cm^{-1}

C	0.778140000	1.130542000	-3.221906000
C	-1.035947000	1.398142000	-1.986014000
C	-0.397705000	0.462142000	-1.184028000
O	1.658130000	1.743192000	-2.668156000
O	0.404073000	0.549670000	-4.183380000
H	-1.584810000	-3.448893000	-3.474421000
H	-1.136669000	-1.029225000	-3.410250000
H	-0.108768000	-1.312372000	0.738599000
H	-0.604577000	-3.758582000	0.696052000
H	-1.334079000	-4.832715000	-1.425417000
C	-0.604239000	-1.001065000	-1.316286000
C	-0.440152000	-1.798017000	-0.174333000
H	-1.731286000	1.036141000	-2.739798000
C	-0.713592000	-3.162986000	-0.207409000
C	-1.027363000	-1.618313000	-2.506216000
C	-1.271349000	-2.988191000	-2.542530000
C	3.139207000	3.256454000	-0.012226000
H	3.475788000	3.211803000	1.031155000
H	4.055496000	3.312771000	-0.614958000
C	2.305986000	4.526443000	-0.237385000
H	1.389057000	4.523177000	0.365713000
H	2.855222000	5.443478000	0.015655000
H	1.995461000	4.615335000	-1.285432000
Zn	2.166272000	1.589516000	-0.545286000

O	0.294199000	0.901192000	-0.147749000
C	-1.126005000	-3.767631000	-1.394661000
C	-1.091619000	2.806155000	-1.544669000
H	-1.793282000	3.395845000	-2.135633000
H	-0.106410000	3.292182000	-1.605817000
Zn	-1.131586000	2.653517000	0.537906000
C	-0.595744000	3.579618000	2.267554000
H	-0.488462000	4.655335000	2.073349000
H	-1.414358000	3.502820000	2.998782000
C	0.705087000	3.076759000	2.913839000
H	1.554886000	3.175962000	2.228125000
H	0.970401000	3.622544000	3.830924000
H	0.647082000	2.014712000	3.187898000
N	2.832788000	-0.412549000	-0.732949000
C	2.708001000	-1.199139000	0.293932000
C	3.635341000	-2.307137000	3.309498000
C	3.810643000	-0.973747000	2.580756000
C	2.630459000	-0.580692000	1.671998000
C	3.509557000	-3.523827000	2.393414000
C	2.369545000	-3.390862000	1.381602000
H	2.732645000	-2.245204000	3.933578000
H	3.932749000	-0.184327000	3.328903000
H	1.677645000	-0.842995000	2.151166000
H	4.444111000	-3.689931000	1.842682000
H	2.104387000	-4.372148000	0.977960000
C	2.941583000	-1.000360000	-2.067295000
H	3.493339000	-0.300847000	-2.700404000
C	2.855162000	-3.244053000	-1.050552000
C	3.619018000	-2.361273000	-2.022206000
H	4.654789000	-2.248006000	-1.683097000
H	3.633737000	-2.821532000	-3.013554000
H	3.398026000	-4.177253000	-0.864435000
N	2.698694000	-2.555705000	0.229853000
H	1.466392000	-3.009963000	1.876768000
H	3.334158000	-4.415963000	3.005531000
H	4.476366000	-2.455728000	3.995347000
H	4.732012000	-0.983356000	1.984840000
H	2.619930000	0.505258000	1.553271000
H	1.936488000	-1.108442000	-2.500994000
H	1.863555000	-3.499030000	-1.452059000
C	-3.518404000	-0.978740000	2.922402000
C	-1.998354000	-0.950642000	3.009052000
C	-1.561485000	0.398707000	2.449204000
N	-2.309587000	0.844306000	1.273908000

C	-3.307043000	0.163290000	0.821280000
N	-3.914922000	-0.862793000	1.523220000
C	-4.314893000	-2.140219000	0.916507000
C	-4.296622000	-2.185449000	-0.603600000
C	-5.362023000	-1.314160000	-1.268629000
C	-5.360314000	0.117593000	-0.719408000
C	-3.939518000	0.625288000	-0.481246000
H	-3.939606000	-0.134985000	3.482697000
H	-3.950749000	-1.897141000	3.326572000
H	-1.602145000	-1.779309000	2.408077000
H	-1.642450000	-1.073912000	4.036500000
H	-0.504470000	0.390062000	2.154556000
H	-5.313047000	-2.402800000	1.293865000
H	-3.616509000	-2.909715000	1.281206000
H	-4.430327000	-3.230896000	-0.902852000
H	-3.295131000	-1.901971000	-0.947668000
H	-6.352450000	-1.764762000	-1.134102000
H	-5.167425000	-1.293812000	-2.347602000
H	-5.919390000	0.161624000	0.221759000
H	-5.871918000	0.782245000	-1.422152000
H	-3.905440000	1.719000000	-0.456843000
H	-3.285078000	0.328208000	-1.308505000
H	-1.657649000	1.173318000	3.219293000

64

F, *E*(SCRF) = -1686.43958885 a.u.

C	-1.080724000	2.415313000	1.353439000
C	-1.420202000	2.479238000	-0.183661000
C	-0.440940000	1.531376000	-0.831384000
O	-1.469551000	1.343042000	1.948626000
O	-0.494812000	3.355633000	1.864537000
H	3.235559000	4.388709000	-0.415433000
H	0.913975000	3.729441000	0.134406000
H	1.296730000	0.276074000	-2.389028000
H	3.636678000	0.938116000	-2.946742000
H	4.597795000	3.000904000	-1.954907000
C	0.960385000	1.961864000	-1.108694000
C	1.740195000	1.170739000	-1.961013000
H	-1.168773000	3.498552000	-0.485926000
C	3.043080000	1.543294000	-2.267901000
C	1.502251000	3.129883000	-0.554878000
C	2.814585000	3.489068000	-0.852345000
C	-2.426889000	-2.081906000	1.770094000
H	-2.062984000	-2.979206000	1.254096000
H	-2.325940000	-2.305199000	2.841900000

C	-3.908867000	-1.852385000	1.447572000
H	-4.076954000	-1.772276000	0.367288000
H	-4.545799000	-2.672026000	1.805018000
H	-4.282622000	-0.928347000	1.903315000
Zn	-1.199873000	-0.527301000	1.484485000
O	-0.767567000	0.367604000	-1.098209000
C	3.581425000	2.705181000	-1.712526000
C	-2.896194000	2.126571000	-0.454820000
H	-3.302496000	2.864576000	-1.156385000
H	-3.472353000	2.228138000	0.468356000
Zn	-3.098322000	0.295183000	-1.257501000
C	-3.497358000	-1.376792000	-2.269380000
H	-4.491567000	-1.758162000	-2.007171000
H	-3.548945000	-1.129391000	-3.338230000
C	-2.447303000	-2.475743000	-2.054059000
H	-2.393055000	-2.776267000	-1.000047000
H	-2.659179000	-3.382455000	-2.636443000
H	-1.446422000	-2.130655000	-2.340395000
N	0.868721000	-0.428773000	1.371321000
C	1.584802000	-1.071264000	0.492634000
C	2.743804000	-3.919001000	-0.784860000
C	1.465204000	-3.556969000	-0.026923000
C	0.922061000	-2.149164000	-0.330983000
C	3.940887000	-3.013781000	-0.493260000
C	3.654438000	-1.535767000	-0.769810000
H	2.531020000	-3.877678000	-1.862254000
H	0.686758000	-4.278989000	-0.294225000
H	1.023478000	-1.920531000	-1.399118000
H	4.257676000	-3.116749000	0.552522000
H	4.589737000	-0.979494000	-0.876589000
C	1.501620000	0.587434000	2.218694000
H	0.941390000	0.644488000	3.155057000
C	3.651818000	0.087014000	1.117140000
C	2.966350000	0.263071000	2.461171000
H	3.052575000	-0.663066000	3.040887000
H	3.450841000	1.063579000	3.025997000
H	4.667782000	-0.300723000	1.246949000
N	2.910194000	-0.872427000	0.298532000
H	3.122789000	-1.424144000	-1.722905000
H	4.785467000	-3.328999000	-1.116531000
H	3.013151000	-4.957017000	-0.562009000
H	1.621975000	-3.654318000	1.055121000
H	-0.148044000	-2.117970000	-0.121294000
H	1.404440000	1.573556000	1.747522000

H	3.717649000	1.045590000	0.583572000

91

TS6, *E*(SCRF) = -2148.4526852 a.u., IF = *i*266.5039 cm^{-1}

Zn	0.758550000	-1.282458000	1.446230000
O	-0.011619000	0.050459000	-0.180130000
C	0.065144000	-1.012467000	-0.888355000
C	-0.213287000	-2.721564000	0.069765000
C	-1.032569000	-2.004356000	-1.012119000
C	3.417599000	-1.272004000	-3.588665000
H	2.755291000	-3.274142000	-4.023476000
H	0.842707000	-3.186972000	-2.482887000
H	1.871217000	0.827069000	-1.404334000
H	3.808389000	0.755396000	-2.967531000
H	4.257683000	-1.313787000	-4.275289000
C	1.240218000	-1.168305000	-1.820632000
C	2.085355000	-0.066908000	-1.979560000
C	3.165561000	-0.113819000	-2.854407000
C	1.487001000	-2.318838000	-2.578407000
C	2.572963000	-2.371874000	-3.448217000
C	0.300455000	-0.922151000	3.374914000
H	1.118237000	-1.299017000	4.005464000
H	0.284735000	0.164337000	3.541491000
C	-1.025255000	-1.542889000	3.832813000
H	-0.984631000	-2.637537000	3.778755000
H	-1.284537000	-1.283829000	4.869153000
H	-1.861630000	-1.238936000	3.193175000
O	-2.710227000	-1.424004000	0.623009000
Zn	-4.519359000	-0.320301000	-0.127461000
C	-6.426981000	-0.850304000	0.170729000
H	-6.744975000	-0.502629000	1.162307000
H	-7.073022000	-0.324637000	-0.543863000
C	-6.661245000	-2.363149000	0.059026000
H	-6.053767000	-2.916874000	0.783614000
H	-7.709132000	-2.643795000	0.233354000
H	-6.388066000	-2.738765000	-0.933566000
C	-2.372072000	-1.449960000	-0.584313000
O	-3.111836000	-0.977865000	-1.505140000
H	-1.119545000	-2.483582000	-1.986795000
H	0.375237000	-3.557557000	-0.302843000
H	-0.883480000	-2.993418000	0.891165000
C	5.375035000	-1.267371000	-0.173061000
C	4.895249000	-2.475223000	0.614948000
C	3.377324000	-2.464006000	0.590408000
N	2.816838000	-1.154980000	0.909066000

C	3.526252000	-0.069475000	0.837843000
N	4.824680000	-0.043059000	0.413797000
C	5.722542000	1.116642000	0.398459000
C	5.506171000	2.135762000	1.515773000
C	4.425972000	3.164684000	1.181821000
C	3.217618000	2.497793000	0.527411000
C	2.798561000	1.208104000	1.236841000
H	5.060866000	-1.350877000	-1.222655000
H	6.464915000	-1.192591000	-0.151405000
H	5.263760000	-2.405527000	1.644109000
H	5.283855000	-3.397995000	0.175288000
H	2.968860000	-3.186368000	1.306625000
H	5.689895000	1.615140000	-0.582489000
H	6.730228000	0.704455000	0.501876000
H	6.456825000	2.644232000	1.705382000
H	5.258273000	1.591676000	2.434218000
H	4.834516000	3.929961000	0.511958000
H	4.117054000	3.678568000	2.099120000
H	3.431956000	2.278182000	-0.526883000
H	2.367642000	3.189265000	0.522998000
H	1.746770000	1.010706000	1.013308000
H	2.868095000	1.326214000	2.326466000
H	3.011496000	-2.758915000	-0.402469000
C	-0.582610000	2.629066000	-2.813114000
C	-1.729147000	1.636703000	-2.616760000
C	-2.935423000	2.170101000	-1.824702000
C	0.047124000	3.132704000	-1.514488000
C	-2.749788000	2.161221000	-0.325538000
C	-0.963589000	3.839162000	-0.608243000
H	-0.945544000	3.495324000	-3.386102000
H	-2.094781000	1.322450000	-3.599796000
H	-3.185070000	3.189569000	-2.150747000
H	0.466272000	2.288270000	-0.952395000
H	-0.449674000	4.498216000	0.099501000
C	-3.361821000	1.525941000	1.851643000
H	-3.908917000	0.703763000	2.323002000
C	-1.186391000	2.629908000	1.522311000
C	-1.877250000	1.445405000	2.172834000
H	-1.490636000	0.512669000	1.754122000
H	-1.693312000	1.446663000	3.250929000
H	-0.117103000	2.413852000	1.385353000
N	-1.758306000	2.924248000	0.203235000
N	-3.554362000	1.448308000	0.405212000
H	-1.628997000	4.478960000	-1.199498000

H	0.865433000	3.824692000	-1.750446000
H	0.191454000	2.151454000	-3.425629000
H	-1.364760000	0.736457000	-2.118221000
H	-3.809333000	1.549921000	-2.035233000
H	-3.789335000	2.468881000	2.223872000
H	-1.271917000	3.532656000	2.142017000

37

TS6', *E*(SCRF) = -1224.34001018 a.u., IF = *i*395.2882 cm^{-1}

Zn	0.148581000	1.592543000	0.306292000
O	0.936706000	0.306201000	-1.074423000
C	1.569021000	-0.317348000	-0.143348000
C	1.187476000	0.085023000	1.694455000
C	0.880939000	-1.180113000	0.877613000
C	5.828939000	-0.565778000	-0.602133000
H	5.815231000	-1.888887000	1.097035000
H	3.383710000	-1.758591000	1.420800000
H	3.056748000	0.882381000	-1.942988000
H	5.525957000	0.765359000	-2.266558000
H	6.903839000	-0.626002000	-0.740631000
C	3.061787000	-0.411608000	-0.242292000
C	3.678743000	0.291591000	-1.280135000
C	5.055838000	0.215571000	-1.457403000
C	3.841471000	-1.201859000	0.608927000
C	5.218406000	-1.274938000	0.430216000
C	-0.726903000	3.379948000	0.290951000
H	-0.462985000	3.933524000	1.198346000
H	-0.345359000	3.968119000	-0.550384000
C	-2.253131000	3.244487000	0.185345000
H	-2.667203000	2.672455000	1.023848000
H	-2.756891000	4.219829000	0.179467000
H	-2.549565000	2.723615000	-0.732479000
O	-1.271584000	-0.183472000	0.552245000
Zn	-2.887210000	-1.362465000	-0.094976000
C	-4.771908000	-1.243052000	-0.667680000
H	-4.908672000	-1.903542000	-1.530193000
H	-5.392397000	-1.655446000	0.135418000
C	-5.218009000	0.185015000	-1.008317000
H	-4.637648000	0.604805000	-1.835764000
H	-6.273250000	0.211821000	-1.304592000
H	-5.101339000	0.862998000	-0.157093000
C	-0.577069000	-1.263490000	0.578266000
O	-1.154434000	-2.354603000	0.320166000
H	1.304082000	-2.151939000	1.130676000
H	2.152678000	0.092337000	2.186223000

H	0.372847000	0.339131000	2.378702000

64

TS6'', *E*(SCRF) = -1686.39908972 a.u., IF = *i*378.3296 cm^{-1}

Zn	0.470829000	0.436646000	1.939315000
O	1.242720000	0.381086000	-0.015429000
C	1.982787000	-0.614640000	0.332980000
C	1.789484000	-1.415918000	2.071962000
C	1.434080000	-1.967018000	0.686278000
C	6.179643000	-0.149961000	-0.455965000
H	6.385076000	-2.225807000	0.077729000
H	3.997636000	-2.532007000	0.577054000
H	3.240196000	1.565181000	-0.430999000
H	5.663242000	1.885669000	-0.928758000
H	7.234893000	-0.019088000	-0.674055000
C	3.461197000	-0.490353000	0.109084000
C	3.945557000	0.748520000	-0.320428000
C	5.297178000	0.918249000	-0.599271000
C	4.351249000	-1.560745000	0.245579000
C	5.702967000	-1.388994000	-0.033511000
C	-0.160921000	2.029903000	2.972809000
H	0.430488000	2.095242000	3.893562000
H	0.080664000	2.937056000	2.403714000
C	-1.653619000	2.019674000	3.331571000
H	-1.919094000	1.139747000	3.927257000
H	-1.946253000	2.904434000	3.912163000
H	-2.294772000	1.997904000	2.441382000
O	-0.762651000	-1.133708000	1.141484000
Zn	-2.458469000	-1.756386000	-0.262285000
C	-4.278696000	-2.198176000	0.429399000
H	-4.826999000	-2.792515000	-0.311088000
H	-4.176033000	-2.837545000	1.313515000
C	-5.098721000	-0.950570000	0.790425000
H	-5.261182000	-0.307029000	-0.083301000
H	-6.090427000	-1.200196000	1.189886000
H	-4.594058000	-0.339927000	1.549553000
C	-0.057999000	-1.987634000	0.493885000
O	-0.591904000	-2.776916000	-0.318229000
H	1.908938000	-2.861587000	0.282322000
H	2.807018000	-1.587257000	2.401902000
H	1.056632000	-1.708985000	2.830492000
C	0.513845000	0.655319000	-3.039659000
C	0.358922000	-0.851967000	-2.946811000
C	-1.124557000	-1.182503000	-2.916636000
N	-1.755248000	-0.528718000	-1.775538000

C	-1.429386000	0.714913000	-1.535184000
N	-0.493413000	1.380711000	-2.247429000
C	-0.229048000	2.824490000	-2.159202000
C	-0.143078000	3.399276000	-0.743585000
C	-1.509735000	3.824072000	-0.197335000
C	-2.607460000	2.825207000	-0.568181000
C	-2.154364000	1.373517000	-0.377300000
H	0.436735000	0.990026000	-4.082177000
H	1.496228000	0.949811000	-2.653360000
H	0.814858000	-1.224018000	-2.026091000
H	0.861403000	-1.325837000	-3.794701000
H	-1.285999000	-2.259983000	-2.835650000
H	-0.972852000	3.372699000	-2.753092000
H	0.730510000	2.969291000	-2.660112000
H	0.528694000	4.264170000	-0.758572000
H	0.330077000	2.647280000	-0.102184000
H	-1.775085000	4.815367000	-0.582968000
H	-1.450835000	3.912441000	0.893846000
H	-2.936427000	2.975117000	-1.603581000
H	-3.488782000	2.999234000	0.056795000
H	-3.026474000	0.746673000	-0.168935000
H	-1.501573000	1.307963000	0.502309000
H	-1.607866000	-0.836944000	-3.842027000

91

G, *E*(SCRF) = -2148.48556878 a.u.

Zn	-0.712940000	-0.864971000	-1.752796000
O	-0.063004000	-0.178513000	-0.068597000
C	-0.182527000	-1.177714000	0.862059000
C	0.246866000	-2.590299000	0.516526000
C	1.089125000	-1.791672000	1.469637000
C	-3.424929000	-0.605364000	3.673149000
H	-2.979807000	-2.593379000	4.372026000
H	-1.140503000	-2.956712000	2.793974000
H	-1.741286000	0.947151000	1.152950000
H	-3.596048000	1.342515000	2.771192000
H	-4.230042000	-0.443922000	4.383119000
C	-1.318176000	-1.022040000	1.842268000
C	-2.031437000	0.179345000	1.861240000
C	-3.070734000	0.389734000	2.764999000
C	-1.681810000	-2.014880000	2.763413000
C	-2.721864000	-1.809453000	3.666180000
C	-0.045010000	-1.420757000	-3.553607000
H	-0.852744000	-1.903797000	-4.117535000
H	0.223601000	-0.525261000	-4.129188000

C	1.166698000	-2.361881000	-3.466242000
H	0.891030000	-3.320712000	-3.010892000
H	1.592522000	-2.588713000	-4.452548000
H	1.969175000	-1.946022000	-2.845912000
O	2.777758000	-1.416080000	-0.194931000
Zn	4.569644000	-0.252274000	0.428924000
C	6.493787000	-0.770857000	0.220613000
H	6.817187000	-0.536337000	-0.802407000
H	7.116246000	-0.144889000	0.872643000
C	6.769544000	-2.251621000	0.515975000
H	6.186819000	-2.906630000	-0.141432000
H	7.826996000	-2.520628000	0.386114000
H	6.494977000	-2.512839000	1.544185000
C	2.408158000	-1.276415000	0.999632000
O	3.136612000	-0.674735000	1.855543000
H	1.051665000	-2.012825000	2.531326000
H	-0.307498000	-3.445862000	0.891723000
H	0.719452000	-2.726734000	-0.456377000
C	-4.910260000	-1.696873000	0.443886000
C	-4.499682000	-2.798189000	-0.522043000
C	-3.037837000	-2.566404000	-0.865430000
N	-2.736082000	-1.170195000	-1.169853000
C	-3.559991000	-0.207351000	-0.878735000
N	-4.724987000	-0.395538000	-0.203122000
C	-5.782656000	0.592957000	0.023287000
C	-5.963828000	1.615915000	-1.096884000
C	-5.028237000	2.818048000	-0.964722000
C	-3.624988000	2.384836000	-0.546259000
C	-3.115580000	1.177765000	-1.334344000
H	-4.302838000	-1.741240000	1.357945000
H	-5.960566000	-1.787132000	0.729337000
H	-5.125603000	-2.746531000	-1.419420000
H	-4.635483000	-3.783225000	-0.067121000
H	-2.729161000	-3.168577000	-1.728103000
H	-5.632159000	1.097200000	0.989782000
H	-6.706175000	0.014351000	0.114676000
H	-7.004620000	1.954513000	-1.092212000
H	-5.810213000	1.104751000	-2.054569000
H	-5.428121000	3.521262000	-0.225493000
H	-4.985637000	3.353806000	-1.919838000
H	-3.604069000	2.146655000	0.523864000
H	-2.923676000	3.213455000	-0.686088000
H	-2.022196000	1.157785000	-1.258576000
H	-3.354402000	1.286331000	-2.400593000

H	-2.410587000	-2.870410000	-0.017130000
C	0.251030000	2.939492000	2.142372000
C	1.391768000	1.923459000	2.199574000
C	2.704840000	2.401444000	1.551214000
C	-0.216444000	3.259458000	0.722689000
C	2.739436000	2.183875000	0.059249000
C	0.902421000	3.818953000	-0.158602000
H	0.565192000	3.874003000	2.630871000
H	1.605000000	1.678963000	3.244696000
H	2.875638000	3.464245000	1.772512000
H	-0.592038000	2.343971000	0.244943000
H	0.481417000	4.342005000	-1.024127000
C	3.622358000	1.261440000	-1.903484000
H	4.276364000	0.429762000	-2.182122000
C	1.370065000	2.237403000	-1.988019000
C	2.186083000	1.006255000	-2.341612000
H	1.824063000	0.137965000	-1.781912000
H	2.111132000	0.797806000	-3.412564000
H	0.301685000	1.980436000	-1.912116000
N	1.792998000	2.799782000	-0.701677000
N	3.655063000	1.418127000	-0.451579000
H	1.491423000	4.559365000	0.394472000
H	-1.037784000	3.986165000	0.761650000
H	-0.596090000	2.552545000	2.720862000
H	1.075196000	0.994811000	1.714194000
H	3.546677000	1.848563000	1.971250000
H	4.016565000	2.171530000	-2.379825000
H	1.471215000	3.015557000	-2.756417000

64

G', *E*(SCRF) = -1686.42440376 a.u.

Zn	2.975598000	0.258493000	-0.343746000
O	2.243054000	1.240533000	-1.805173000
C	0.979313000	1.704872000	-1.699174000
C	-0.106528000	0.592168000	-2.044827000
C	1.663118000	3.949980000	1.287500000
C	0.414032000	4.490293000	1.569962000
H	-1.635884000	4.617400000	0.923640000
H	-1.353036000	3.085776000	-0.963612000
H	2.818730000	2.716146000	-0.029931000
H	2.525062000	4.217720000	1.891498000
H	0.283358000	5.170571000	2.405643000
C	0.739658000	2.701678000	-0.590062000
C	1.823408000	3.074747000	0.214463000
H	0.707640000	1.554060000	-3.872249000

H	-0.625075000	2.495669000	-3.016419000
C	0.172587000	1.761094000	-2.951605000
C	-0.501345000	3.302307000	-0.331594000
C	-0.661204000	4.173997000	0.741368000
C	4.689065000	-0.254730000	0.554716000
H	5.346072000	-0.777392000	-0.150754000
H	5.241303000	0.641746000	0.860886000
C	4.426453000	-1.145366000	1.776566000
H	3.894928000	-2.064558000	1.499552000
H	5.348790000	-1.447835000	2.290018000
H	3.797277000	-0.636796000	2.518259000
Zn	-3.438741000	0.046908000	-0.198931000
C	-1.390330000	0.386454000	-1.379542000
O	-2.347273000	1.231468000	-1.433624000
C	-5.076266000	-0.263491000	0.867345000
H	-5.601979000	-1.121493000	0.432759000
H	-4.771220000	-0.576845000	1.872657000
C	-6.019925000	0.943152000	0.955363000
H	-6.366837000	1.259184000	-0.033418000
H	-6.909936000	0.716079000	1.554454000
H	-5.531210000	1.807477000	1.416097000
O	-1.594711000	-0.689529000	-0.705829000
H	0.414832000	-0.334095000	-2.254602000
C	-0.709229000	-1.695678000	2.289425000
C	0.254071000	-0.683374000	2.878998000
C	0.753282000	0.204971000	1.754418000
N	1.291448000	-0.584959000	0.651037000
C	0.839858000	-1.778135000	0.414849000
N	-0.084310000	-2.425096000	1.188188000
C	-0.906811000	-3.547680000	0.720953000
C	-0.139531000	-4.723907000	0.114962000
C	0.115892000	-4.572379000	-1.387187000
C	0.482374000	-3.134914000	-1.757745000
C	1.468774000	-2.507369000	-0.760946000
H	-1.622139000	-1.193936000	1.930021000
H	-1.009329000	-2.429157000	3.044755000
H	1.099862000	-1.207320000	3.338456000
H	-0.245453000	-0.096445000	3.654616000
H	1.539614000	0.881741000	2.106488000
H	-1.674543000	-3.177767000	0.024644000
H	-1.430486000	-3.902641000	1.612685000
H	-0.711131000	-5.639594000	0.297498000
H	0.801372000	-4.835901000	0.665094000
H	-0.777129000	-4.872822000	-1.946762000

H	0.919758000	-5.254363000	-1.687200000
H	-0.417257000	-2.511448000	-1.798879000
H	0.926338000	-3.113485000	-2.757810000
H	2.081794000	-1.770393000	-1.288013000
H	2.162763000	-3.266691000	-0.377372000
H	-0.055349000	0.843860000	1.370835000

64

G'', *E*(SCRF) = -1686.44680565 a.u.

Zn	0.065484000	0.317718000	-1.890675000
O	-1.641087000	0.530340000	-1.115657000
C	-2.470314000	-0.532747000	-0.957544000
C	-2.392854000	-1.699156000	-1.883812000
C	-1.900038000	-1.922798000	-0.473814000
C	-6.244354000	0.579751000	0.811967000
H	-6.775733000	-1.500682000	0.644510000
H	-4.646203000	-2.168772000	-0.367534000
H	-3.277596000	1.887243000	-0.225465000
H	-5.430724000	2.570734000	0.824069000
H	-7.188416000	0.870804000	1.261599000
C	-3.800891000	-0.177391000	-0.349521000
C	-4.046423000	1.154607000	-0.009130000
C	-5.257159000	1.529395000	0.568764000
C	-4.802581000	-1.124615000	-0.108751000
C	-6.011846000	-0.750002000	0.466284000
C	1.555537000	0.801209000	-3.122881000
H	1.142098000	0.990114000	-4.120656000
H	2.048127000	1.737088000	-2.829784000
C	2.586674000	-0.334915000	-3.197443000
H	2.130113000	-1.266969000	-3.546155000
H	3.415282000	-0.100661000	-3.877527000
H	3.025380000	-0.559381000	-2.216573000
O	0.426528000	-1.451708000	-0.877525000
Zn	1.842624000	-1.934359000	0.917770000
C	3.610653000	-2.851319000	0.737295000
H	3.723778000	-3.534136000	1.588707000
H	3.571157000	-3.491591000	-0.151716000
C	4.842975000	-1.940939000	0.655565000
H	4.923068000	-1.282836000	1.530044000
H	5.779667000	-2.510395000	0.598917000
H	4.812717000	-1.295624000	-0.231278000
C	-0.470831000	-2.012239000	-0.144415000
O	-0.120165000	-2.598343000	0.916437000
H	-2.526391000	-2.492913000	0.202338000
H	-3.299218000	-2.220885000	-2.175800000

H	-1.630351000	-1.650759000	-2.659507000
C	-0.730651000	1.912987000	1.950972000
C	-1.103100000	0.469620000	2.221609000
C	0.113592000	-0.240064000	2.790183000
N	1.234552000	-0.155648000	1.852203000
C	1.436994000	0.979497000	1.237696000
N	0.585630000	2.035210000	1.303214000
C	0.909812000	3.423114000	0.939461000
C	1.631641000	3.623917000	-0.391465000
C	3.151999000	3.469225000	-0.294138000
C	3.554690000	2.312969000	0.621676000
C	2.694079000	1.062466000	0.391095000
H	-0.716457000	2.495818000	2.882600000
H	-1.464237000	2.364392000	1.275506000
H	-1.404233000	-0.008361000	1.286027000
H	-1.954286000	0.433911000	2.907213000
H	-0.096512000	-1.292897000	2.982491000
H	1.470957000	3.898776000	1.757253000
H	-0.055632000	3.931456000	0.887772000
H	1.389873000	4.625876000	-0.760659000
H	1.210839000	2.921794000	-1.120474000
H	3.597831000	4.399697000	0.075449000
H	3.558619000	3.301212000	-1.298152000
H	3.483591000	2.613690000	1.674220000
H	4.603824000	2.053972000	0.451519000
H	3.278560000	0.174394000	0.639114000
H	2.431311000	0.986300000	-0.672097000
H	0.416828000	0.224120000	3.739305000

37

G''', *E*(SCRF) = -1224.39147577 a.u.

Zn	0.372602000	1.814988000	-0.105976000
O	-1.388369000	1.232081000	0.062131000
C	-1.959121000	0.168179000	-0.537424000
C	-1.571801000	-0.224204000	-1.914377000
C	-1.114349000	-1.158072000	-0.808595000
C	-5.977508000	-0.561132000	0.845224000
H	-6.040180000	-2.149578000	-0.608274000
H	-3.758518000	-1.768491000	-1.414777000
H	-3.293314000	1.466429000	1.359128000
H	-5.606442000	1.079747000	2.185722000
H	-6.988647000	-0.733565000	1.200154000
C	-3.364118000	-0.116121000	-0.072978000
C	-3.904352000	0.675581000	0.941363000
C	-5.201508000	0.453768000	1.396275000

C	-4.150267000	-1.133786000	-0.623746000
C	-5.445406000	-1.354942000	-0.168474000
C	1.956577000	2.995516000	0.000431000
H	1.956194000	3.688155000	-0.848102000
H	1.910426000	3.618500000	0.899612000
C	3.249528000	2.168155000	-0.003247000
H	3.311133000	1.519977000	-0.886586000
H	4.150197000	2.795467000	0.000028000
H	3.316154000	1.516085000	0.878431000
O	1.060724000	-0.165453000	-0.550093000
Zn	2.592561000	-1.331481000	0.331280000
C	4.476890000	-1.379963000	0.923120000
H	4.530967000	-0.840694000	1.875856000
H	4.722189000	-2.423576000	1.147740000
C	5.490810000	-0.796671000	-0.068494000
H	5.292905000	0.259049000	-0.277214000
H	6.511966000	-0.864045000	0.324873000
H	5.477081000	-1.327644000	-1.025611000
C	0.277309000	-1.192800000	-0.407155000
O	0.792183000	-2.223722000	0.121575000
H	-1.633454000	-2.098493000	-0.671093000
H	-2.316660000	-0.616648000	-2.599227000
H	-0.789291000	0.369833000	-2.380193000

64

TS7, *E*(SCRF) = -1686.40327795 a.u., IF = *i*151.222 cm^{-1}

Zn	0.623274000	0.526546000	0.460273000
O	0.774635000	2.116544000	-0.854516000
C	-0.448519000	1.968219000	-1.247544000
C	-0.694073000	0.050738000	-1.673658000
C	-2.077171000	3.962851000	1.505320000
C	-3.430429000	3.885790000	1.183277000
H	-4.883408000	3.110610000	-0.202798000
H	-3.229968000	1.955259000	-1.596501000
H	-0.078995000	3.389788000	0.941528000
H	-1.753888000	4.525393000	2.375608000
H	-4.168641000	4.384222000	1.804142000
C	-1.531467000	2.588484000	-0.417452000
C	-1.136177000	3.322411000	0.709259000
H	0.039320000	1.370771000	-3.262704000
H	-1.742403000	1.491215000	-2.949995000
C	-0.759305000	1.296240000	-2.526005000
C	-2.891887000	2.529908000	-0.744635000
C	-3.831269000	3.171866000	0.057158000
C	-0.249001000	0.326790000	2.251377000

H	0.182149000	1.034009000	2.971427000
H	-1.304006000	0.610893000	2.148804000
C	-0.143096000	-1.104820000	2.793526000
H	0.902908000	-1.431601000	2.872955000
H	-0.587373000	-1.216531000	3.792004000
H	-0.652068000	-1.813418000	2.129510000
Zn	-3.638110000	-1.877160000	-0.251236000
C	-1.816764000	-0.685364000	-1.211220000
O	-3.033201000	-0.320396000	-1.419713000
C	-5.205644000	-2.798466000	0.536554000
H	-5.240626000	-3.820058000	0.140300000
H	-5.035995000	-2.901915000	1.614454000
C	-6.544642000	-2.093105000	0.283074000
H	-6.757482000	-2.000767000	-0.787024000
H	-7.384587000	-2.635680000	0.735008000
H	-6.552834000	-1.080230000	0.698902000
O	-1.642795000	-1.762530000	-0.501756000
H	0.257324000	-0.471043000	-1.684237000
C	4.074410000	-2.853350000	-0.022547000
C	2.672681000	-3.079452000	0.513557000
C	1.729639000	-2.147348000	-0.222797000
N	2.174745000	-0.757504000	-0.091858000
C	3.445148000	-0.480744000	-0.029501000
N	4.426273000	-1.428475000	-0.010445000
C	5.857746000	-1.180521000	-0.231973000
C	6.457553000	0.004696000	0.524767000
C	6.306280000	1.331989000	-0.221853000
C	4.924104000	1.467164000	-0.861044000
C	3.795339000	0.994152000	0.064978000
H	4.162824000	-3.247735000	-1.044856000
H	4.810207000	-3.377741000	0.595696000
H	2.641068000	-2.858463000	1.586070000
H	2.387582000	-4.125548000	0.374806000
H	0.701338000	-2.220248000	0.144847000
H	6.064110000	-1.089865000	-1.308710000
H	6.358003000	-2.093184000	0.101337000
H	7.518855000	-0.201075000	0.697231000
H	5.989249000	0.052624000	1.513953000
H	7.077690000	1.411900000	-0.996212000
H	6.474913000	2.159217000	0.476706000
H	4.879216000	0.900817000	-1.799237000
H	4.740518000	2.510217000	-1.132925000
H	2.882694000	1.534841000	-0.192129000
H	4.034588000	1.235089000	1.109134000

H	1.695806000	-2.409191000	-1.289918000

91

TS7', *E*(SCRF) = -2148.44122841 a.u., IF = *i*180.4538 cm^{-1}

Zn	-2.104220000	0.627338000	-0.038527000
O	-2.830456000	1.705979000	1.613590000
C	-1.603846000	1.970267000	1.924014000
C	-0.558294000	0.299573000	1.806123000
C	-1.121553000	4.903800000	-0.408446000
C	0.150331000	5.361722000	-0.069752000
H	1.849111000	5.082694000	1.221882000
H	0.909567000	3.130056000	2.363562000
H	-2.661695000	3.457794000	0.007262000
H	-1.690906000	5.399109000	-1.188862000
H	0.583331000	6.210576000	-0.590472000
C	-0.943391000	3.141661000	1.250990000
C	-1.666008000	3.810313000	0.253130000
H	-1.561918000	0.756067000	3.704444000
H	-0.026100000	1.679123000	3.428836000
C	-0.891899000	1.203825000	2.971996000
C	0.322411000	3.625595000	1.603135000
C	0.862035000	4.726457000	0.943797000
C	-1.762500000	1.186664000	-1.933927000
H	-2.502179000	1.923989000	-2.272922000
H	-0.790019000	1.692196000	-1.973340000
C	-1.757508000	-0.022066000	-2.877682000
H	-2.719936000	-0.553761000	-2.858864000
H	-1.563050000	0.247692000	-3.925192000
H	-0.985248000	-0.745054000	-2.583689000
Zn	2.338837000	0.967162000	-0.542937000
C	0.695281000	0.315851000	1.103976000
O	1.641620000	1.111660000	1.439325000
C	2.209103000	2.496157000	-1.850557000
H	2.175139000	2.141577000	-2.890299000
H	1.239554000	2.980970000	-1.674317000
C	3.318222000	3.546997000	-1.702377000
H	4.316677000	3.123771000	-1.877467000
H	3.205831000	4.390399000	-2.398798000
H	3.330335000	3.968782000	-0.690380000
O	0.844506000	-0.404755000	0.041716000
H	-1.162015000	-0.600049000	1.729249000
C	6.199310000	-1.890063000	-1.560731000
C	5.407781000	-1.071856000	-2.562854000
C	4.890291000	0.166696000	-1.856273000
N	4.131354000	-0.181038000	-0.655764000

C	4.439831000	-1.246660000	0.026267000
N	5.416112000	-2.131427000	-0.346142000
C	5.959182000	-3.213067000	0.486312000
C	4.934841000	-4.044452000	1.258099000
C	4.580633000	-3.438543000	2.617045000
C	4.418625000	-1.921879000	2.522707000
C	3.626005000	-1.485865000	1.284707000
H	7.139713000	-1.380095000	-1.305369000
H	6.461551000	-2.864259000	-1.985400000
H	4.563742000	-1.662042000	-2.936136000
H	6.042002000	-0.809649000	-3.414181000
H	4.239372000	0.755889000	-2.508110000
H	6.724818000	-2.819732000	1.171910000
H	6.482192000	-3.872574000	-0.211112000
H	5.343447000	-5.050990000	1.395615000
H	4.042522000	-4.154918000	0.632559000
H	5.360007000	-3.683939000	3.347950000
H	3.651247000	-3.889454000	2.982829000
H	5.401440000	-1.434191000	2.517448000
H	3.905426000	-1.548717000	3.413643000
H	3.126538000	-0.540283000	1.494177000
H	2.826229000	-2.202103000	1.058535000
H	5.727398000	0.818785000	-1.570605000
C	-3.826025000	-3.852626000	-0.716084000
C	-2.431071000	-3.376403000	-1.079798000
C	-1.977151000	-2.381664000	-0.027748000
N	-2.954738000	-1.303491000	0.128405000
C	-4.218047000	-1.519155000	-0.085766000
N	-4.728680000	-2.718624000	-0.497882000
C	-6.150510000	-3.083272000	-0.508174000
C	-7.100485000	-2.036260000	-1.090720000
C	-7.572755000	-1.012989000	-0.055602000
C	-6.433702000	-0.573318000	0.864021000
C	-5.124900000	-0.314880000	0.107180000
H	-3.794309000	-4.484820000	0.183420000
H	-4.246597000	-4.457199000	-1.526035000
H	-2.448710000	-2.886553000	-2.059238000
H	-1.751221000	-4.230862000	-1.138533000
H	-1.008649000	-1.932629000	-0.276830000
H	-6.478639000	-3.377518000	0.500151000
H	-6.208361000	-3.983882000	-1.124786000
H	-7.965508000	-2.555208000	-1.516319000
H	-6.593659000	-1.544009000	-1.927949000
H	-8.385015000	-1.439537000	0.544239000

H -7.988462000 -0.141694000 -0.574122000
H -6.256278000 -1.328107000 1.639906000
H -6.717305000 0.339865000 1.394657000
H -4.533560000 0.413158000 0.666896000
H -5.332313000 0.128894000 -0.876393000
H -1.853156000 -2.886933000 0.941059000
64
TS7'', *E*(SCRF) = -1686.39543565 a.u., IF = *i*290.5489 cm^{-1}
Zn 3.708619000 -0.493589000 -0.716478000
O 2.583561000 0.324276000 -2.390744000
C 1.699658000 0.326429000 -1.450951000
C 1.635396000 -1.300101000 -0.527752000
C 2.706906000 3.631591000 0.082134000
C 1.914358000 3.707603000 1.222347000
H 0.415163000 2.721996000 2.411225000
H 0.224254000 0.775682000 0.921959000
H 3.217472000 2.450031000 -1.659075000
H 3.405136000 4.429047000 -0.151954000
H 1.996833000 4.559063000 1.890480000
C 1.715953000 1.491168000 -0.478987000
C 2.612054000 2.527614000 -0.762048000
H 0.341638000 -1.128141000 -2.331912000
H -0.319053000 -0.265038000 -0.870039000
C 0.569574000 -0.630384000 -1.390929000
C 0.908330000 1.579943000 0.663059000
C 1.018212000 2.678226000 1.508865000
C 5.638028000 -0.444792000 -0.281021000
H 6.038045000 -1.460209000 -0.376989000
H 6.162518000 0.172199000 -1.017896000
C 5.869984000 0.082182000 1.140806000
H 5.329188000 -0.517334000 1.878818000
H 6.933983000 0.069800000 1.410641000
H 5.518752000 1.114762000 1.246854000
Zn -1.123846000 -2.148464000 0.525855000
C 1.571209000 -1.440581000 0.973749000
O 0.403472000 -1.538177000 1.539772000
C -1.792030000 -3.711809000 -0.523797000
H -1.607237000 -3.528300000 -1.591442000
H -1.200723000 -4.599658000 -0.274586000
C -3.282345000 -4.005369000 -0.301870000
H -3.910624000 -3.132986000 -0.523626000
H -3.646953000 -4.828499000 -0.929439000
H -3.485295000 -4.280746000 0.738891000
O 2.638319000 -1.427349000 1.582790000

H	2.069916000	-2.171917000	-1.027882000
C	-3.831260000	1.743614000	-2.620797000
C	-2.755928000	0.733672000	-2.215065000
C	-3.256330000	-0.368908000	-1.258859000
C	-4.423243000	2.545962000	-1.461451000
C	-3.204241000	0.075711000	0.183714000
C	-5.019655000	1.662855000	-0.362649000
H	-4.644920000	1.203639000	-3.124760000
H	-2.369438000	0.251395000	-3.118554000
H	-4.273523000	-0.673108000	-1.533279000
H	-3.657575000	3.192019000	-1.012251000
H	-5.699366000	2.244808000	0.265565000
C	-2.201100000	0.028430000	2.343529000
H	-1.189726000	-0.210045000	2.684533000
C	-3.846877000	1.796685000	1.819588000
C	-2.463429000	1.527078000	2.382075000
H	-1.716041000	2.039876000	1.765967000
H	-2.389305000	1.914630000	3.401561000
H	-3.984735000	2.867505000	1.633361000
N	-4.018143000	1.098031000	0.543227000
N	-2.336892000	-0.485394000	0.977887000
H	-5.620128000	0.858350000	-0.800731000
H	-5.209669000	3.204086000	-1.847424000
H	-3.412429000	2.434441000	-3.359814000
H	-1.905665000	1.254557000	-1.753982000
H	-2.633354000	-1.261415000	-1.352603000
H	-2.908987000	-0.494734000	3.001456000
H	-4.628261000	1.476470000	2.521145000

37

TS7''', *E*(SCRF) = -1224.35434119 a.u., IF = *i*209.0648 cm^{-1}

Zn	-2.250395000	1.174903000	0.206928000
O	-3.119457000	-0.070927000	-1.054341000
C	-1.970436000	-0.645500000	-1.286280000
C	-0.635244000	0.767932000	-1.502594000
C	-2.091419000	-3.066979000	1.602409000
C	-0.938574000	-3.824649000	1.412222000
H	0.789893000	-4.149344000	0.170802000
H	0.268207000	-2.317826000	-1.376431000
H	-3.322469000	-1.477885000	0.832088000
H	-2.757451000	-3.283125000	2.431479000
H	-0.695830000	-4.629292000	2.099138000
C	-1.554003000	-1.748044000	-0.361006000
C	-2.401149000	-2.039925000	0.718170000
H	-1.775633000	0.136718000	-3.279955000

H	-0.426081000	-0.984632000	-2.805600000
C	-1.177673000	-0.274166000	-2.469009000
C	-0.406303000	-2.526593000	-0.556848000
C	-0.104163000	-3.555107000	0.329682000
C	-2.158229000	2.500895000	1.667210000
H	-3.169173000	2.763747000	1.993005000
H	-1.666271000	2.017845000	2.517882000
C	-1.374231000	3.747373000	1.235216000
H	-1.884054000	4.282904000	0.426738000
H	-1.259270000	4.453772000	2.066399000
H	-0.373892000	3.485830000	0.876821000
Zn	2.878374000	0.593516000	-0.001769000
C	0.701760000	0.686535000	-0.970873000
O	1.449649000	-0.333831000	-1.165462000
C	4.697239000	0.407525000	0.754122000
H	5.305990000	1.240228000	0.384091000
H	4.630064000	0.544461000	1.839257000
C	5.380811000	-0.927974000	0.432785000
H	5.488819000	-1.079036000	-0.646103000
H	6.385074000	-0.984274000	0.870262000
H	4.810903000	-1.778617000	0.820466000
O	1.170469000	1.644099000	-0.247799000
H	-1.003791000	1.783289000	-1.665528000

37

H, *E*(SCRF) = -1224.39559853 a.u.

Zn	0.545612000	-2.469188000	0.009185000
O	-1.430465000	-1.588081000	-0.495839000
C	-1.768844000	-0.772612000	0.364154000
C	0.421724000	-1.082635000	1.537798000
C	-4.968877000	0.531837000	-1.110555000
C	-5.094892000	1.765613000	-0.472060000
H	-4.217348000	3.155731000	0.917530000
H	-2.274843000	1.711083000	1.414573000
H	-3.751136000	-1.237600000	-1.330997000
H	-5.718973000	0.209327000	-1.825198000
H	-5.945066000	2.403968000	-0.691676000
C	-2.918730000	0.125381000	0.100540000
C	-3.877400000	-0.280654000	-0.835448000
H	-1.555422000	-1.570788000	2.283445000
H	-1.246304000	0.158474000	2.242499000
C	-1.061181000	-0.775747000	1.706476000
C	-3.044194000	1.366203000	0.732953000
C	-4.128692000	2.186155000	0.438748000
C	1.146568000	-3.922920000	-1.205362000

H	0.391031000	-4.718133000	-1.171105000
H	1.116882000	-3.535588000	-2.231249000
C	2.533371000	-4.507411000	-0.907327000
H	2.583365000	-4.939320000	0.098024000
H	2.806122000	-5.302283000	-1.613009000
H	3.315261000	-3.742994000	-0.964005000
Zn	2.309013000	1.928875000	0.069482000
C	1.140587000	0.075953000	1.014003000
O	0.494109000	1.018631000	0.424064000
C	3.180387000	3.512166000	-0.736235000
H	3.826136000	3.969710000	0.021705000
H	3.850965000	3.162157000	-1.529193000
C	2.202913000	4.554171000	-1.295597000
H	1.540665000	4.947579000	-0.517570000
H	2.730549000	5.409548000	-1.735039000
H	1.563323000	4.131806000	-2.077205000
O	2.418731000	0.173336000	1.063320000
H	0.910603000	-1.487405000	2.423564000

64

H', *E*(SCRF) = -1686.43561944 a.u.

Zn	0.303959000	-1.480288000	0.416387000
O	0.220570000	0.802782000	0.663759000
C	-0.299747000	1.290994000	-0.337190000
C	-0.795069000	-0.961503000	-1.318199000
C	-0.474269000	4.929939000	0.608716000
C	-1.526935000	5.381388000	-0.186756000
H	-3.032370000	4.844361000	-1.628201000
H	-2.348077000	2.463929000	-1.721440000
H	0.710838000	3.217851000	1.182534000
H	0.040210000	5.617636000	1.272308000
H	-1.831446000	6.422530000	-0.142085000
C	-0.741768000	2.711265000	-0.309681000
C	-0.092330000	3.595473000	0.557653000
H	0.640858000	0.499577000	-2.018152000
H	-1.021806000	0.975149000	-2.348055000
C	-0.383228000	0.473619000	-1.613889000
C	-1.802638000	3.164472000	-1.098769000
C	-2.197050000	4.496976000	-1.028891000
C	0.348532000	-2.481316000	2.164243000
H	0.344354000	-1.763183000	2.996125000
H	-0.561312000	-3.080030000	2.289741000
C	1.577184000	-3.393148000	2.285979000
H	2.513361000	-2.832414000	2.163118000
H	1.637953000	-3.909281000	3.254144000

H	1.578381000	-4.168586000	1.509985000
Zn	-4.402952000	-1.188761000	-0.072101000
C	-2.179033000	-1.044829000	-0.908003000
O	-2.786635000	0.008319000	-0.467258000
C	-6.249633000	-1.197085000	0.645450000
H	-6.904112000	-1.684714000	-0.086144000
H	-6.265783000	-1.838223000	1.534331000
C	-6.796119000	0.193776000	0.993864000
H	-6.825211000	0.848782000	0.116758000
H	-7.816571000	0.143268000	1.394147000
H	-6.177797000	0.694794000	1.745733000
O	-2.842291000	-2.147143000	-0.900110000
H	-0.553832000	-1.682094000	-2.099837000
C	4.620181000	-0.972257000	-2.037809000
C	3.849363000	-2.273151000	-2.164997000
C	2.404257000	-2.006060000	-1.782111000
N	2.305061000	-1.353506000	-0.480612000
C	3.282938000	-0.635414000	-0.025567000
N	4.463291000	-0.412266000	-0.695669000
C	5.345957000	0.739999000	-0.465393000
C	5.717209000	1.050429000	0.984075000
C	4.683710000	1.910117000	1.719005000
C	3.246801000	1.465964000	1.431247000
C	3.114817000	-0.062097000	1.366044000
H	4.277322000	-0.249147000	-2.794636000
H	5.689517000	-1.137677000	-2.205268000
H	4.278905000	-3.019250000	-1.487804000
H	3.925066000	-2.657410000	-3.185846000
H	1.831340000	-2.938063000	-1.732051000
H	4.925135000	1.638023000	-0.946368000
H	6.265229000	0.502134000	-1.008516000
H	6.679354000	1.573192000	0.975481000
H	5.889953000	0.103991000	1.506802000
H	4.808698000	2.960777000	1.432961000
H	4.879706000	1.853431000	2.795693000
H	2.893586000	1.896002000	0.484959000
H	2.571823000	1.849052000	2.202114000
H	2.132360000	-0.372630000	1.719830000
H	3.846548000	-0.537036000	2.030648000
H	1.917204000	-1.379804000	-2.542888000

37

TS8, *E*(SCRF) = -1224.3683365 a.u., IF = *i*72.0409 cm^{-1}

Zn	0.774233000	1.531001000	-0.168140000
C	-0.149451000	0.416785000	2.331969000

C	-1.527537000	0.924149000	1.956438000
C	-4.889526000	-1.778141000	-0.987269000
H	-4.554941000	-2.940621000	0.794033000
H	-2.828058000	-1.410476000	1.683259000
H	-3.234011000	1.081867000	-1.790104000
H	-4.997756000	-0.442513000	-2.674620000
H	-5.649331000	-2.450182000	-1.373903000
C	-2.950302000	-0.050596000	0.009598000
C	-3.546232000	0.205830000	-1.231505000
H	0.084187000	0.469678000	3.388558000
C	-4.523663000	-0.648708000	-1.720772000
H	-1.622079000	1.990156000	2.209809000
H	-2.295594000	0.410075000	2.544082000
C	-3.318559000	-1.184915000	0.741672000
C	-4.280424000	-2.052219000	0.235293000
C	1.896587000	3.084655000	-0.676095000
H	2.674332000	3.221424000	0.082522000
H	2.417971000	2.854036000	-1.611729000
C	1.072759000	4.369256000	-0.831444000
H	0.569085000	4.640566000	0.102420000
H	1.698594000	5.222067000	-1.122606000
H	0.294497000	4.261312000	-1.594270000
C	-1.891799000	0.861413000	0.491962000
O	-1.329515000	1.614571000	-0.313202000
Zn	2.341103000	-1.649755000	0.318264000
C	0.702154000	-0.332546000	1.574571000
O	1.733682000	-0.994276000	2.029814000
C	3.406286000	-2.532111000	-1.092284000
H	4.469043000	-2.475264000	-0.834236000
H	3.154182000	-3.598318000	-1.108003000
C	3.163217000	-1.914635000	-2.476847000
H	3.450538000	-0.857748000	-2.504979000
H	3.738660000	-2.425824000	-3.258561000
H	2.107680000	-1.967501000	-2.764067000
O	0.606066000	-0.398971000	0.205508000

37

I, E(SCRF) = -1224.38509722 a.u.

Zn	0.823539000	1.431955000	0.347411000
C	-0.802610000	-1.535765000	1.326429000
C	-1.417835000	-0.282154000	1.887504000
C	-5.629454000	-0.658999000	-1.188875000
H	-5.858398000	-2.099111000	0.396291000
H	-3.697546000	-1.565556000	1.445800000
H	-2.943409000	1.387847000	-1.578122000

H	-5.125166000	0.844830000	-2.645206000
H	-6.581679000	-0.900279000	-1.650712000
C	-3.181459000	-0.041771000	0.000585000
C	-3.591950000	0.627119000	-1.158085000
H	-1.361260000	-2.461854000	1.337819000
C	-4.811589000	0.320652000	-1.748330000
H	-0.690429000	0.371638000	2.385075000
H	-2.225545000	-0.498775000	2.588186000
C	-4.008363000	-1.024204000	0.558464000
C	-5.225412000	-1.331668000	-0.036710000
C	1.748957000	3.181842000	0.198396000
H	2.393649000	3.319350000	1.073857000
H	2.423660000	3.141504000	-0.664686000
C	0.784476000	4.366815000	0.059209000
H	0.123423000	4.454687000	0.928026000
H	1.318297000	5.320269000	-0.039607000
H	0.140605000	4.262535000	-0.820498000
C	-1.891974000	0.352521000	0.621402000
O	-1.176226000	1.188824000	0.027252000
Zn	2.643859000	-1.765230000	-0.304641000
C	0.431336000	-1.541018000	0.743373000
O	1.015864000	-2.606038000	0.254617000
C	4.363071000	-1.151370000	-1.049088000
H	5.185304000	-1.502062000	-0.416737000
H	4.529325000	-1.585327000	-2.040044000
C	4.371870000	0.383353000	-1.128811000
H	4.202612000	0.844161000	-0.148307000
H	5.326160000	0.770125000	-1.507204000
H	3.586558000	0.758691000	-1.795780000
O	1.210054000	-0.439858000	0.637632000

40

TS9, *E*(SCRF) = -1412.95301814 a.u., IF = *i*294.8583 cm^{-1}

Zn	-0.418127000	-1.169802000	-0.090124000
C	0.396934000	1.993532000	-0.552940000
C	0.356361000	0.506219000	-2.446711000
O	-0.533033000	-0.244064000	-2.189735000
C	1.692047000	1.685466000	0.152489000
C	6.400280000	-0.401386000	0.976379000
H	6.473662000	1.653369000	1.616464000
H	4.111823000	2.094502000	1.095231000
H	3.636969000	-1.964612000	-0.235979000
H	6.030500000	-2.404871000	0.276291000
H	7.444425000	-0.590297000	1.205136000
C	3.718960000	0.087635000	0.384150000

C	4.270364000	-1.181672000	0.166306000
H	0.391169000	2.893118000	-1.156590000
C	5.606455000	-1.422365000	0.454943000
H	1.616868000	1.867088000	1.240526000
H	2.456293000	2.381810000	-0.203574000
C	4.522724000	1.108954000	0.905916000
C	5.857132000	0.860928000	1.205719000
C	-1.324412000	-2.938861000	-0.090795000
H	-0.982264000	-3.517368000	-0.955751000
H	-1.056652000	-3.522401000	0.796310000
C	-2.848800000	-2.754212000	-0.155522000
H	-3.147629000	-2.163752000	-1.031827000
H	-3.387524000	-3.708739000	-0.216680000
H	-3.229245000	-2.230165000	0.730333000
C	2.282092000	0.297356000	0.063400000
O	1.605768000	-0.694255000	-0.230595000
O	1.250931000	0.981136000	-3.029158000
Zn	-3.087645000	0.924576000	0.619959000
C	-0.834173000	1.601908000	-0.072113000
O	-1.965551000	2.122287000	-0.428224000
C	-4.745592000	0.220923000	1.435961000
H	-4.460632000	-0.548249000	2.162926000
H	-5.199653000	1.033299000	2.015390000
C	-5.769244000	-0.346554000	0.443888000
H	-5.358426000	-1.186451000	-0.125797000
H	-6.666640000	-0.711821000	0.957880000
H	-6.094710000	0.408087000	-0.279309000
O	-0.969605000	0.543272000	0.751154000

40

J, *E*(SCRF) = -1413.00214569 a.u.

Zn	-0.461313000	0.909131000	1.477049000
C	0.326961000	-2.038637000	0.480712000
C	0.384695000	-1.688597000	2.014548000
O	-0.429975000	-0.778165000	2.431504000
C	1.675277000	-1.671919000	-0.152783000
C	5.940947000	0.896696000	-1.624795000
H	6.112768000	-1.135111000	-2.316900000
H	3.958569000	-1.838343000	-1.363196000
H	3.322914000	2.129331000	0.159567000
H	5.503407000	2.841174000	-0.807516000
H	6.893361000	1.201975000	-2.046599000
C	3.495159000	0.110703000	-0.541564000
C	3.943321000	1.428174000	-0.387863000
H	0.222238000	-3.123839000	0.422457000

C	5.160379000	1.819071000	-0.928688000
H	1.683122000	-1.887161000	-1.228004000
H	2.419862000	-2.333819000	0.306756000
C	4.283566000	-0.810690000	-1.240450000
C	5.503330000	-0.416773000	-1.778966000
C	-1.301758000	2.706415000	1.343861000
H	-1.747966000	2.999105000	2.299662000
H	-0.540414000	3.461042000	1.117675000
C	-2.376035000	2.715424000	0.244984000
H	-3.179059000	1.994755000	0.457638000
H	-2.859033000	3.694398000	0.129271000
H	-1.955602000	2.448972000	-0.733602000
C	2.181807000	-0.257908000	0.056418000
O	1.570695000	0.579443000	0.711078000
O	1.207371000	-2.307689000	2.655183000
Zn	-2.842465000	-0.463035000	-1.213225000
C	-0.859584000	-1.449272000	-0.221872000
O	-1.797975000	-2.164893000	-0.652519000
C	-4.459245000	0.349103000	-2.003351000
H	-4.186589000	1.362571000	-2.317009000
H	-4.713356000	-0.205470000	-2.912760000
C	-5.664787000	0.395232000	-1.054937000
H	-5.448389000	0.972552000	-0.150438000
H	-6.529792000	0.864176000	-1.538279000
H	-5.973169000	-0.605814000	-0.738025000
O	-0.959334000	-0.171884000	-0.381112000

57

K, E(SCRF) = -1231.29846874 a.u.

Zn	0.047599000	2.061741000	0.066415000
O	-0.494864000	-0.219103000	-1.690256000
C	-1.346067000	-0.466559000	-0.855520000
C	-1.825908000	1.421226000	0.606056000
C	-2.427406000	0.550379000	-0.516210000
C	-6.224599000	-1.424774000	0.154032000
H	-6.065650000	-2.016172000	-1.912351000
H	-3.907253000	-0.861157000	-2.264728000
H	-3.946491000	0.484417000	1.801125000
H	-6.105147000	-0.668702000	2.163327000
H	-7.174064000	-1.927048000	0.311274000
C	-3.768429000	-0.119517000	-0.249823000
C	-4.404159000	-0.072930000	0.990128000
C	-5.623420000	-0.719987000	1.191334000
C	-4.388440000	-0.826310000	-1.288417000
C	-5.601912000	-1.475252000	-1.092859000

C	1.159463000	3.631350000	-0.552183000
H	0.677004000	4.128949000	-1.402820000
H	1.098083000	4.360577000	0.269928000
C	2.636769000	3.386102000	-0.879198000
H	2.751406000	2.840434000	-1.822648000
H	3.211482000	4.317011000	-0.981517000
H	3.133420000	2.786397000	-0.104095000
H	-1.789885000	0.831743000	1.534257000
H	-2.511453000	2.254505000	0.802984000
C	-1.303812000	-1.740544000	-0.040887000
H	-2.087063000	-2.425785000	-0.381353000
H	-0.326836000	-2.215752000	-0.145755000
H	-1.511988000	-1.518048000	1.011516000
H	-2.530324000	1.168260000	-1.417386000
C	3.626238000	-2.155882000	-2.040045000
C	2.360382000	-1.301292000	-1.972356000
C	2.512482000	0.014783000	-1.188666000
C	4.122607000	-2.658466000	-0.684802000
C	2.332671000	-0.146892000	0.301065000
C	4.394922000	-1.521660000	0.302503000
H	4.427690000	-1.566379000	-2.507686000
H	2.049187000	-1.044576000	-2.989453000
H	3.490061000	0.469677000	-1.395630000
H	3.388413000	-3.341164000	-0.236579000
H	5.039653000	-1.872386000	1.114138000
C	1.215161000	0.297280000	2.327197000
H	0.193876000	0.573674000	2.605188000
C	2.947050000	-1.456631000	2.304264000
C	1.523111000	-1.140158000	2.727776000
H	0.822968000	-1.810509000	2.217170000
H	1.412151000	-1.290915000	3.804972000
H	3.126693000	-2.538727000	2.328913000
N	3.195692000	-0.986809000	0.942647000
N	1.359156000	0.479218000	0.887371000
H	4.940215000	-0.711987000	-0.195844000
H	5.046941000	-3.230470000	-0.826376000
H	3.447870000	-3.012929000	-2.698949000
H	1.530095000	-1.874685000	-1.546528000
H	1.755238000	0.725484000	-1.522022000
H	1.894098000	0.982751000	2.855824000
H	3.666396000	-0.988359000	2.990267000

57

K', *E*(SCRF) = -1231.31256113 a.u.

Zn	-0.819662000	-0.907032000	0.365115000

O	-0.427594000	1.334619000	0.930546000
C	-0.692979000	2.006374000	-0.054704000
C	-1.953944000	0.049204000	-1.095605000
C	-5.548947000	-0.879861000	-0.308623000
C	-6.031604000	0.261670000	0.334551000
H	-5.532195000	2.243915000	0.999867000
H	-3.231598000	2.166372000	0.179535000
H	-3.876337000	-1.827782000	-1.261304000
H	-6.199335000	-1.738385000	-0.451370000
H	-7.053418000	0.300886000	0.697848000
C	-3.358829000	0.157379000	-0.613499000
C	-4.241701000	-0.928669000	-0.768578000
H	-0.387857000	1.254258000	-1.971591000
H	-1.893107000	2.149466000	-1.812915000
C	-1.265705000	1.389243000	-1.317438000
C	-3.866045000	1.294853000	0.033318000
C	-5.178318000	1.345121000	0.502219000
C	-0.707177000	-2.180376000	1.928392000
H	-0.540347000	-1.611176000	2.854067000
H	-1.670061000	-2.685684000	2.072274000
C	0.397161000	-3.235110000	1.777450000
H	1.384800000	-2.775022000	1.639613000
H	0.476838000	-3.906545000	2.643941000
H	0.229101000	-3.868625000	0.897555000
H	-1.940977000	-0.525912000	-2.027645000
C	-0.426400000	3.491006000	-0.059606000
H	0.128290000	3.781456000	0.833376000
H	0.123909000	3.774779000	-0.962772000
H	-1.382125000	4.026668000	-0.087095000
C	4.189777000	1.627143000	1.643204000
C	2.681893000	1.383418000	1.558959000
C	2.291405000	-0.097699000	1.400080000
C	4.975820000	1.202782000	0.401478000
C	2.314437000	-0.541339000	-0.042148000
C	4.776912000	-0.271198000	0.034822000
H	4.584452000	1.078050000	2.509602000
H	2.206550000	1.764761000	2.467950000
H	2.957932000	-0.728952000	2.001271000
H	4.686797000	1.816818000	-0.462154000
H	5.579199000	-0.605238000	-0.629754000
C	1.240776000	-1.249905000	-2.014907000
H	0.242764000	-1.116094000	-2.442484000
C	3.634784000	-0.653485000	-2.125168000
C	2.280838000	-0.442150000	-2.781858000

H	2.010394000	0.619725000	-2.739225000
H	2.325755000	-0.736444000	-3.833815000
H	4.351915000	0.100249000	-2.472950000
N	3.526924000	-0.540709000	-0.671125000
N	1.193511000	-0.843622000	-0.616705000
H	4.841403000	-0.897437000	0.931422000
H	6.043295000	1.380828000	0.575674000
H	4.375715000	2.688928000	1.839599000
H	2.251116000	1.955496000	0.726775000
H	1.280437000	-0.264229000	1.773657000
H	1.480102000	-2.321511000	-2.078394000
H	4.041209000	-1.639702000	-2.387590000

58

L, *E*(SCRF) = -1269.41301489 a.u.

N	1.179380000	-0.407051000	1.193460000
C	-1.944303000	-1.633195000	0.493012000
C	-2.041644000	-0.391875000	-0.375578000
O	-1.138306000	-0.071345000	-1.146437000
C	-5.420322000	2.195240000	-0.035739000
H	-6.199796000	0.773834000	1.376306000
H	-2.452898000	1.886410000	-1.662715000
H	-4.408552000	3.434065000	-1.483515000
H	-6.279226000	2.854547000	0.046523000
C	-3.235926000	0.488858000	-0.245491000
C	-3.284443000	1.667488000	-1.000344000
C	-4.370902000	2.521699000	-0.897329000
C	-4.286771000	0.155461000	0.623645000
C	-5.376180000	1.025170000	0.713129000
C	2.203869000	-1.226008000	-1.999381000
H	1.881277000	-0.689902000	-2.903715000
H	2.903961000	-0.540206000	-1.495530000
C	2.962536000	-2.493085000	-2.421789000
H	2.308935000	-3.187304000	-2.962202000
H	3.821854000	-2.287092000	-3.075487000
H	3.347766000	-3.045135000	-1.554560000
Zn	0.592195000	-1.554150000	-0.814957000
C	-0.950642000	-2.648060000	-0.074166000
H	-0.699912000	-3.368749000	0.712285000
H	-1.447400000	-3.219845000	-0.871380000
C	2.627201000	3.215442000	-1.022173000
C	1.444913000	2.245034000	-1.033527000
C	0.945638000	1.840145000	0.362670000
C	3.874769000	2.699345000	-0.303030000
C	1.752959000	0.742712000	1.016459000

C	3.621498000	2.354969000	1.169656000
H	2.311743000	4.150301000	-0.536243000
H	0.610414000	2.716429000	-1.563987000
H	0.919283000	2.719129000	1.021073000
H	4.264561000	1.805549000	-0.808422000
H	4.563292000	2.360968000	1.726440000
C	1.957237000	-1.408369000	1.915410000
H	1.495896000	-2.387263000	1.748891000
C	3.994222000	-0.020416000	1.723408000
C	3.417049000	-1.404729000	1.471196000
H	3.463375000	-1.644197000	0.403933000
H	4.004950000	-2.152095000	2.011817000
H	4.899169000	0.129882000	1.121351000
N	3.037074000	1.033419000	1.375239000
H	2.986530000	3.119602000	1.632127000
H	4.657502000	3.465121000	-0.355721000
H	2.889347000	3.475264000	-2.053699000
H	1.697026000	1.340639000	-1.599129000
H	-0.072878000	1.460285000	0.292890000
H	1.898715000	-1.202762000	2.994923000
H	4.278820000	0.094945000	2.778135000
C	-4.230995000	-1.108210000	1.447529000
C	-3.350937000	-2.170113000	0.791417000
H	-3.819546000	-0.865518000	2.438301000
H	-3.263860000	-3.048430000	1.438581000
H	-5.247650000	-1.479733000	1.616211000
H	-3.811543000	-2.508165000	-0.146476000
H	-1.528700000	-1.234916000	1.438380000

58

L', *E*(SCRF) = -1269.40116285 a.u.

N	-2.335799000	0.754383000	0.524284000
C	2.277084000	1.295249000	0.511799000
C	3.467273000	0.742916000	1.274811000
O	3.691342000	1.073456000	2.422537000
C	6.450003000	-1.575422000	-0.764095000
H	4.852883000	-2.809839000	-1.492893000
H	6.040021000	1.037481000	1.356988000
H	7.836854000	-0.209106000	0.161842000
H	7.216480000	-2.135983000	-1.290728000
C	4.451440000	-0.141880000	0.551274000
C	5.796420000	0.202567000	0.707107000
C	6.797376000	-0.498496000	0.044606000
C	4.091831000	-1.245062000	-0.240675000
C	5.113802000	-1.945851000	-0.886997000

C	-1.057654000	2.857785000	-1.695677000
H	-0.560607000	2.751818000	-2.668816000
H	-2.133262000	2.770800000	-1.909530000
C	-0.761407000	4.264674000	-1.149644000
H	0.310485000	4.404783000	-0.967971000
H	-1.077846000	5.064843000	-1.832705000
H	-1.263768000	4.450786000	-0.191342000
Zn	-0.462008000	1.348406000	-0.479118000
C	-3.342697000	-3.444255000	-0.719111000
C	-2.222384000	-2.413037000	-0.872246000
C	-1.910728000	-1.619852000	0.409574000
C	-4.701509000	-2.866660000	-0.320464000
C	-2.832432000	-0.441635000	0.608786000
C	-4.657068000	-2.068839000	0.986488000
H	-3.038696000	-4.174787000	0.043673000
H	-1.310540000	-2.936656000	-1.178259000
H	-1.953489000	-2.284128000	1.281855000
H	-5.088783000	-2.214111000	-1.114304000
H	-5.663417000	-1.970604000	1.403945000
C	-3.255623000	1.867582000	0.737441000
H	-2.796979000	2.773850000	0.335514000
C	-5.178583000	0.326704000	0.683750000
C	-4.596503000	1.589146000	0.070696000
H	-4.432554000	1.450182000	-1.003764000
H	-5.293164000	2.421195000	0.202015000
H	-5.978679000	-0.076961000	0.051097000
N	-4.150430000	-0.707971000	0.827259000
H	-4.063984000	-2.601321000	1.738001000
H	-5.417533000	-3.688543000	-0.207092000
H	-3.449313000	-4.001625000	-1.655983000
H	-2.461254000	-1.708040000	-1.680144000
H	-0.897215000	-1.218747000	0.372590000
H	-3.399223000	2.015772000	1.817379000
H	-5.613489000	0.539024000	1.669326000
C	2.673022000	-1.765415000	-0.341175000
C	1.604071000	-0.772845000	-0.830073000
C	1.156240000	0.285919000	0.185176000
H	2.363642000	-2.137834000	0.646400000
H	2.687303000	-2.637755000	-1.005550000
H	2.652784000	1.730223000	-0.426495000
H	1.898767000	2.115306000	1.132574000
H	0.737437000	-1.375325000	-1.142311000
H	1.966892000	-0.281769000	-1.745119000
H	0.891492000	-0.227408000	1.124156000

I. References

(1) Jia, K.; Zhang, F.; Huang H.; Chen Y. Visible-Light-Induced Alkoxyl Radical Generation Enables Selective C(sp^3)-C(sp^3) Bond Cleavage and Functionalizations. *J. Am. Chem. Soc.* **2016**, *138*, 1514-1517.

(2) Laktsevich-Iskryk, M. V.; Varabyeva N. A.; Kazlova V. V.; Zhabinskii V. N.; Khripach V. A.; Hurski A. L. Visible-Light-Promoted Catalytic Ring-Opening Isomerization of 1,2-Disubstituted Cyclopropanols to Linear Ketones. *Eur. J. Org. Chem*. **2020**, *16*, 2431-2434.

(3) Jiao, J.; Nguyen L. X.; Patterson D. R.; Flowers R. A. An Efficient and General Approach to *β*-Functionalized Ketones. *Org. Lett*. **2007**, *9*, 1323-1326.

(4) Klikar, M.; Jelinkova, V.; Ruzickova, Z.; Mikysek, T.; Pytela, O.; Ludwig, M.; Bures, F. Malonic Acid Derivatives on Duty as Electron-Withdrawing Units in Push-Pull Molecules. Eur. *J. Org. Chem.* **2017**, 2764-2779.

(5) Muratore, M. E.; Holloway, C. A.; Pilling, A. W.; Storer, R. I.; Trevitt, G.; Dixon, D. J. Enantioselective Brønsted Acid Catalyzed *N*-Acyliminium Cyclization Cascades. *J. Am. Chem. Soc.* **2009**, *131*, 10796-10797.

(6) Mizuno, H.; Takaya, J.; Iwasawa, N. Rhodium(I)-Catalyzed Direct Car-boxylation of Arenes with CO_2 via Chelation-Assisted C-H Bond Activa-tion. *J. Am. Chem. Soc.* **2011**, *133*, 1251-1253.

(7) Chowdhury, S. R.; Ul Hoque, I.; Maity, S. TBAI/TBHP-Promoted Generation of Malonyl Radicals: Oxidative Coupling with Styrenes Leads to *γ*-Keto Diesters. *Chem. Asian J.* **2018**, *13*, 2824-2828.

(8) Bunrit, A.; Srifa, P.; Rukkijakan, T.; Dahlstrand, C.; Huang, G. P.; Biswas, S.; Watile, R. A.; Samec, J. S. M. H_3PO_2-Catalyzed Intramolecular Stereospecific Substitution of the Hydroxyl Group in Enantioenriched Secondary Alcohols by *N*-, *O*-, and *S*-centered Nucleophiles to Generate Heterocycles. *ACS Catal.* **2020**, *10*, 1344-1352.

(9) Shaw, P.; Hassell-Hart, S. J.; Douglas, G. E.; Malcolm, A. G.; Kennedy, A. R.; White, G. V.; Paterson, L. C.; Kerr, W. J. Oxygenated Cyclopentenones via the Pauson-Khand Reaction of Silyl Enol Ether Substrates. *Org. Lett.* **2022**, *24*, 2750-2755.

(10) Ishizaki, M.; Satoh, H.; Hoshino, O.; Nishitani, K.; Hara, H. Investigation of Intramolecular Pauson-Khand Reaction of 2-Aryl-1,6- and 1-Methyl-1,7-enynes (*exo*-Olefins) and 1-Phenyl-1-octen-7-yne (*endo*-Olefin). Heterocycle **2004**, *63*, 827-844.

(11) Ragusa, G.; Gomez-Canas, M.;Morales, P.; Rodriguez-Cueto, C.; Pazos, M. R.; Asproni, B.; Cichero, E.; Fossa, P.; Pinna, G. A.; Jagerovic, N.; Fernandez-Ruiz, J.; Murineddu, G. New pyridazinone-4-carboxamides as new cannabinoid receptor type-2 inverse agonists: Synthesis, pharmacological data andmolecular docking. *Eur. J. Med. Chem.* **2017**, *127*, 398-412.

(12) Kokuev, A. O.; Ioffe S. L.; Sukhorukov A. Y. Addition of malonic esters to azoalkenes generated *in situ* from a-bromo- and a-chlorohydrazones. *Tetrahedron Lett.* **2021**, *83*, 153414-153420.

(13) Chowdhury, S. R.; Ul Hoque, I.; Maity, S. TBAI/TBHP-Promoted Generation of Malonyl Radicals: Oxidative Coupling with Styrenes Leads to *γ*-Keto Diesters. *Chem. Asian J.* **2018**, *13*, 2824-2828.

(14) Frisch, M. J.; Trucks, G. W.; Schlegel, H. B.; Scuseria, G. E.; Robb, M. A.; Cheeseman, J. R.; Scalmani, G.; Barone, V.; Mennucci, B.; Petersson, G. A.; Nakatsuji, H.; Caricato, M.; Li, X.; Hratchian, H. P.; Izmaylov, A. F.; Bloino, J.; Zheng, G.; Sonnenberg, J. L.; Hada, M.; Ehara, M.; Toyota, K.; Fukuda, R.; Hasegawa, J.; Ishida, M.; Nakajima, T.; Honda, Y.; Kitao, O.; Nakai, H.; Vreven, T.; Montgomery, J. A. Jr.; Peralta, J. E.; Ogliaro, F.; Bearpark, M.; Heyd, J. J.; Brothers, E.; Kudin, K. N.; Staroverov, V. N.; Kobayashi, R.; Normand, J.; Raghavachari, K.; Rendell, A.; Burant, J. C.; Iyengar, S. S.; Tomasi, J.; Cossi, M.; Rega, N.; Millam, N. J.; Klene, M.; Knox, J. E.; Cross, J. B.; Bakken, V.; Adamo, C.; Jaramillo, J.; Gomperts, R.; Stratmann, R. E.; Yazyev, O.; Austin, A. J.; Cammi, R.; Pomelli, C.; Ochterski, J. W.; Martin, R. L.; Morokuma, K.; Zakrzewski, V. G.; Voth, G. A.; Salvador, P.; Dannenberg, J. J.; Dapprich, S.; Daniels, A. D.; Farkas, Ö.; Foresman, J. B.; Ortiz, J. V.; Cioslowski, J.; Fox, D. J. *Gaussian 16, Revision A.03*; Gaussian, Inc., Wallingford, CT, 2016.

(15) Zhao, Y.; Truhlar, D. G. The M06 suite of density functionals for main group thermochemistry, thermochemical kinetics, noncovalent interactions, excited states, and transition elements: two new functionals and systematic testing of four M06-class functionals and 12 other functionals. *Theor. Chem. Acc.* **2008**, *120*, 215-241.

(16) Marenich, A. V.; Cramer, C. J.; Truhlar, D. G. Universal solvation model based on solute electron density and on a continuum model of the solvent defined by the bulk dielectric constant and atomic surface tensions. *J. Phys. Chem. B.* **2009**, *113*, 6378-6396.

J. Copies of NMR Spectroscopies

400 MHz ^{1}H NMR Spectrum of 1d in $CDCl_3$

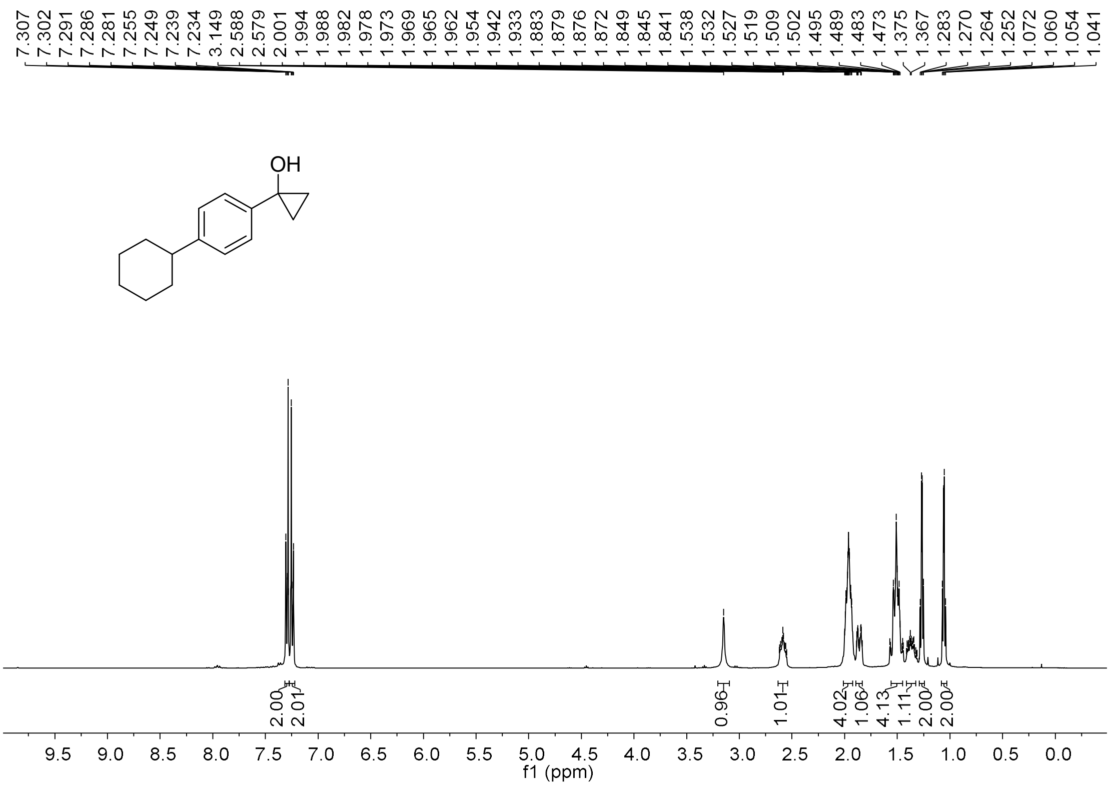

100 MHz ^{13}C{1H} NMR Spectrum of 1d in $CDCl_3$

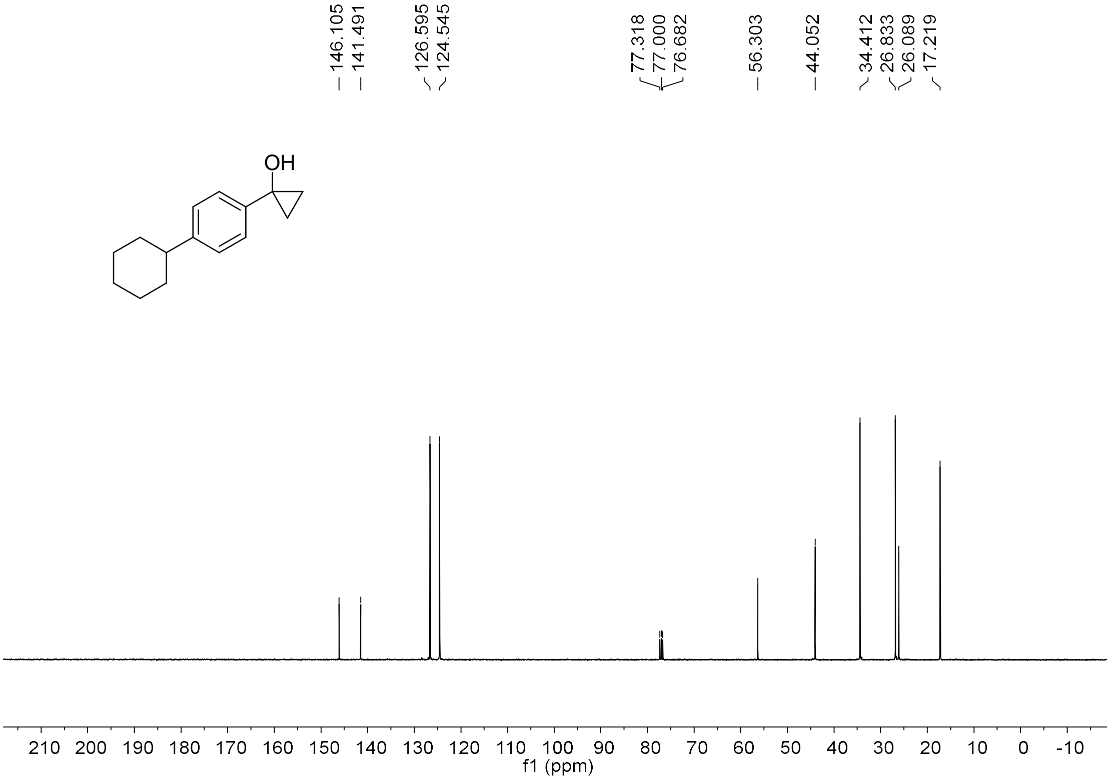

400 MHz ^{1}H NMR Spectrum of 1t in CDCl$_3$

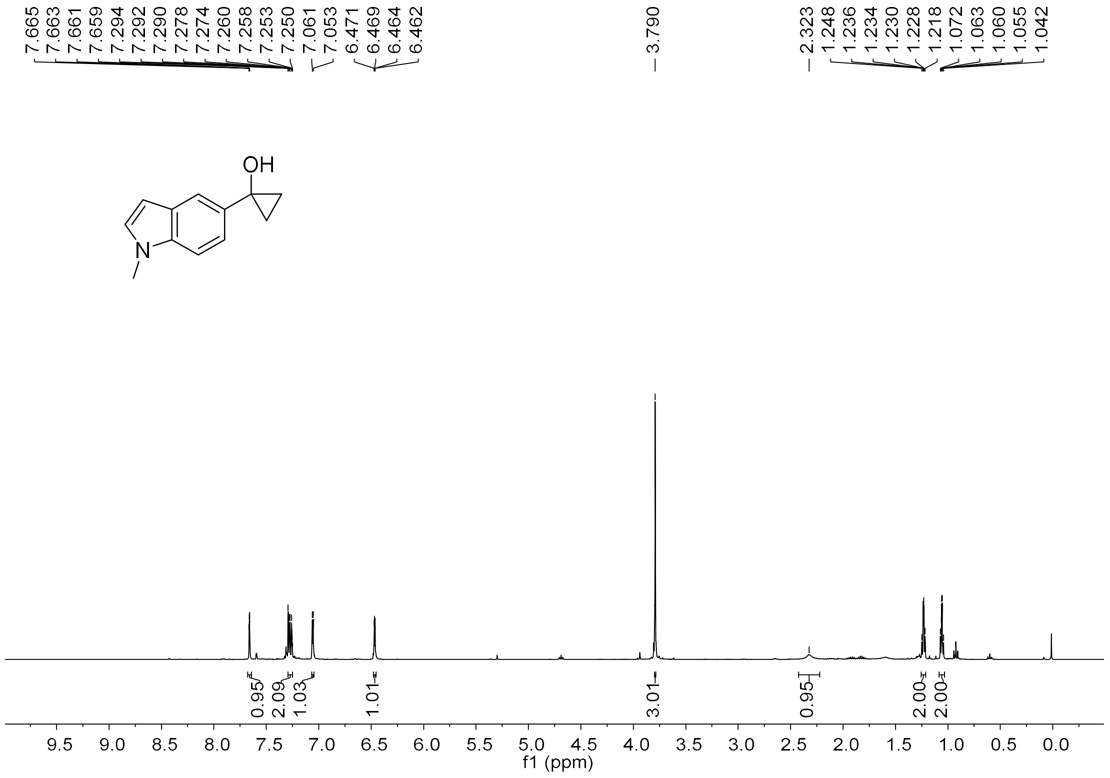

100 MHz ^{13}C{1H} NMR Spectrum of 1t in CDCl$_3$

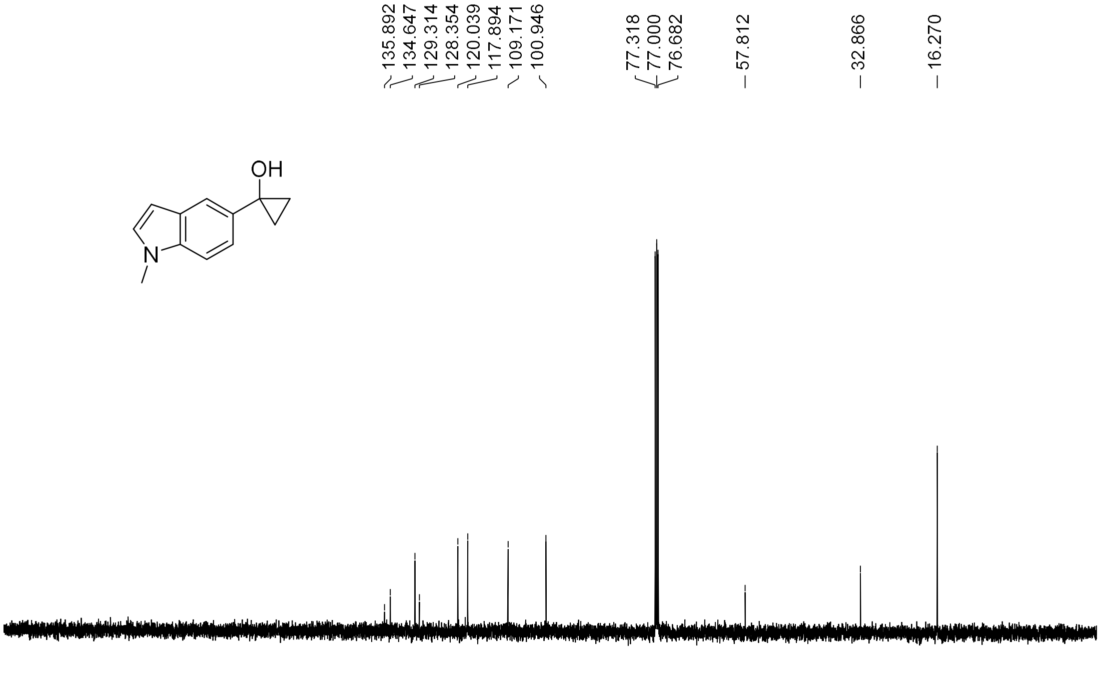

400 MHz ^{1}H NMR Spectrum of 1s in $CDCl_3$

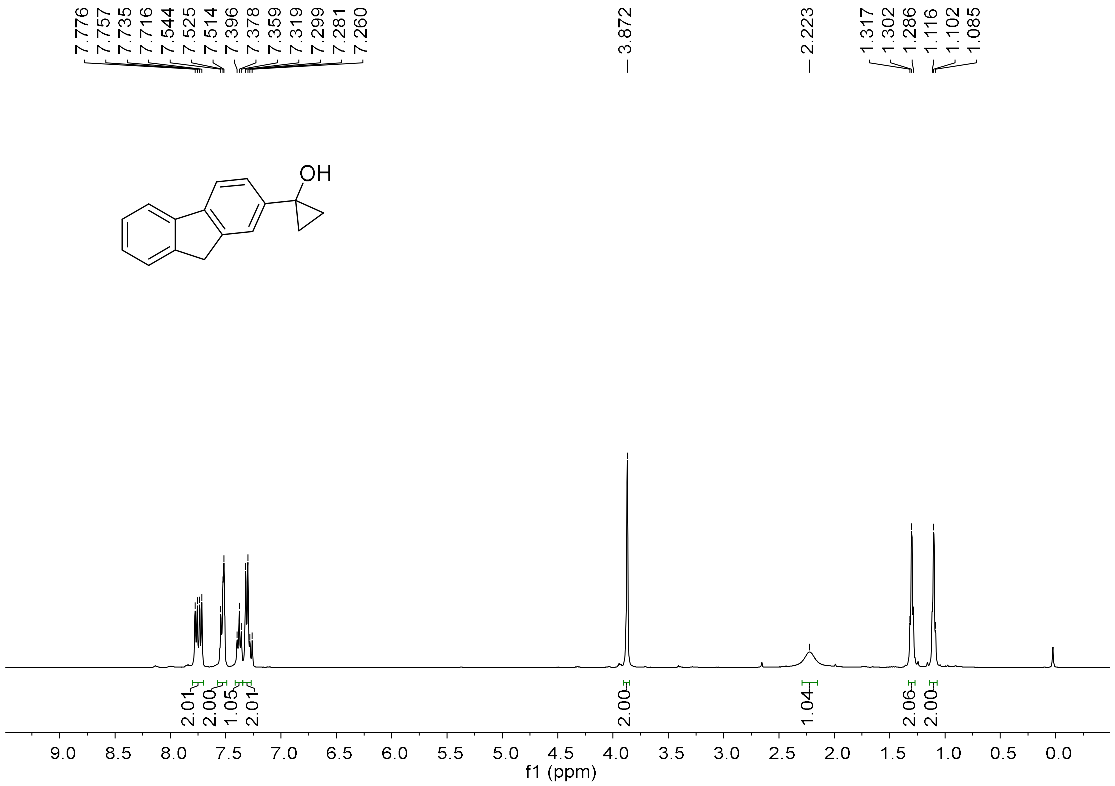

100 MHz $^{13}C\{1H\}$ NMR Spectrum of 1s in $CDCl_3$

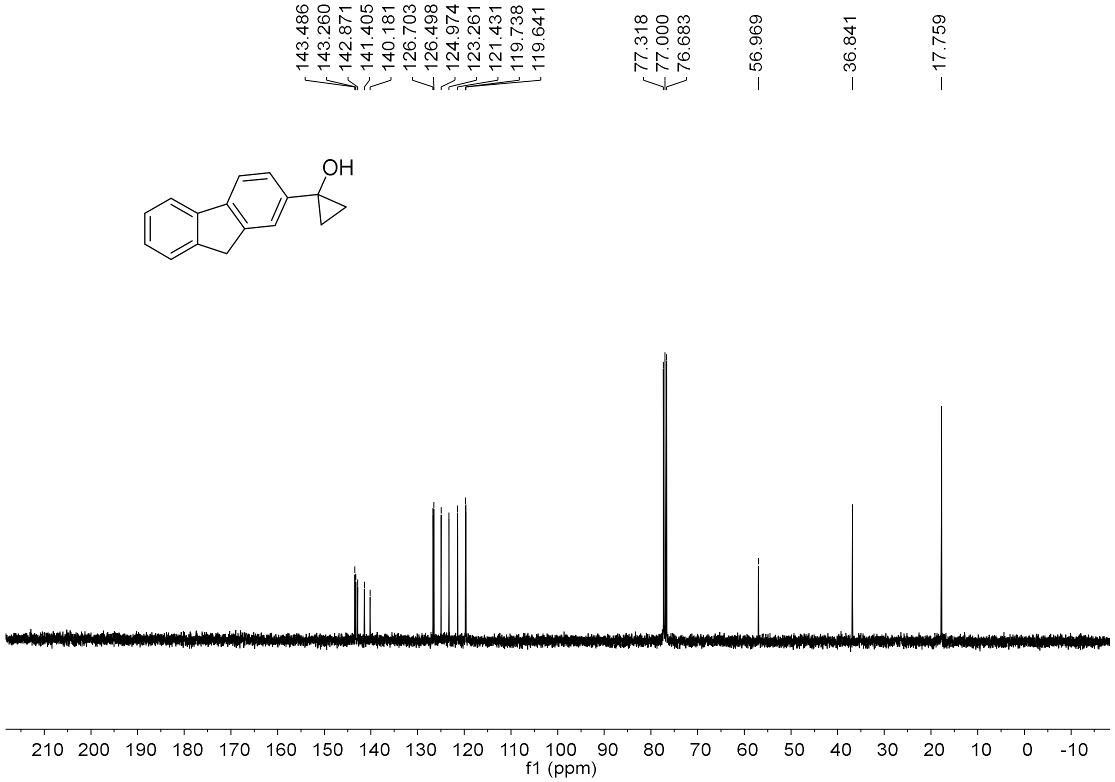

400 MHz ^{1}H NMR Spectrum of 1y in $CDCl_3$

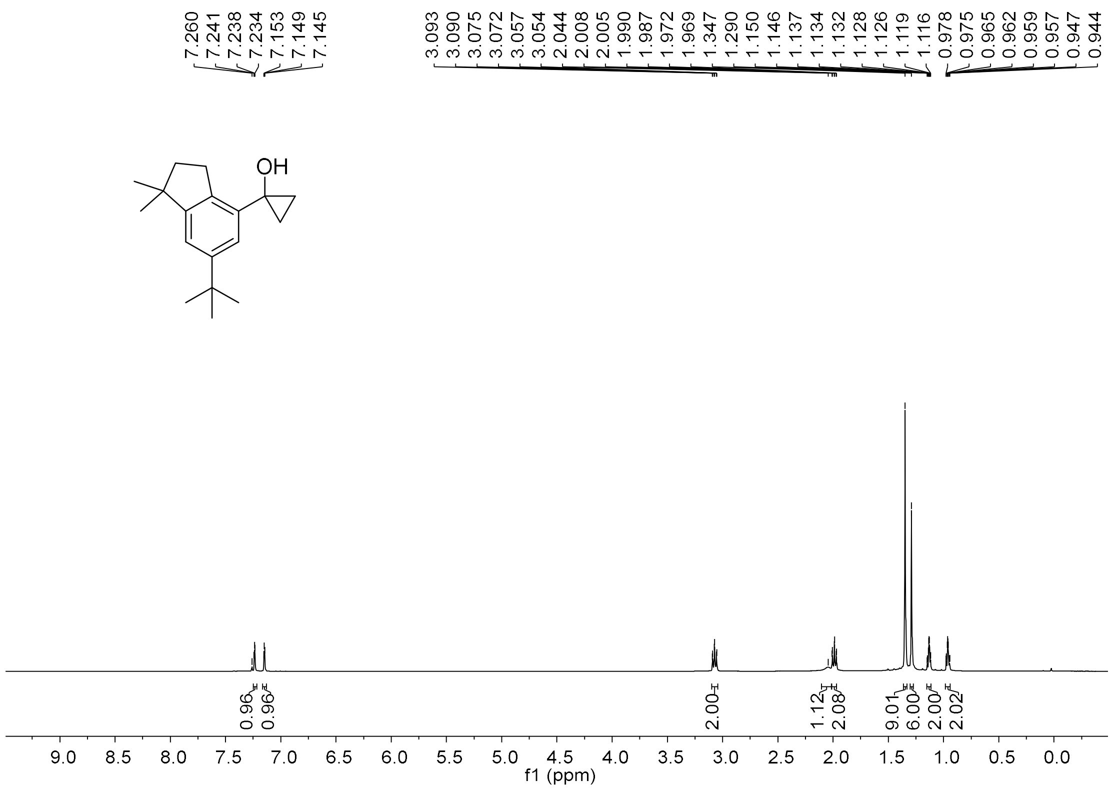

100 MHz $^{13}C\{1H\}$ NMR Spectrum of 1y in $CDCl_3$

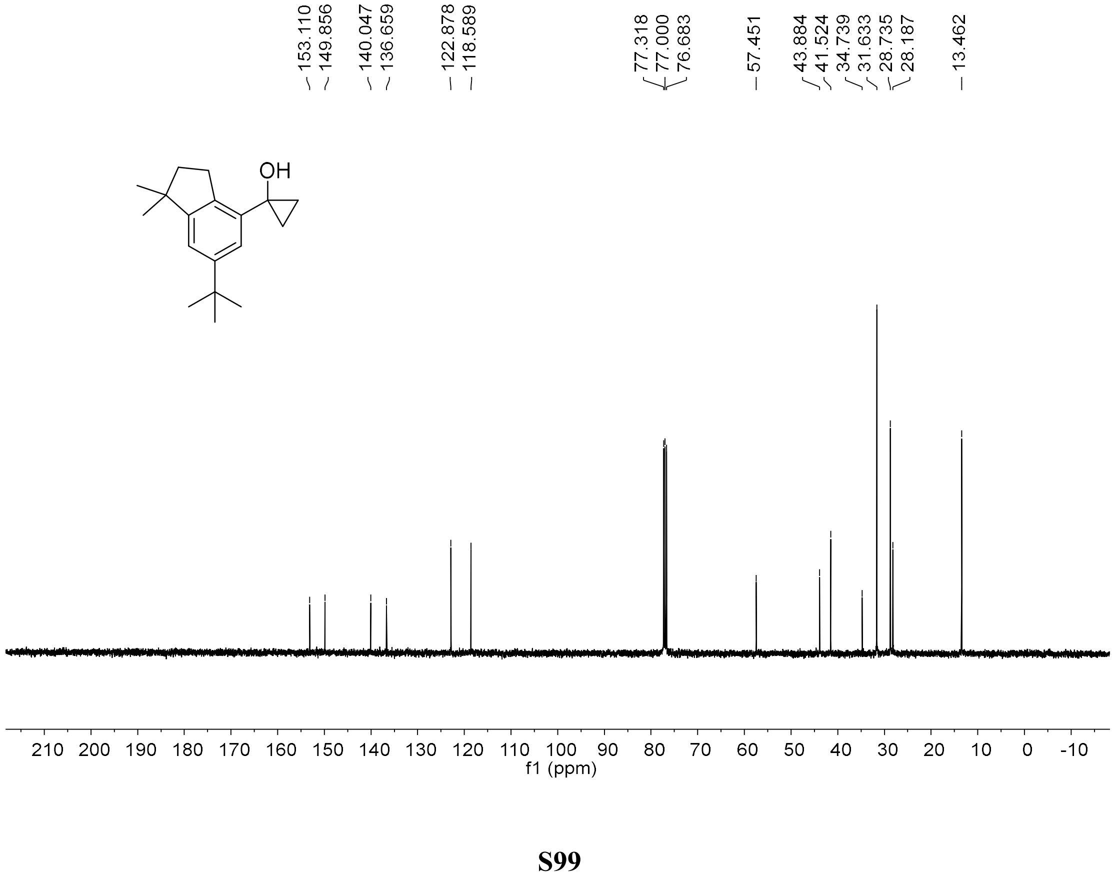

400 MHz ¹H NMR Spectrum of 1z in CDCl₃

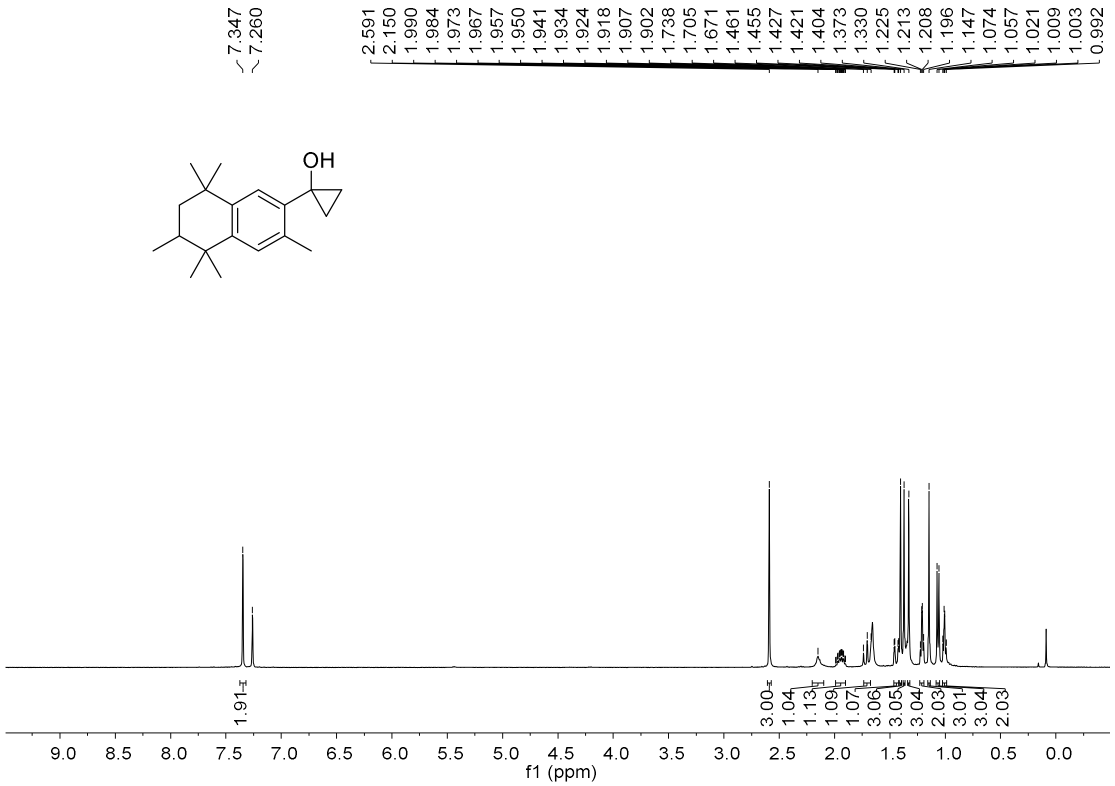

100 MHz ¹³C{1H} NMR Spectrum of 1z in CDCl₃

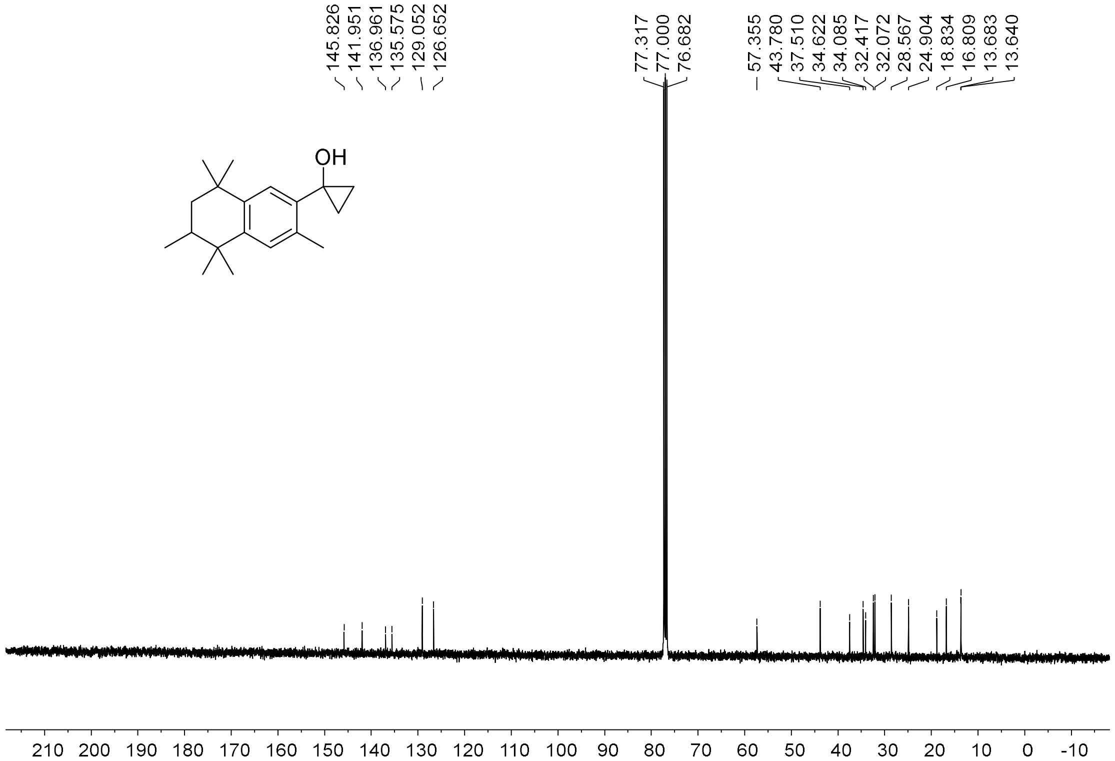

400 MHz ^{1}H NMR Spectrum of 1ab in CDCl$_3$

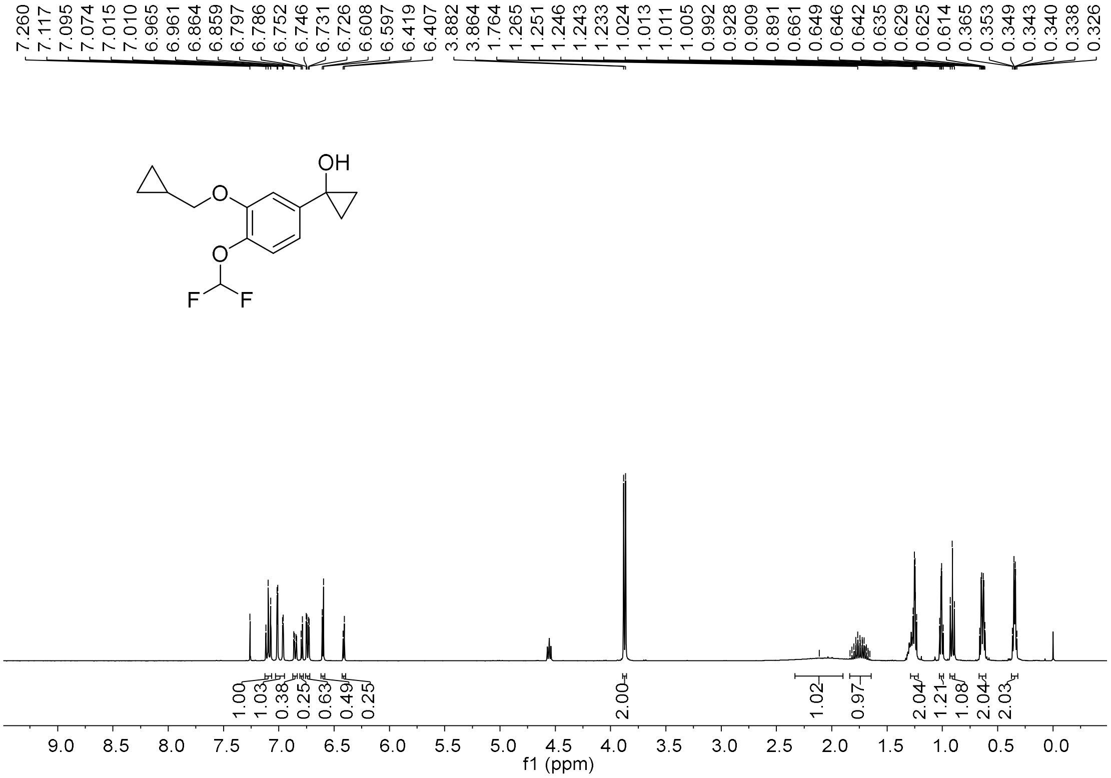

100 MHz ^{13}C{1H} NMR Spectrum of 1ab in CDCl$_3$

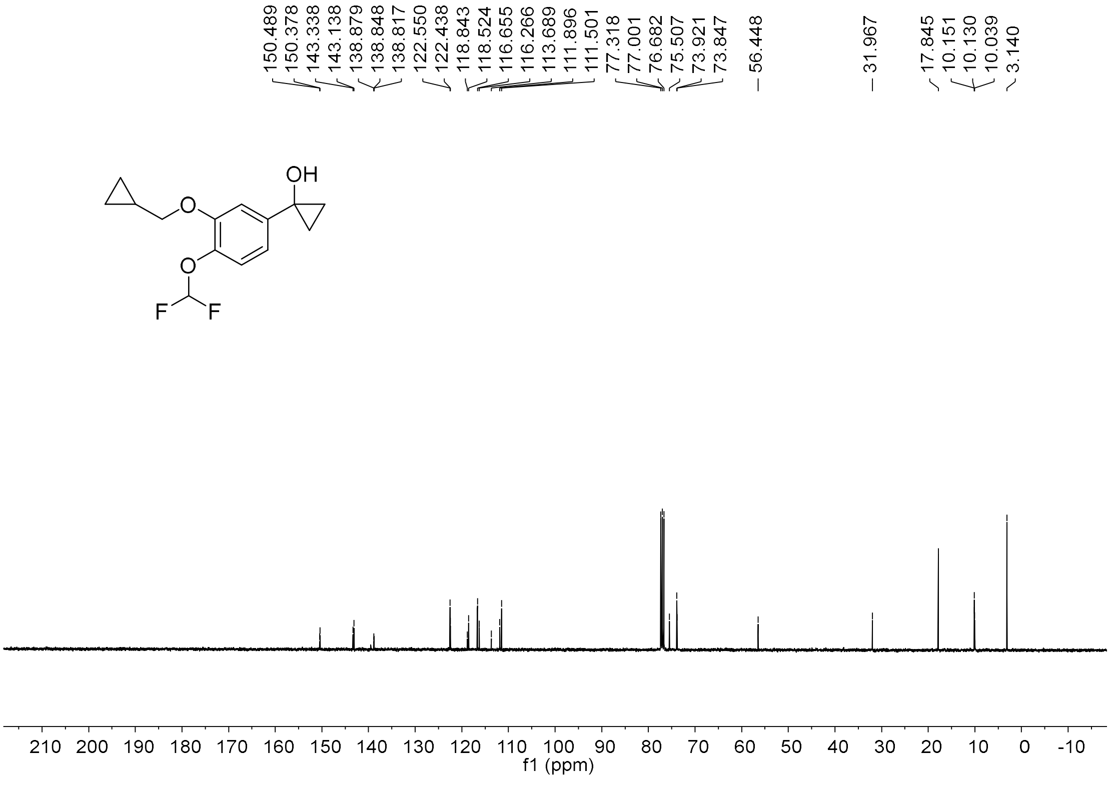

400 MHz ^{1}H NMR Spectrum of 1ad in CDCl$_3$

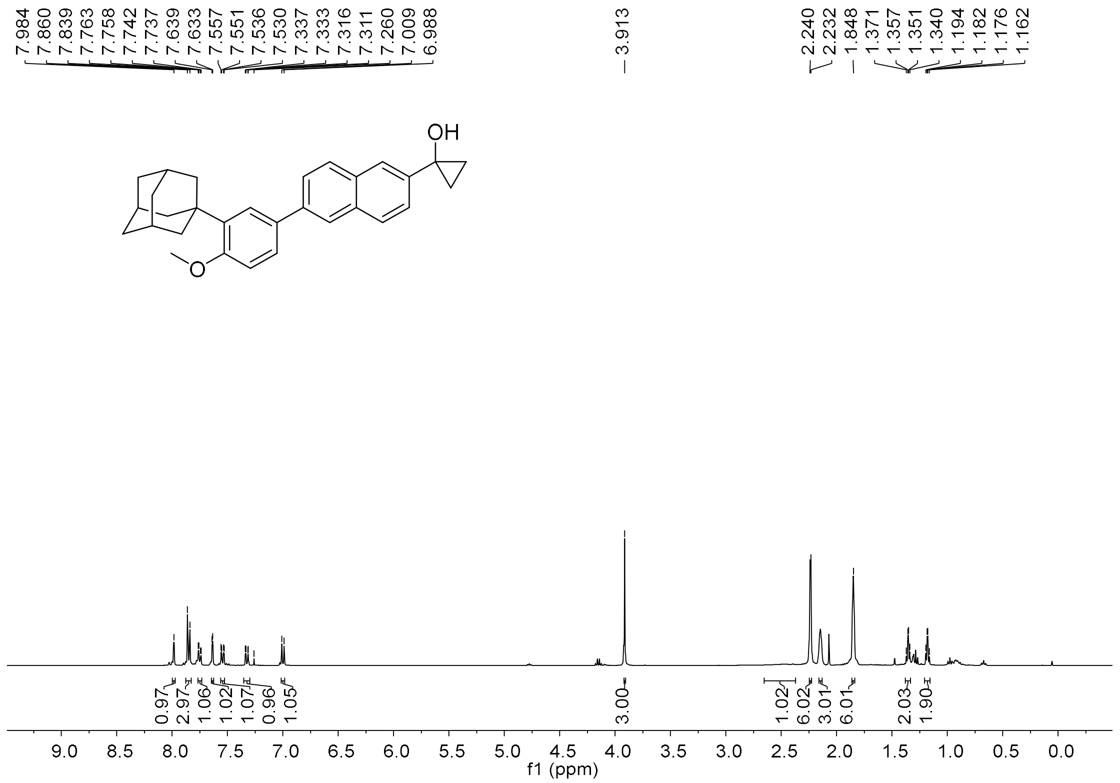

100 MHz ^{13}C{1H} NMR Spectrum of 1ad in CDCl$_3$

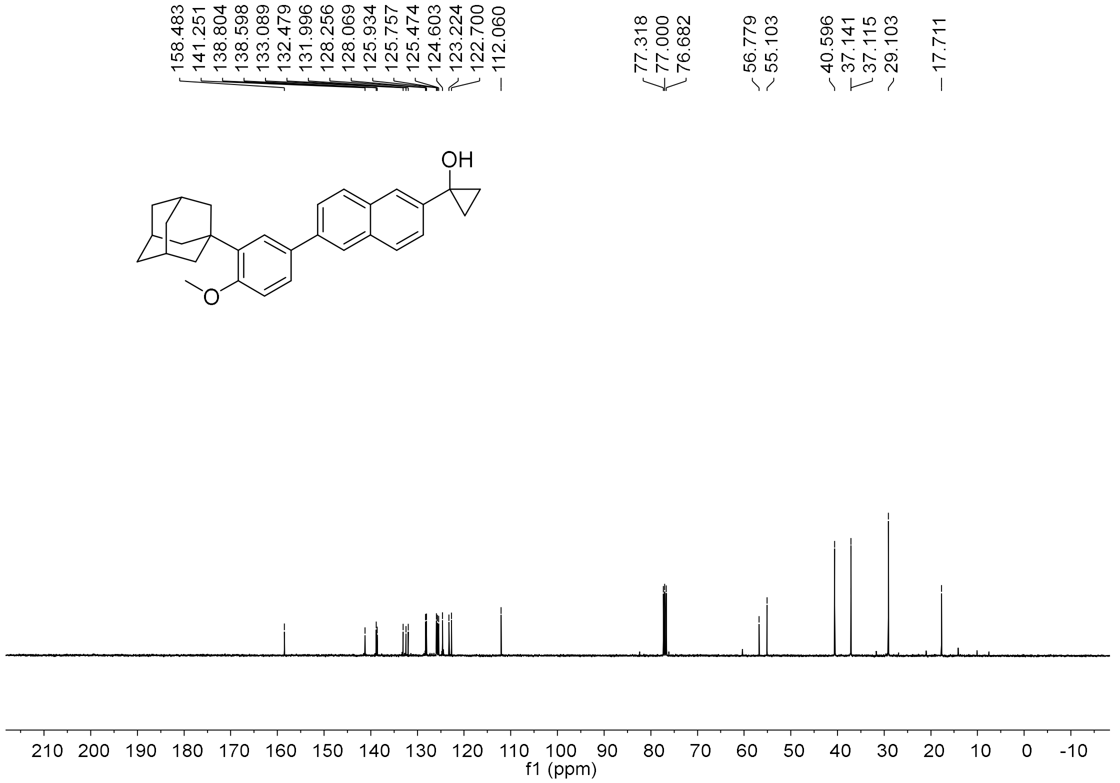

400 MHz ^{1}H NMR Spectrum of 1at in $CDCl_3$

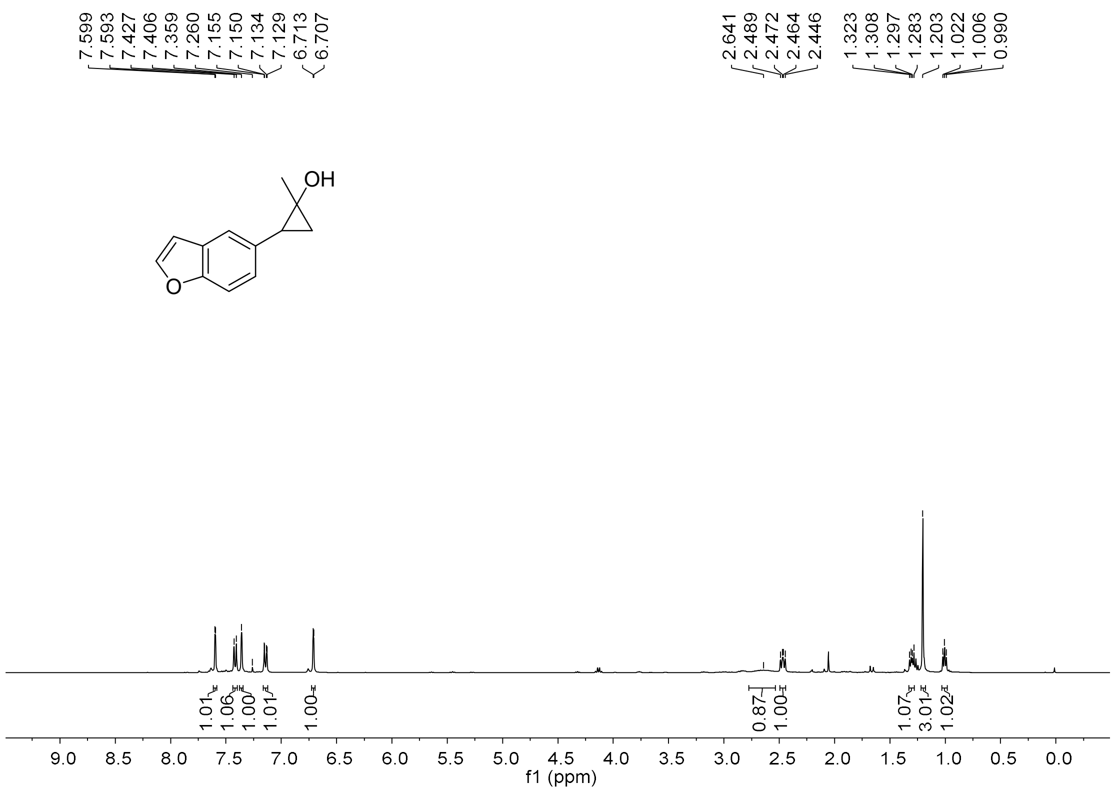

100 MHz ^{13}C{1H} NMR Spectrum of 1at in $CDCl_3$

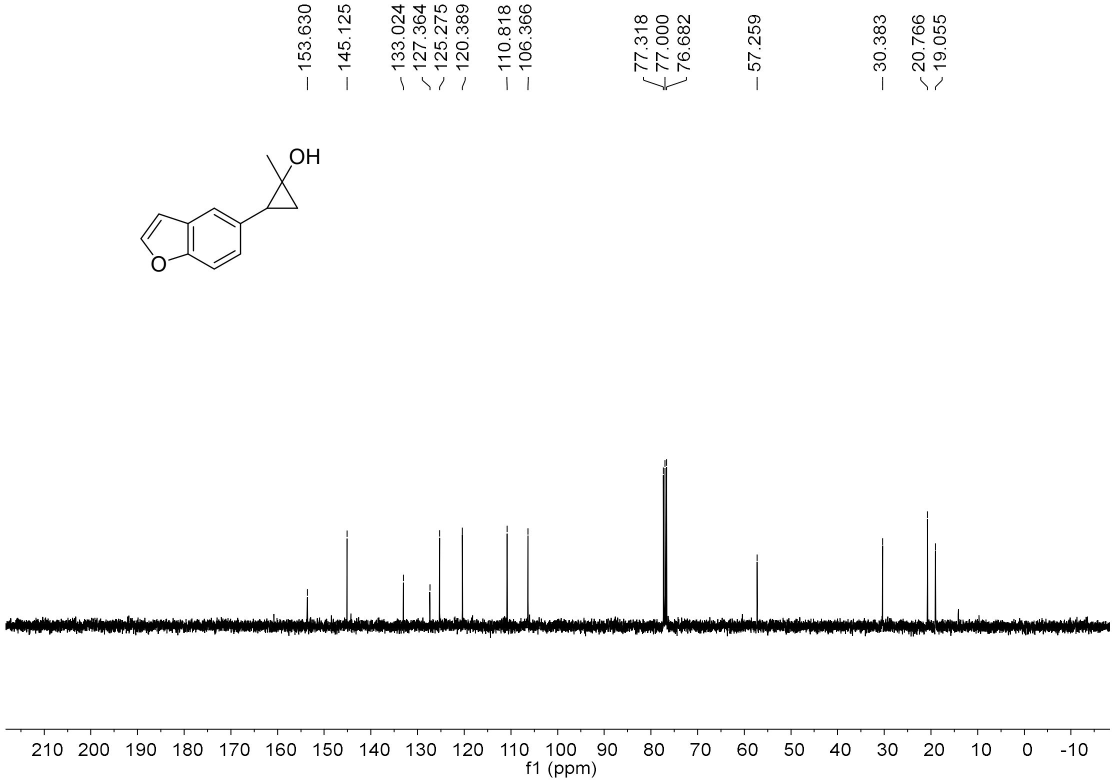

400 MHz ^{1}H NMR Spectrum of 2 in DMSO-d_6

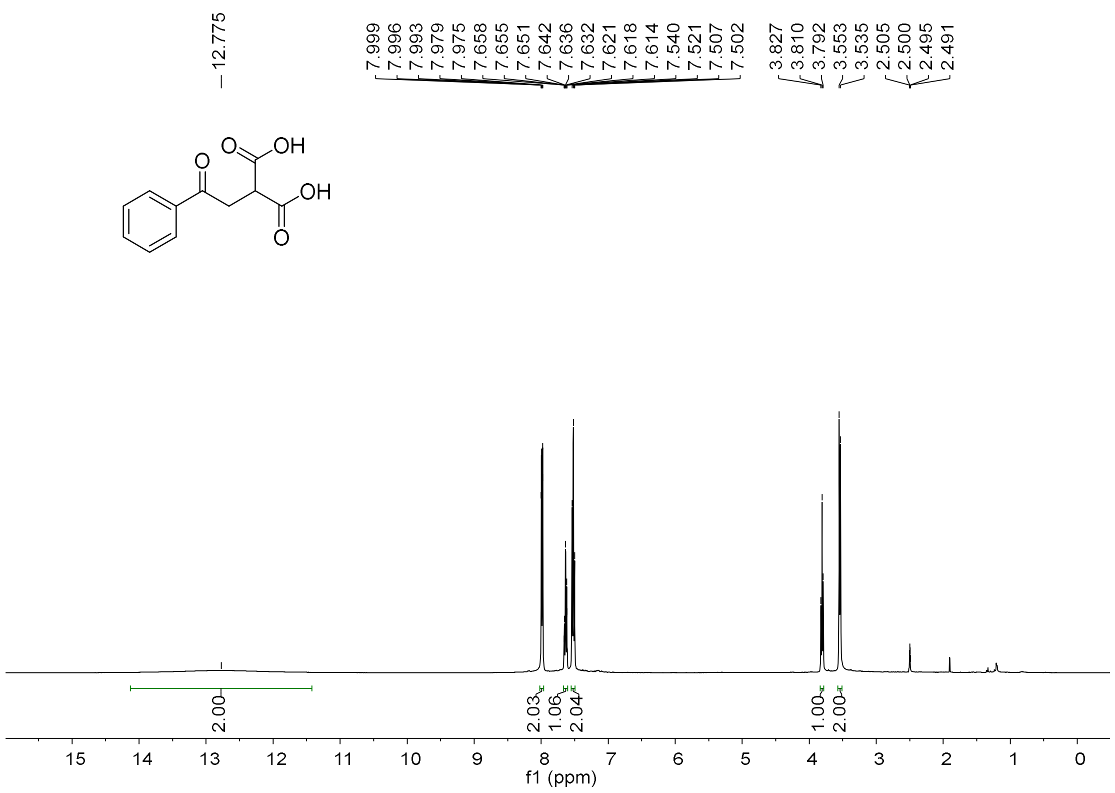

100 MHz ^{13}C{1H} NMR Spectrum of 2 in DMSO-d_6

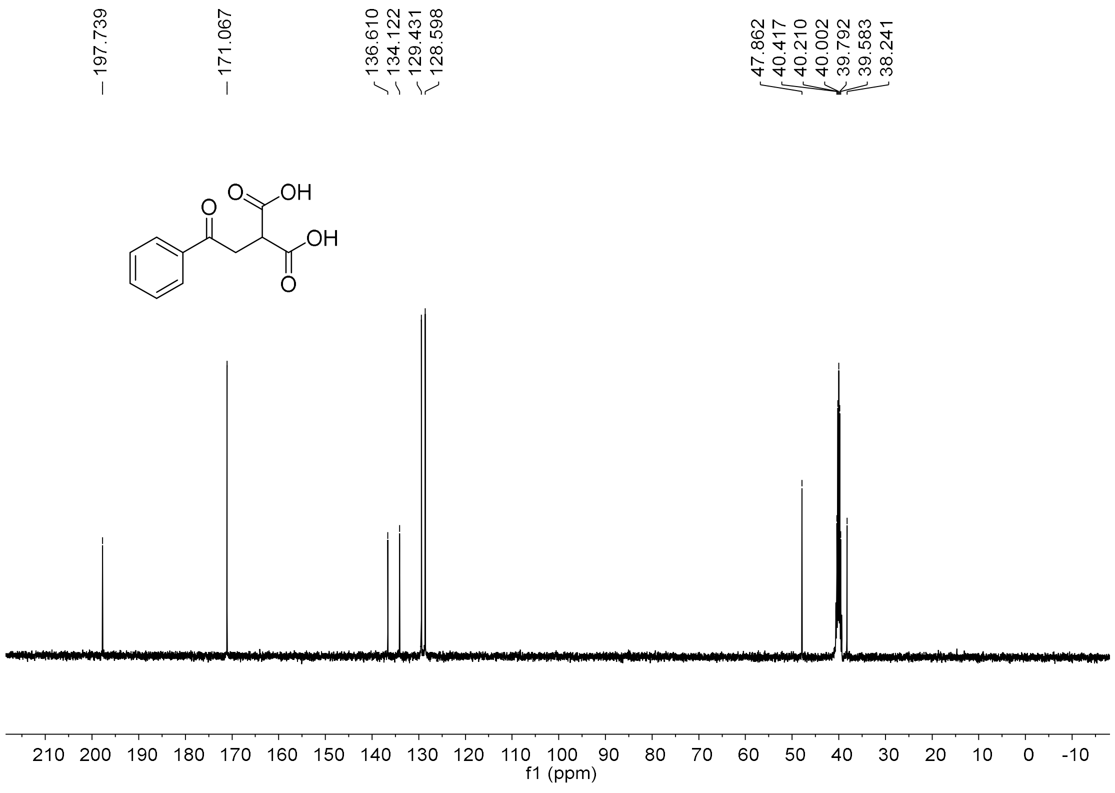

400 MHz ¹H NMR Spectrum of 3 in DMSO-d_6

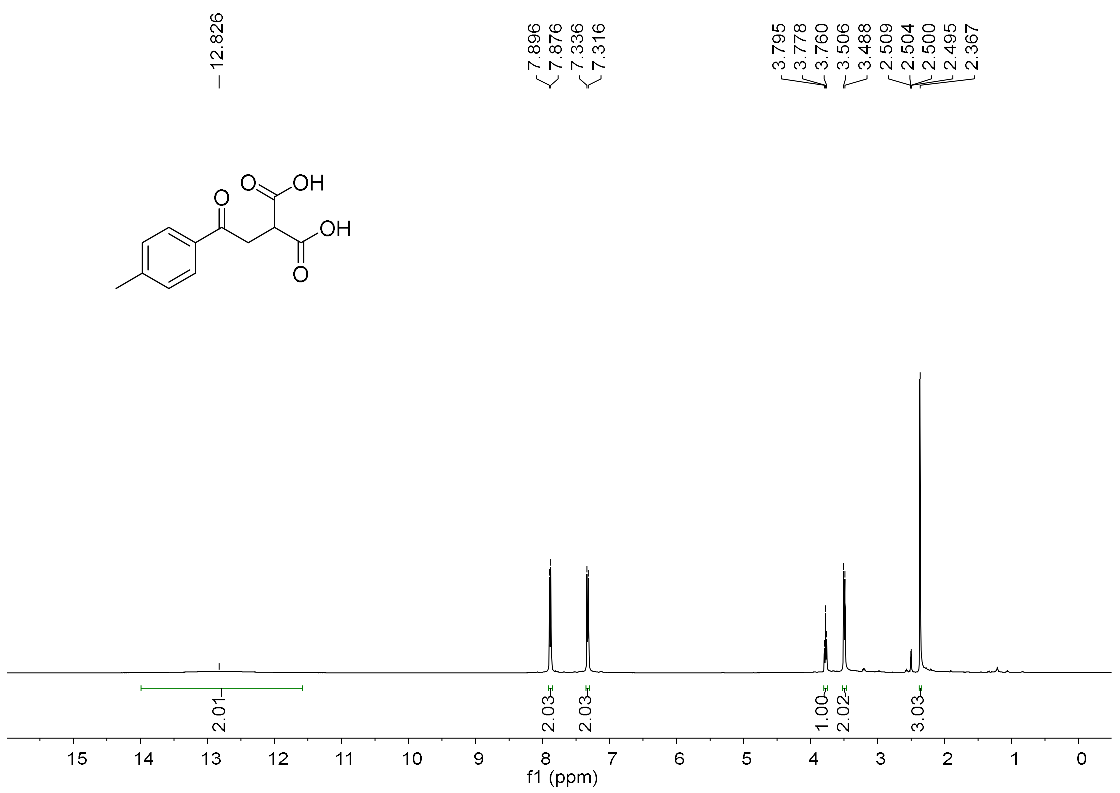

100 MHz ¹³C{1H} NMR Spectrum of 3 in DMSO-d_6

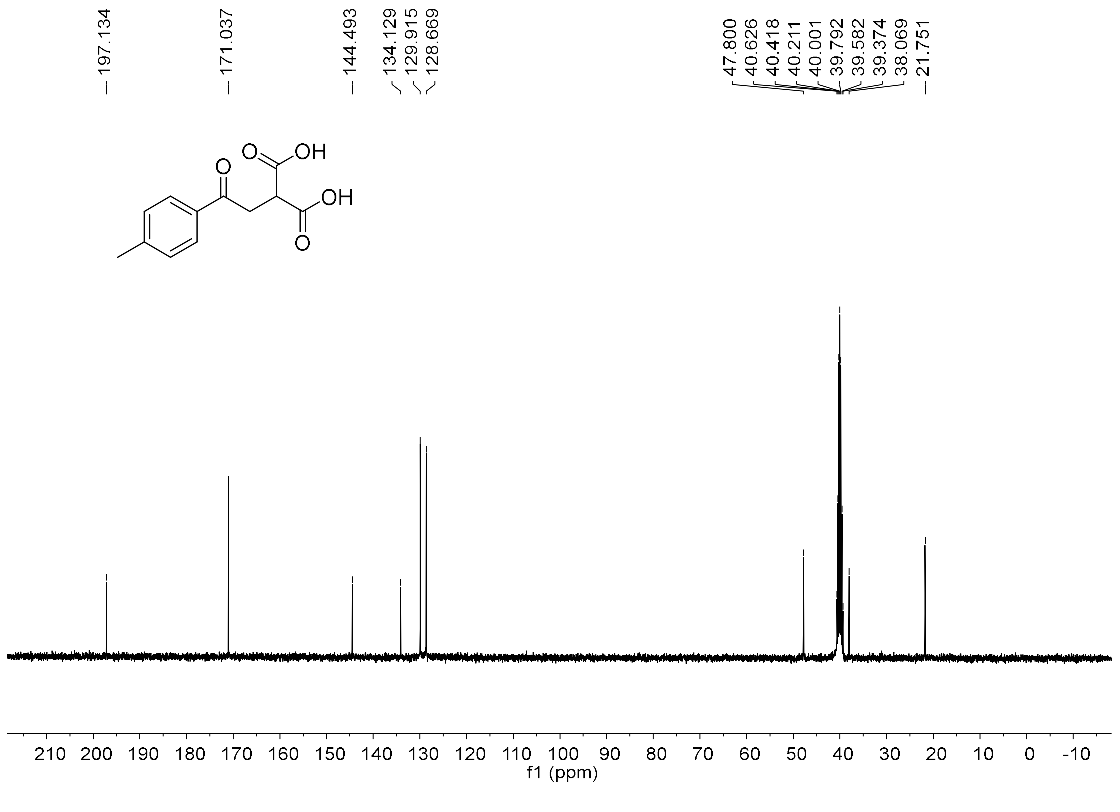

400 MHz ^{1}H NMR Spectrum of 4 in DMSO-d_6

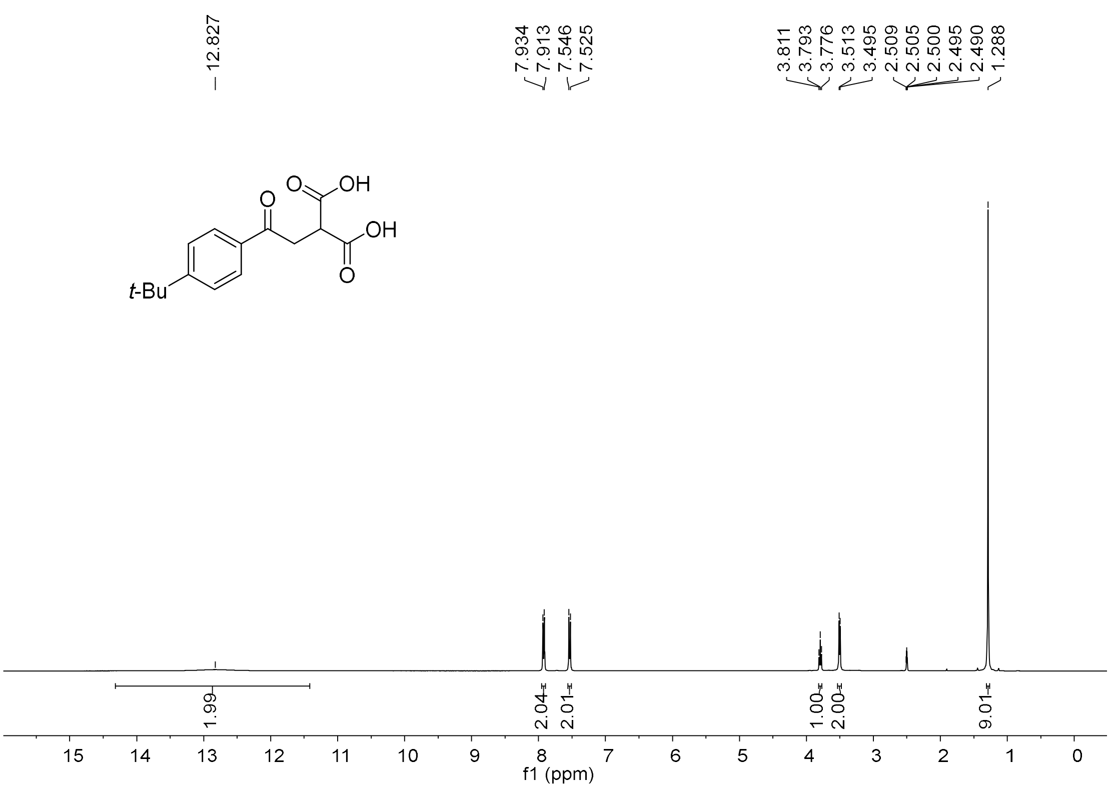

100 MHz ^{13}C{1H} NMR Spectrum of 4 in DMSO-d_6

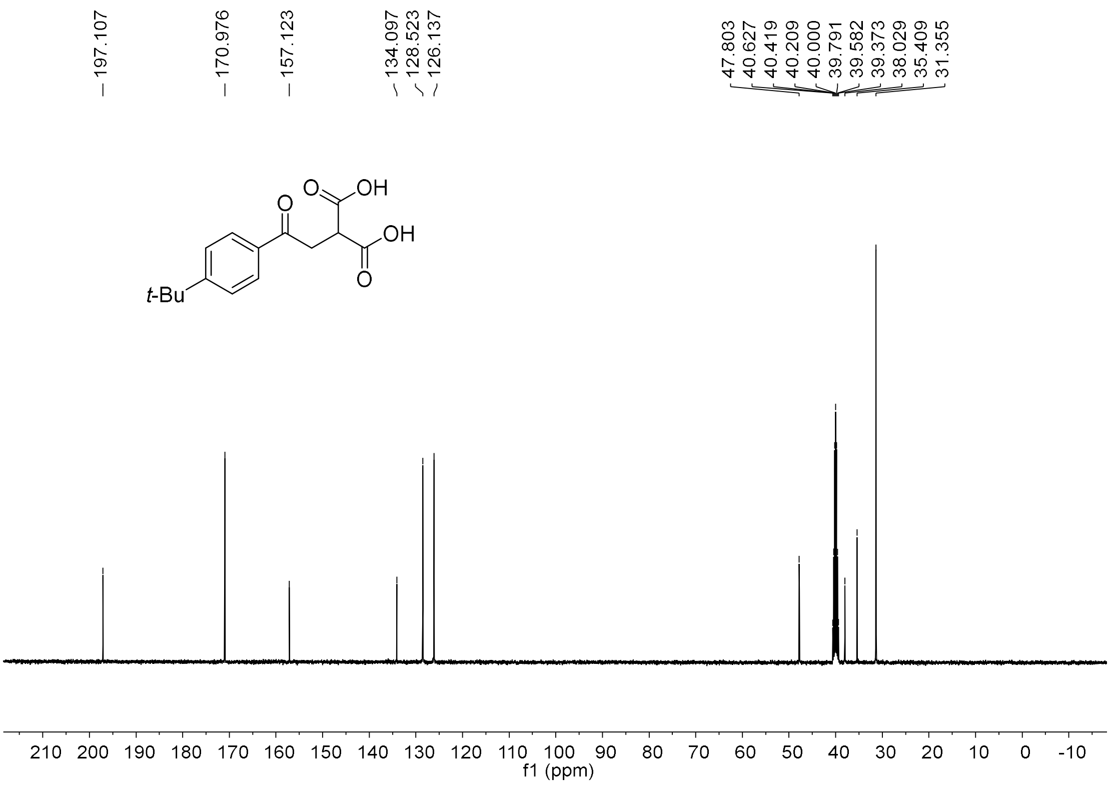

400 MHz ^{1}H NMR Spectrum of 5 in DMSO-d_6

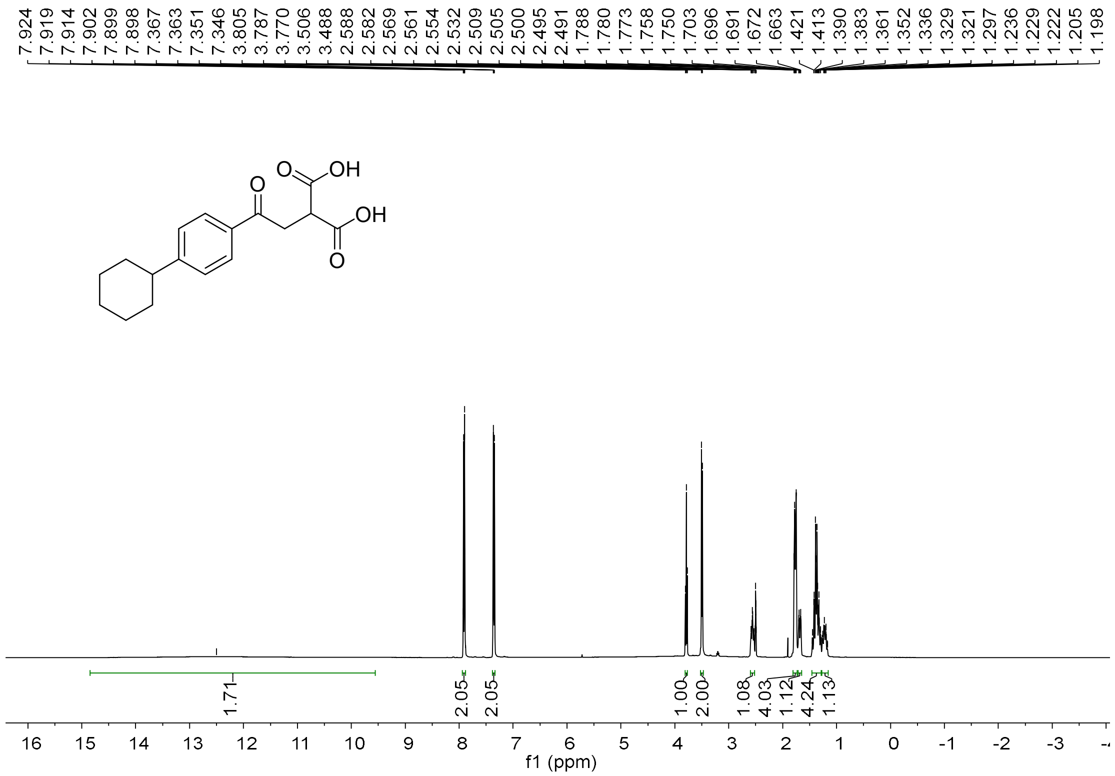

100 MHz ^{13}C{1H} NMR Spectrum of 5 in DMSO-d_6

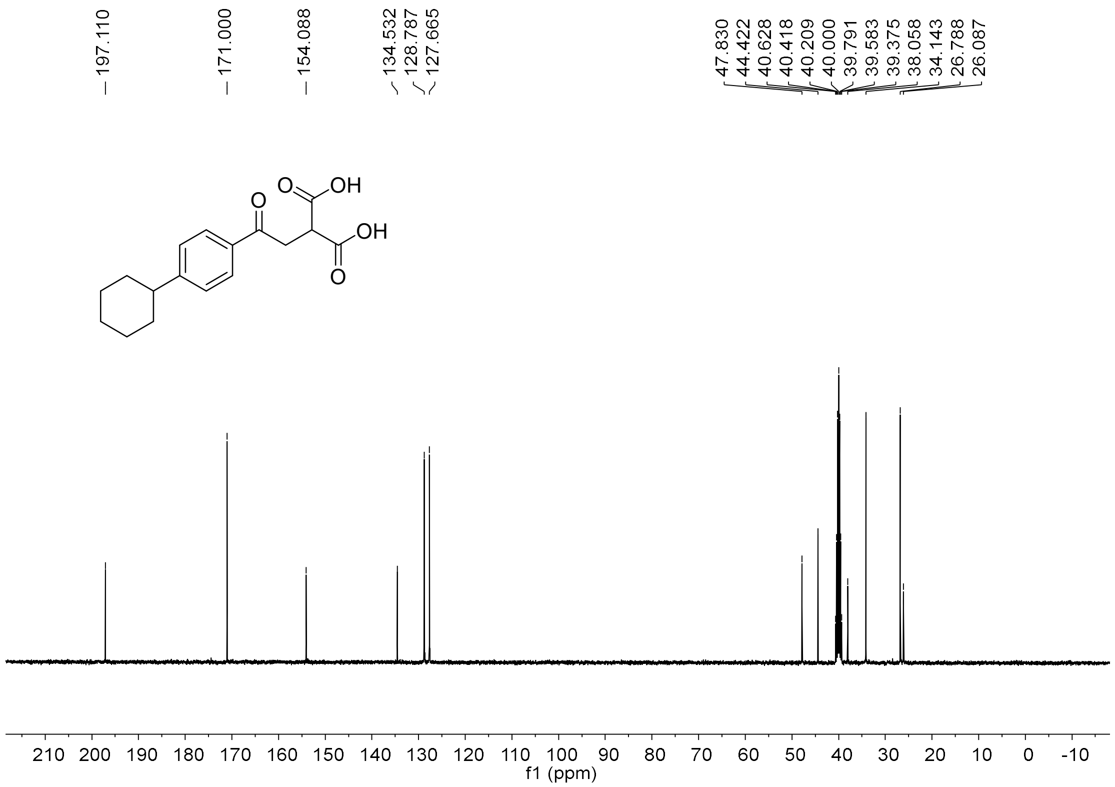

400 MHz ^{1}H NMR Spectrum of 6 in DMSO-d_6

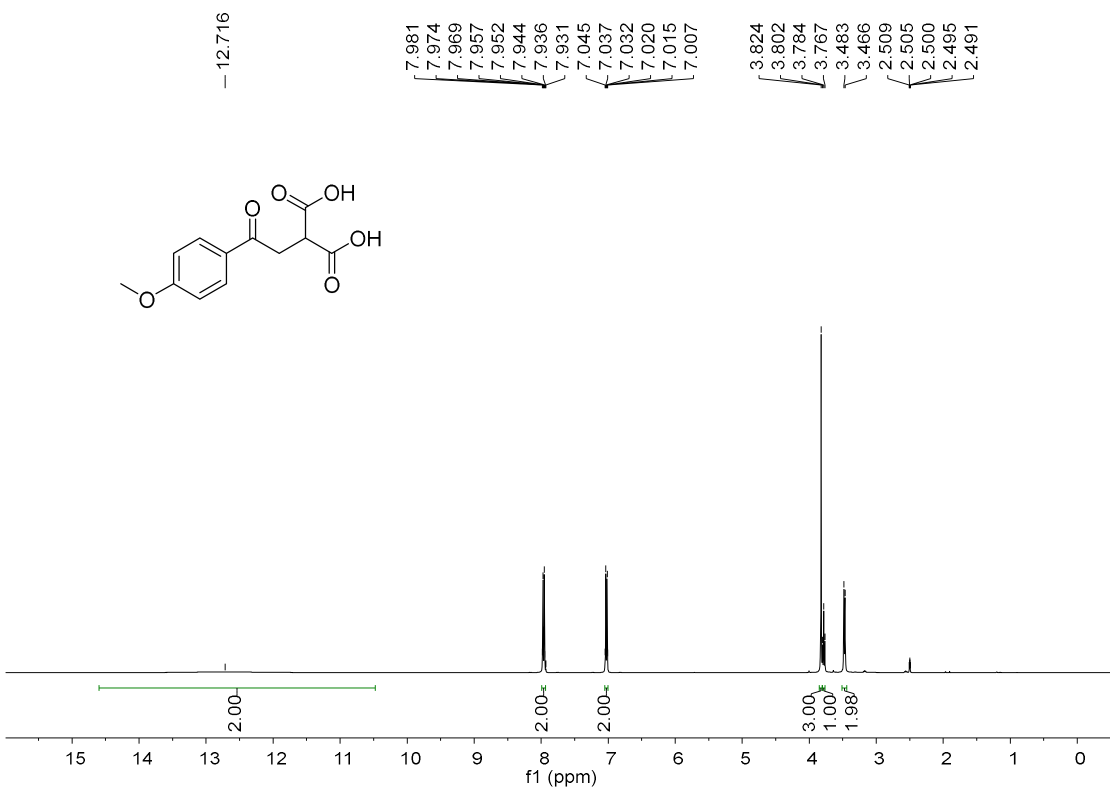

100 MHz $^{13}C\{1H\}$ NMR Spectrum of 6 in DMSO-d_6

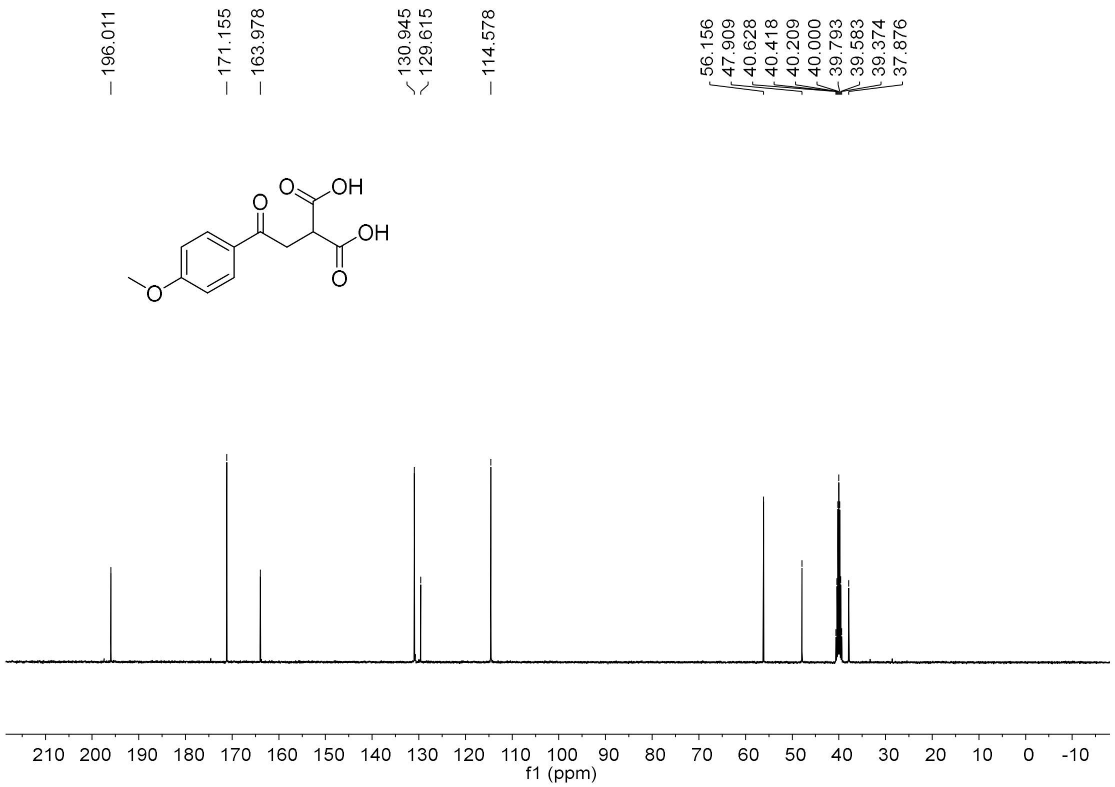

400 MHz ^{1}H NMR Spectrum of 7 in DMSO-d_6

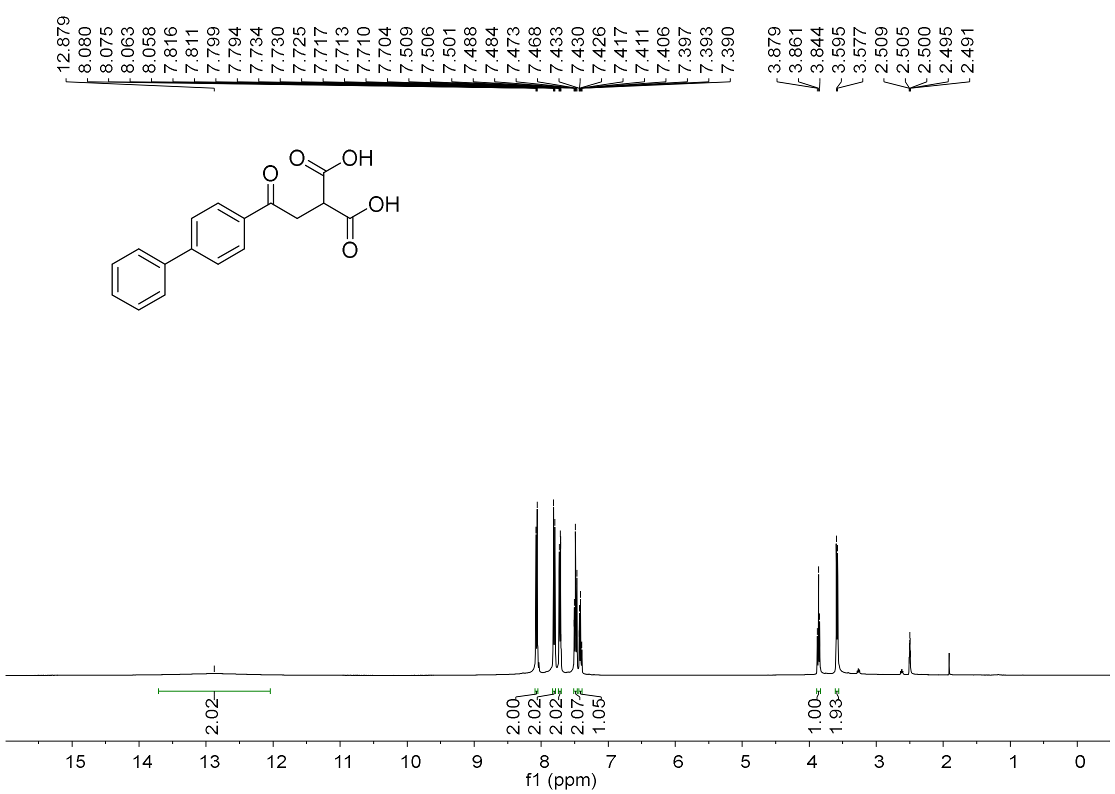

100 MHz ^{13}C{1H} NMR Spectrum of 7 in DMSO-d_6

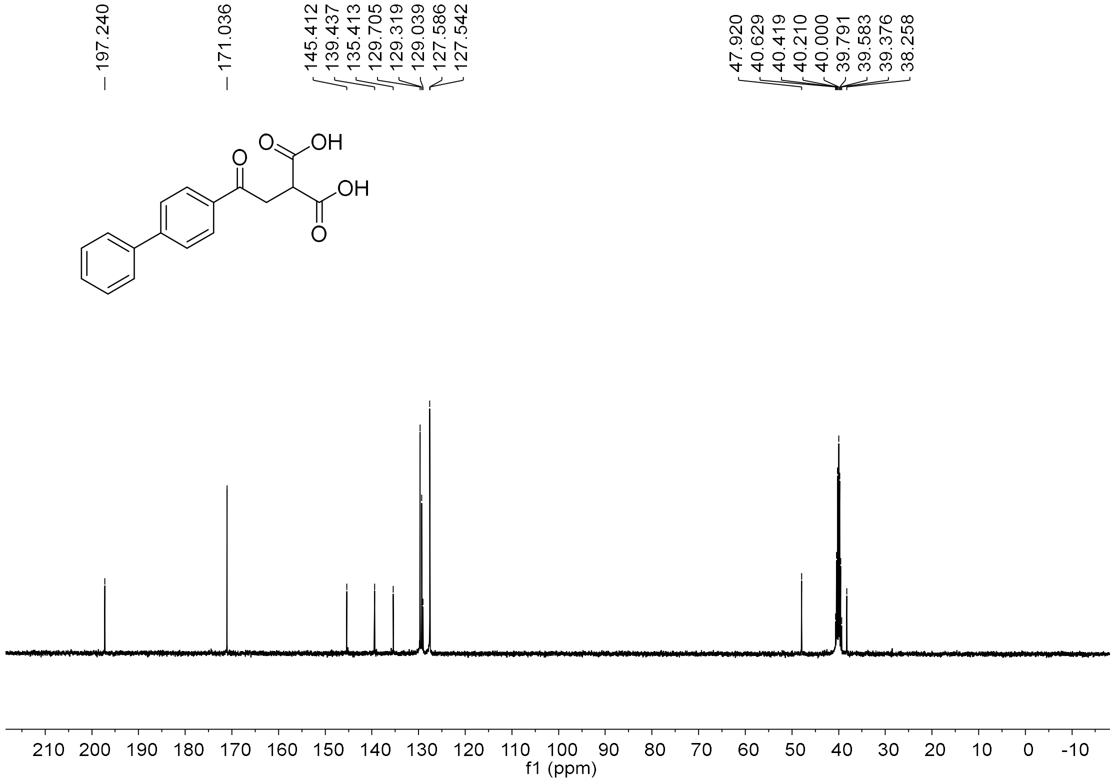

400 MHz ^{1}H NMR Spectrum of 8 in DMSO-d_6

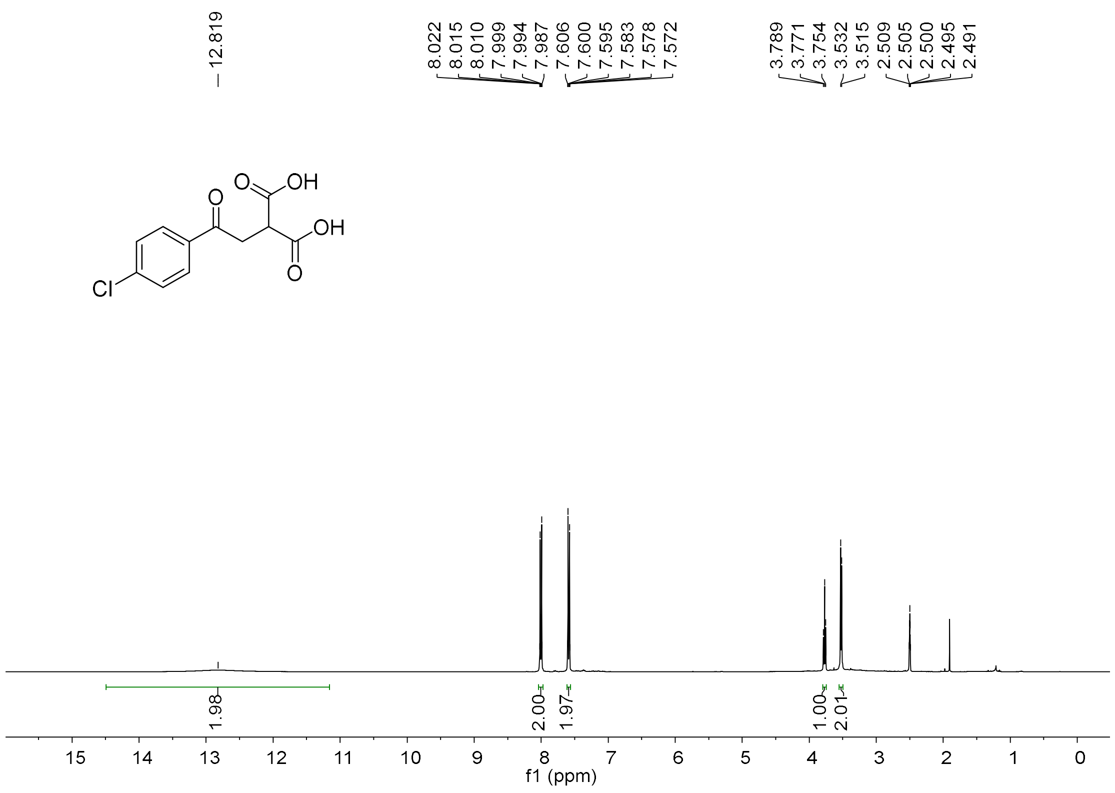

100 MHz ^{13}C{1H} NMR Spectrum of 8 in DMSO-d_6

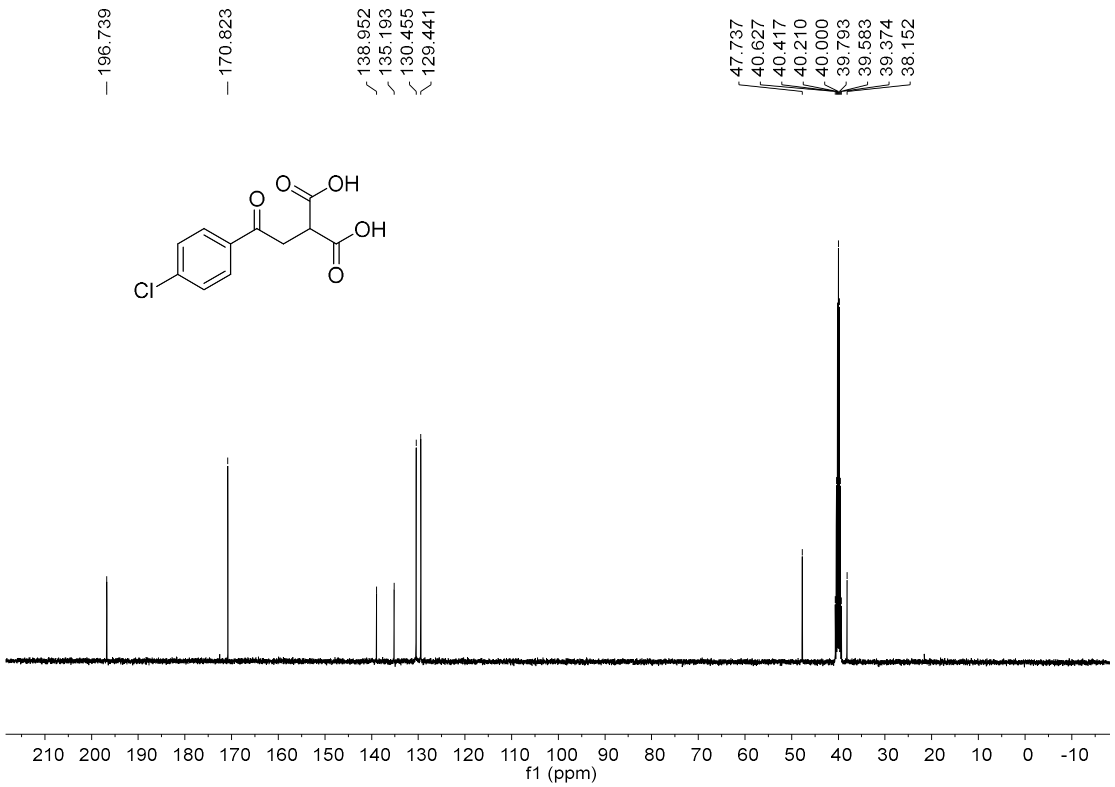

400 MHz ^{1}H NMR Spectrum of 9 in DMSO-d_6

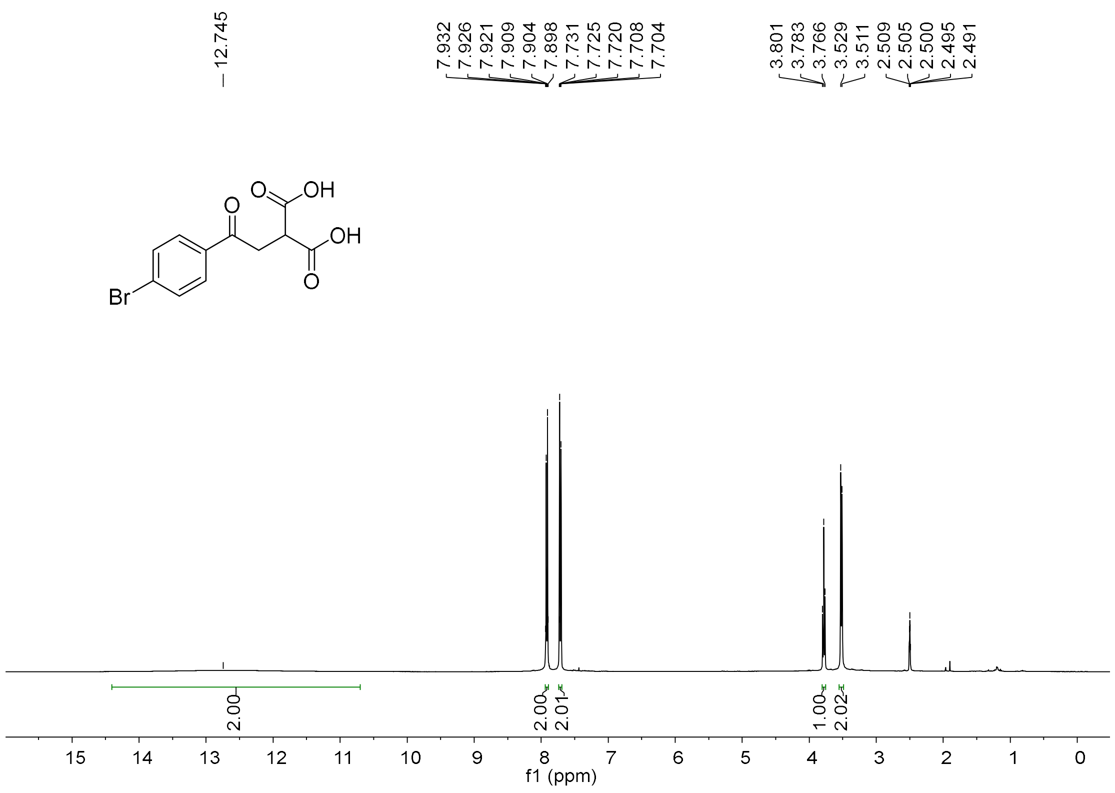

100 MHz ^{13}C{1H} NMR Spectrum of 9 in DMSO-d_6

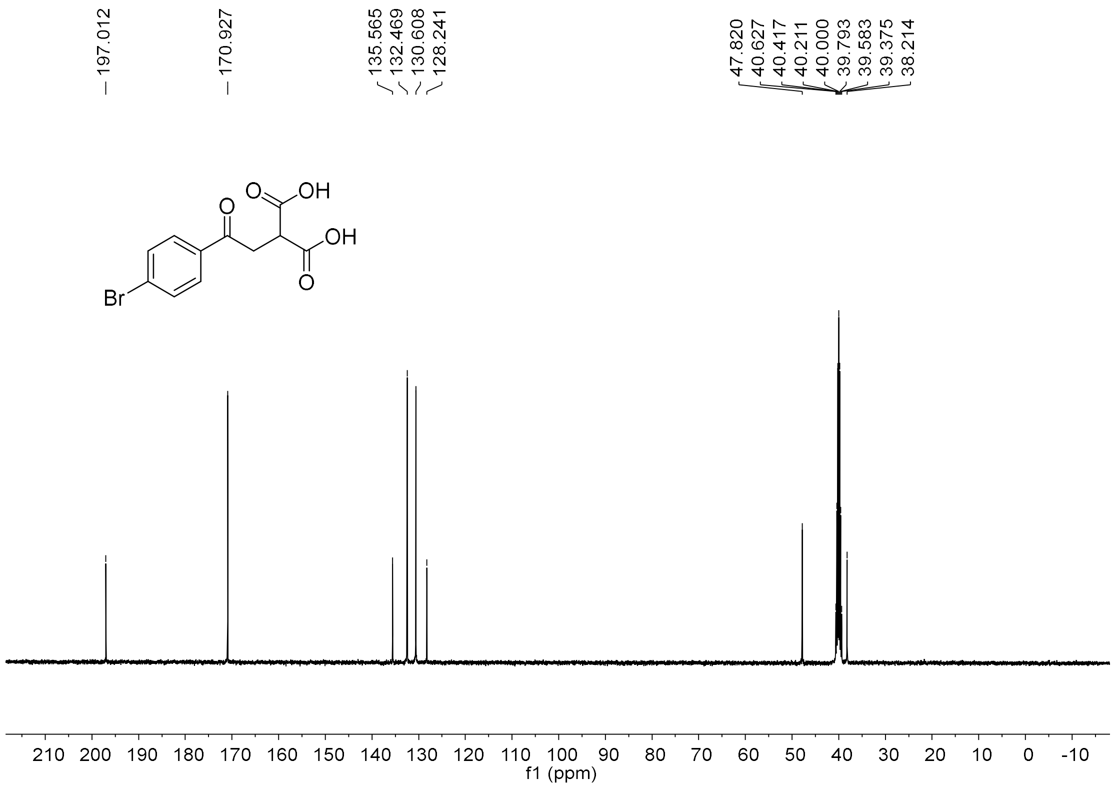

400 MHz ^{1}H NMR Spectrum of 10 in DMSO-d_6

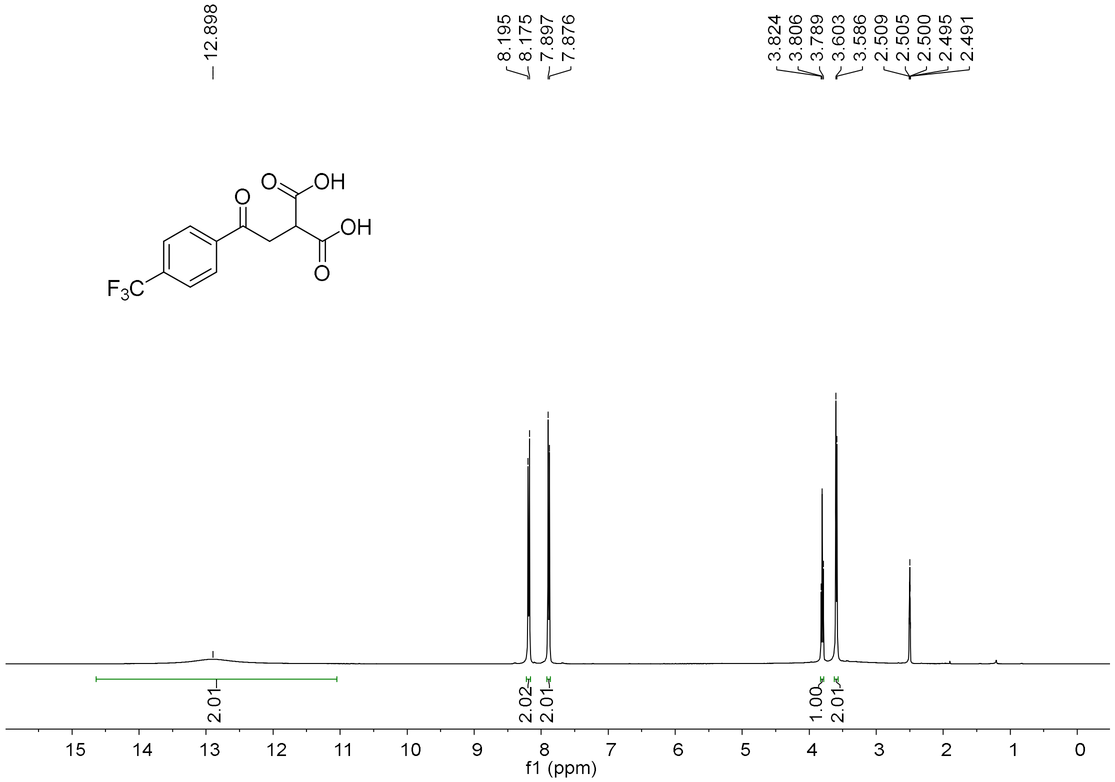

100 MHz ^{13}C{1H} NMR Spectrum of 10 in DMSO-d_6

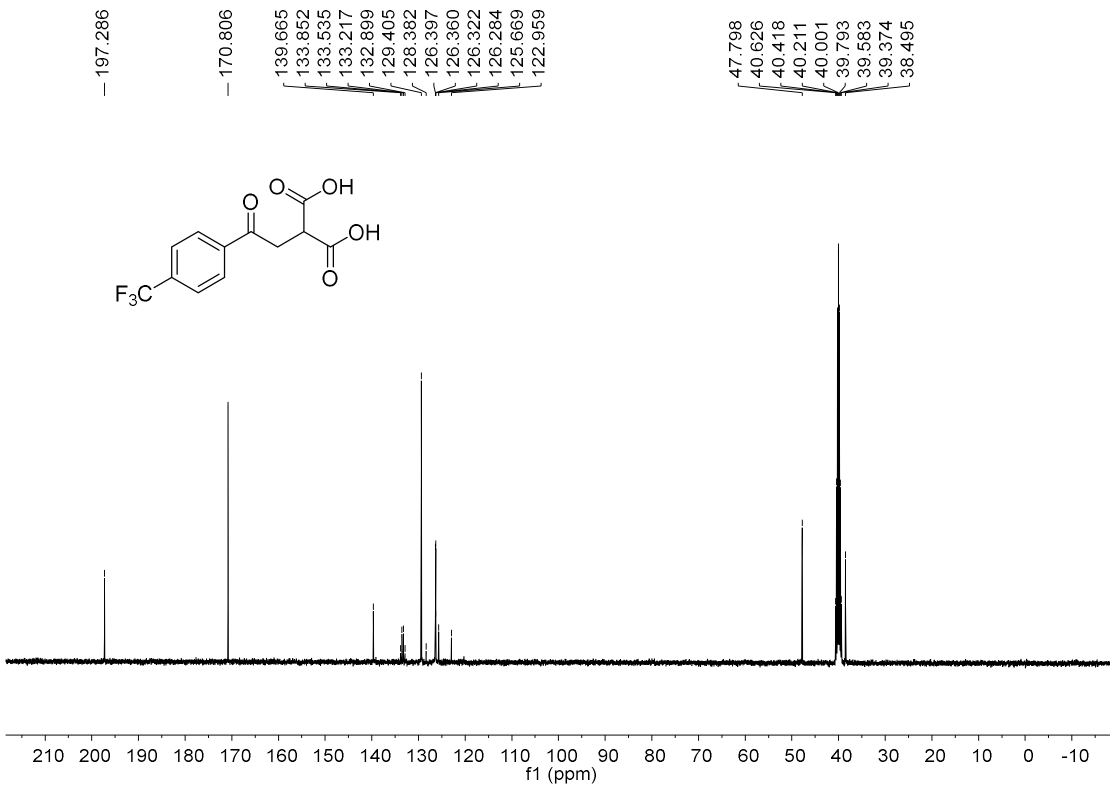

400 MHz ^{1}H NMR Spectrum of 11 in DMSO-d_6

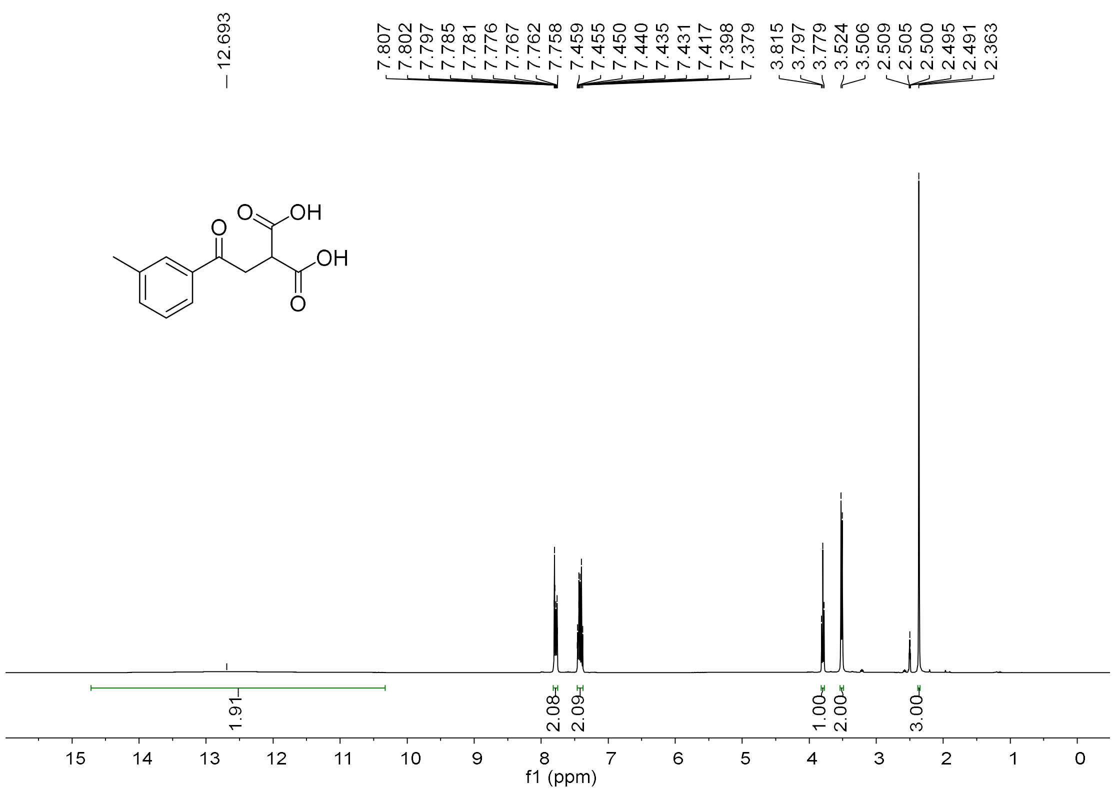

100 MHz ^{13}C{1H} NMR Spectrum of 11 in DMSO-d_6

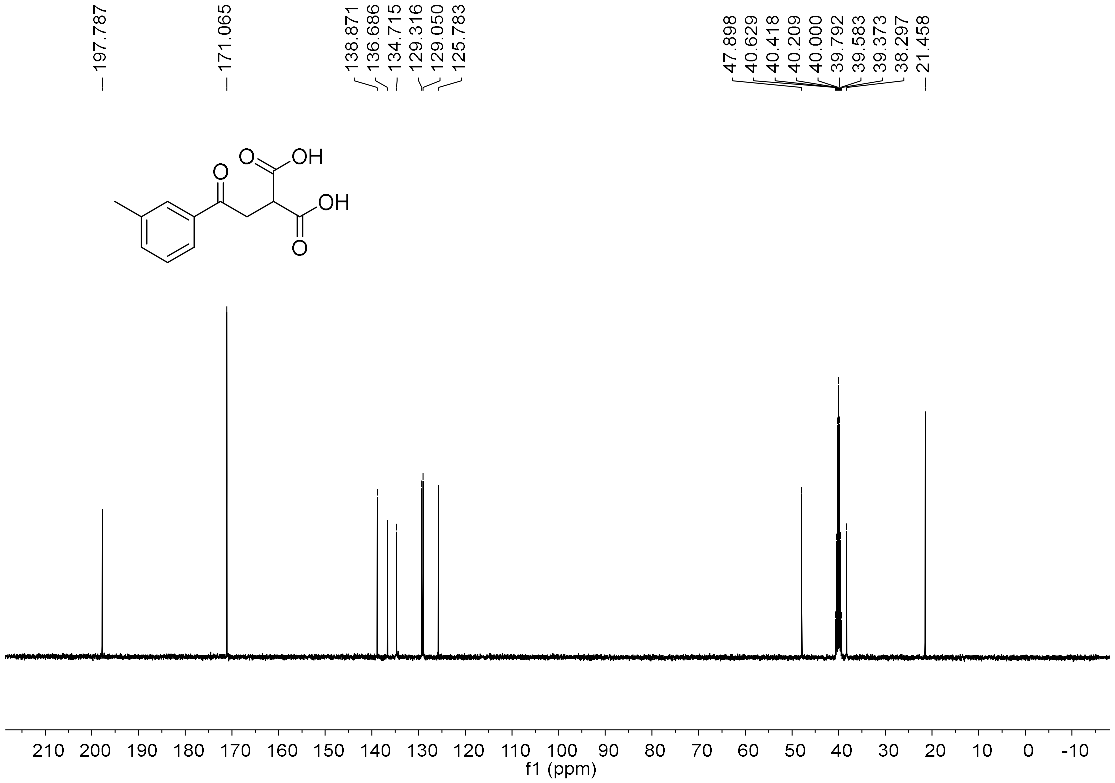

400 MHz ^{1}H NMR Spectrum of 12 in DMSO-d_6

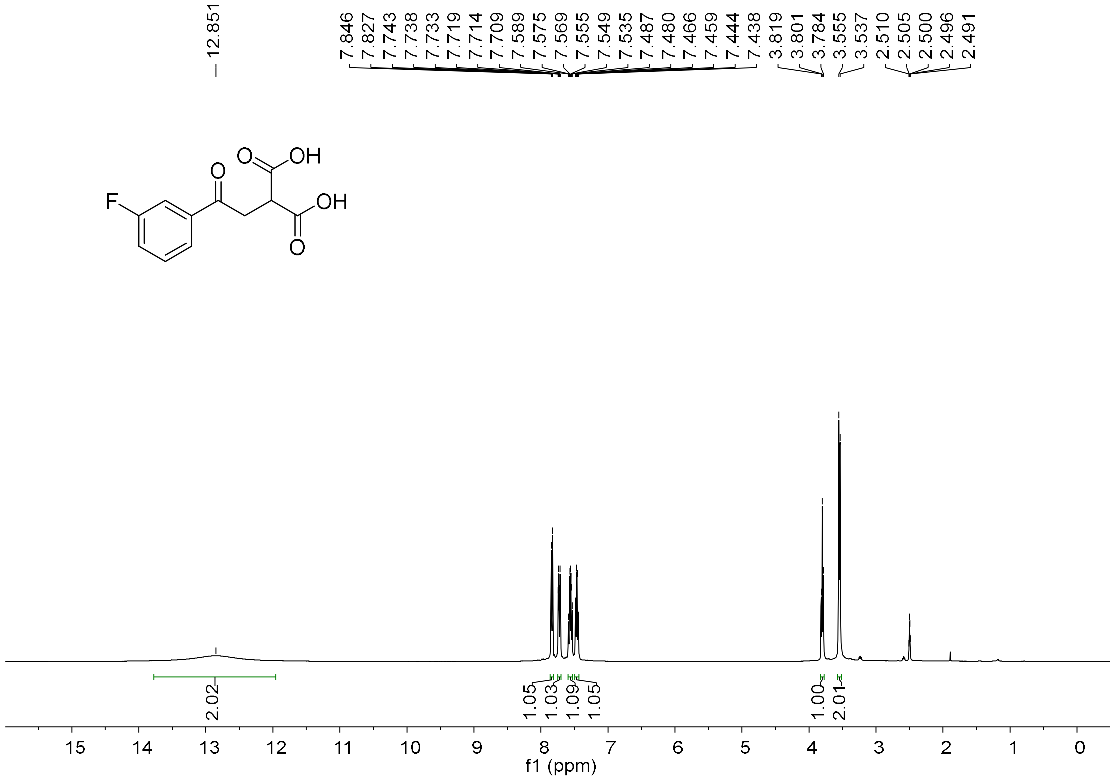

100 MHz ^{13}C{1H} NMR Spectrum of 12 in DMSO-d_6

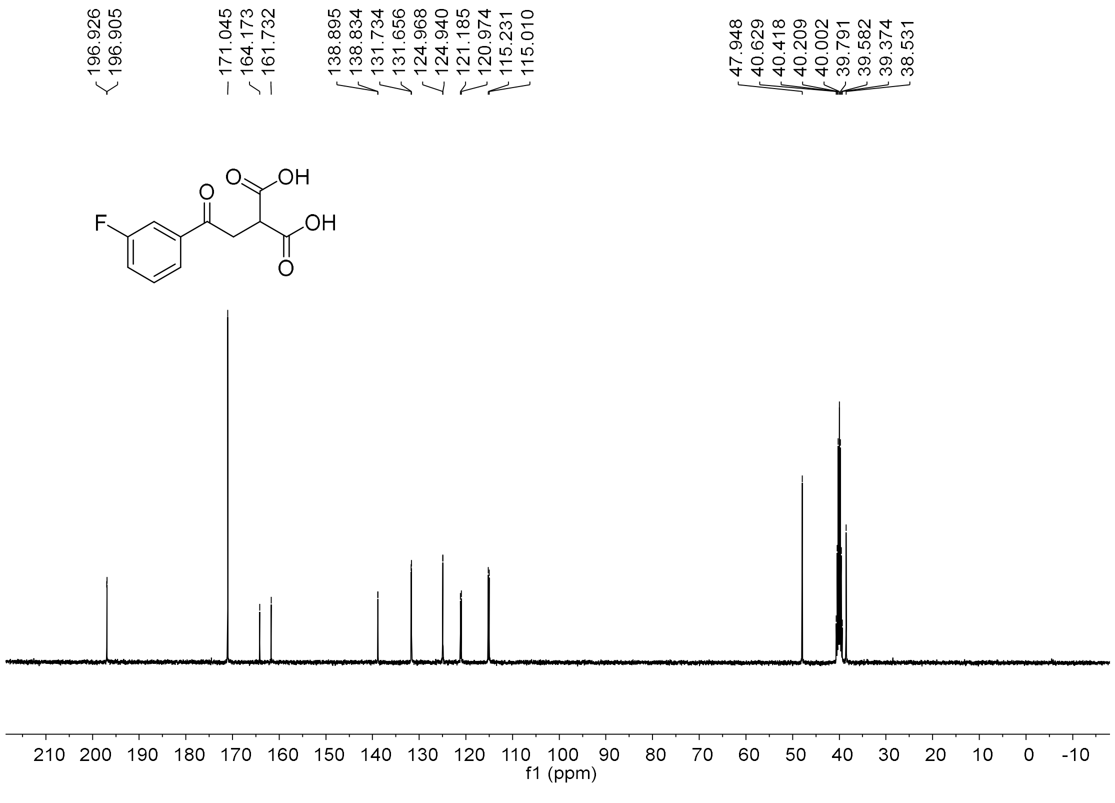

400 MHz ^{1}H NMR Spectrum of 13 in DMSO-d_6

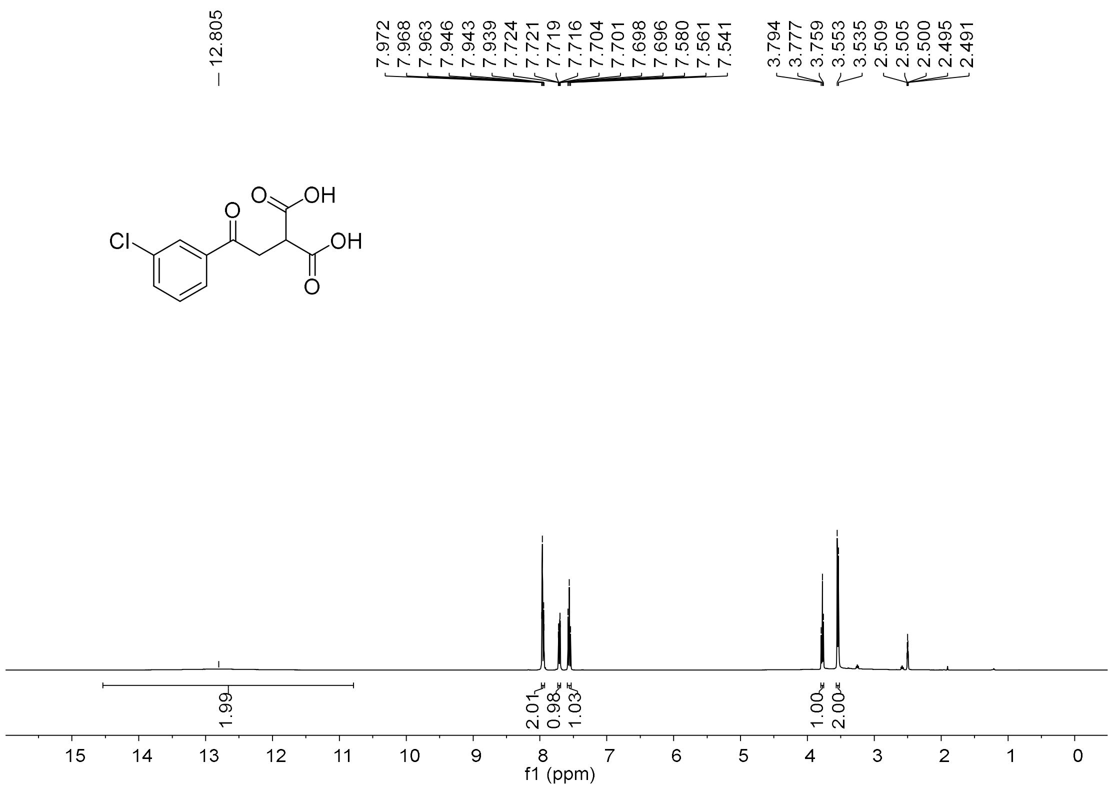

100 MHz ^{13}C{1H} NMR Spectrum of 13 in DMSO-d_6

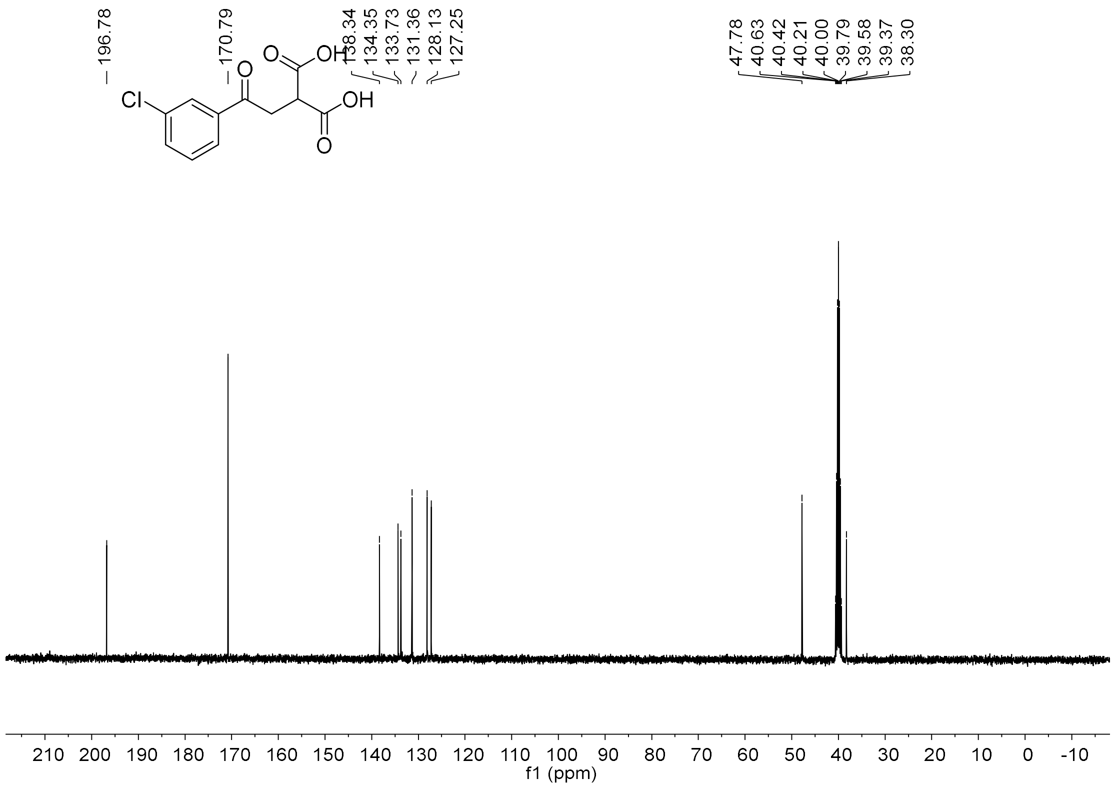

400 MHz ^{1}H NMR Spectrum of 14 in DMSO-d_6

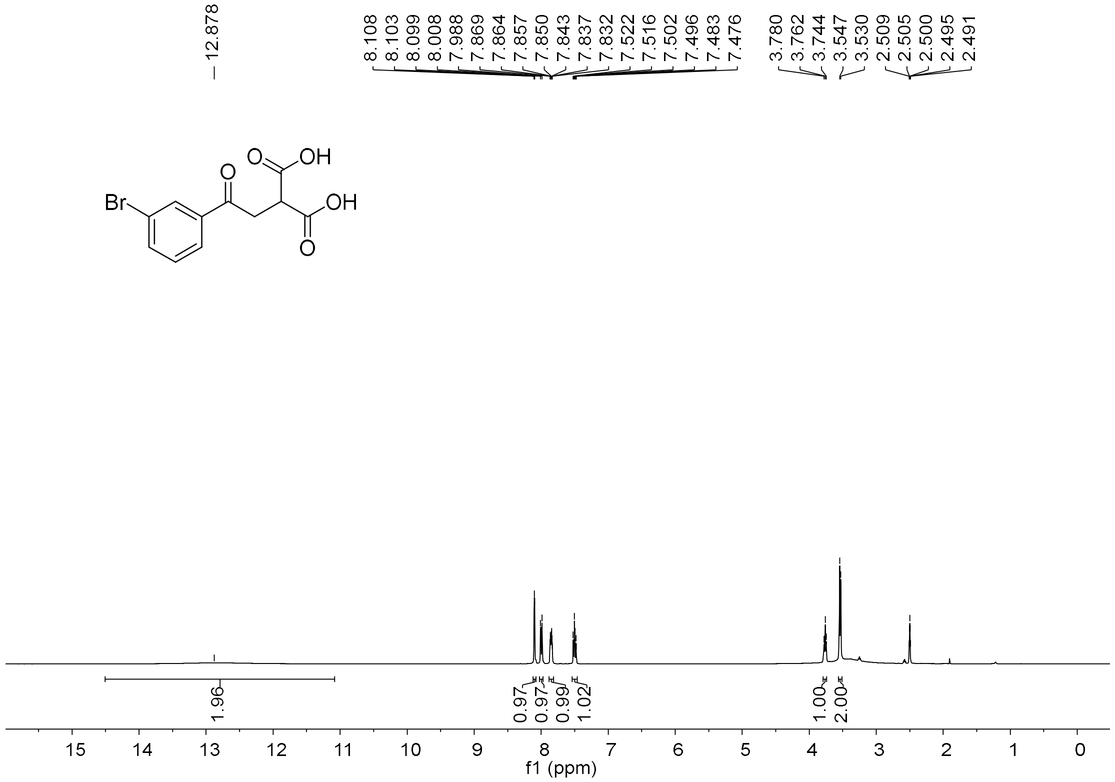

100 MHz ^{13}C{1H} NMR Spectrum of 14 in DMSO-d_6

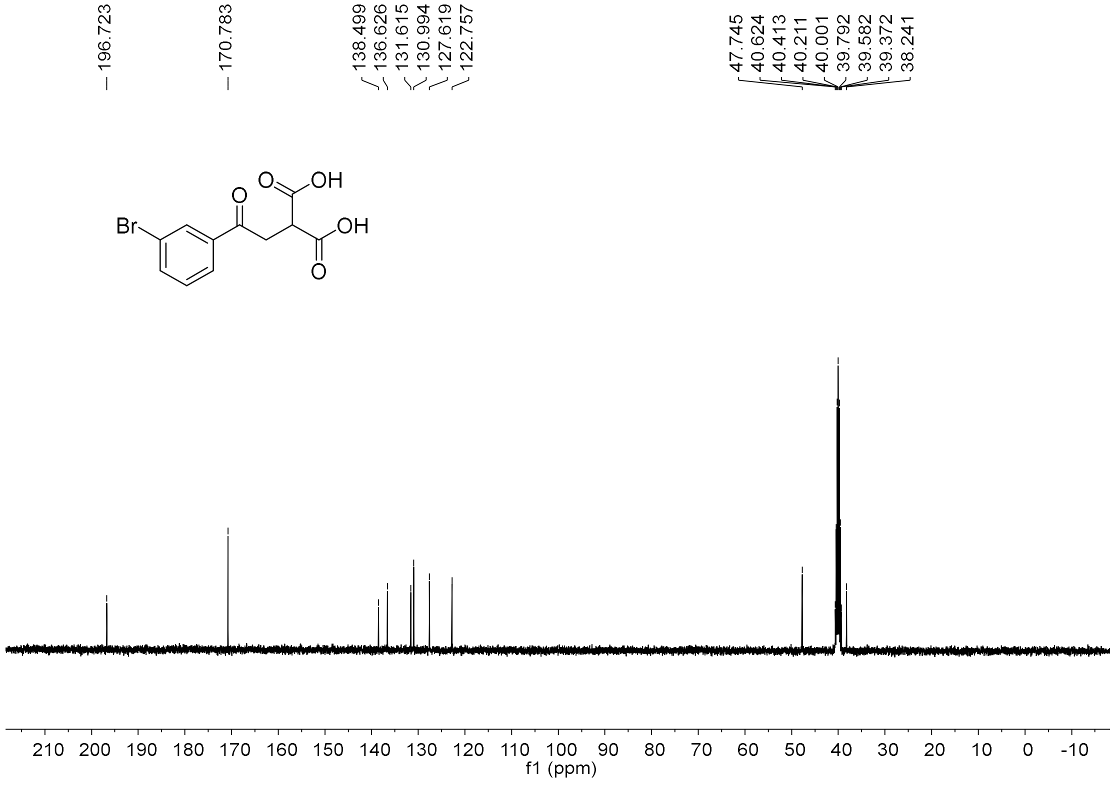

400 MHz ^{1}H NMR Spectrum of 15 in DMSO-d_6

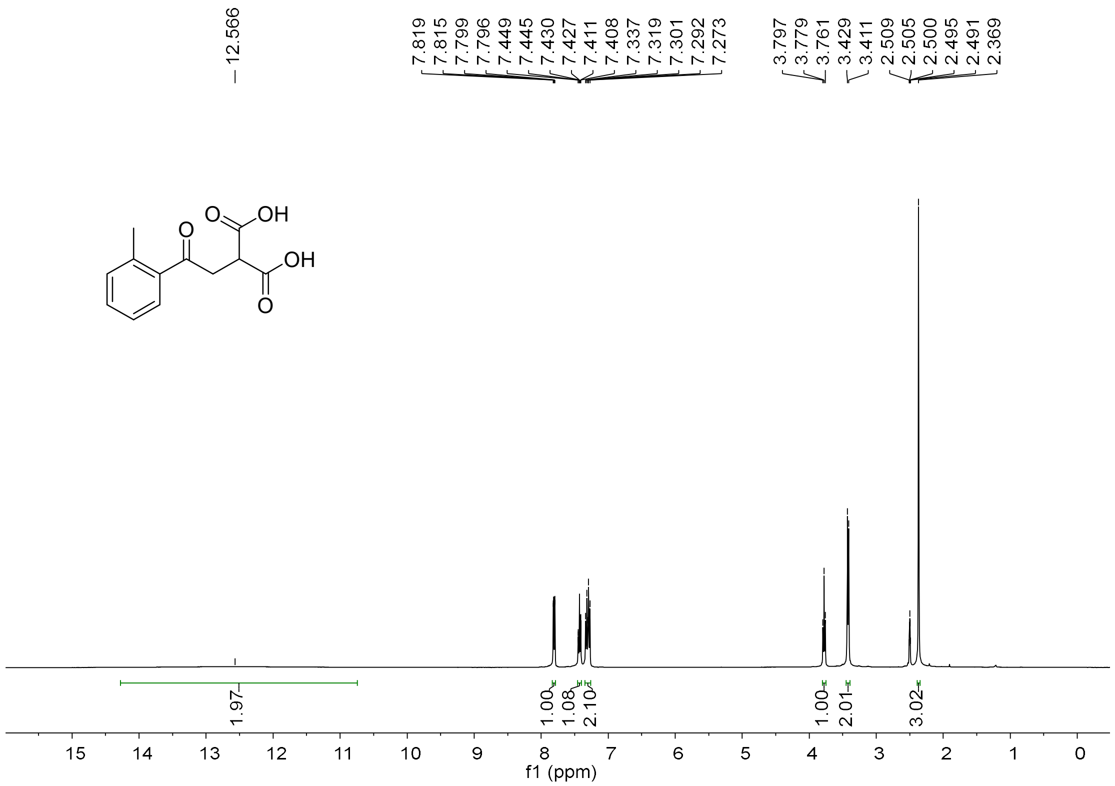

100 MHz ^{13}C{1H} NMR Spectrum of 15 in DMSO(d_6)

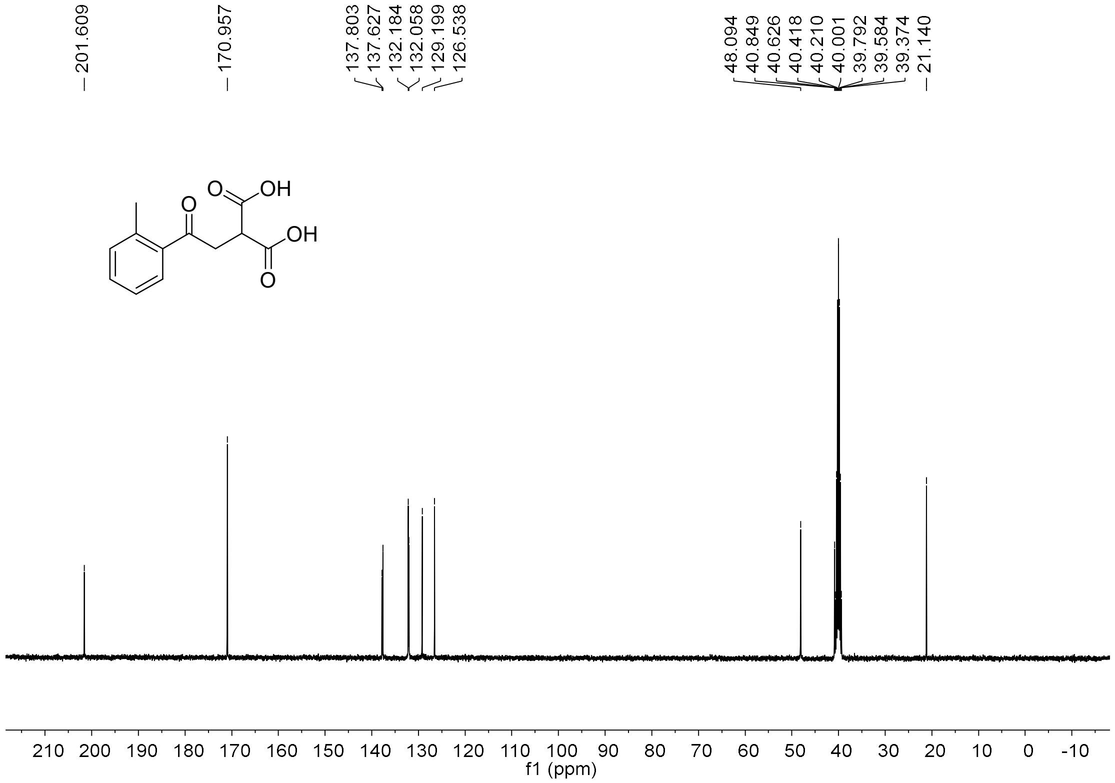

400 MHz ^{1}H NMR Spectrum of 16 in DMSO-d_6

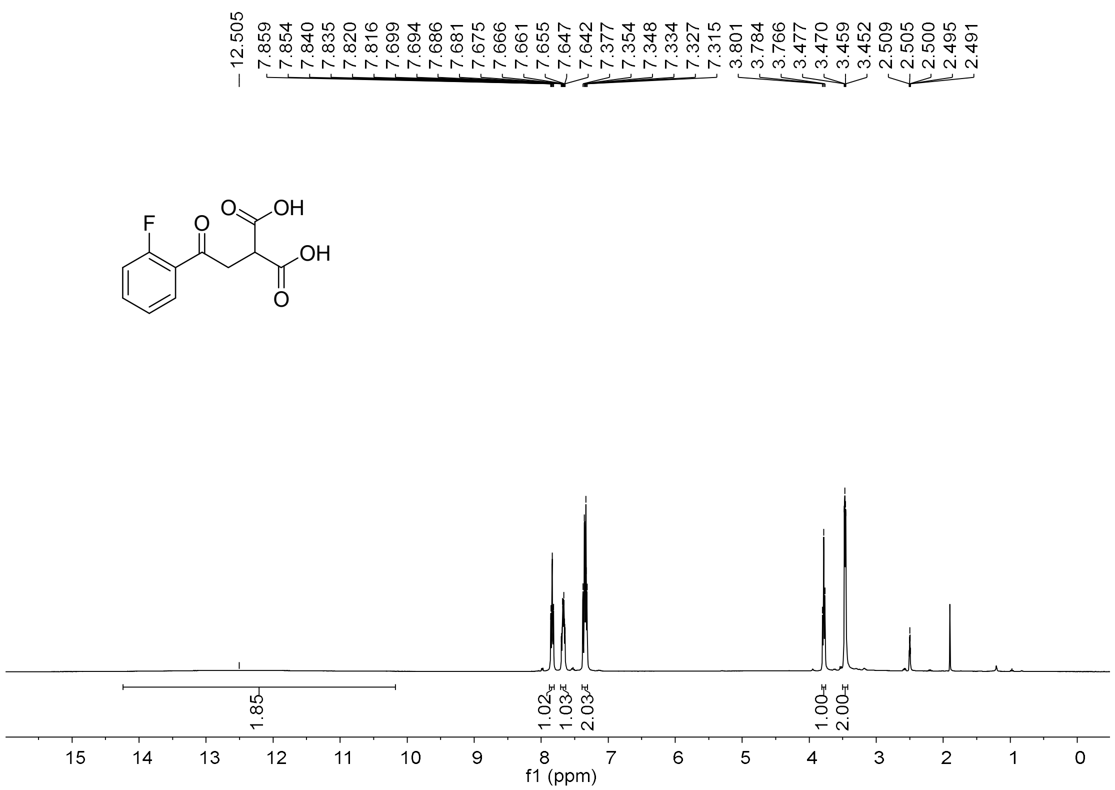

100 MHz ^{13}C{1H} NMR Spectrum of 16 in DMSO-d_6

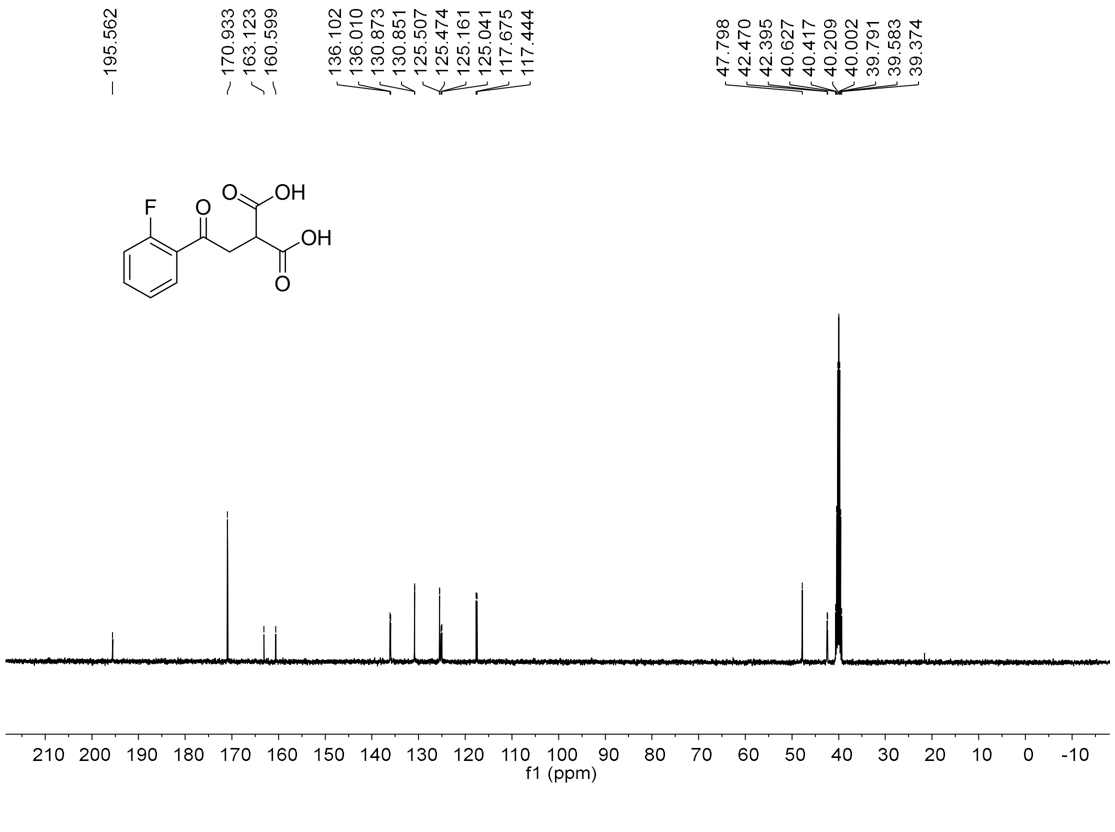

400 MHz ^{1}H NMR Spectrum of 17 in DMSO-d_6

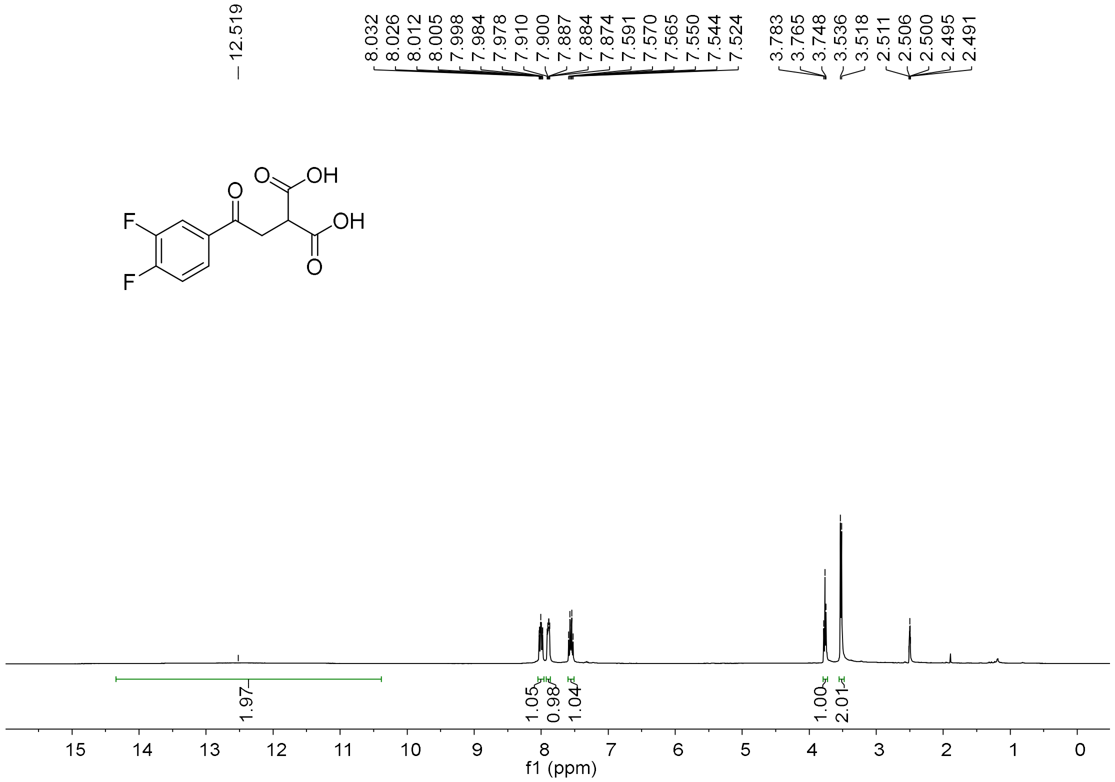

100 MHz ^{13}C{1H} NMR Spectrum of 17 in DMSO-d_6

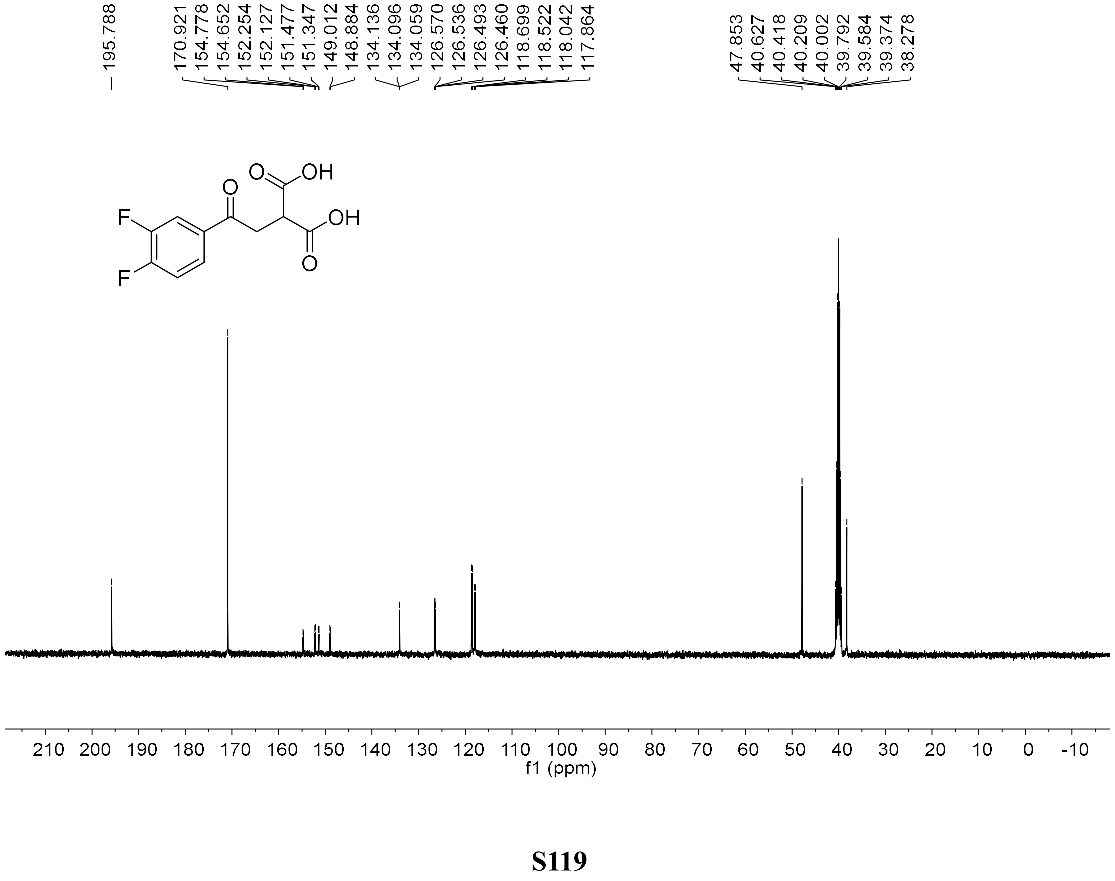

400 MHz ^{1}H NMR Spectrum of 18 in DMSO-d_6

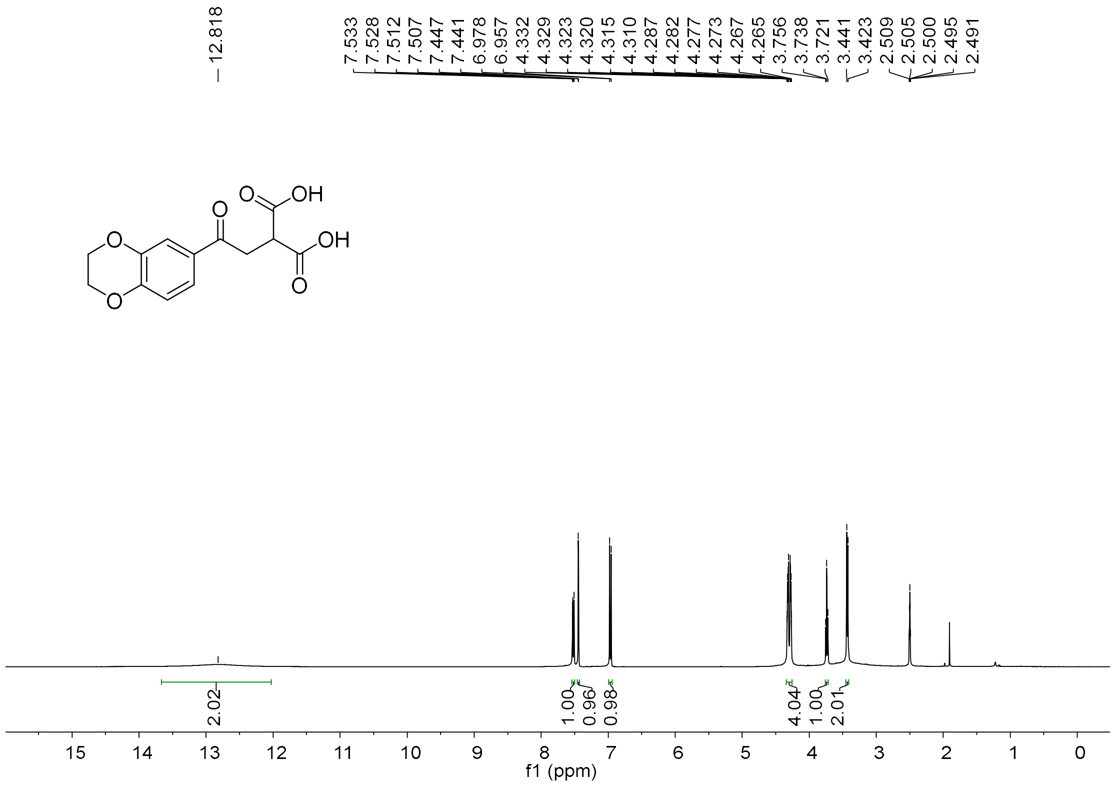

100 MHz ^{13}C{1H} NMR Spectrum of 18 in DMSO-d_6

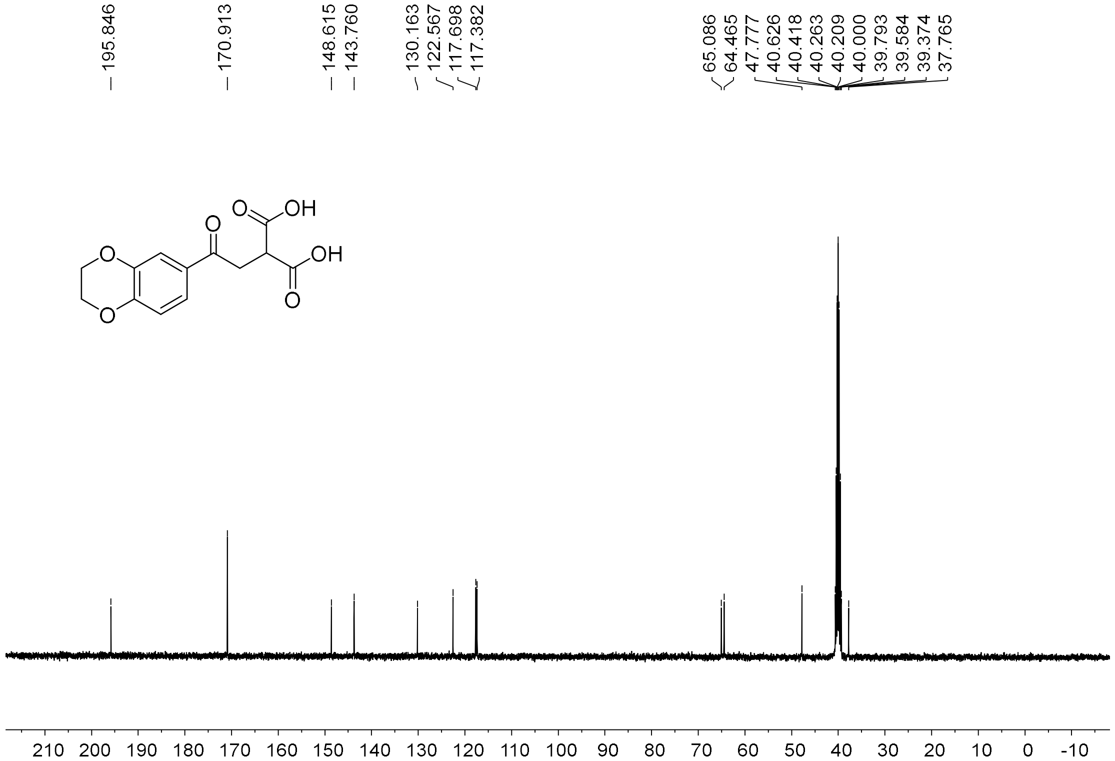

400 MHz ^{1}H NMR Spectrum of 19 in DMSO-d_6

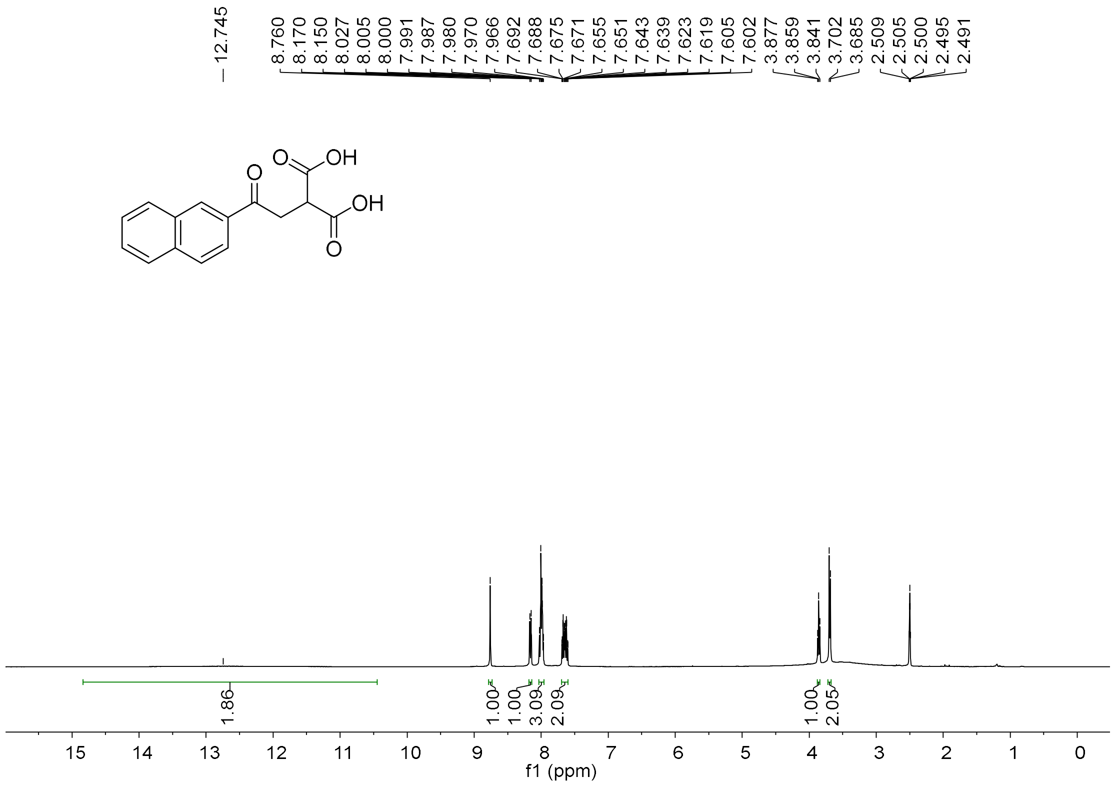

100 MHz ^{13}C{1H} NMR Spectrum of 19 in DMSO-d_6

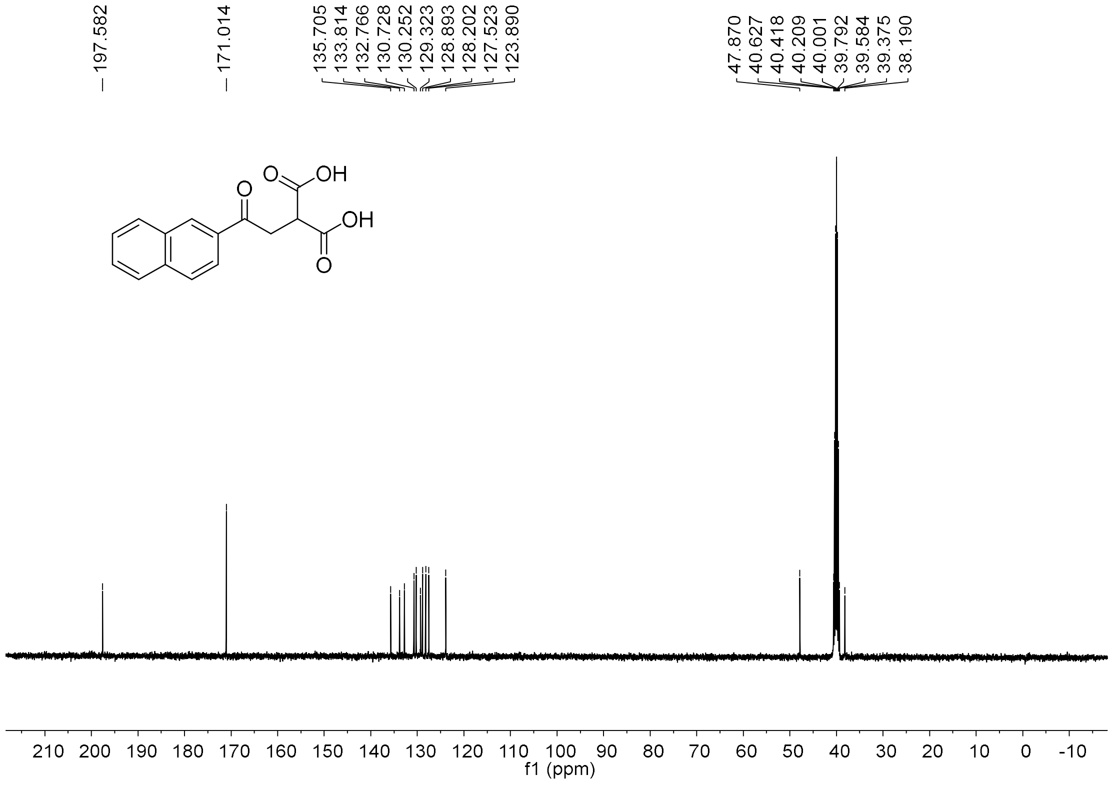

400 MHz ^{1}H NMR Spectrum of 20 in DMSO-d_6

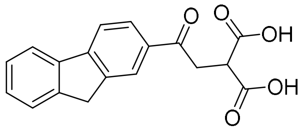

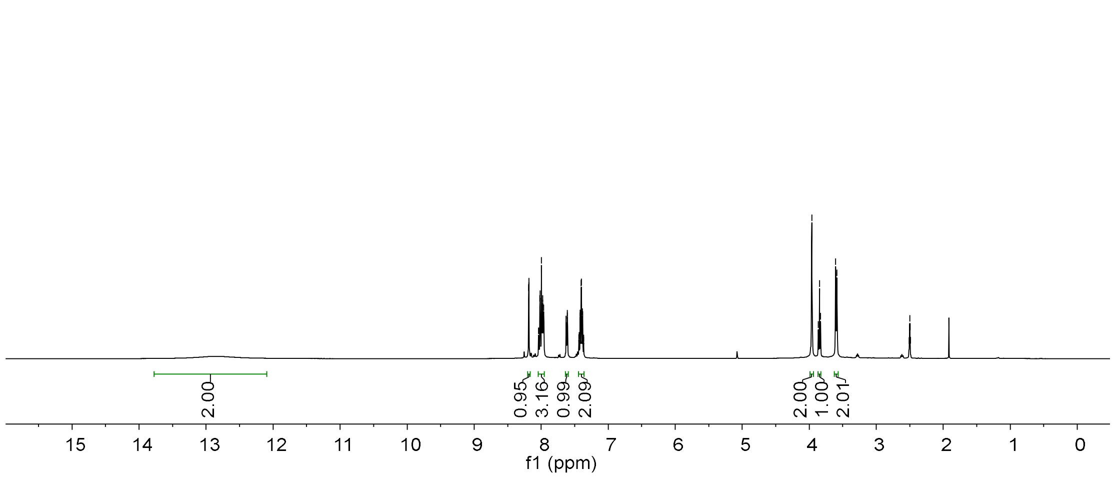

100 MHz ^{13}C{1H} NMR Spectrum of 20 in DMSO-d_6

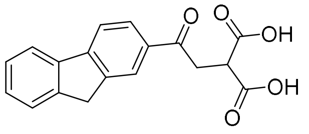

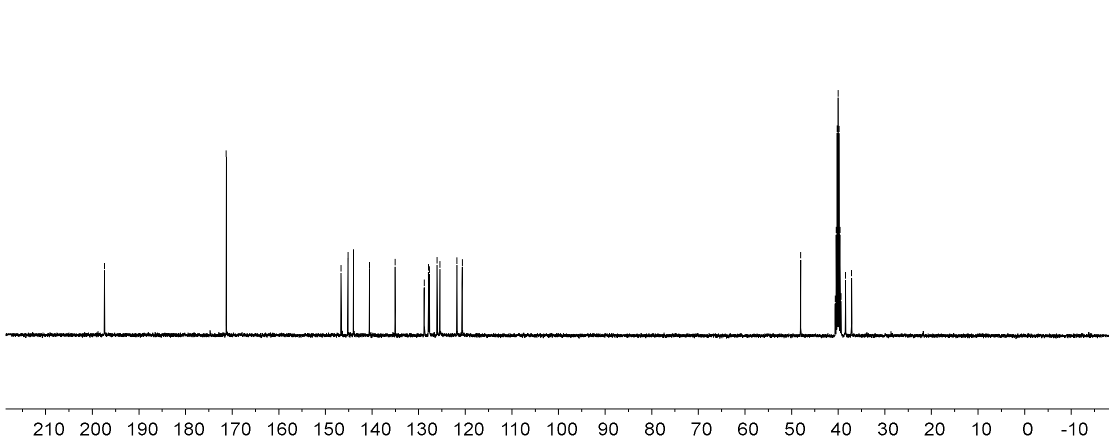

400 MHz ^{1}H NMR Spectrum of 21 in DMSO-d_6

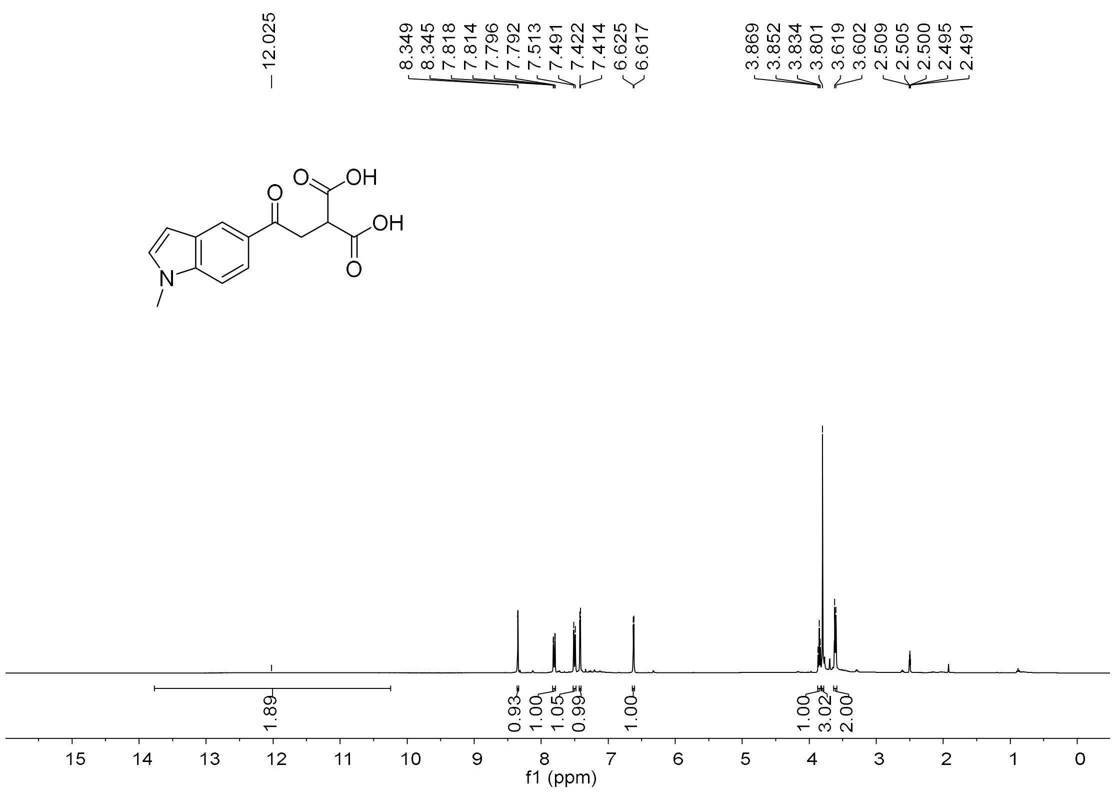

100 MHz ^{13}C{1H} NMR Spectrum of 21 in DMSO-d_6

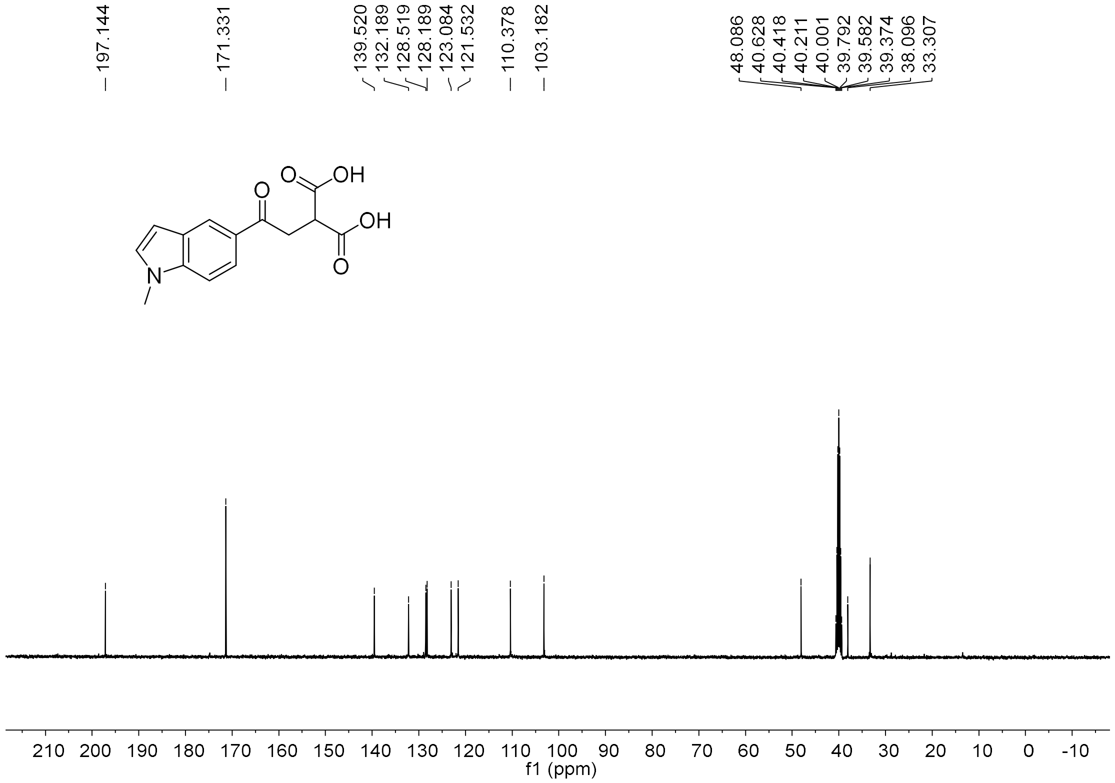

400 MHz ^{1}H NMR Spectrum of 22 in DMSO-d_6

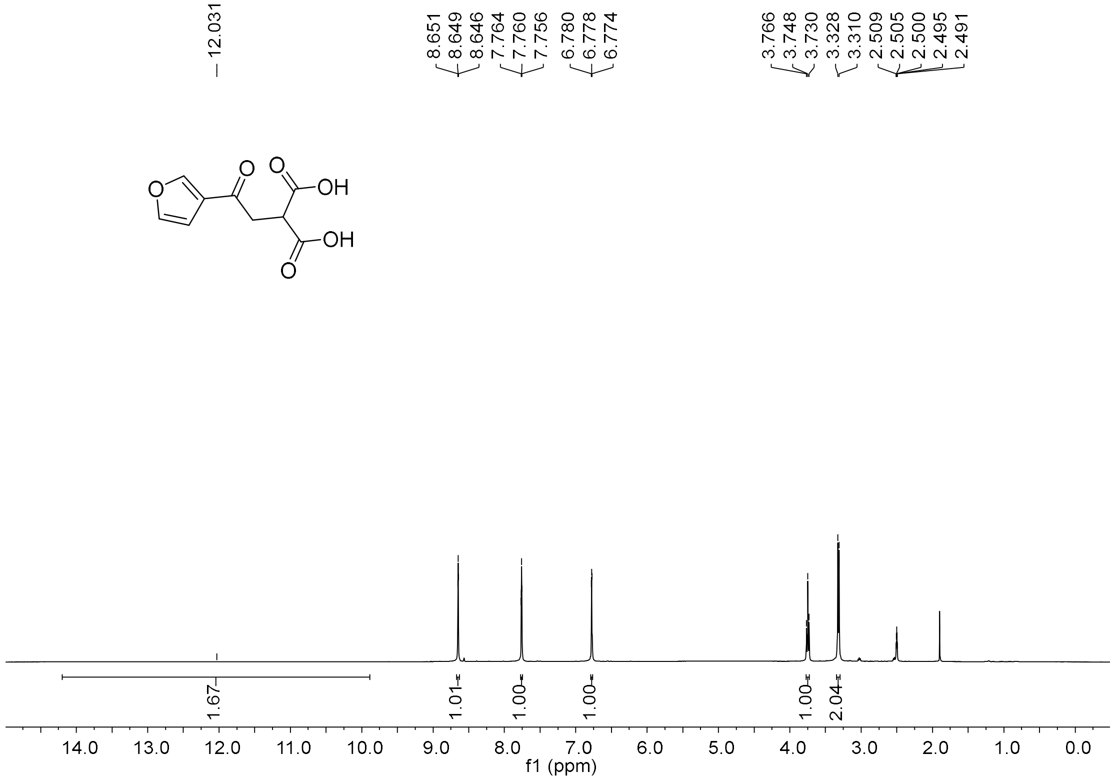

100 MHz ^{13}C{1H} NMR Spectrum of 22 in DMSO-d_6

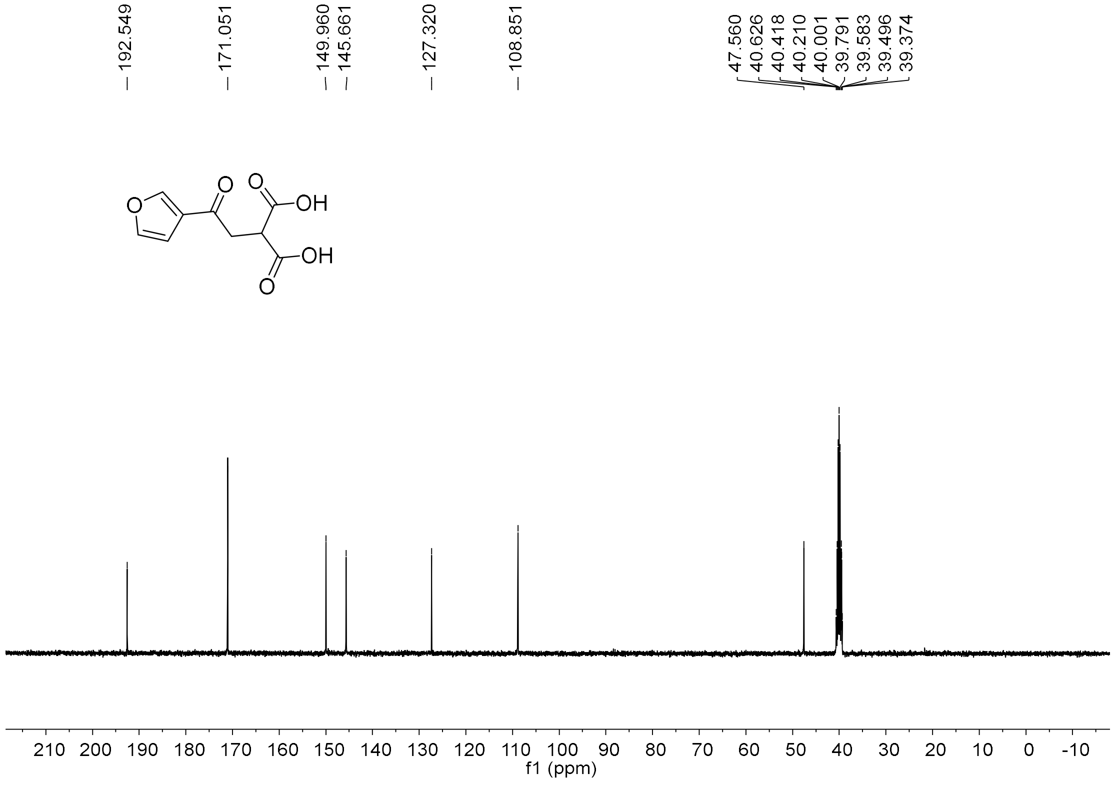

400 MHz ^{1}H NMR Spectrum of 23 in DMSO-d_6

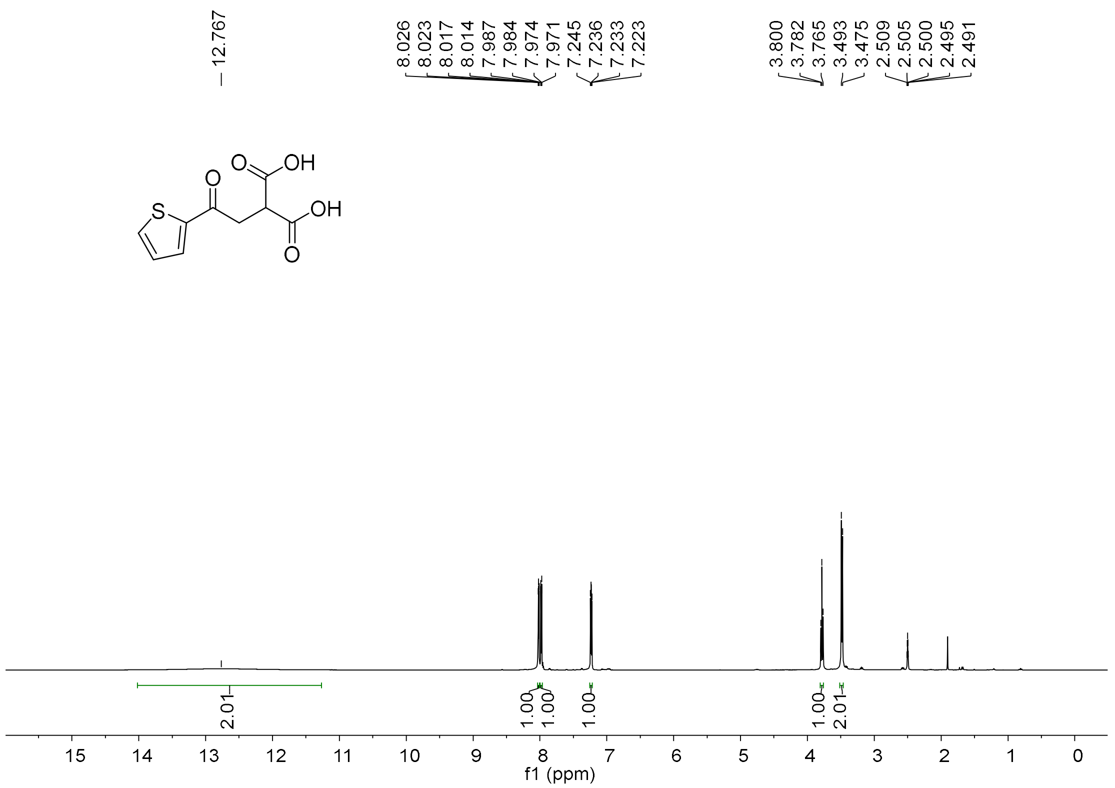

100 MHz ^{13}C{1H} NMR Spectrum of 23 in DMSO-d_6

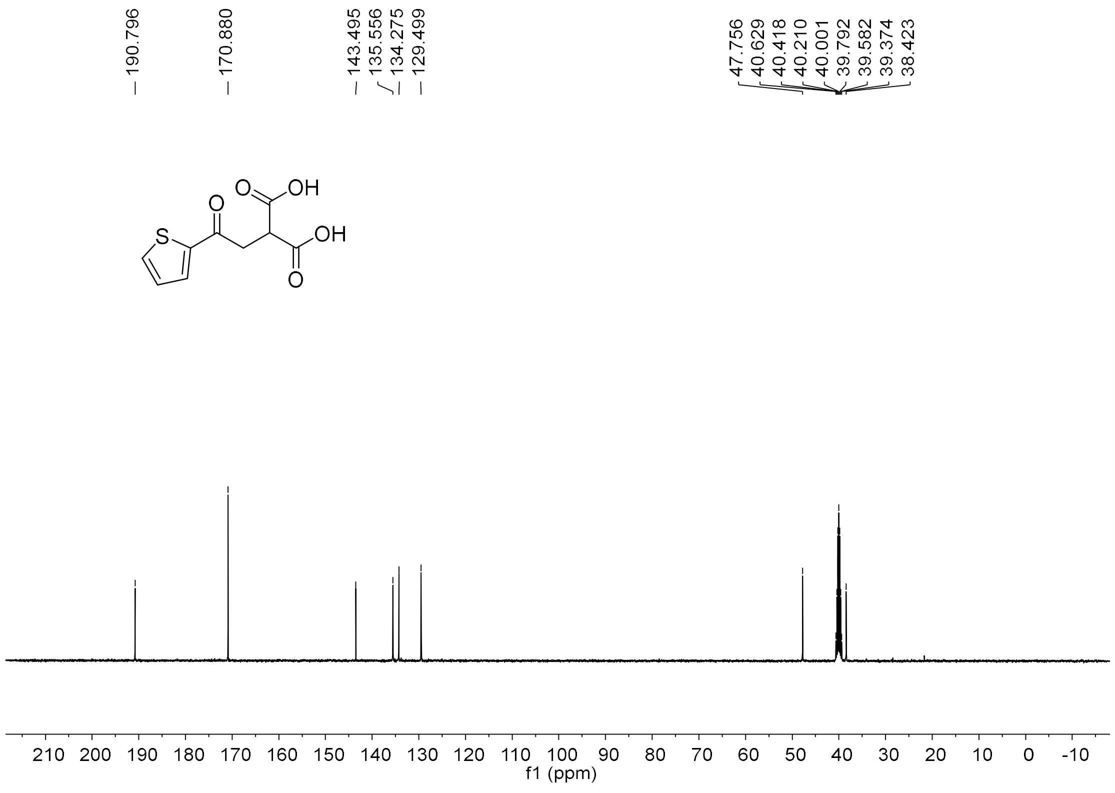

400 MHz ^{1}H NMR Spectrum of 24 in DMSO-d_6

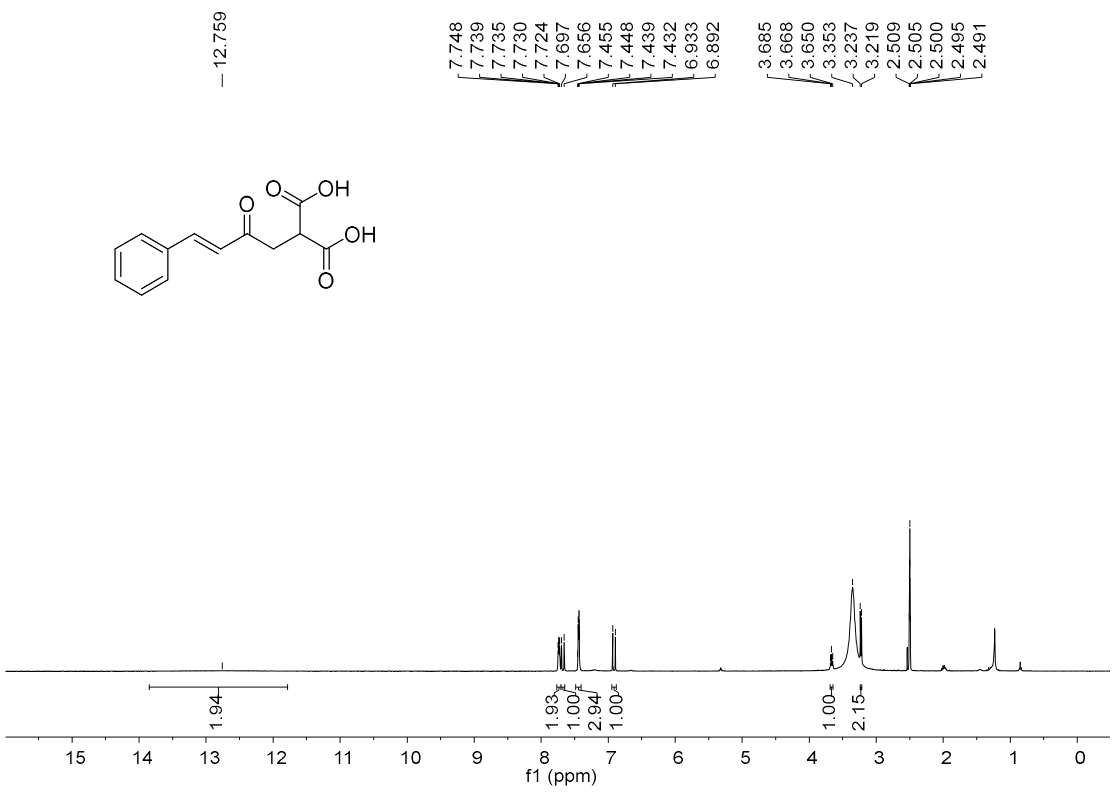

100 MHz ^{13}C{1H} NMR Spectrum of 24 in DMSO-d_6

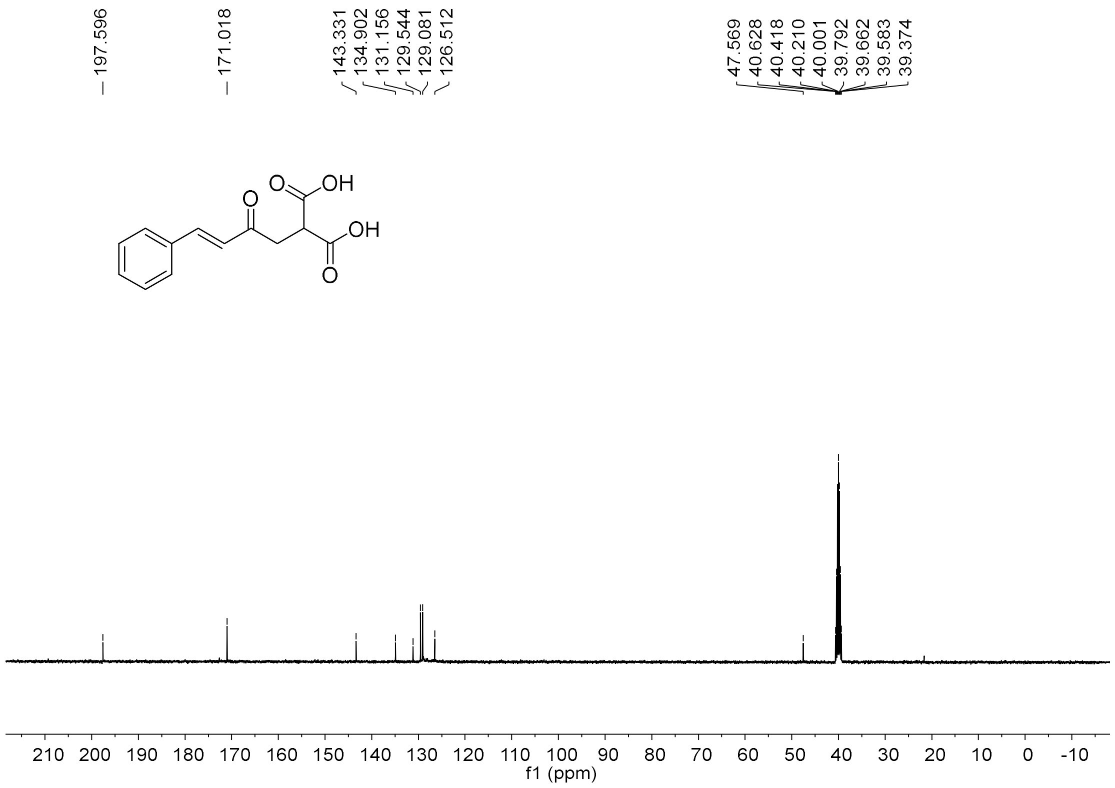

400 MHz ^{1}H NMR Spectrum of 25 in DMSO-d_6

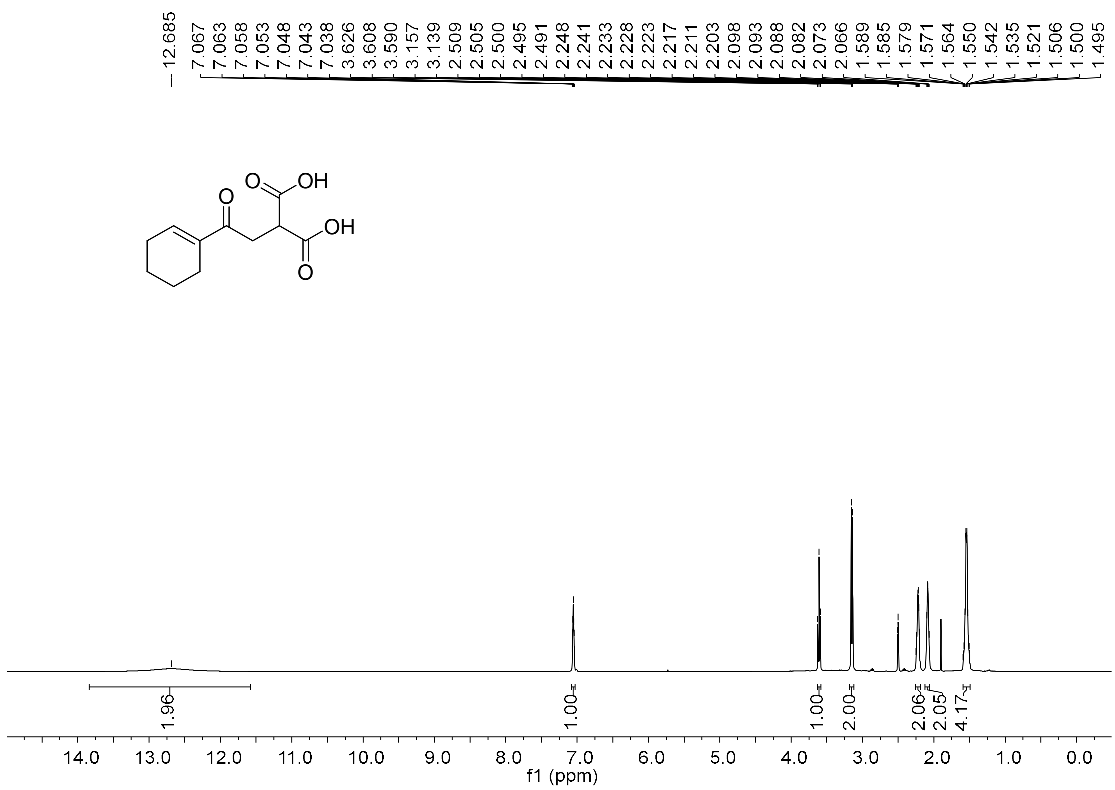

100 MHz ^{13}C{1H} NMR Spectrum of 25 in DMSO-d_6

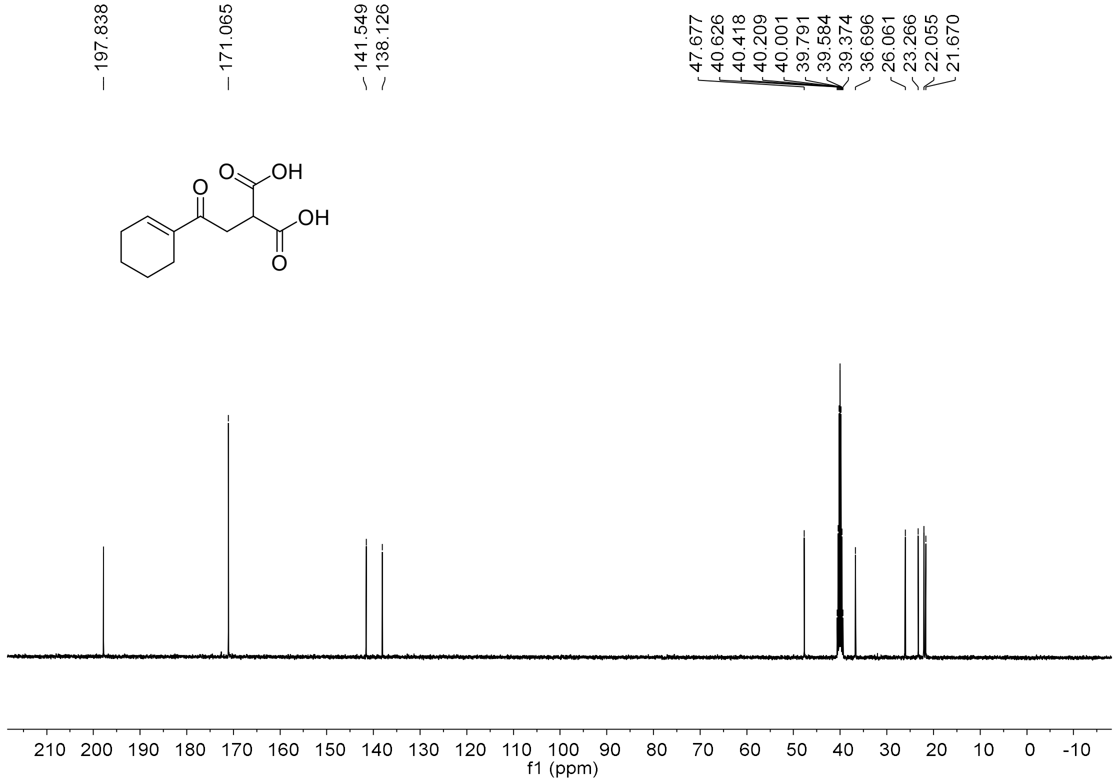

400 MHz ^{1}H NMR Spectrum of 27 in DMSO-d_6

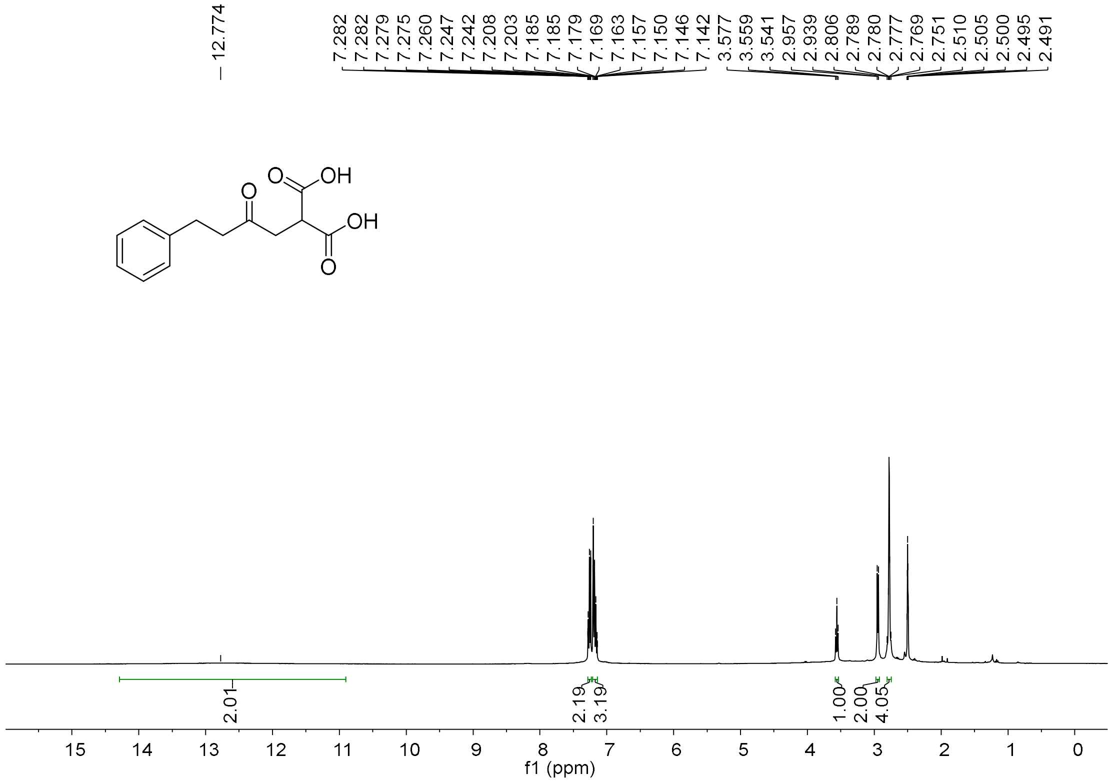

100 MHz ^{13}C{1H} NMR Spectrum of 27 in DMSO-d_6

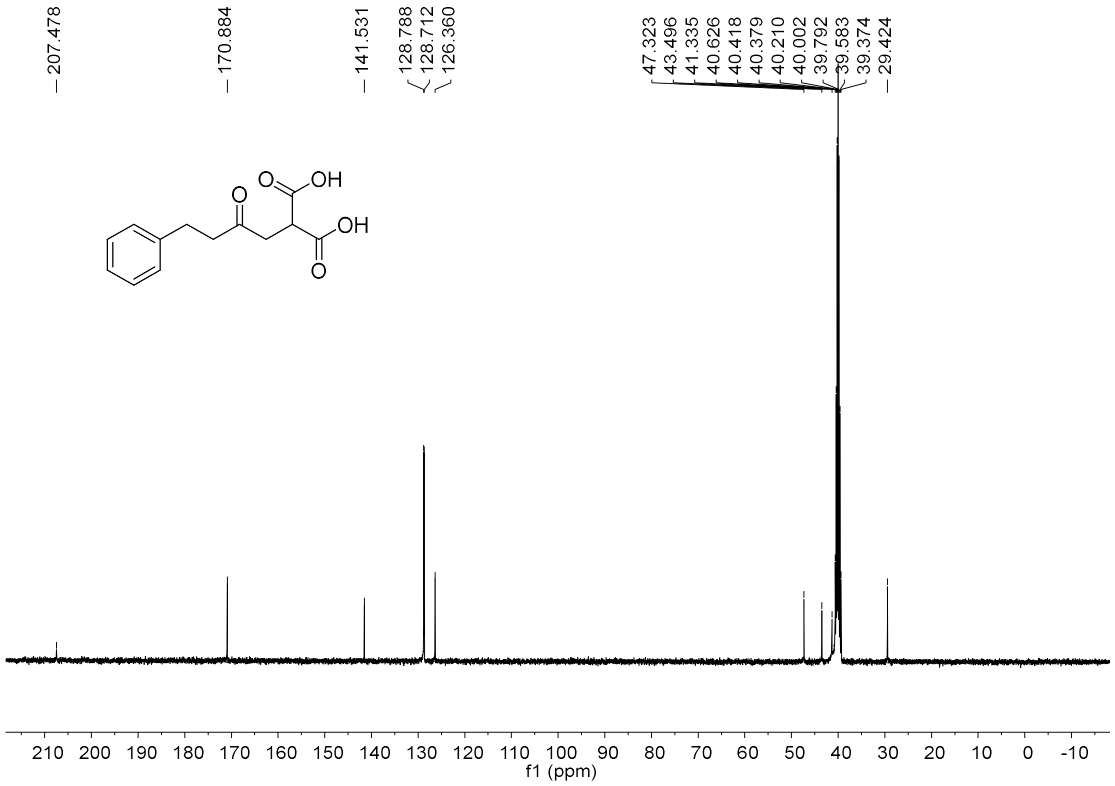

400 MHz ^{1}H NMR Spectrum of 28 in DMSO-d_6

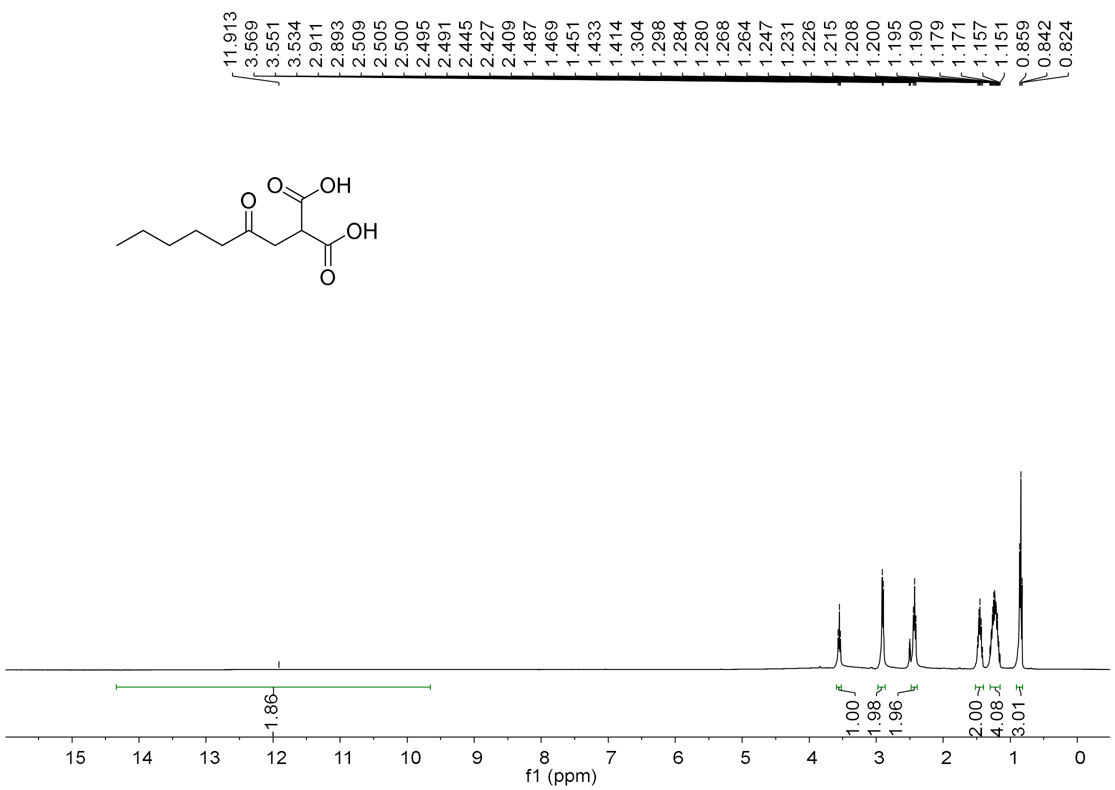

100 MHz ^{13}C{1H} NMR Spectrum of 28 in DMSO-d_6

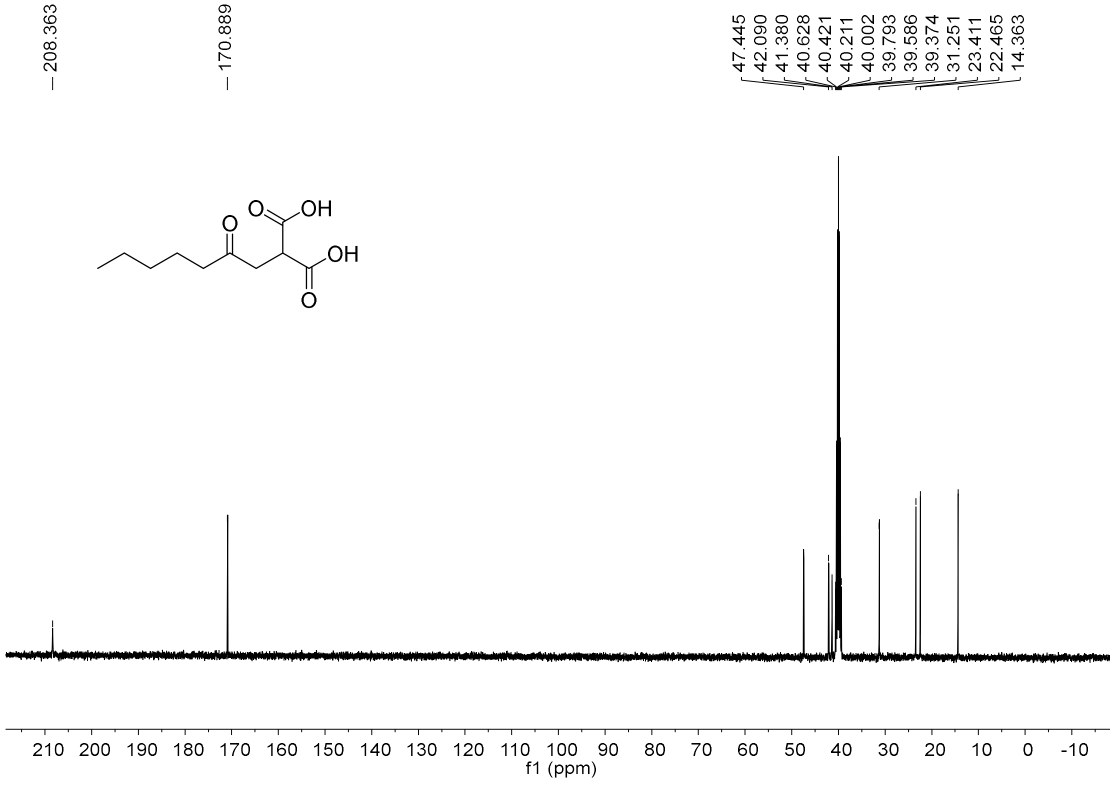

400 MHz ^{1}H NMR Spectrum of 29 in DMSO-d_6

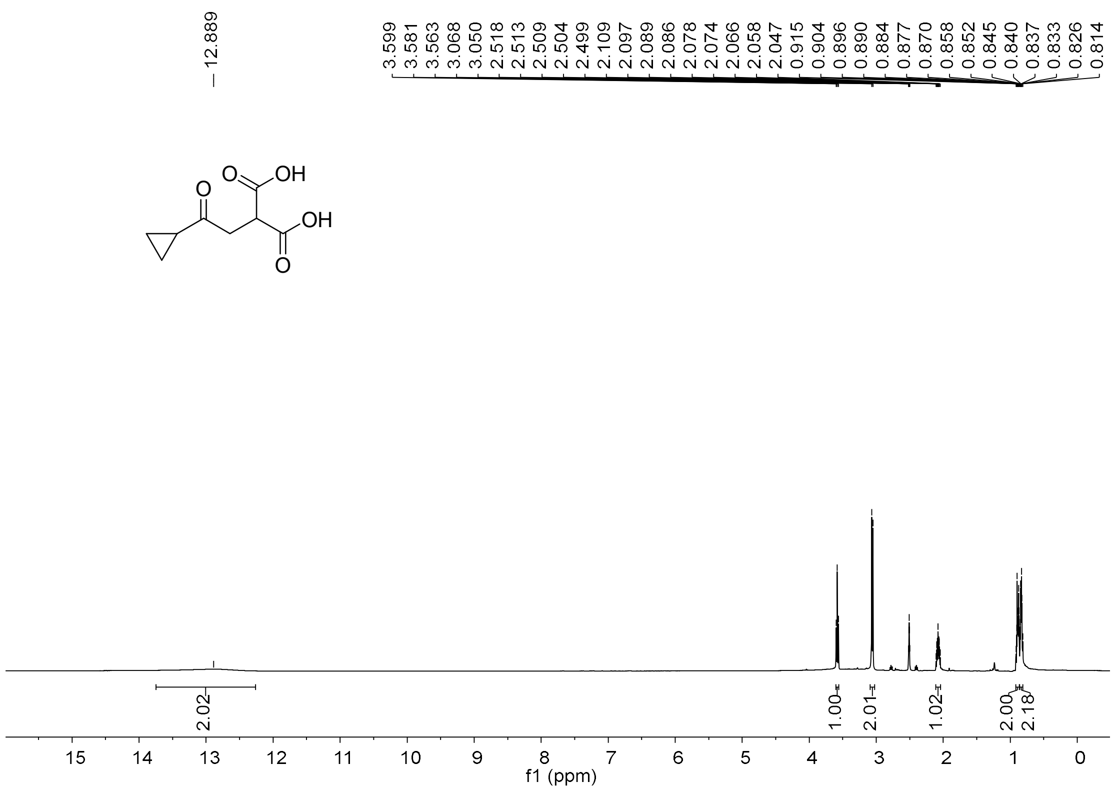

100 MHz ^{13}C{1H} NMR Spectrum of 29 in DMSO-d_6

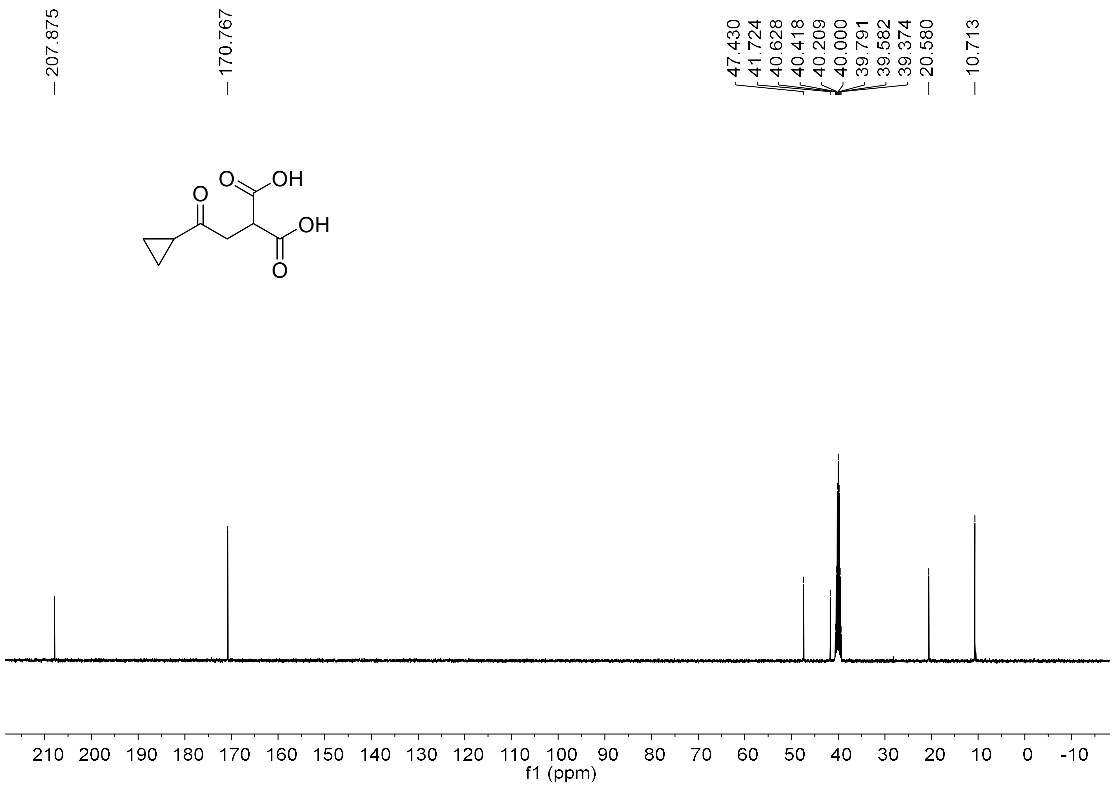

400 MHz ¹H NMR Spectrum of 30 in DMSO-d_6

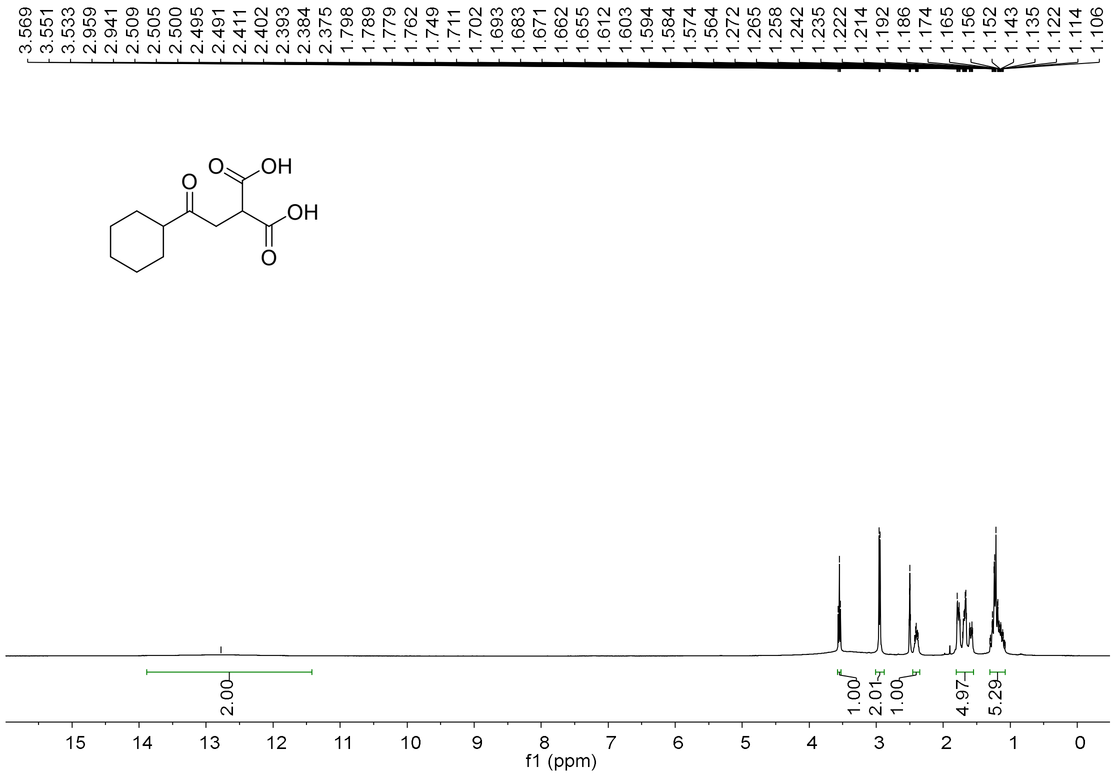

100 MHz ¹³C{1H} NMR Spectrum of 30 in DMSO-d_6

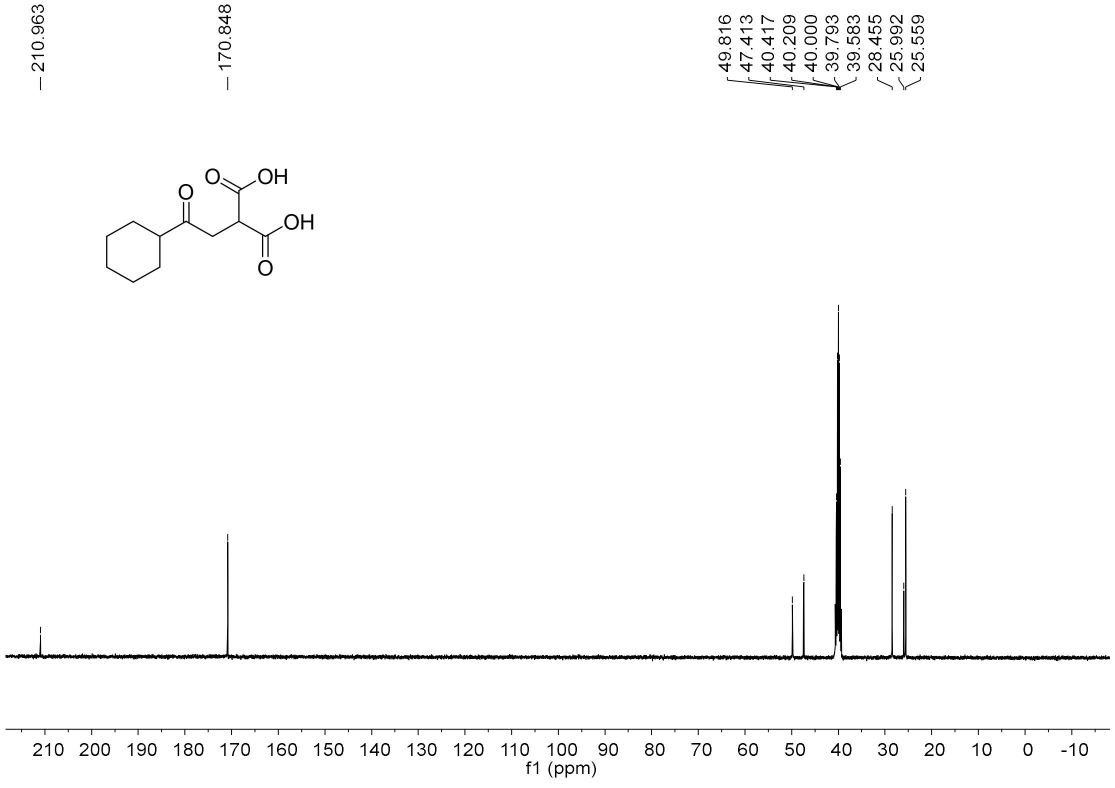

400 MHz ^{1}H NMR Spectrum of 31 in DMSO-d_6

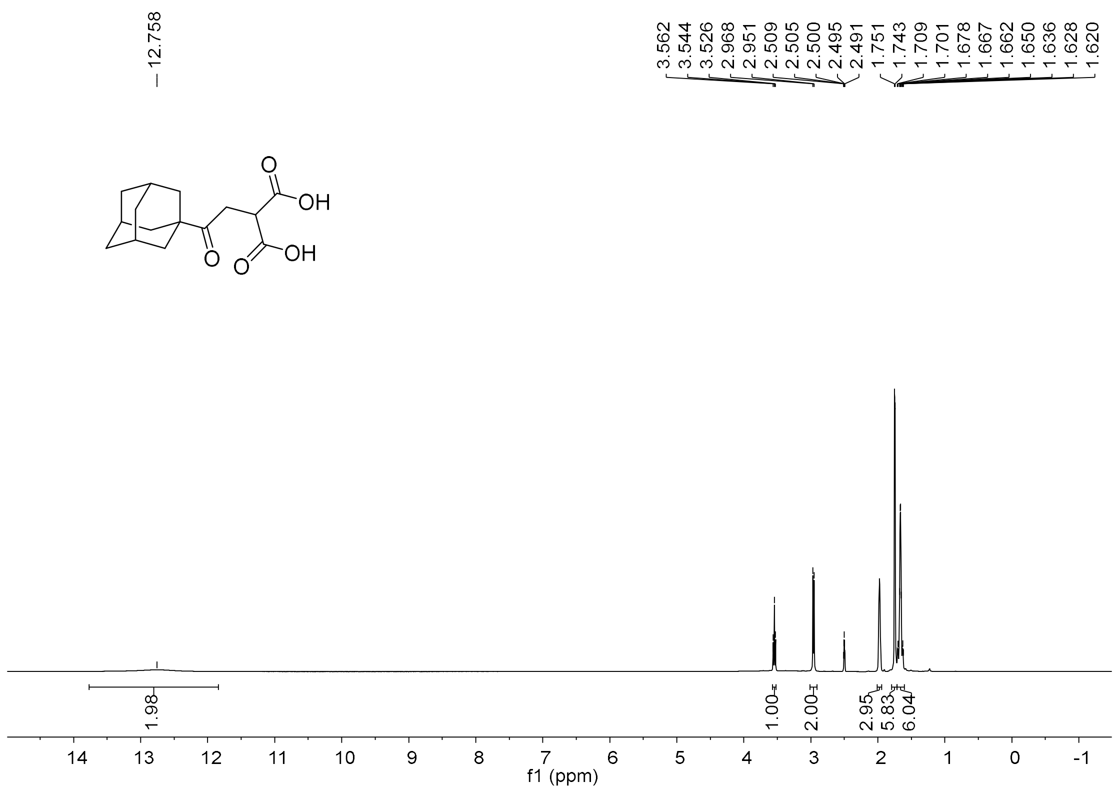

100 MHz $^{13}C\{1H\}$ NMR Spectrum of 31 in DMSO-d_6

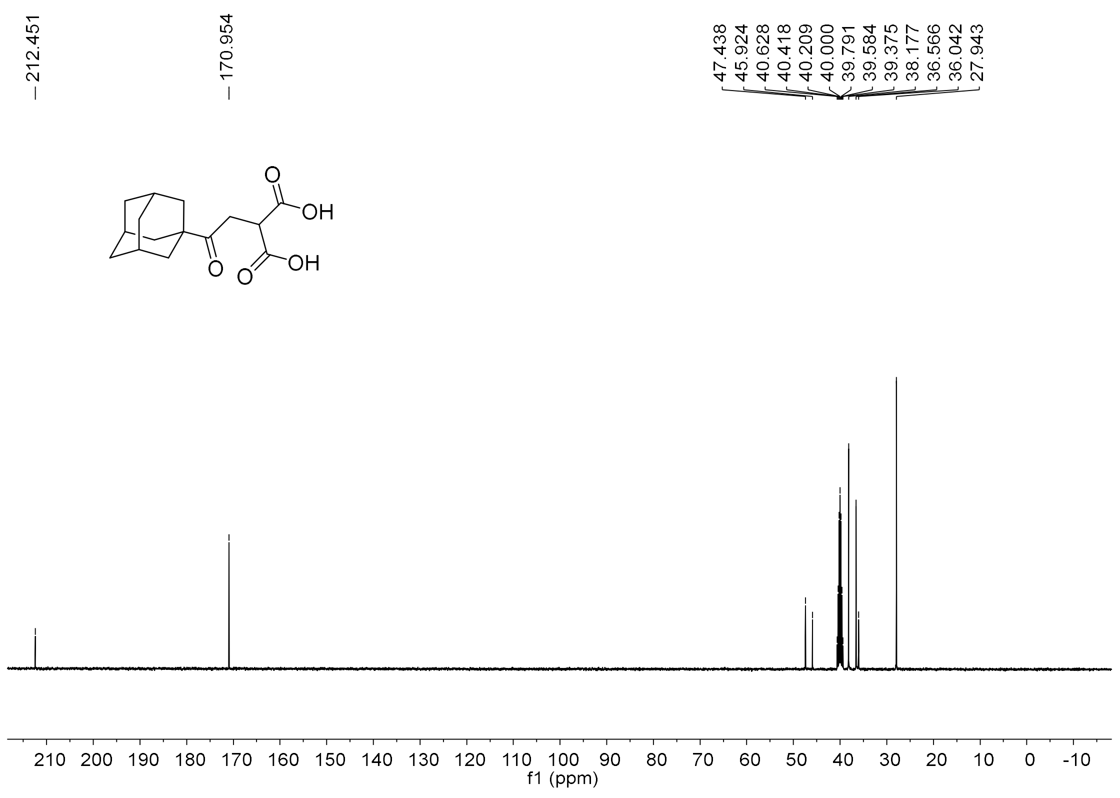

400 MHz ^{1}H NMR Spectrum of 32 in $CDCl_3$

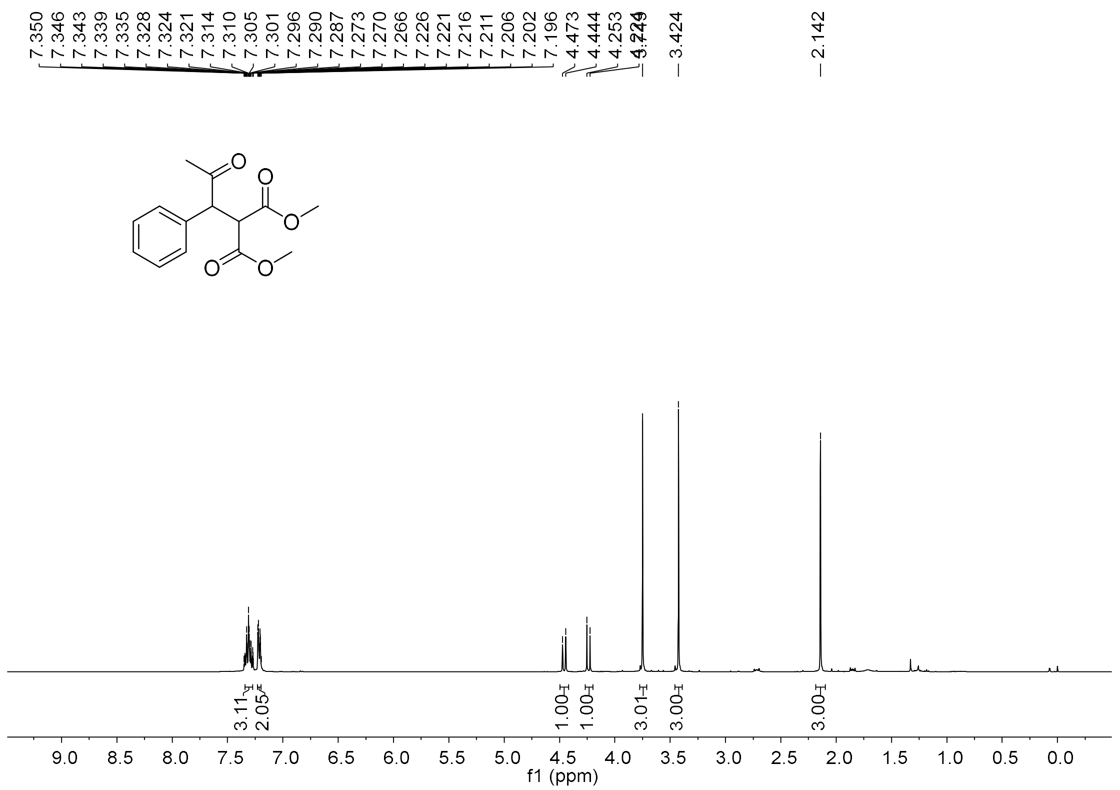

100 MHz ^{13}C{1H} NMR Spectrum of 32 in $CDCl_3$

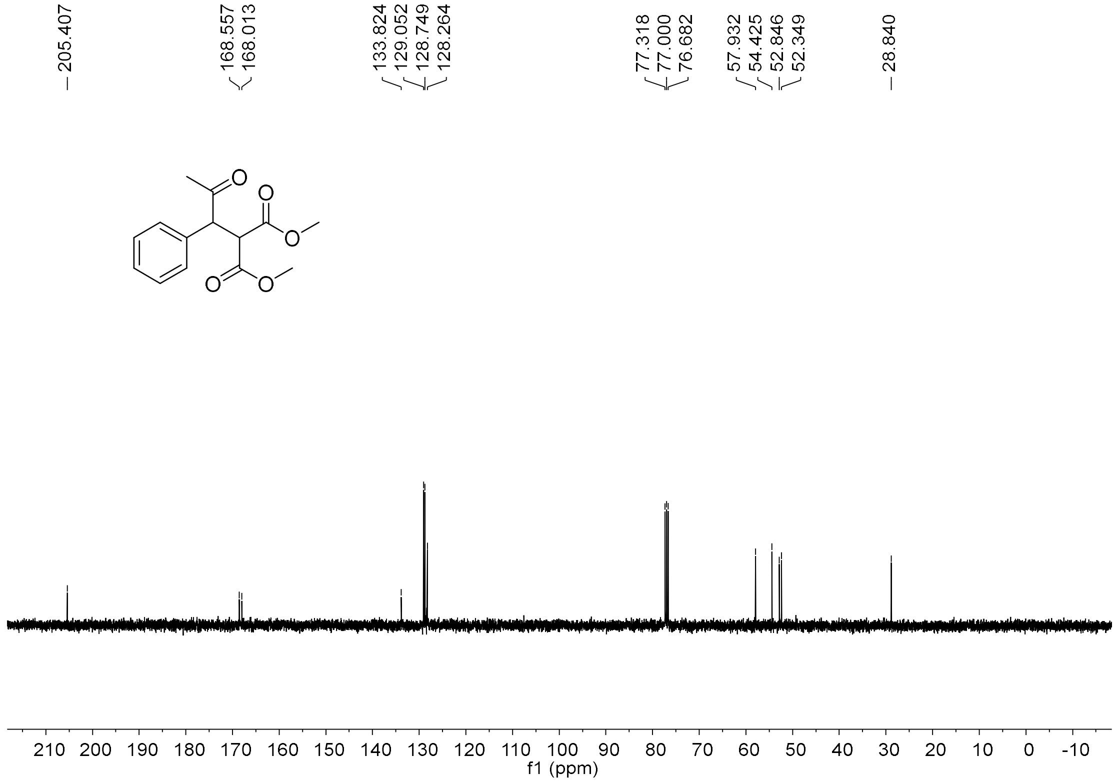

400 MHz ^{1}H NMR Spectrum of 33 in $CDCl_3$

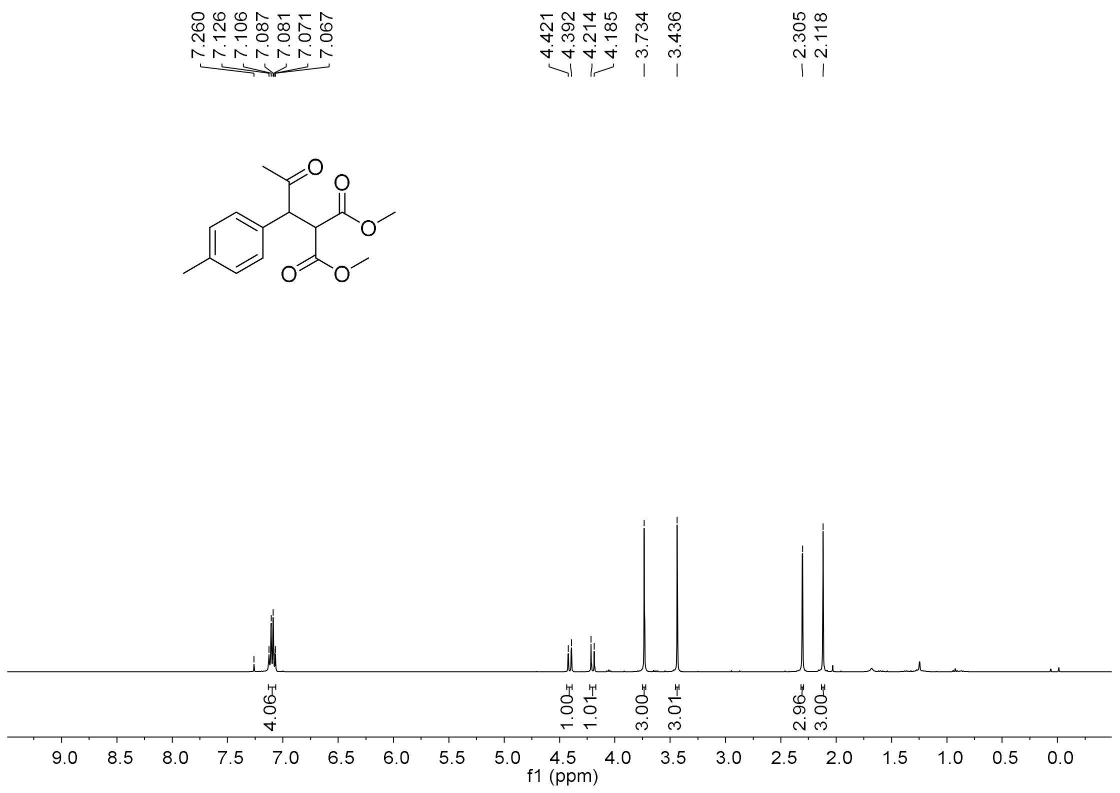

100 MHz ^{13}C{1H} NMR Spectrum of 33 in $CDCl_3$

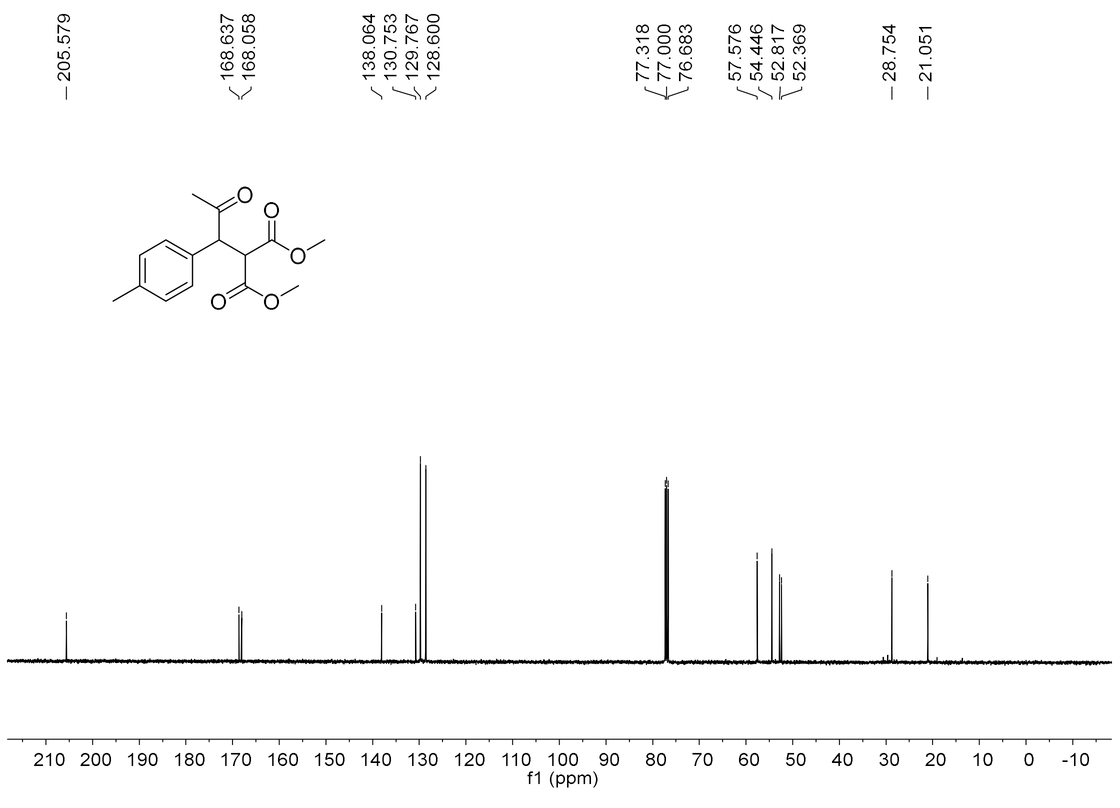

400 MHz ^{1}H NMR Spectrum of 34 in $CDCl_3$

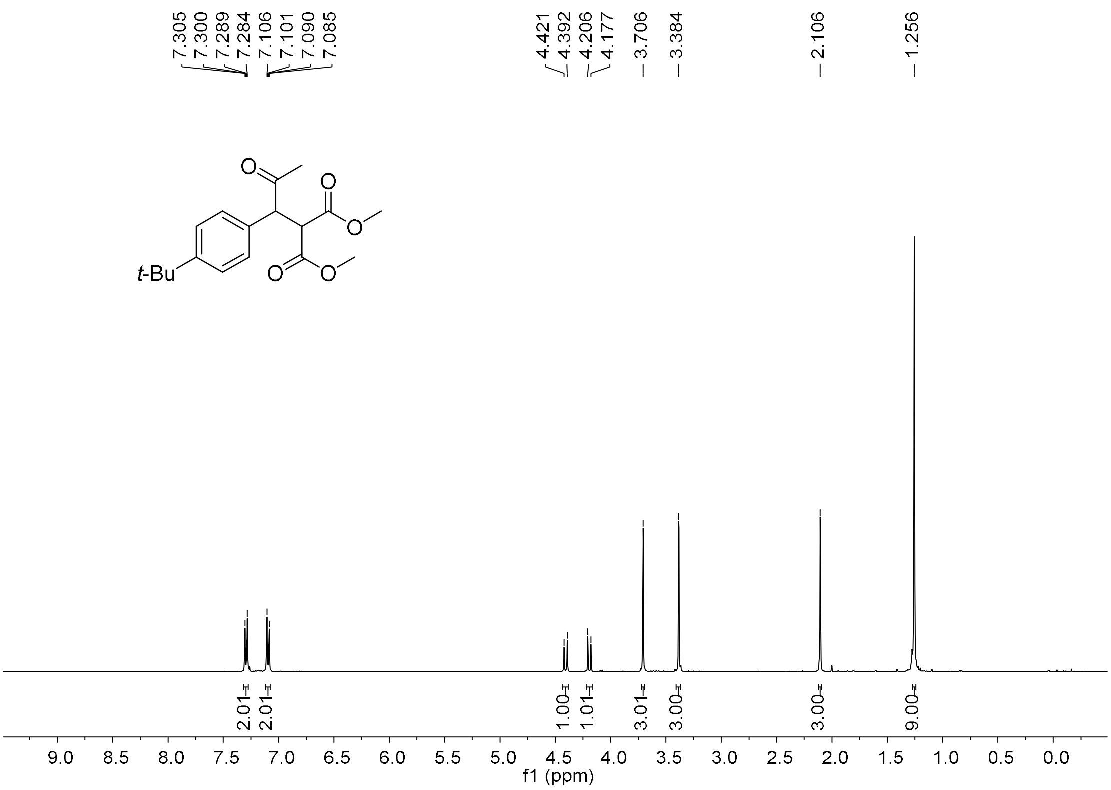

100 MHz ^{13}C{1H} NMR Spectrum of 34 in $CDCl_3$

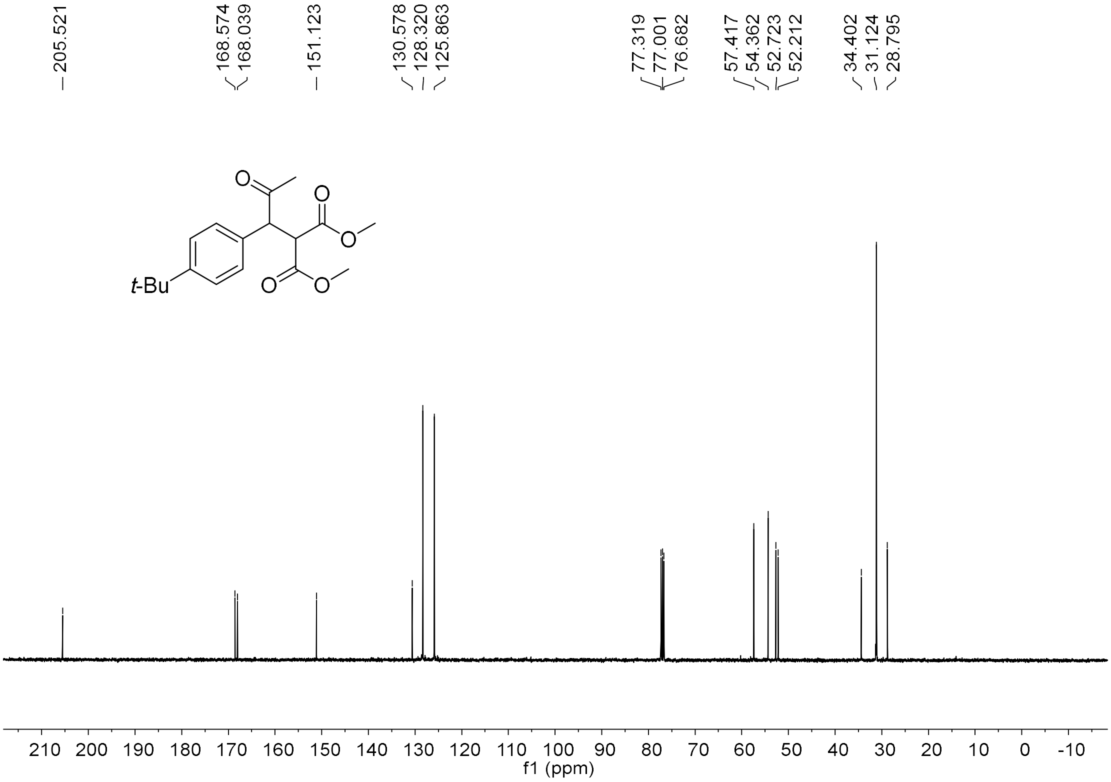

400 MHz ^{1}H NMR Spectrum of 35 in $CDCl_3$

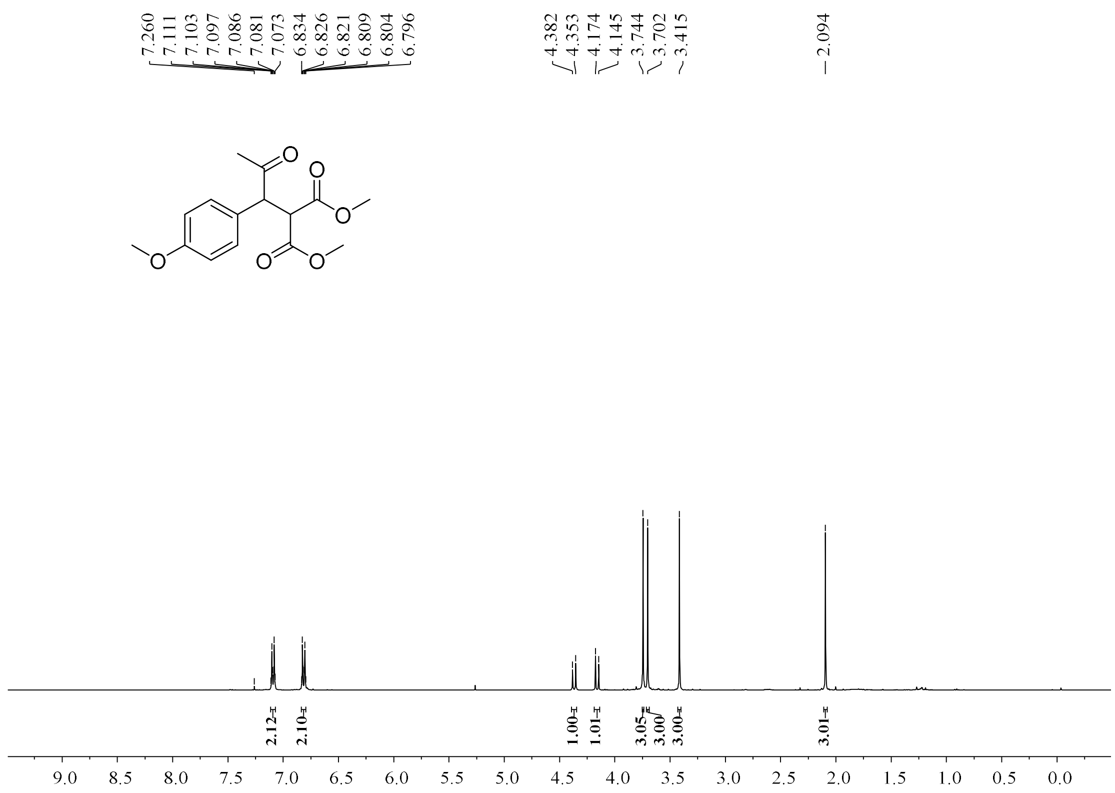

100 MHz $^{13}C\{1H\}$ NMR Spectrum of 36 in $CDCl_3$

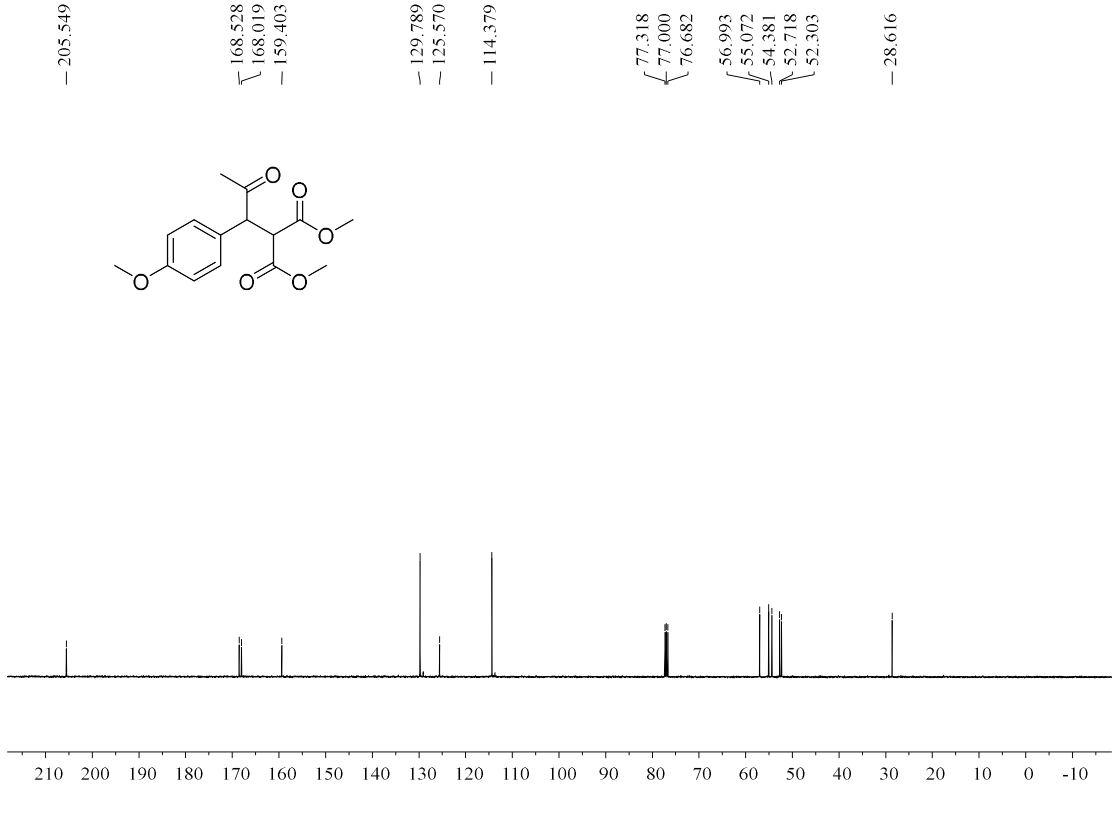

400 MHz ¹H NMR Spectrum of 36 in CDCl$_3$

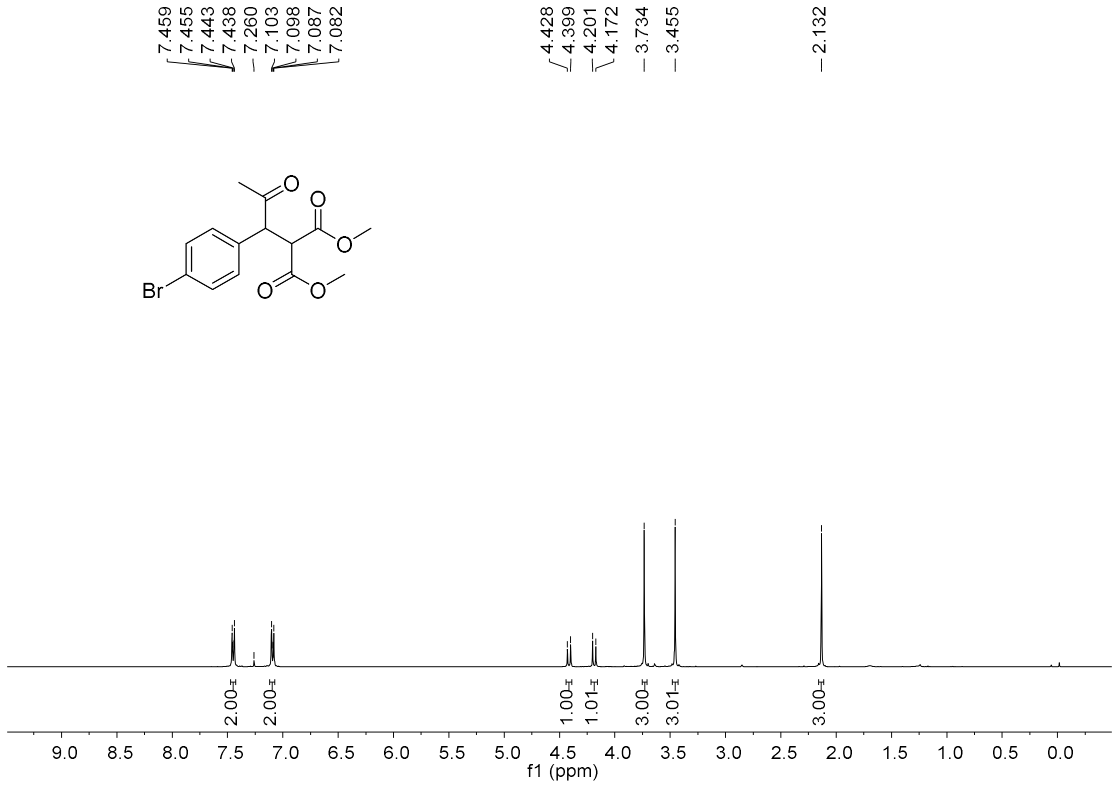

100 MHz ¹³C{1H} NMR Spectrum of 36 in CDCl$_3$

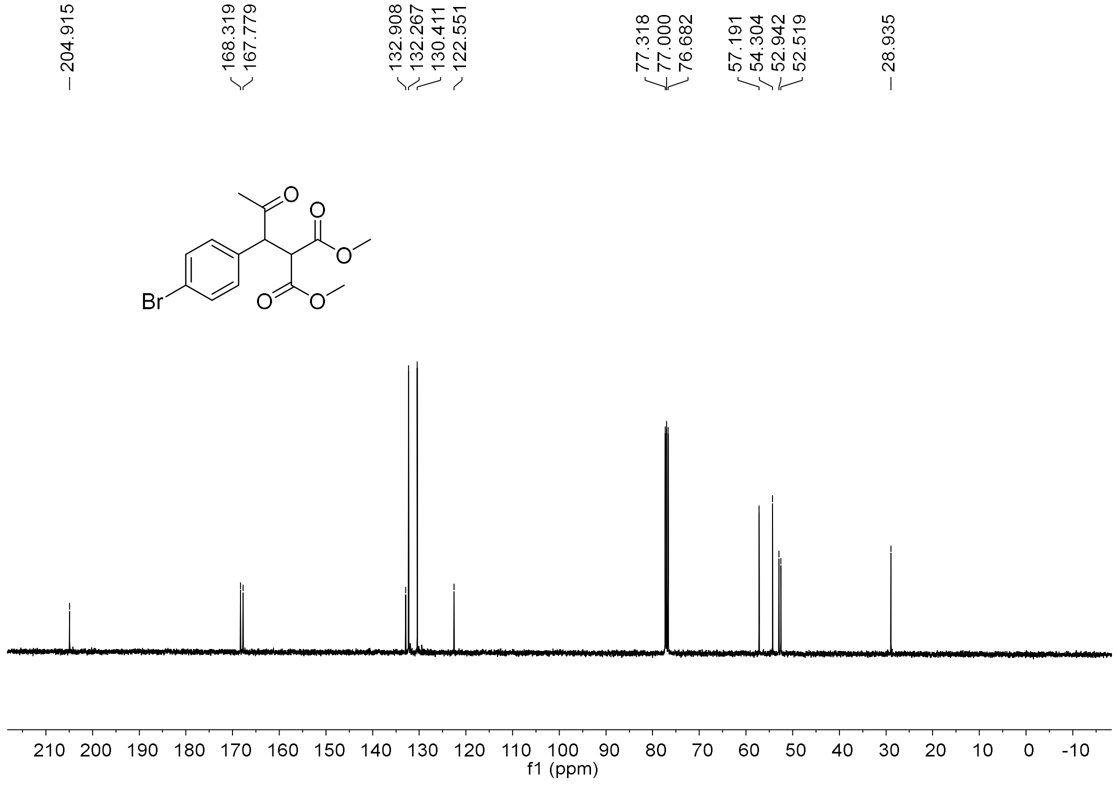

400 MHz ^{1}H NMR Spectrum of 37 in $CDCl_3$

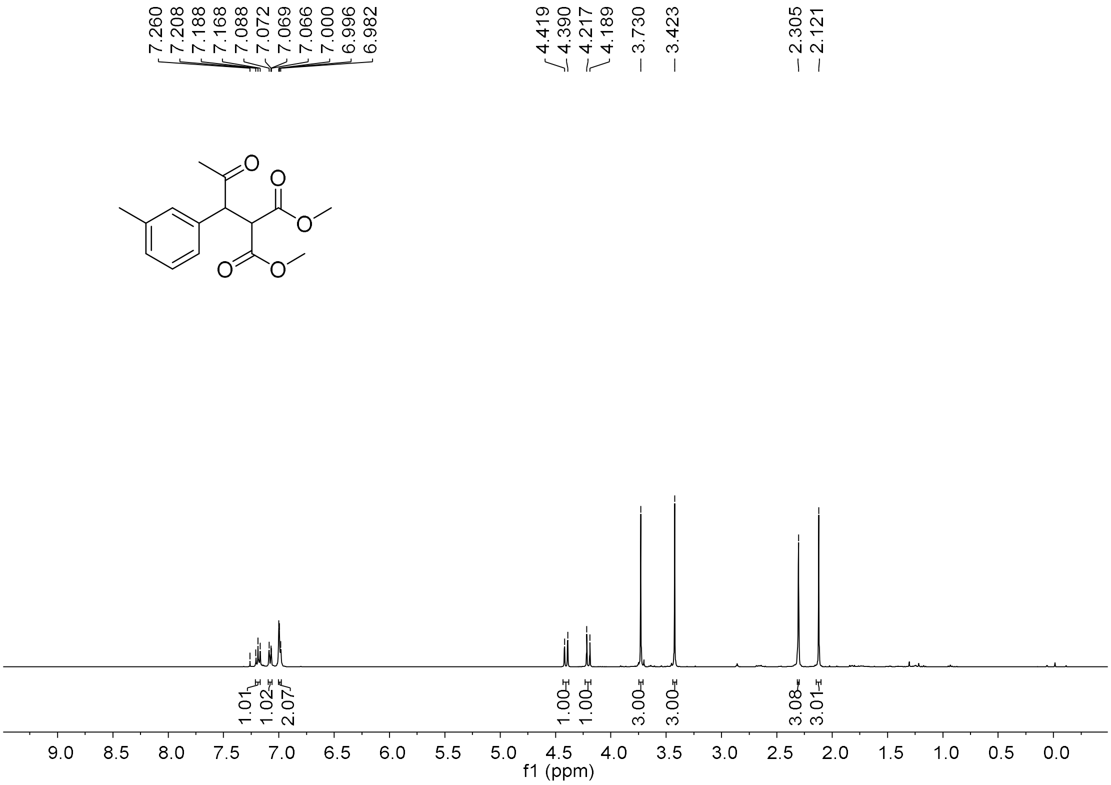

100 MHz $^{13}C\{1H\}$ NMR Spectrum of 37 in $CDCl_3$

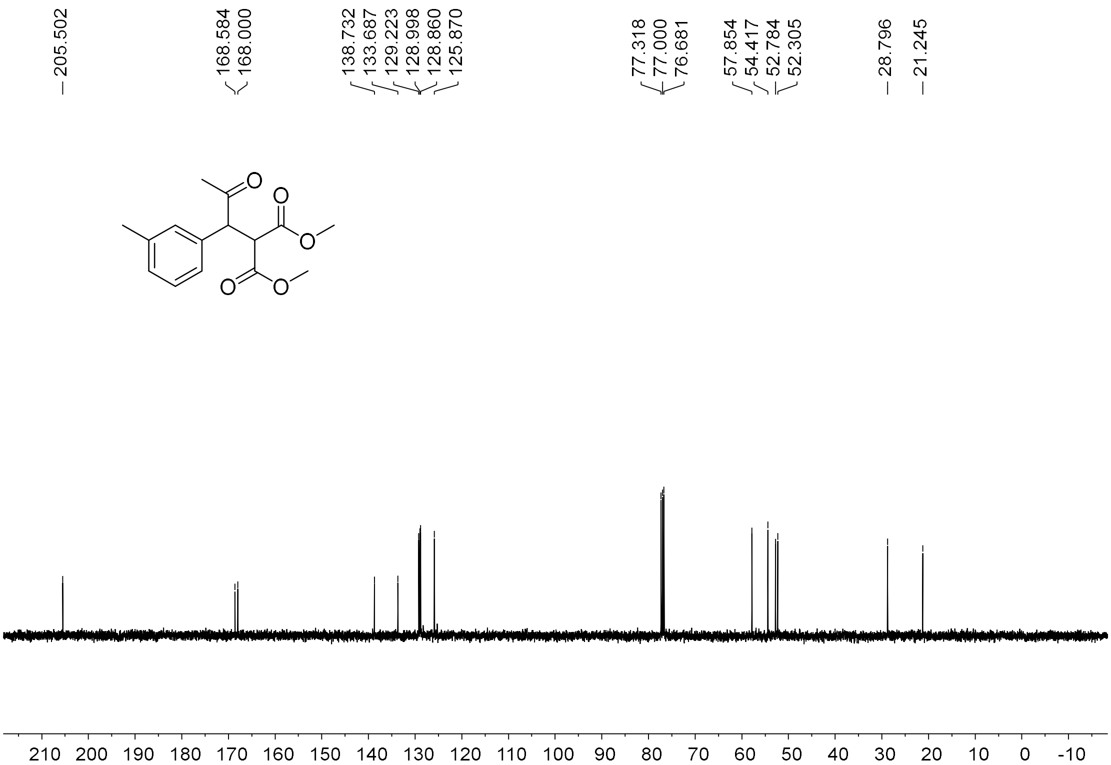

400 MHz ^{1}H NMR Spectrum of 38 in $CDCl_3$

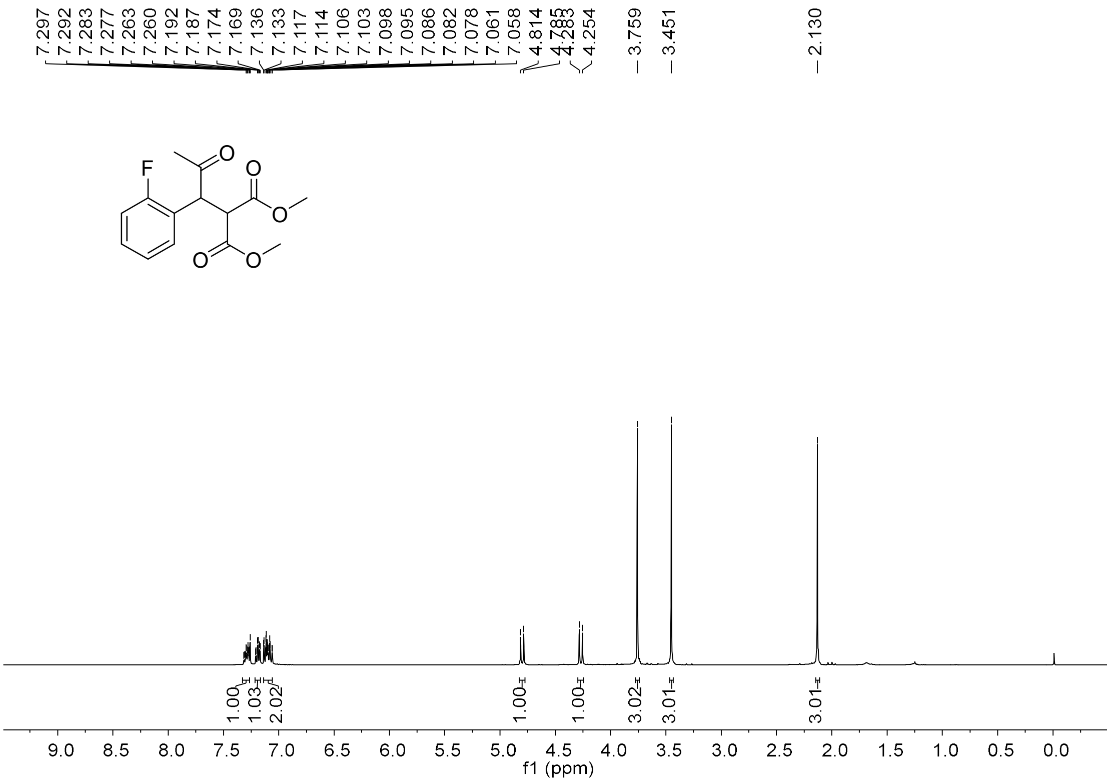

100 MHz ^{13}C{1H} NMR Spectrum of 38 in $CDCl_3$

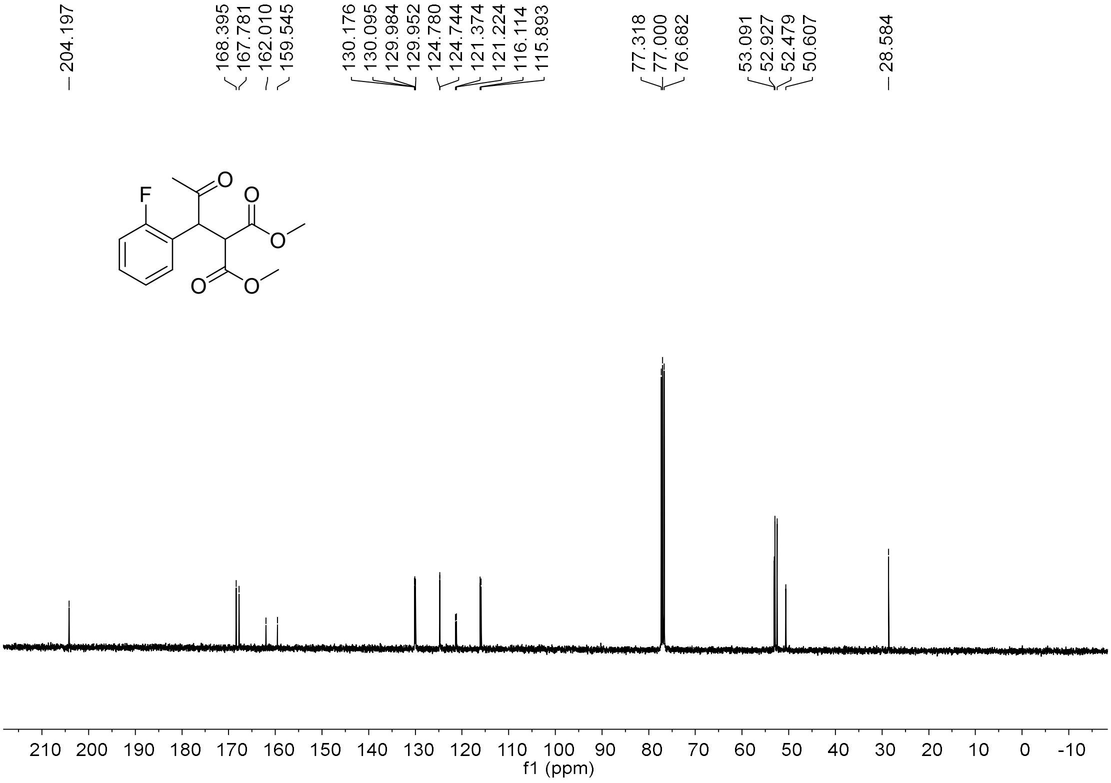

400 MHz ^{1}H NMR Spectrum of 39 in $CDCl_3$

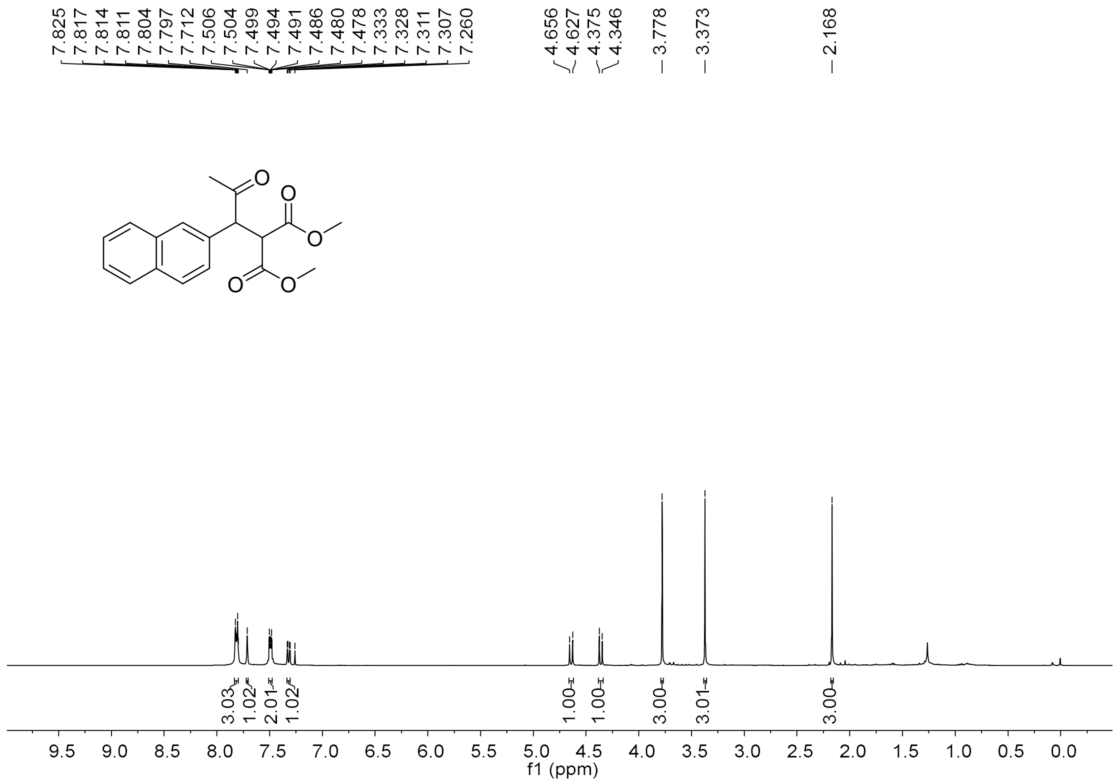

100 MHz $^{13}C\{1H\}$ NMR Spectrum of 39 in $CDCl_3$

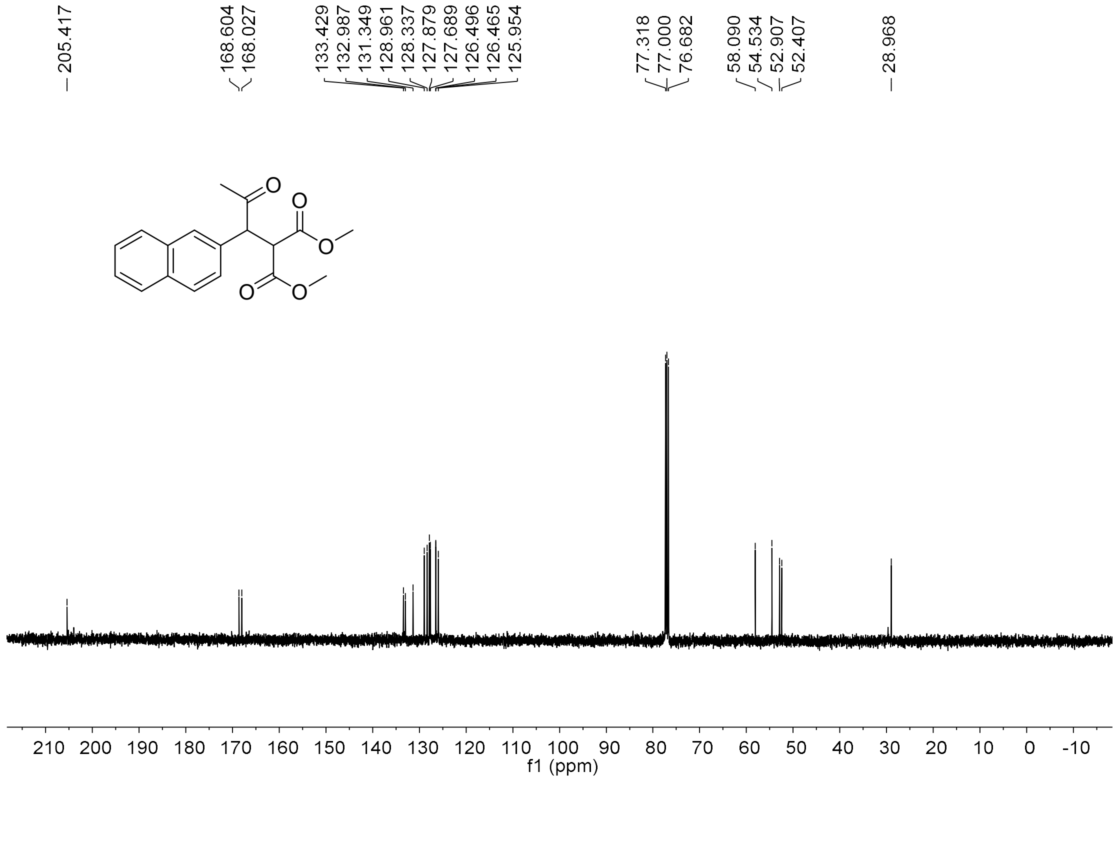

400 MHz ^{1}H NMR Spectrum of 40 in $CDCl_3$

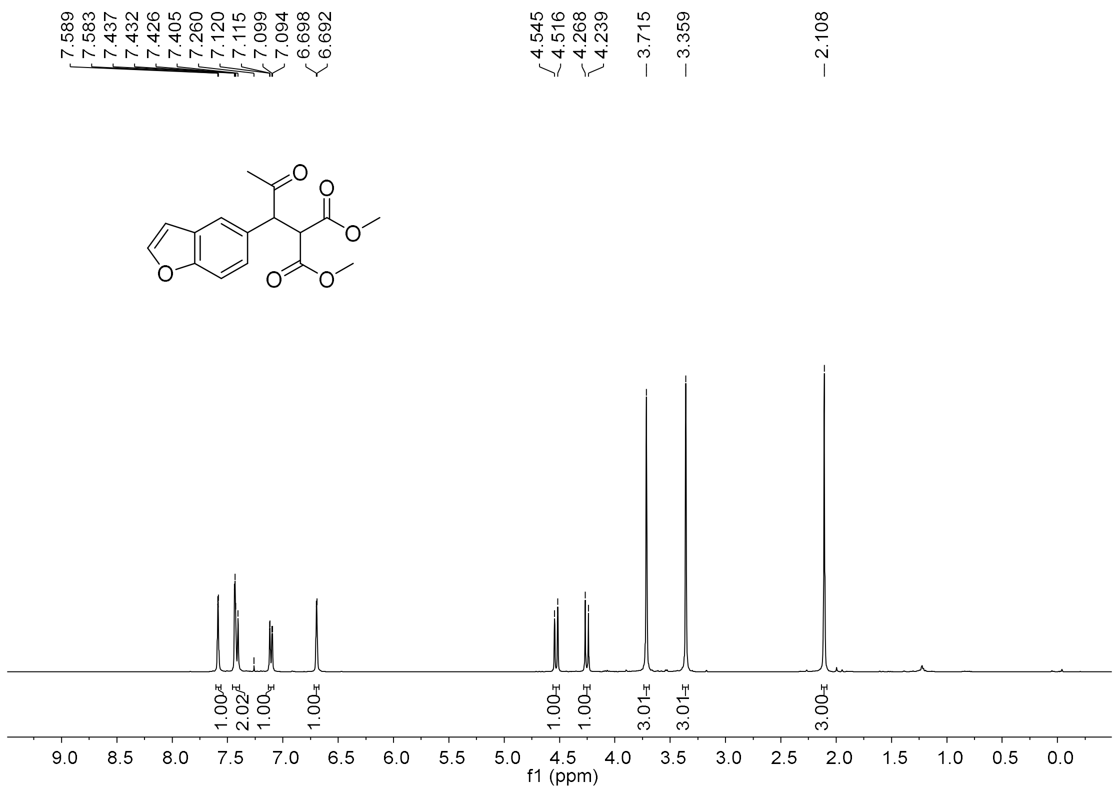

100 MHz ^{13}C{1H} NMR Spectrum of 40 in $CDCl_3$

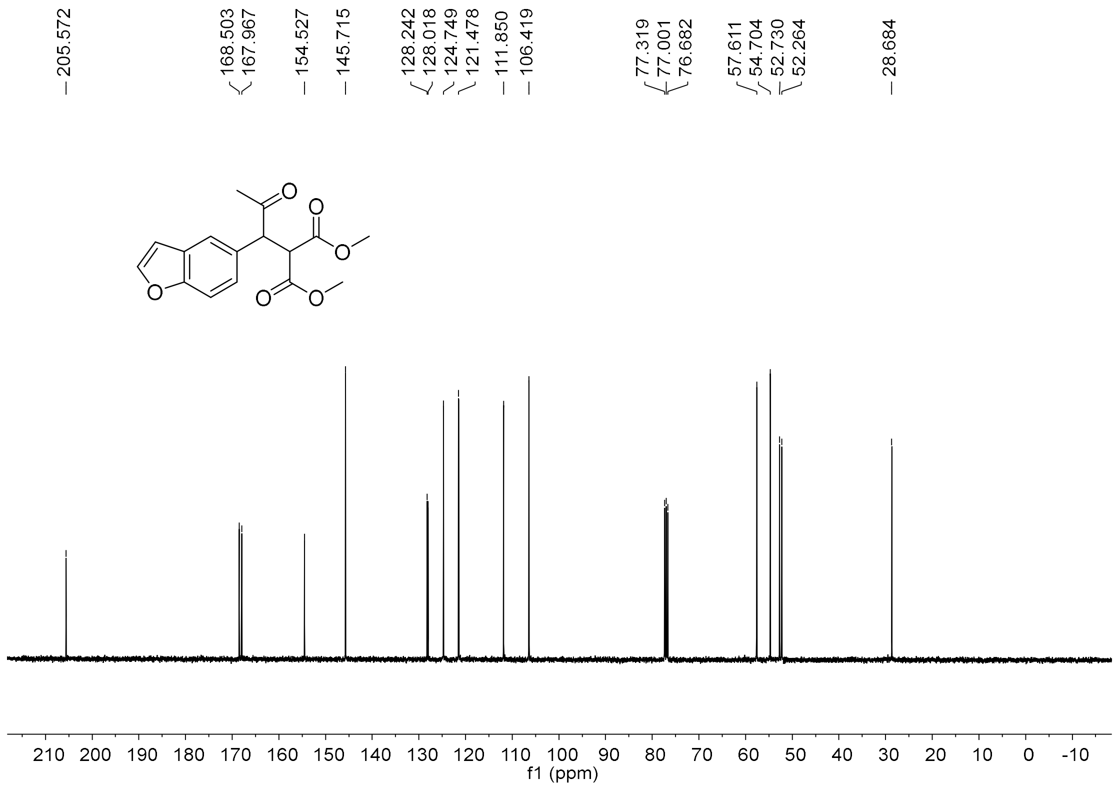

400 MHz ^{1}H NMR Spectrum of 41 in $CDCl_3$

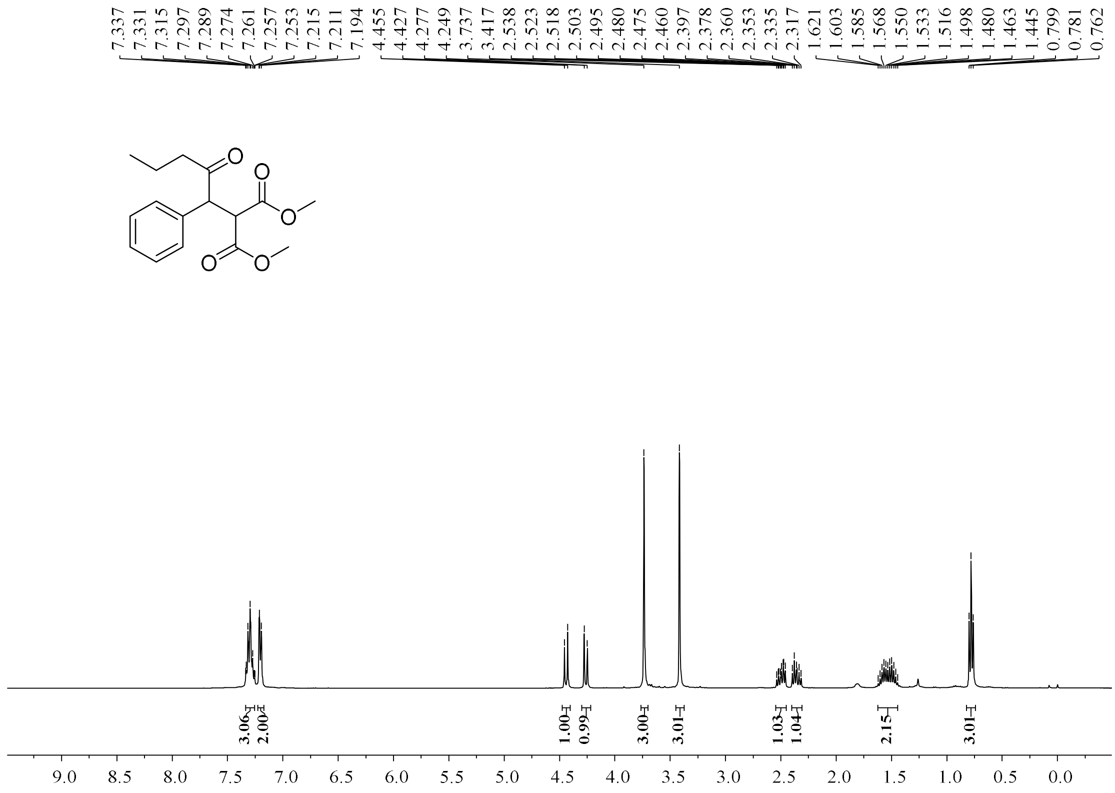

100 MHz $^{13}C\{1H\}$ NMR Spectrum of 41 in $CDCl_3$

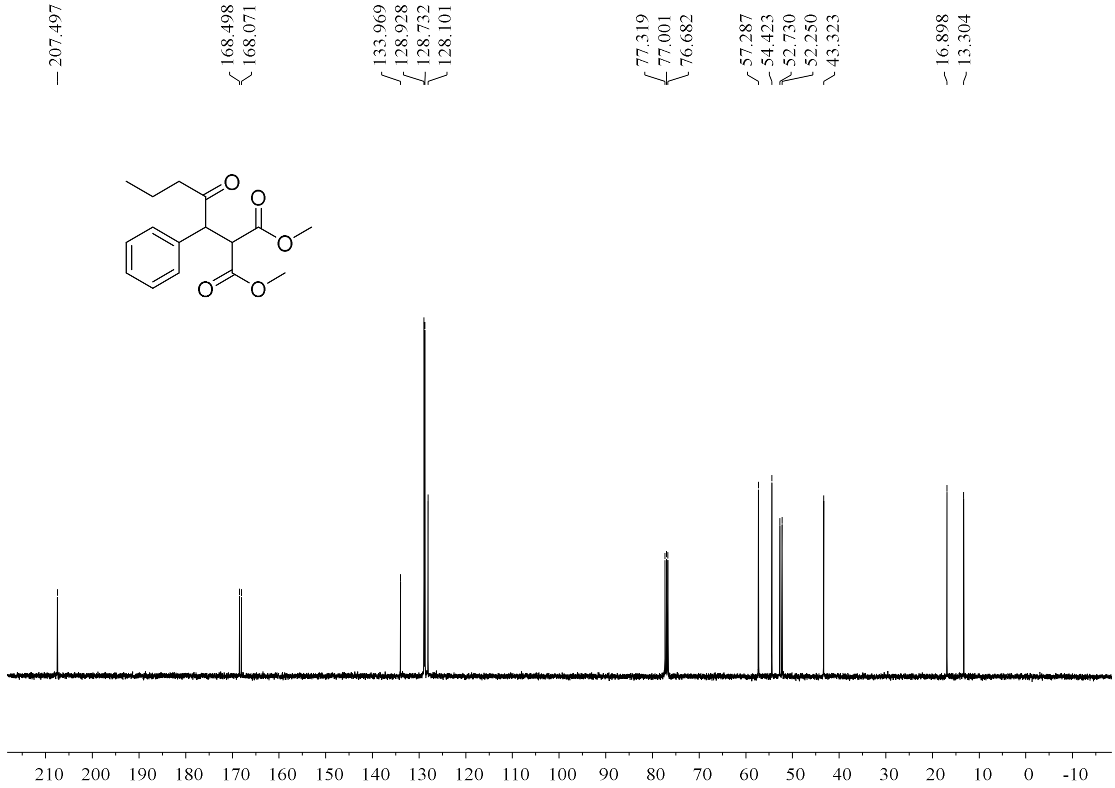

400 MHz ^{1}H NMR Spectrum of 42 in DMSO-d_6

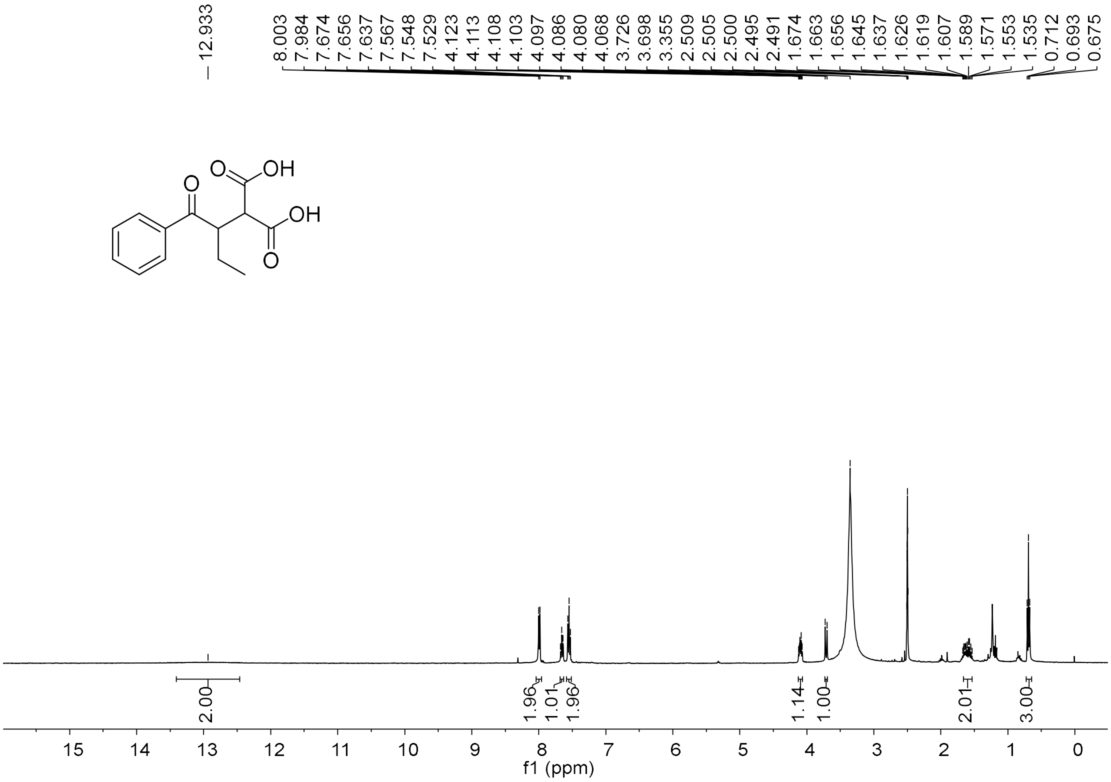

100 MHz ^{13}C{1H} NMR Spectrum of 42 in DMSO-d_6

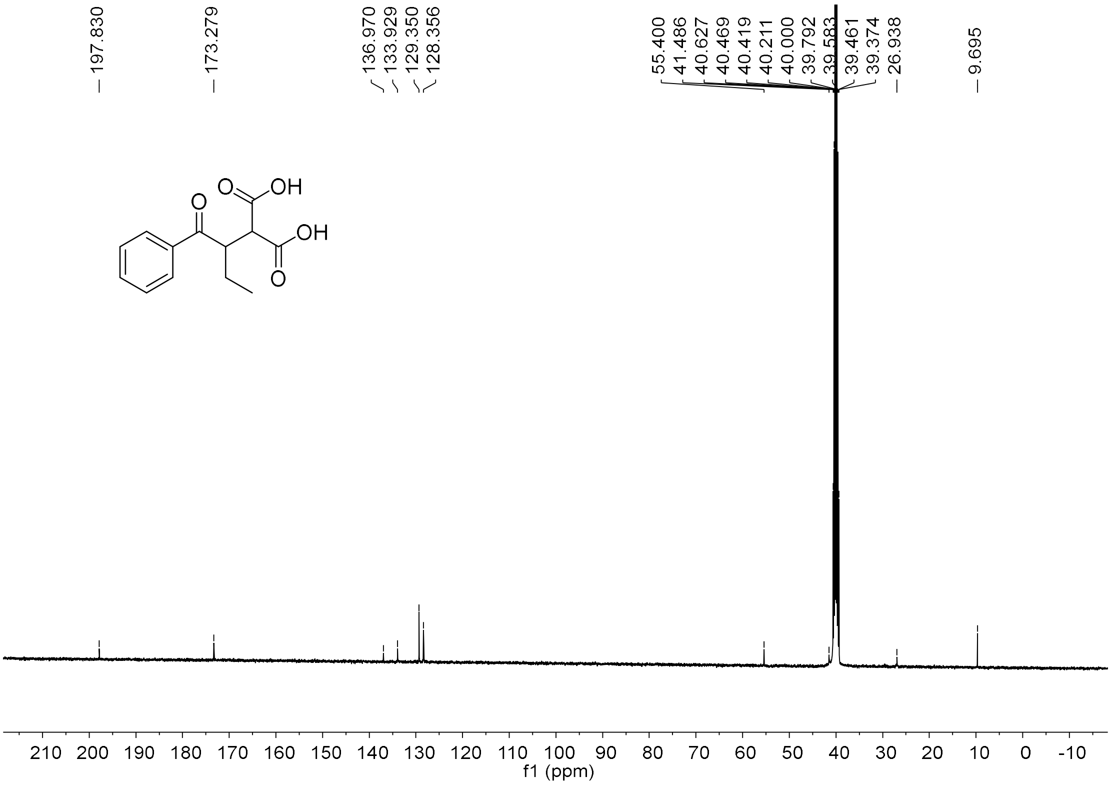

400 MHz ^{1}H NMR Spectrum of 43 in DMSO-d_6

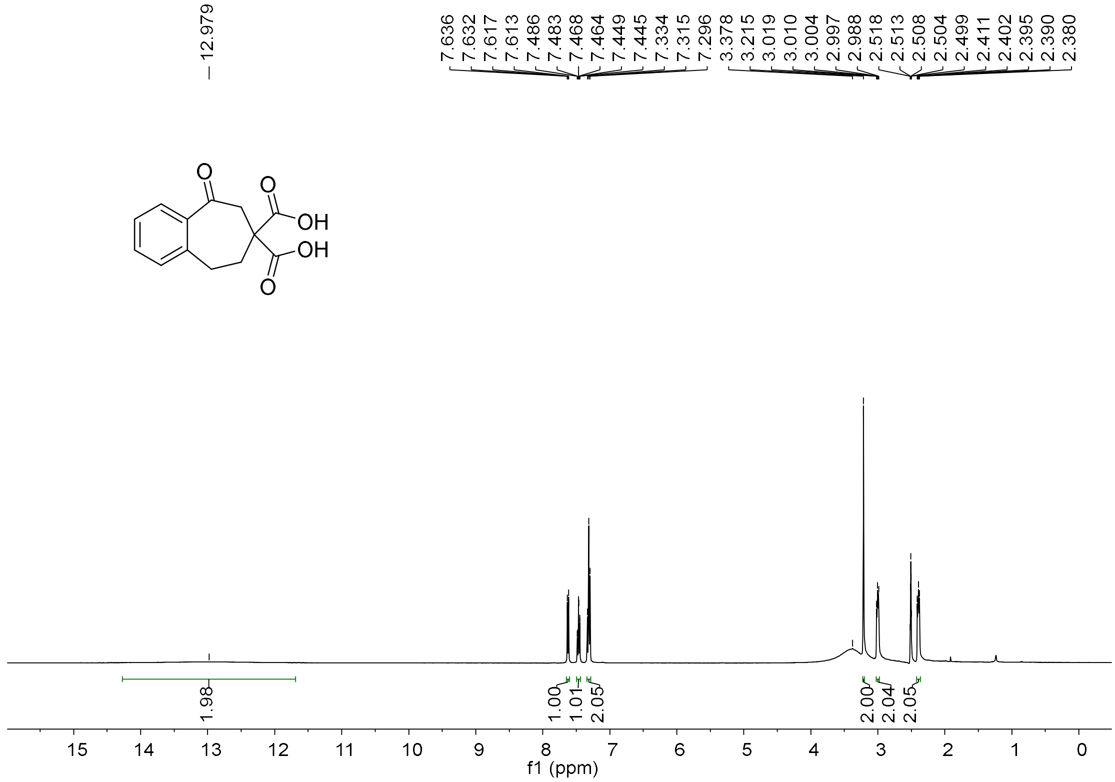

100 MHz ^{13}C{1H} NMR Spectrum of 43 in DMSO-d_6

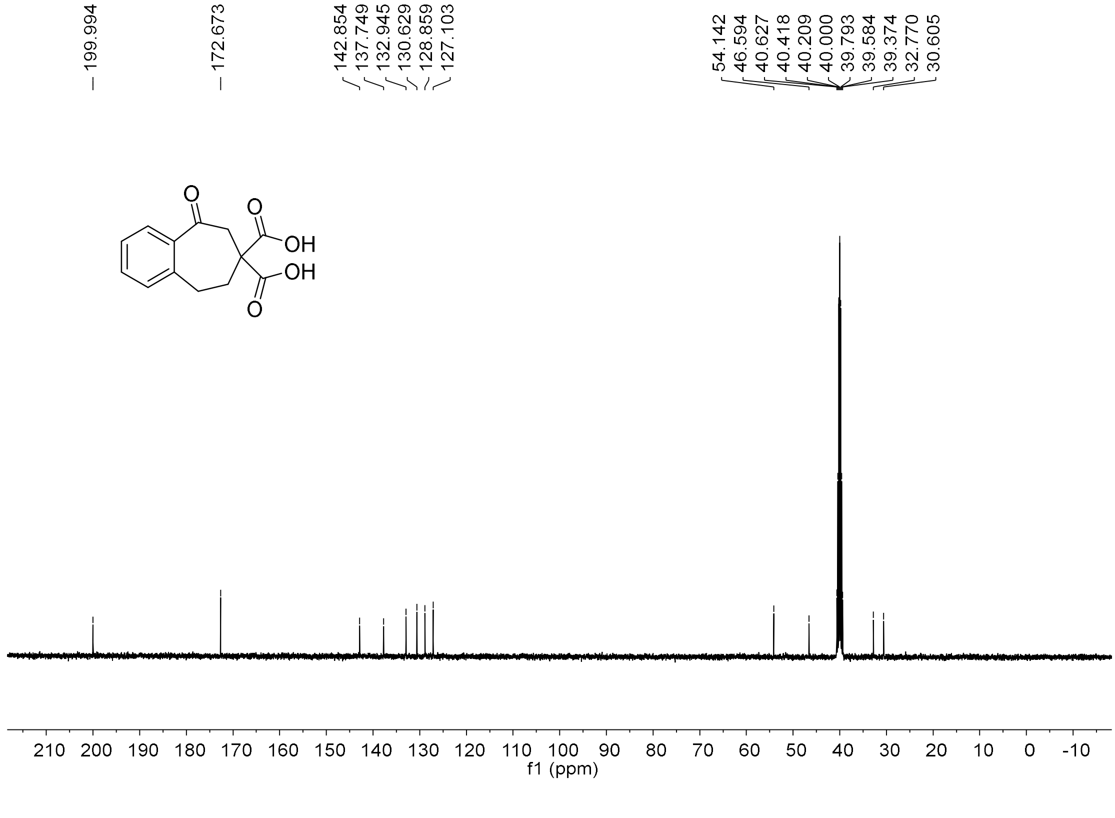

400 MHz ^{1}H NMR Spectrum of 44 in DMSO-d_6

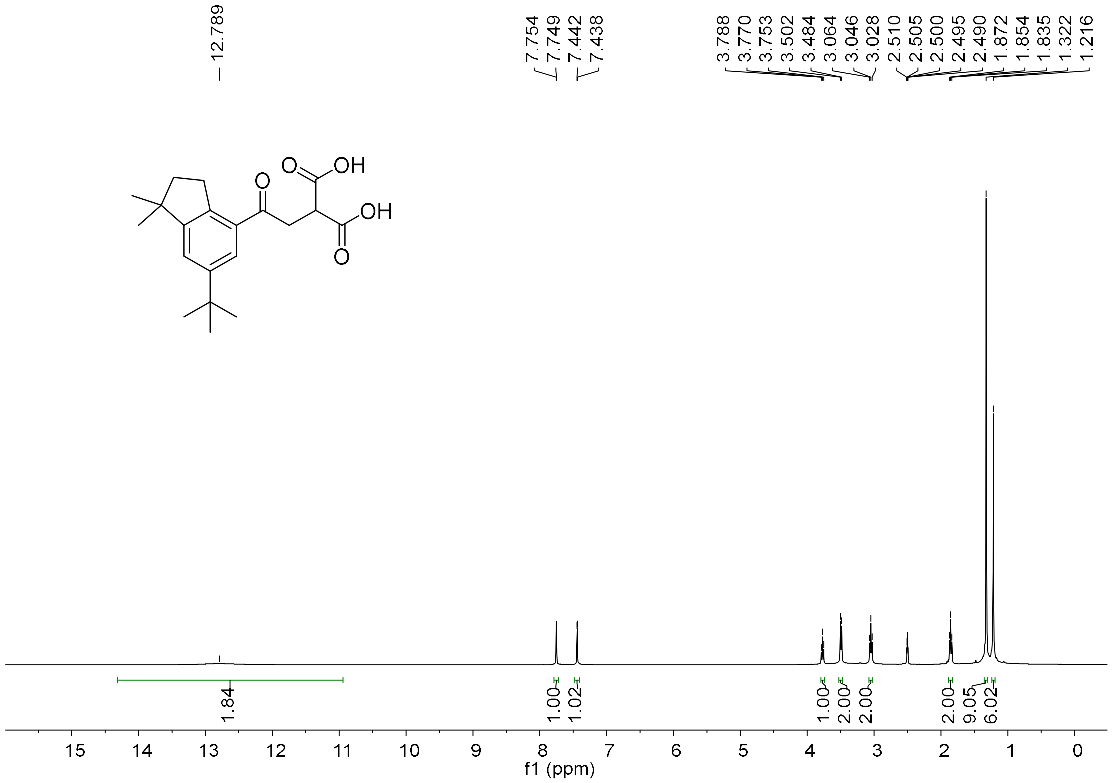

100 MHz ^{13}C{1H} NMR Spectrum of 44 in DMSO-d_6

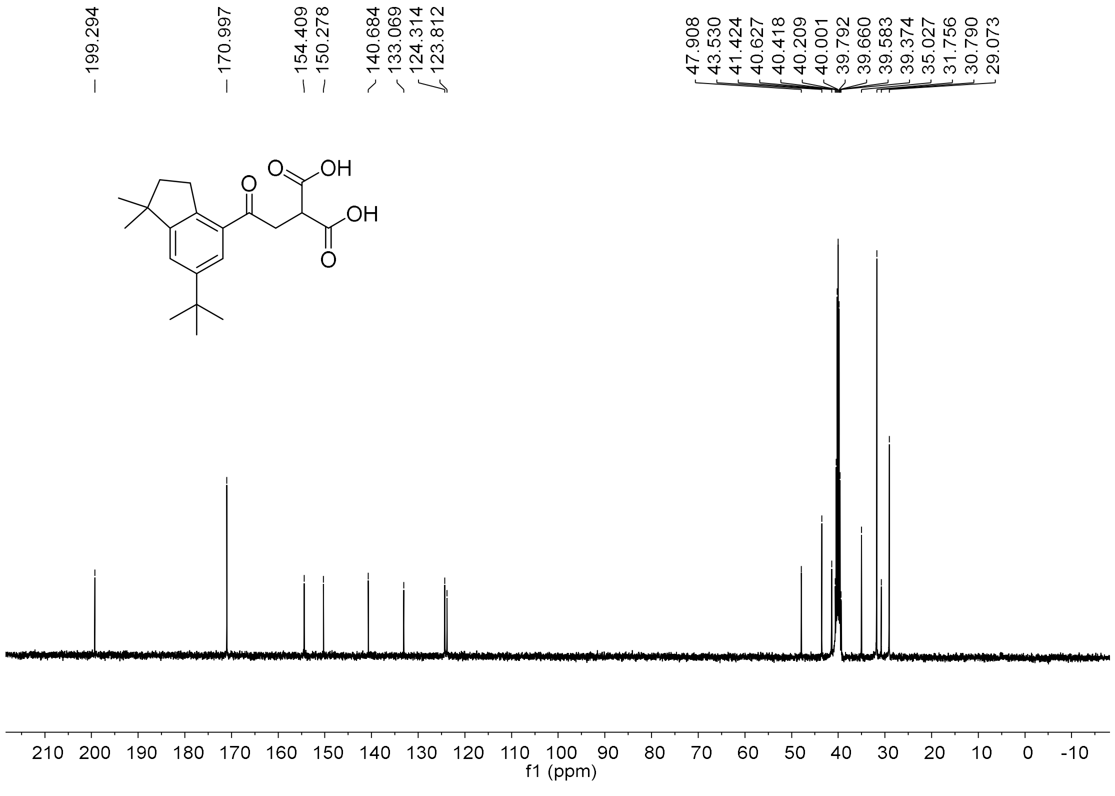

400 MHz ¹H NMR Spectrum of 45 in DMSO-d_6

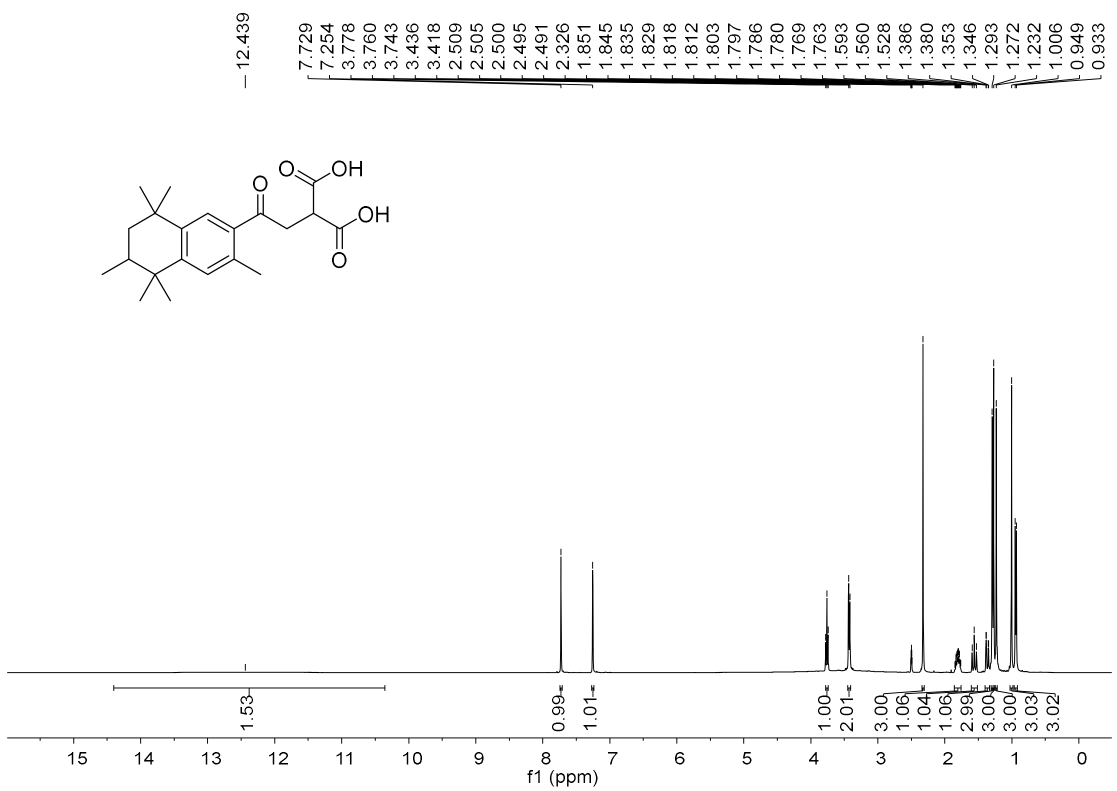

100 MHz ¹³C{1H} NMR Spectrum of 45 in DMSO-d_6

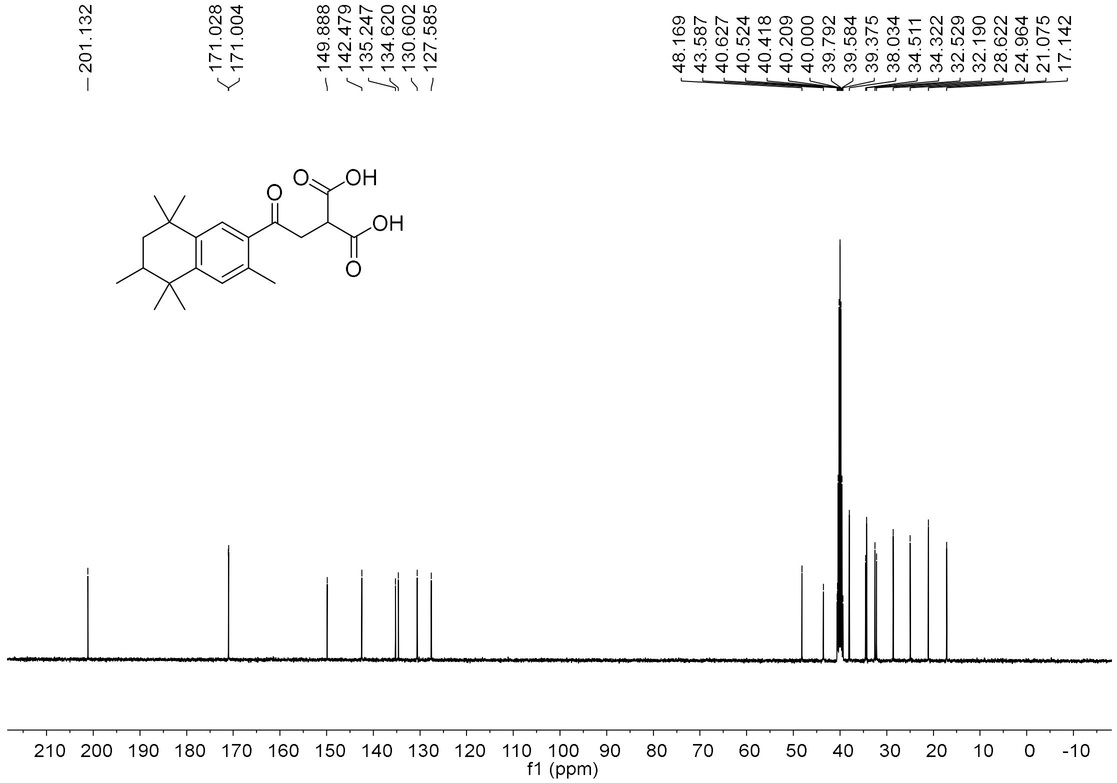

400 MHz ^{1}H NMR Spectrum of 46 in DMSO-d_6

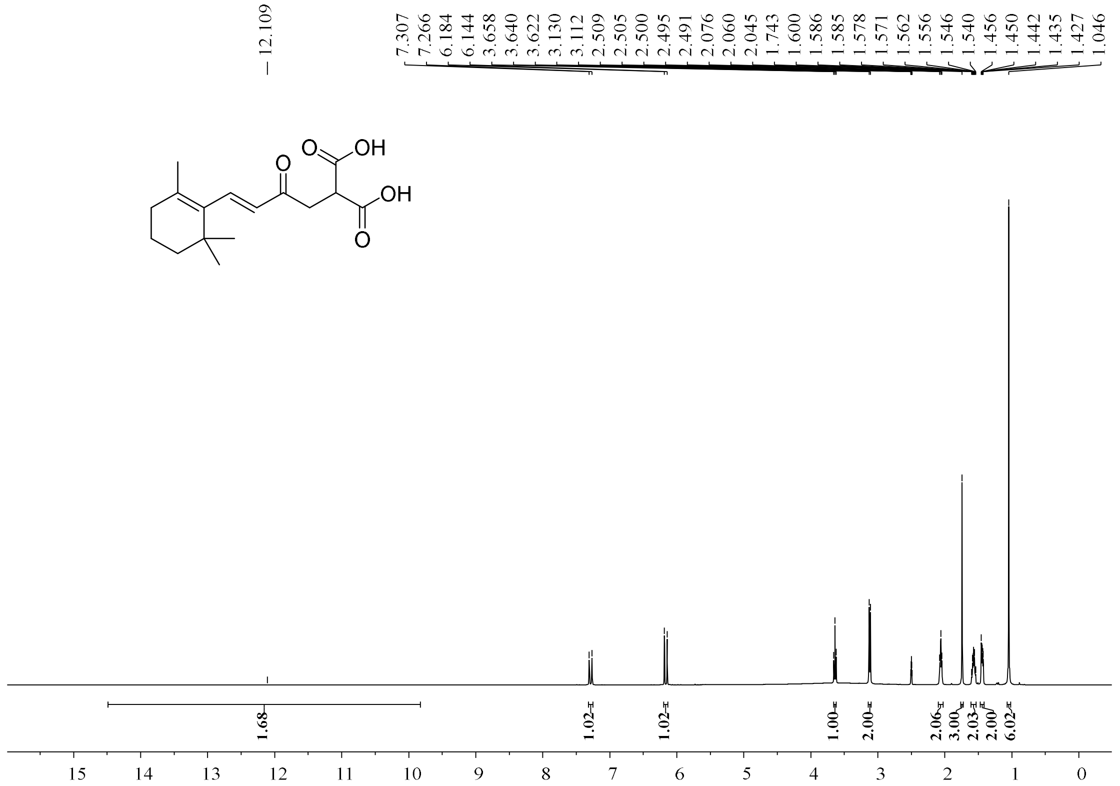

100 MHz $^{13}C\{1H\}$ NMR Spectrum of 46 in DMSO-d_6

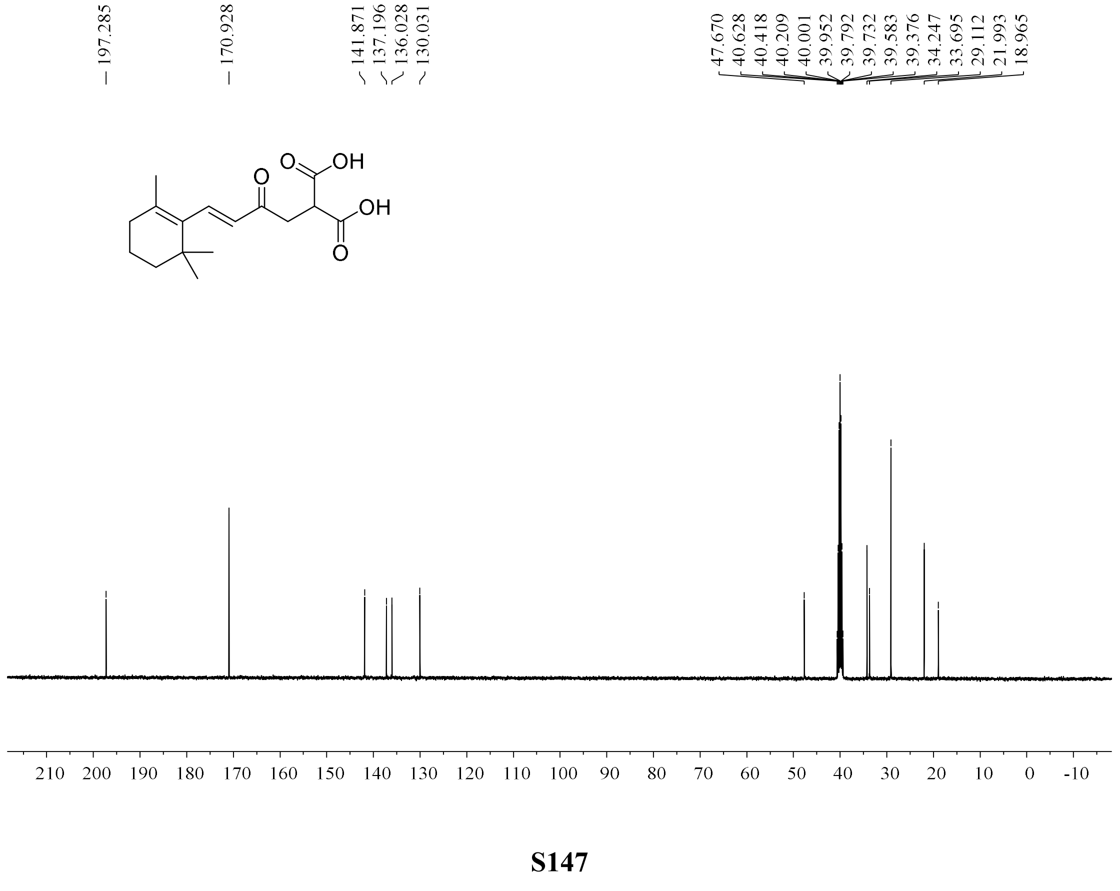

400 MHz ^{1}H NMR Spectrum of 47 in DMSO-d_6

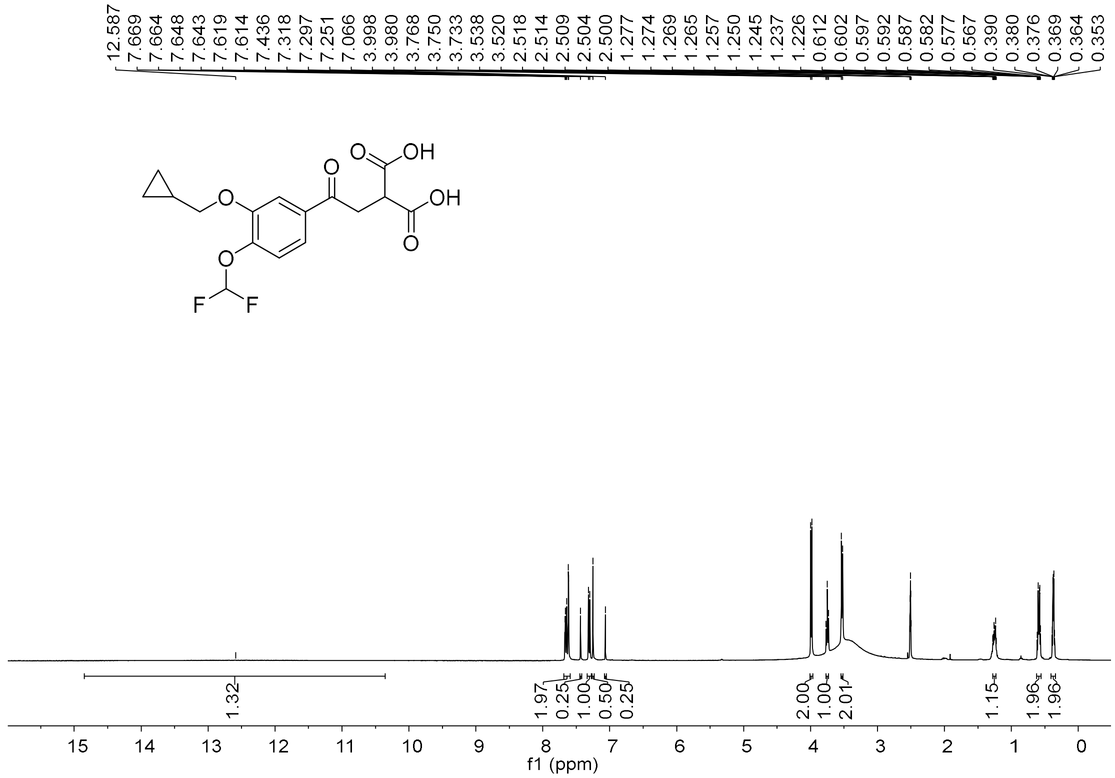

100 MHz ^{13}C{1H} NMR Spectrum of 47 in DMSO-d_6

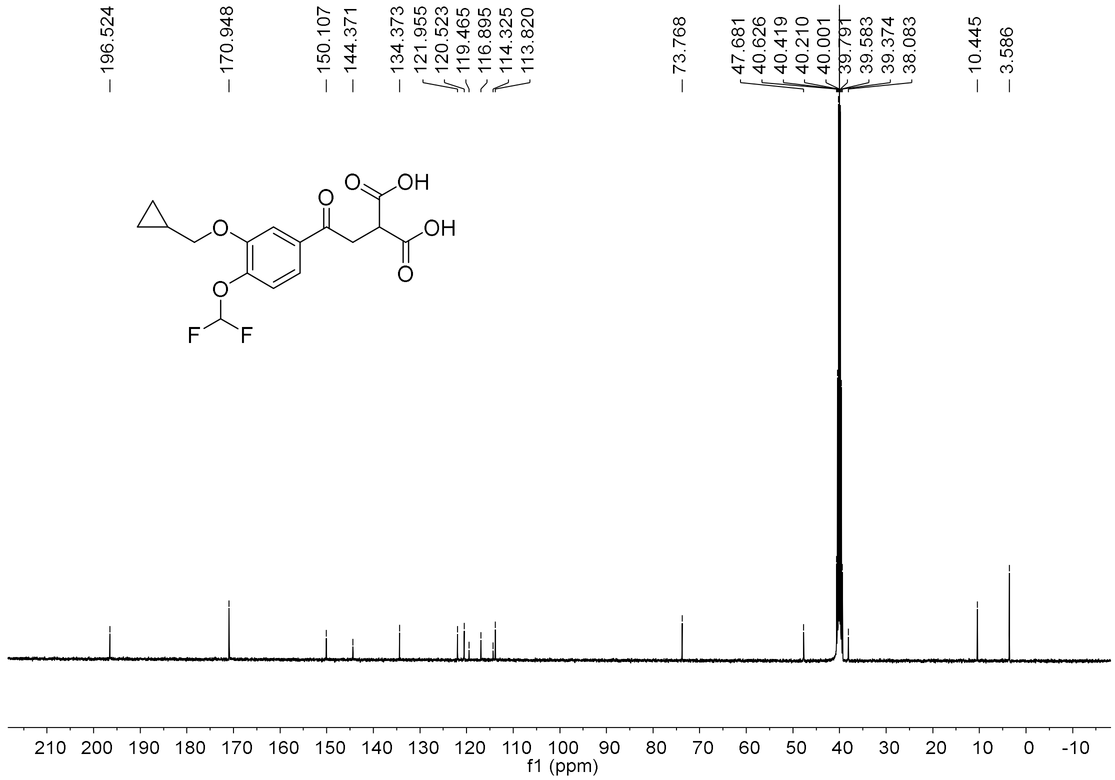

400 MHz ^{1}H NMR Spectrum of 48 in DMSO-d_6

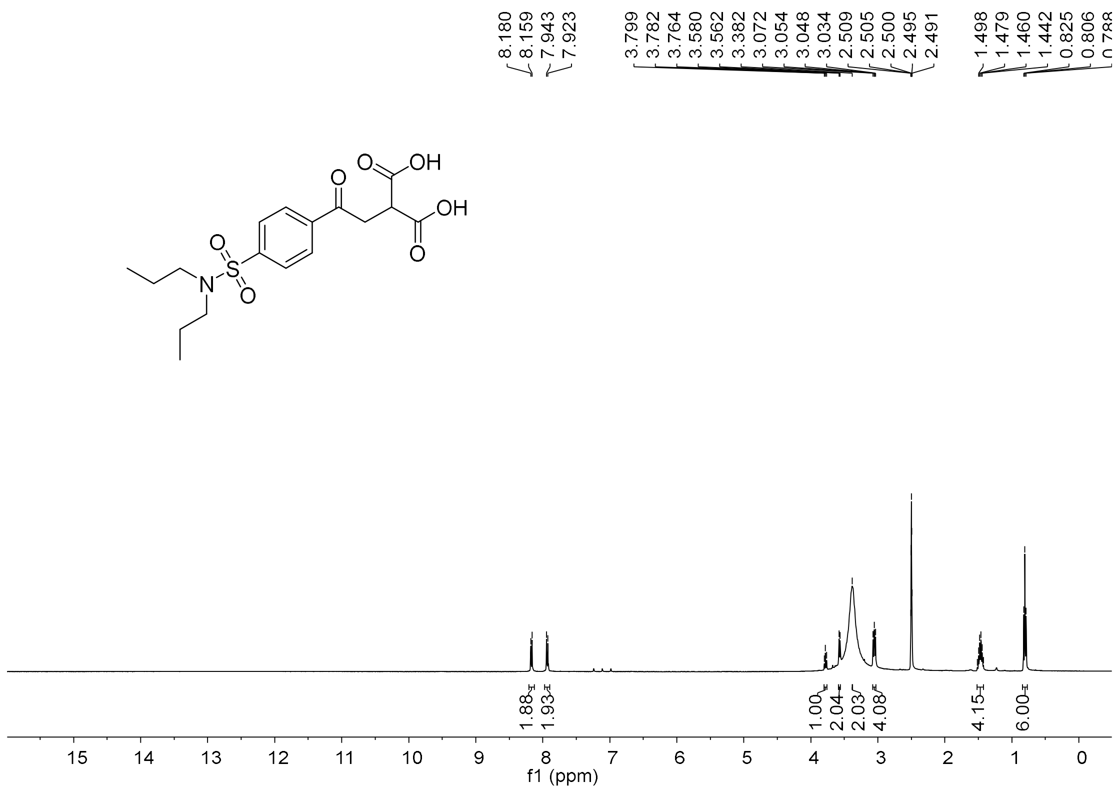

100 MHz ^{13}C{1H} NMR Spectrum of 48 in DMSO-d_6

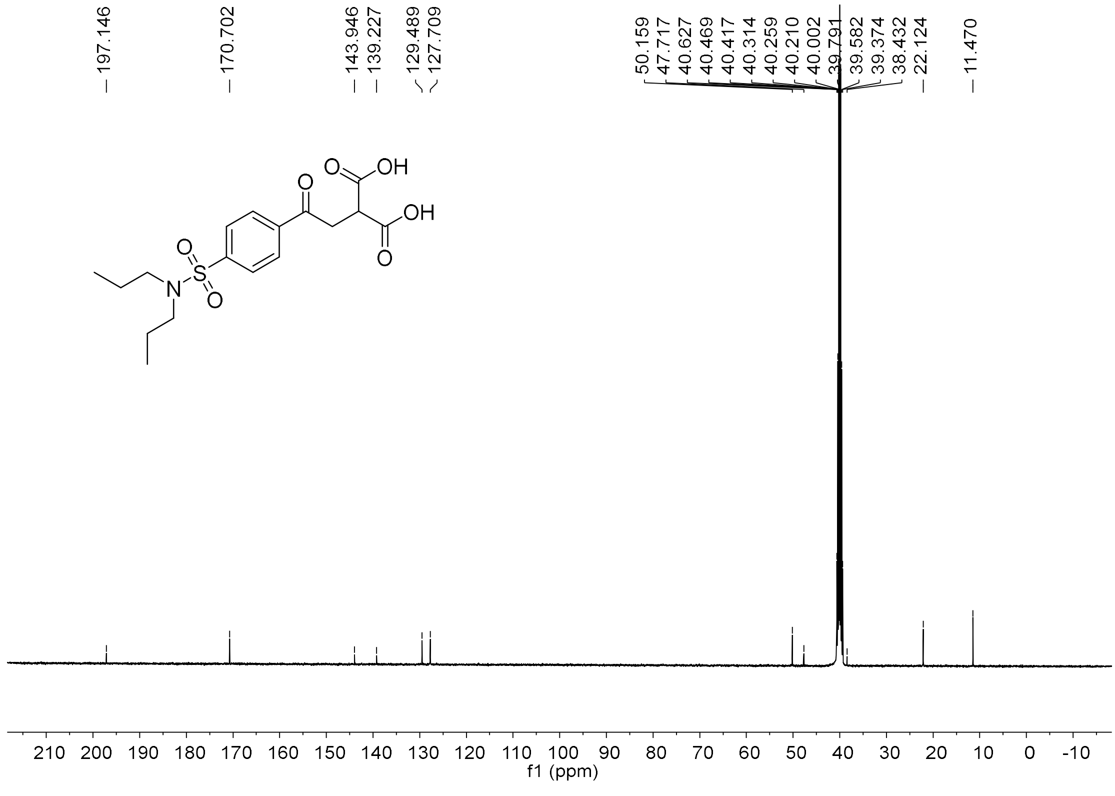

400 MHz ^{1}H NMR Spectrum of 49 in DMSO-d_6

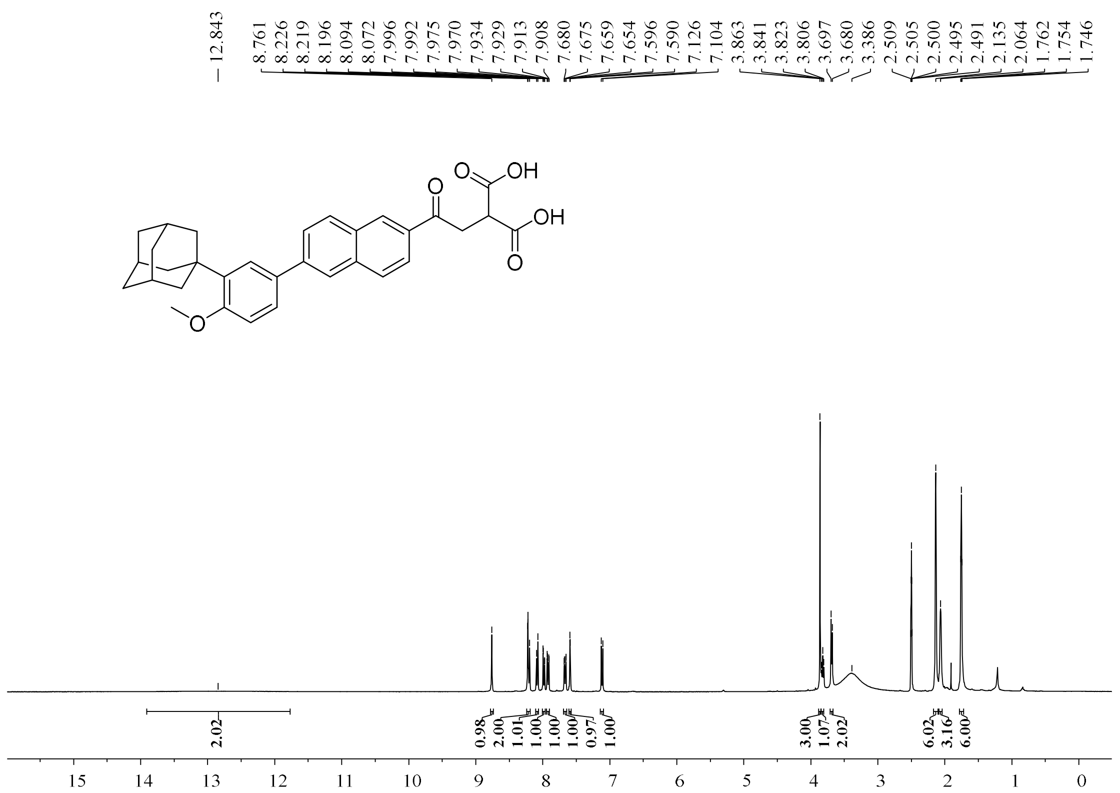

100 MHz ^{13}C{1H} NMR Spectrum of 49 in DMSO-d_6

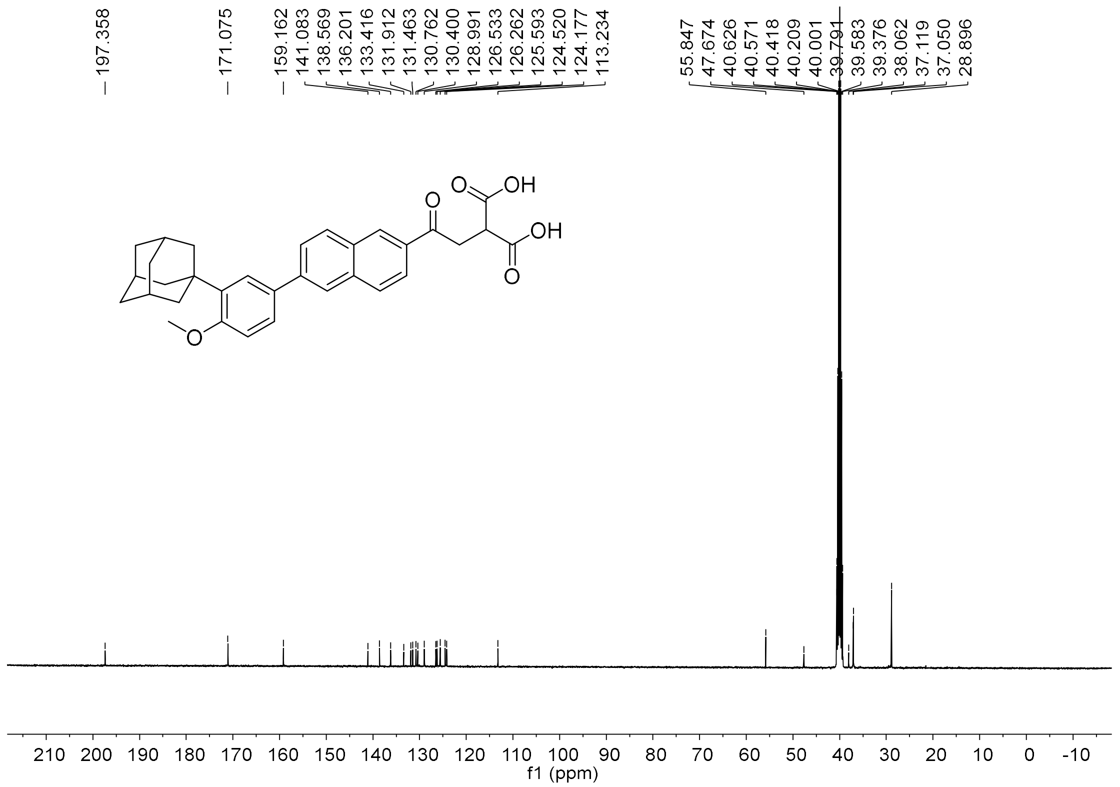

400 MHz ^{1}H NMR Spectrum of 50 in $CDCl_3$

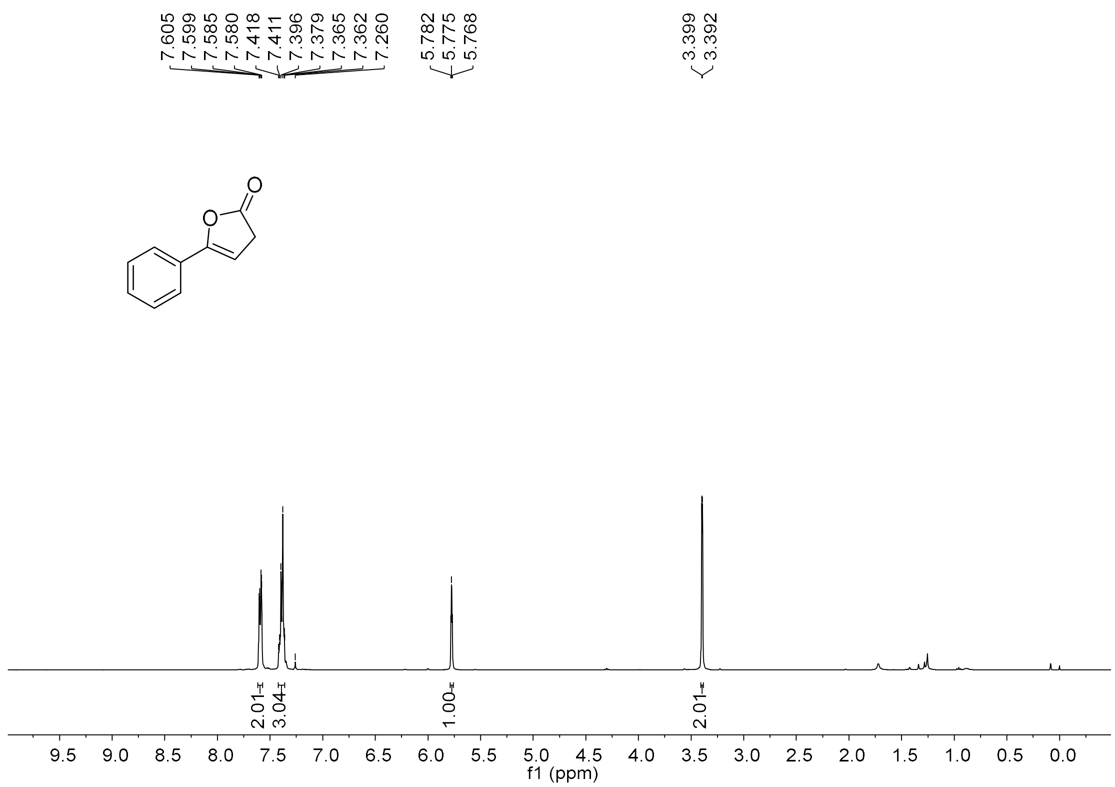

100 MHz $^{13}C\{1H\}$ NMR Spectrum of 50 in $CDCl_3$

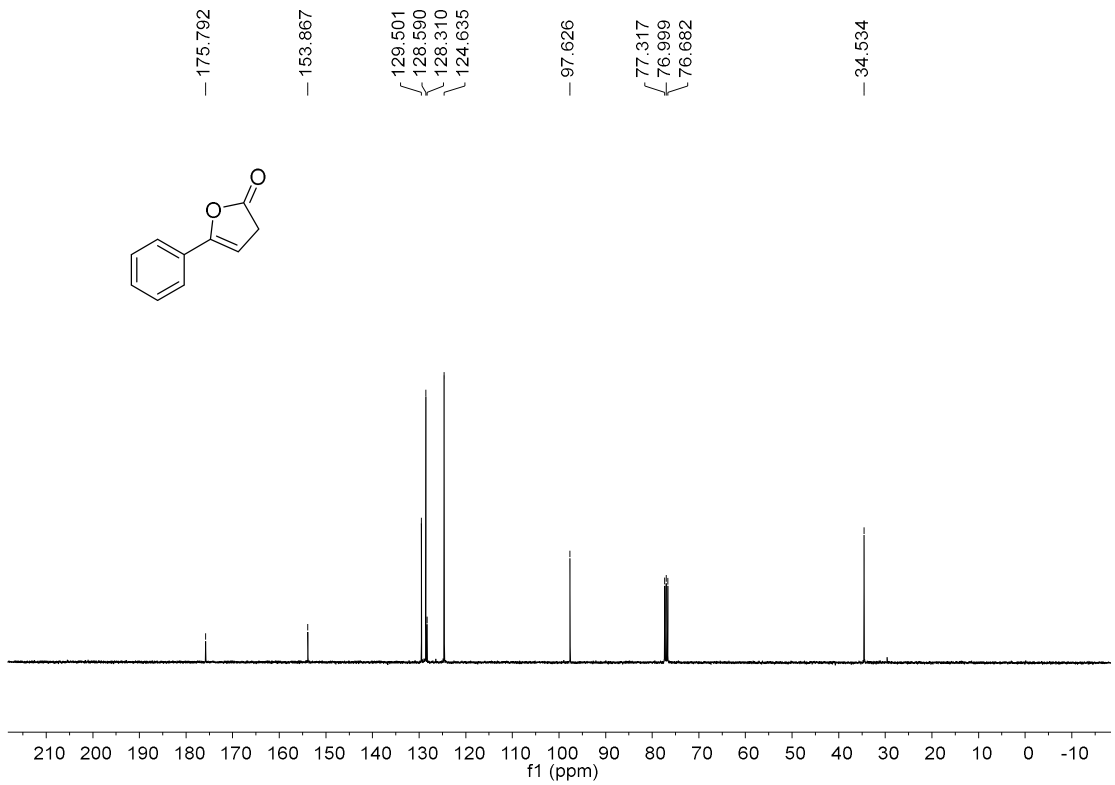

400 MHz ^{1}H NMR Spectrum of 51 in $CDCl_3$

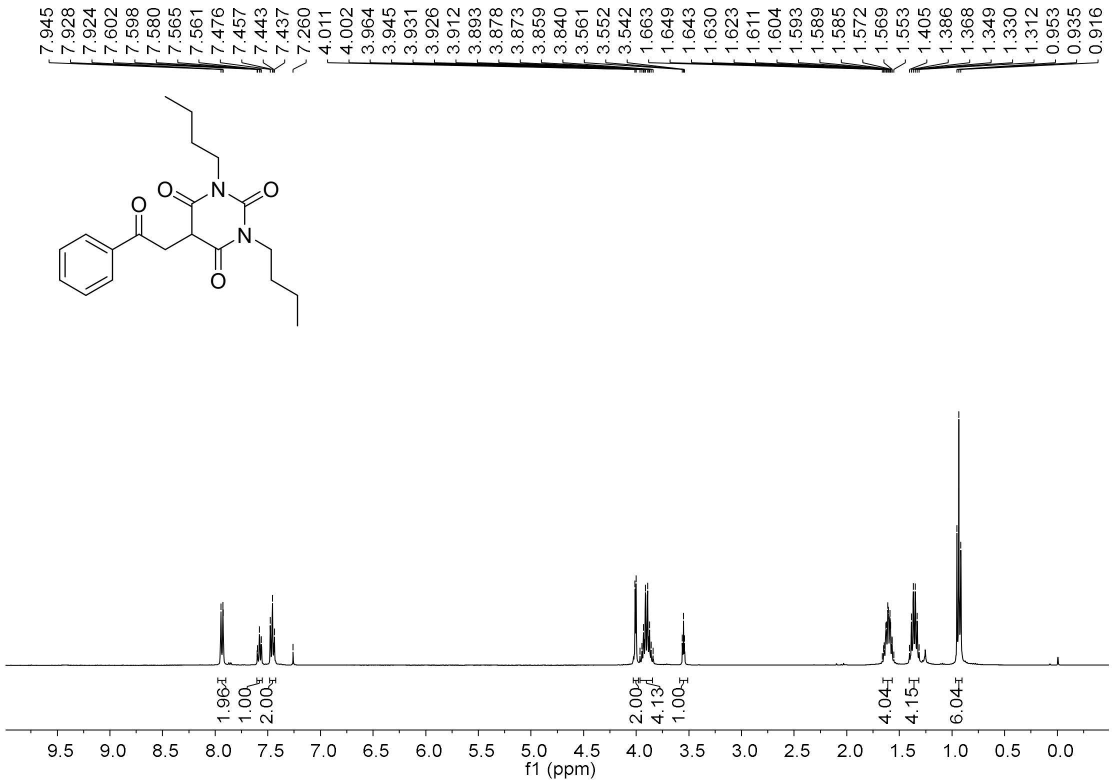

100 MHz ^{13}C{1H} NMR Spectrum of 51 in $CDCl_3$

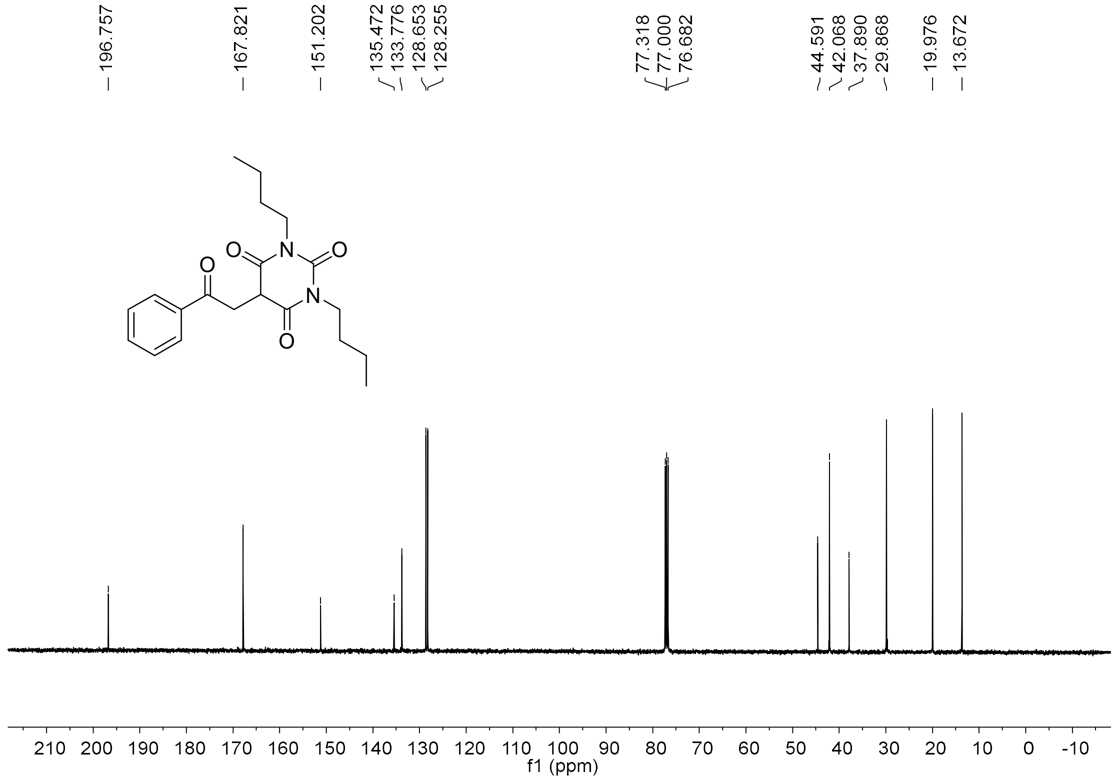

400 MHz ^{1}H NMR Spectrum of 52 in $CDCl_3$

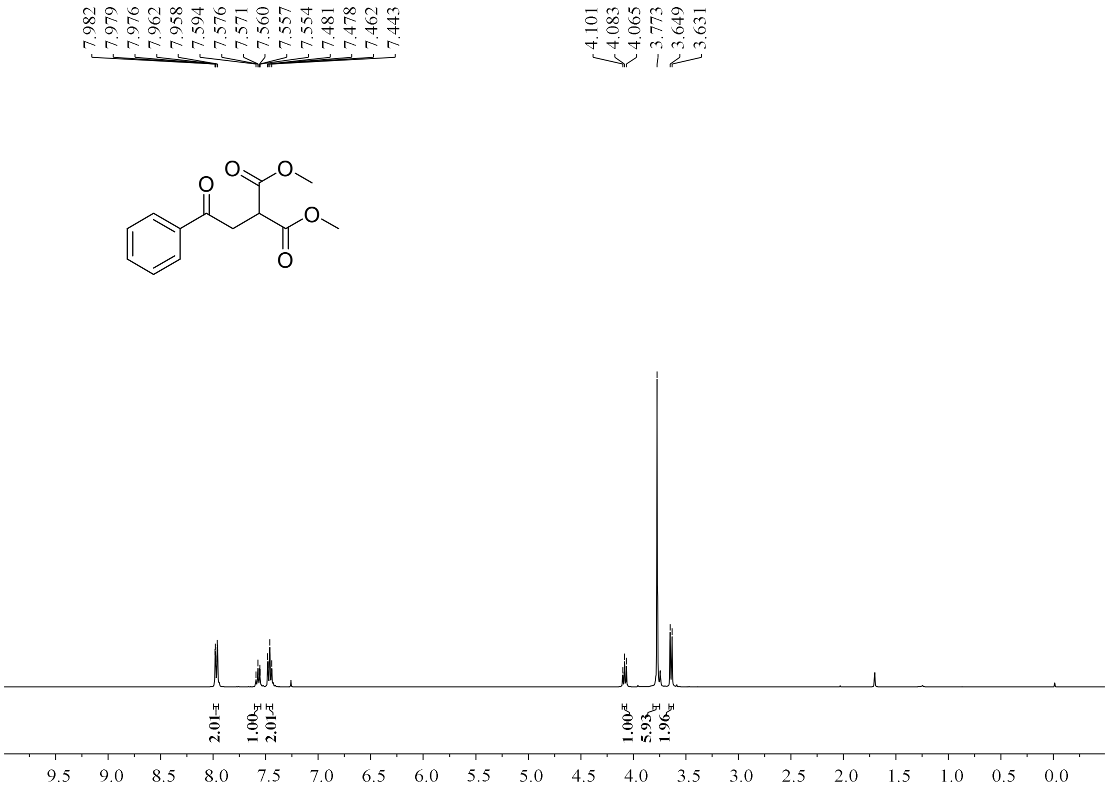

100 MHz ^{13}C{1H} NMR Spectrum of 52 in $CDCl_3$

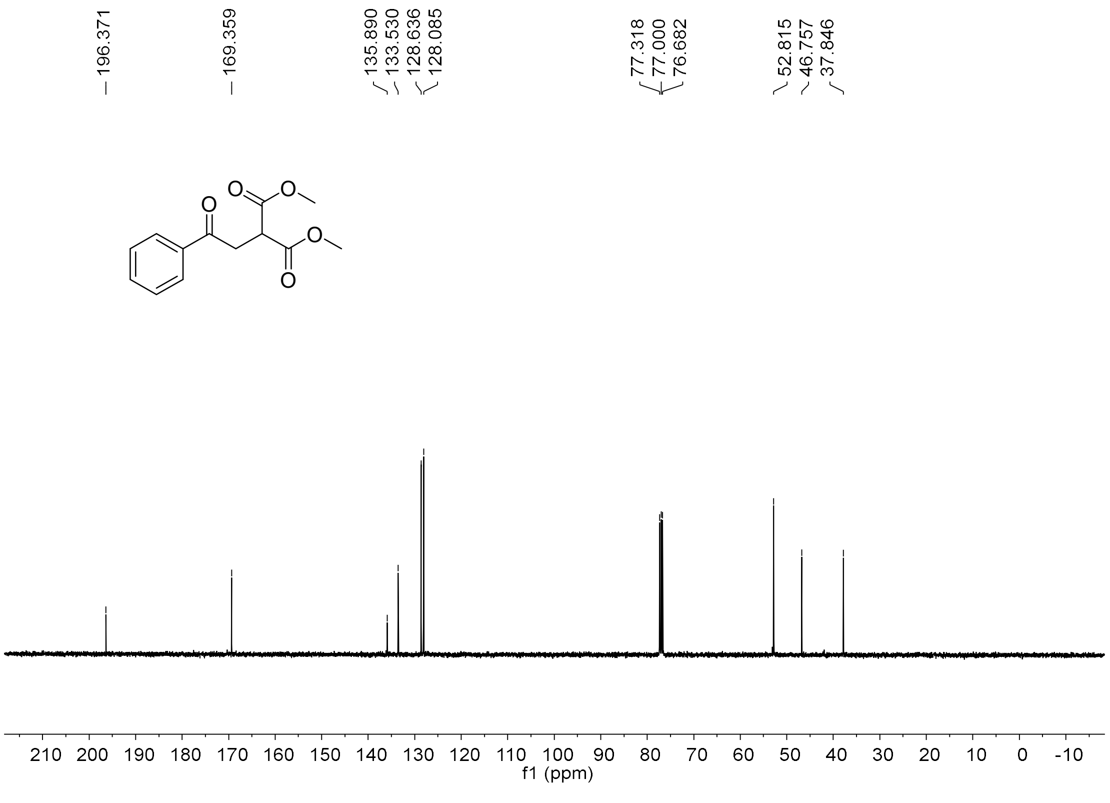

400 MHz ^{1}H NMR Spectrum of 53 in $CDCl_3$

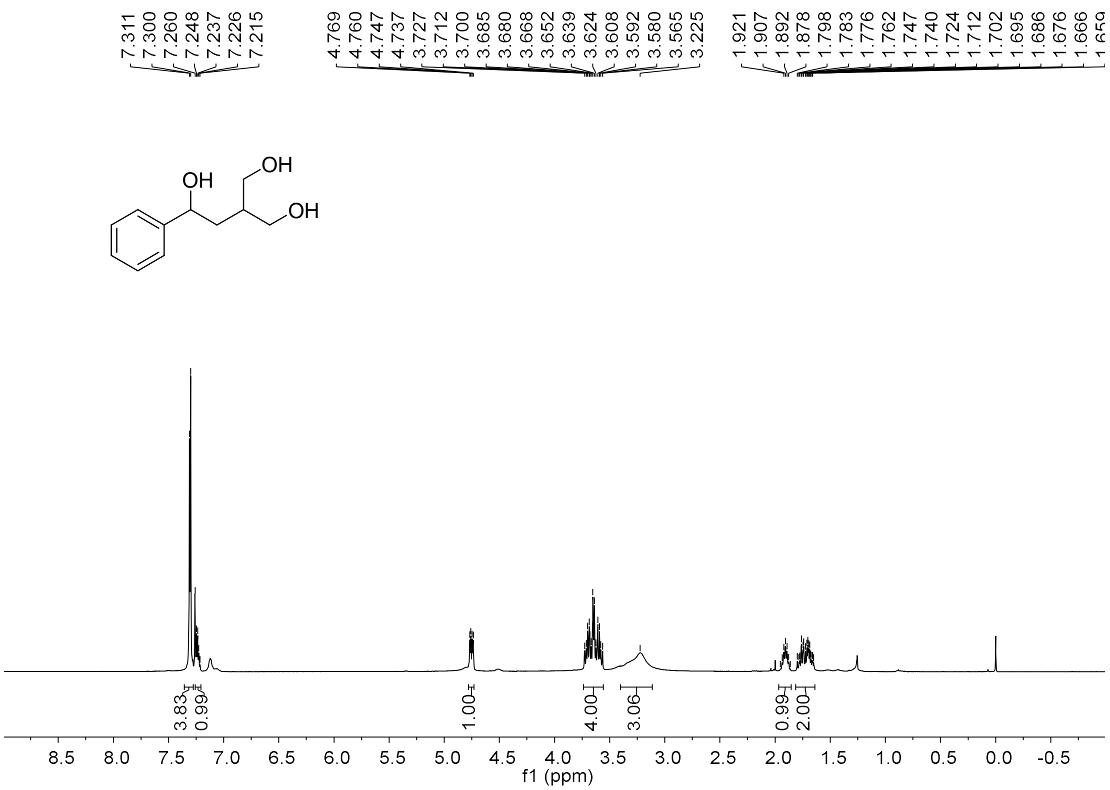

100 MHz ^{13}C{1H} NMR Spectrum of 53 in $CDCl_3$

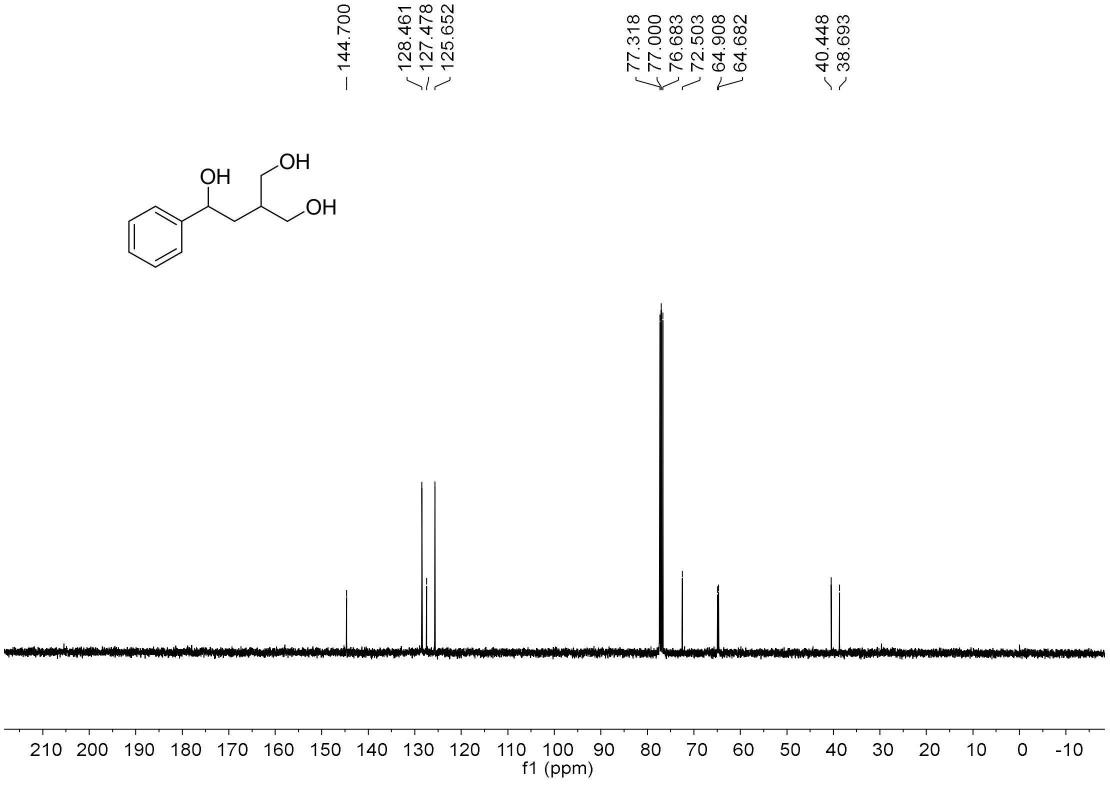

400 MHz ^{1}H NMR Spectrum of 54 in $CDCl_3$

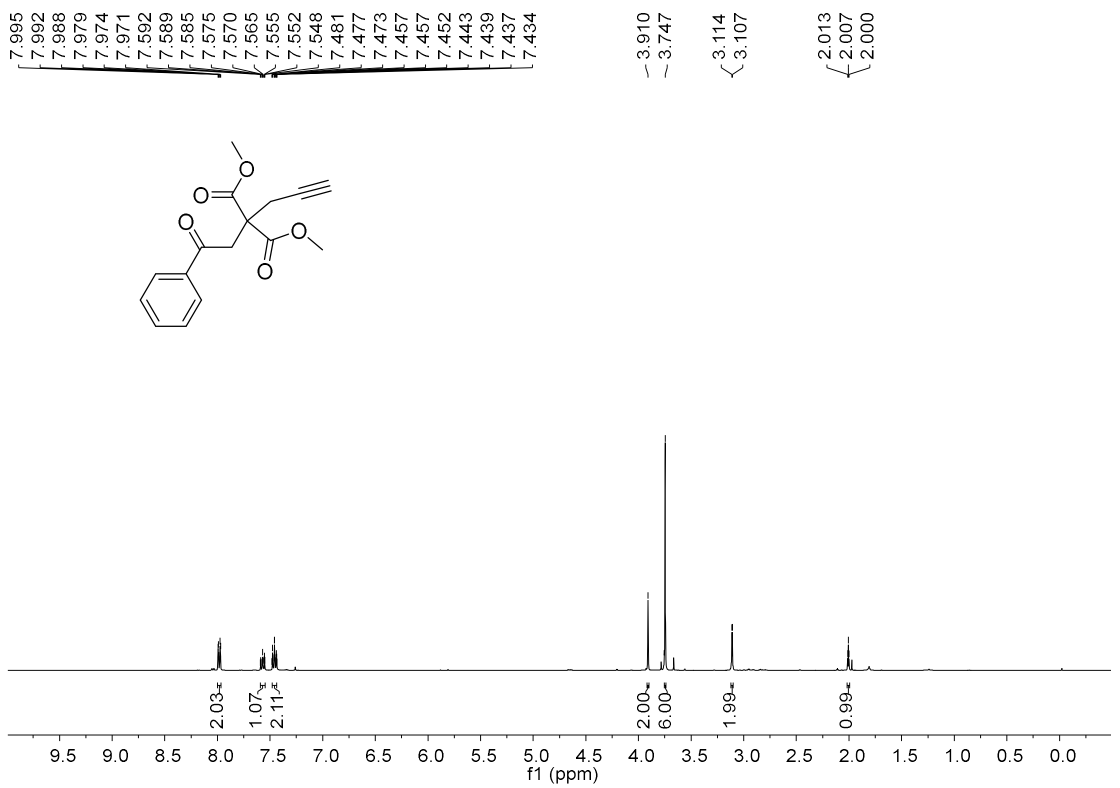

100 MHz ^{13}C{1H} NMR Spectrum of 54 in $CDCl_3$

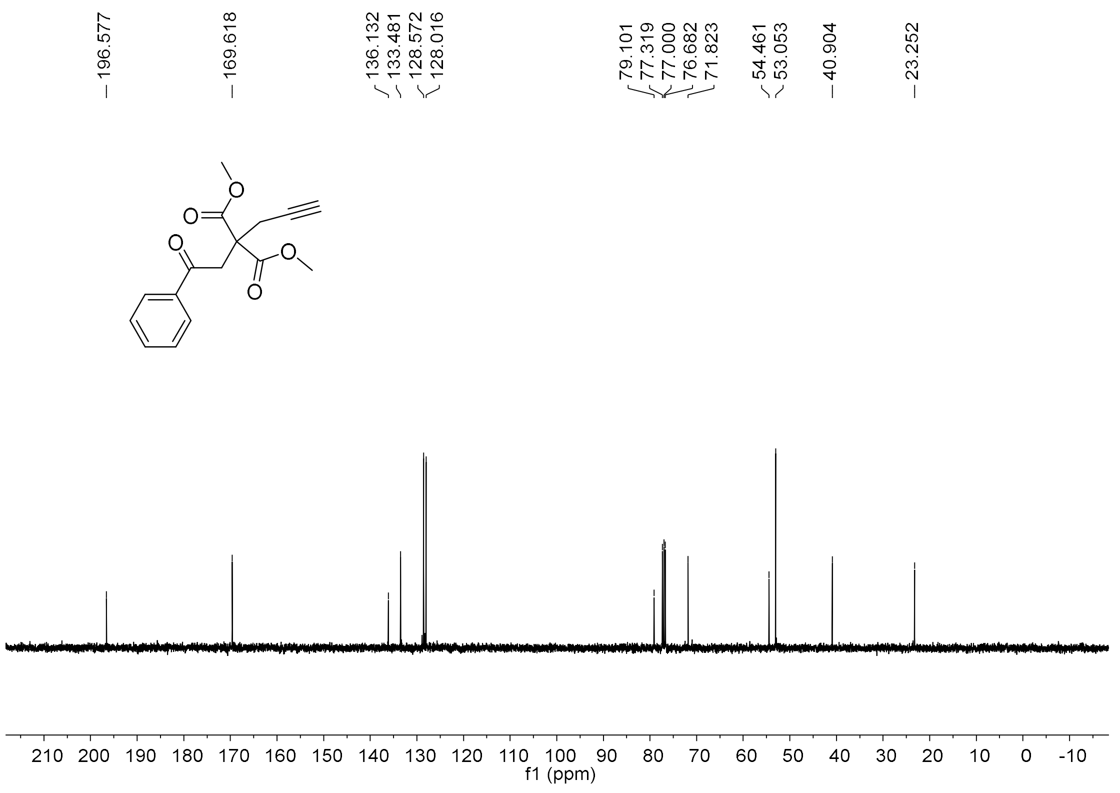

400 MHz ^{1}H NMR Spectrum of 55 in $CDCl_3$

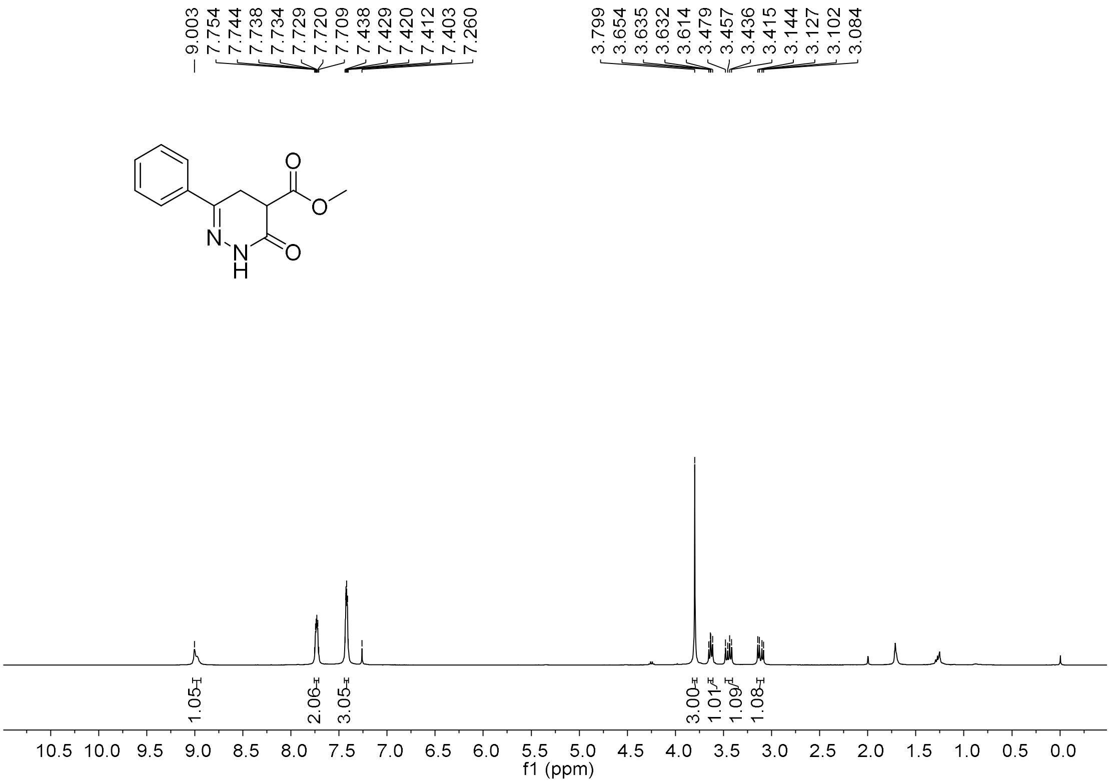

100 MHz $^{13}C\{1H\}$ NMR Spectrum of 55 in $CDCl_3$

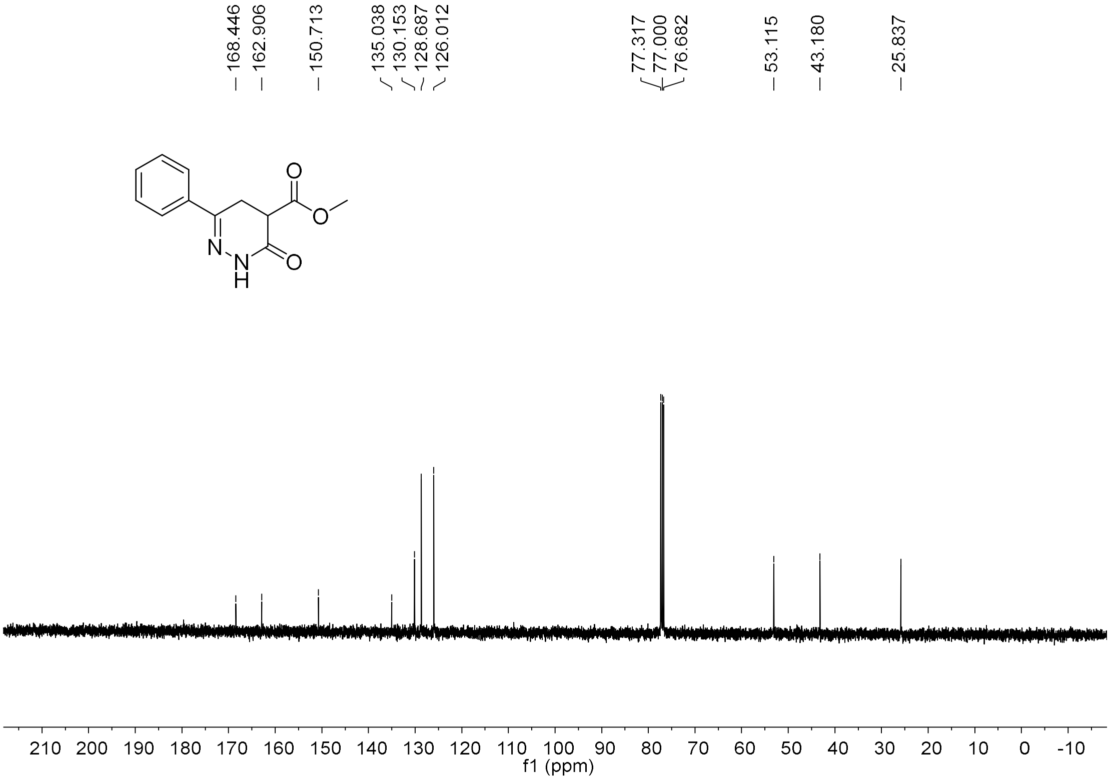

400 MHz ^{1}H NMR Spectrum of 56 in $CDCl_3$

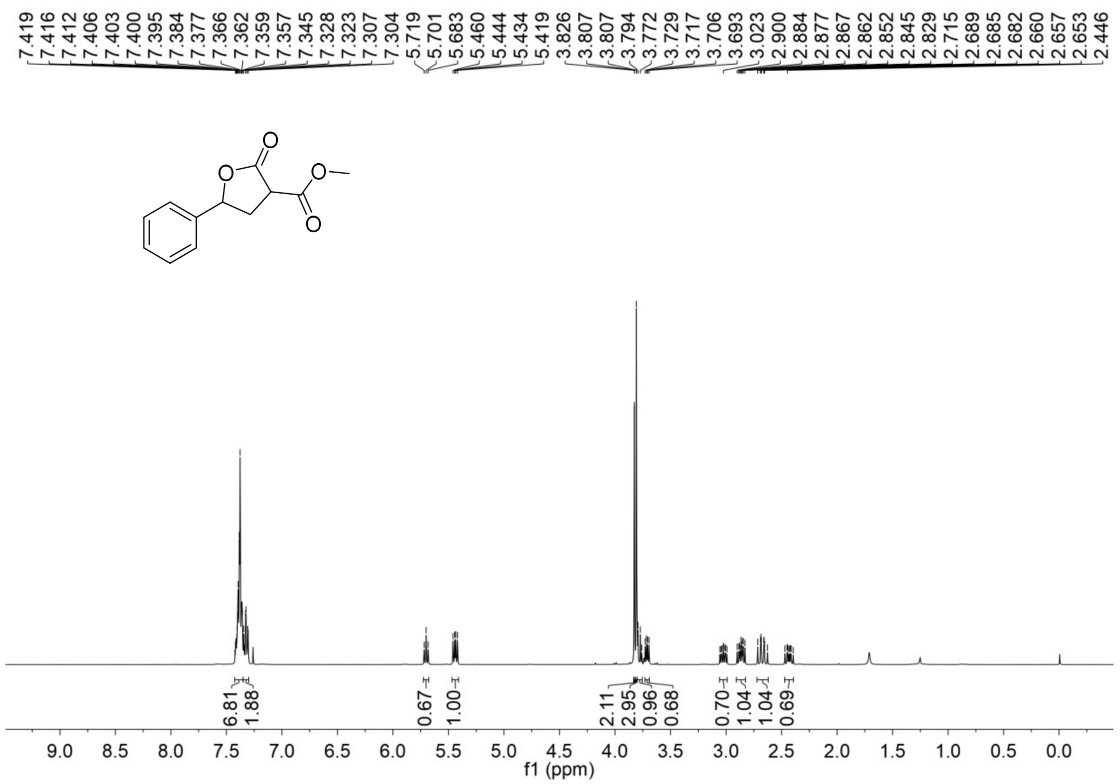

100 MHz ^{13}C{1H} NMR Spectrum of 56 in $CDCl_3$

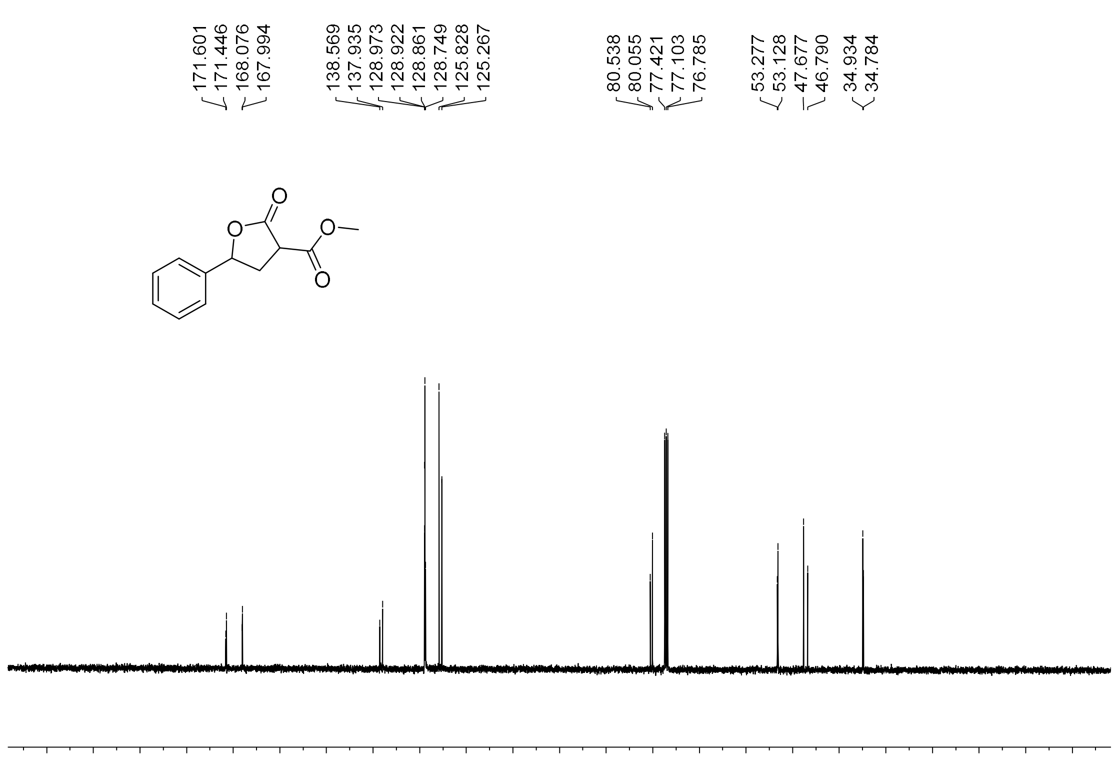

Supplement: Supplementary file 1 — Supporting Information [file ADVS-11-2307633-s001.pdf]
